# Synthesis of Azaisoindolinones by Heterogeneous Catalyzed Regioselective Hydrodeoxygenation of *N*‑Heteroaromatic Phthalimides

**DOI:** 10.1021/acssuschemeng.5c05996

**Published:** 2025-07-30

**Authors:** Carles Lluna-Galán, Luis Izquierdo-Aranda, Patricia de la Iglesia-Gómez, Camille Béchet, Pau Vidal-Puyuelo, Juan Camilo Arango-Daza, Rosa Adam, Jose R. Cabrero-Antonino

**Affiliations:** ∥ Instituto de Tecnología Química (UPV-CSIC), Universitat Politècnica de València-Consejo Superior de Investigaciones Científicas, Av. de los Naranjos s/n, València 46022, Spain; ¶ Departament de Química Orgànica, Facultat de Farmàcia, Universitat de València, Av. Vicent Andrés Estellés s/n, Burjassot, València 46100, Spain

**Keywords:** Regioselective Hydrogenation, Bimetallic Supported Catalysis, Silver, Rhenium, Azalactams

## Abstract

The development of efficient and sustainable methods for producing azalactams, pivotal compounds in medicinal chemistry, is a relevant topic in fine chemistry. Here, we present a heterogeneous catalytic protocol to synthesize azaisoindolinones via the hydro-deoxygenation of azaphthalimides, producing water as the only byproduct. Notably, the [AgRe/Al_2_O_3_] nanocatalyst promotes the desired transformation, showing full tolerance to the pyridine ring, a substantial improvement in heterogeneous catalysis, and total regioselectivity. The Ag–Re nanocatalyst efficiency is explained by a Ag–Re cooperative effect for which the presence of an equimolar ratio of both metals in the material is key.

## Graphical abstract

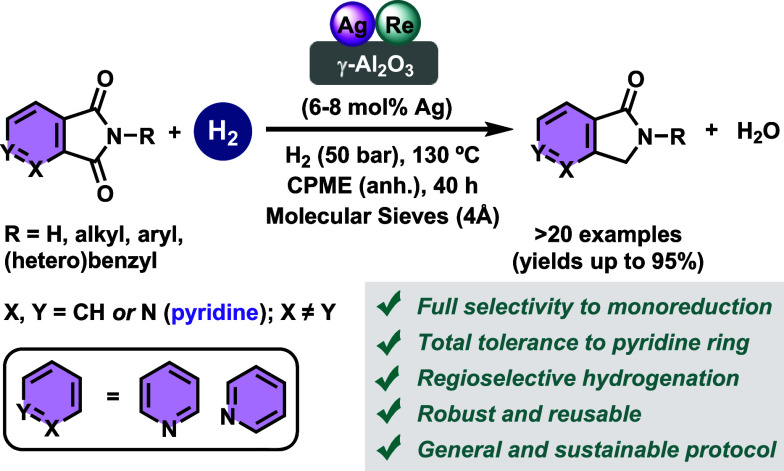

## Introduction

*N*-Heterocycles are extremely relevant compounds for the pharmaceutical industry due to their important biological activities and, hence, their high potential as drugs. Moreover, these scaffolds are very frequently present in the structure of natural products and, in general, they are very common structures in important fine chemicals. Therefore, the development of sustainable methods for their synthesis involving the use of heterogeneous catalysis and green reagents is a topic of interest nowadays.^1–4^ Among *N*-heterocycles, azaisoindolinones are an important type of isoindolinone derivative containing a pyridine ring fused with a pyrrolidone fragment. This kind of *N*-heterocyclic lactam motif is present in a wide range of biologically active substrates, especially in those modulating acetylcholine metabolism and biological functions and, hence, presenting utility in the treatment of diseases such as Alzheimer or schizophrenia.^5–7^ In fact, T-82 (Scheme 1, A) has been reported to be an acetylcholinesterase inhibitor with a great potential to treat Alzheimer’s disease.^8^ On the other hand, VU0453595 ^9^ and VU0550164 ^10^ (Scheme 1, A) are positive allosteric modulators of M_1_ subtype muscarinic acetylcholine receptor with utility in the treatment of schizophrenia and Alzheimer’s disease.^11,12^ Moreover, azaisoindolinones with other kinds of biological activity have been described lately, such as several inhibitors of phosphoinositide 3-kinases (PI3Ks) with utility as drugs in autoimmune disorders, including multiple sclerosis (Scheme 1, A).^13,14^ Thus, considering the large interest in these compounds, the organic chemistry community has devoted efforts to develop diverse strategies for the synthesis of azalactams. In recent years, great advances have been made such as the development of multistep organic procedures^5–7,15–19^ and protocols employing structurally specific presynthesized substrates^20,21^ for producing azaisoindolinones and related lactams. In most of these examples, stoichiometric reagents or homogeneous catalysts are required.

**Scheme 1:**
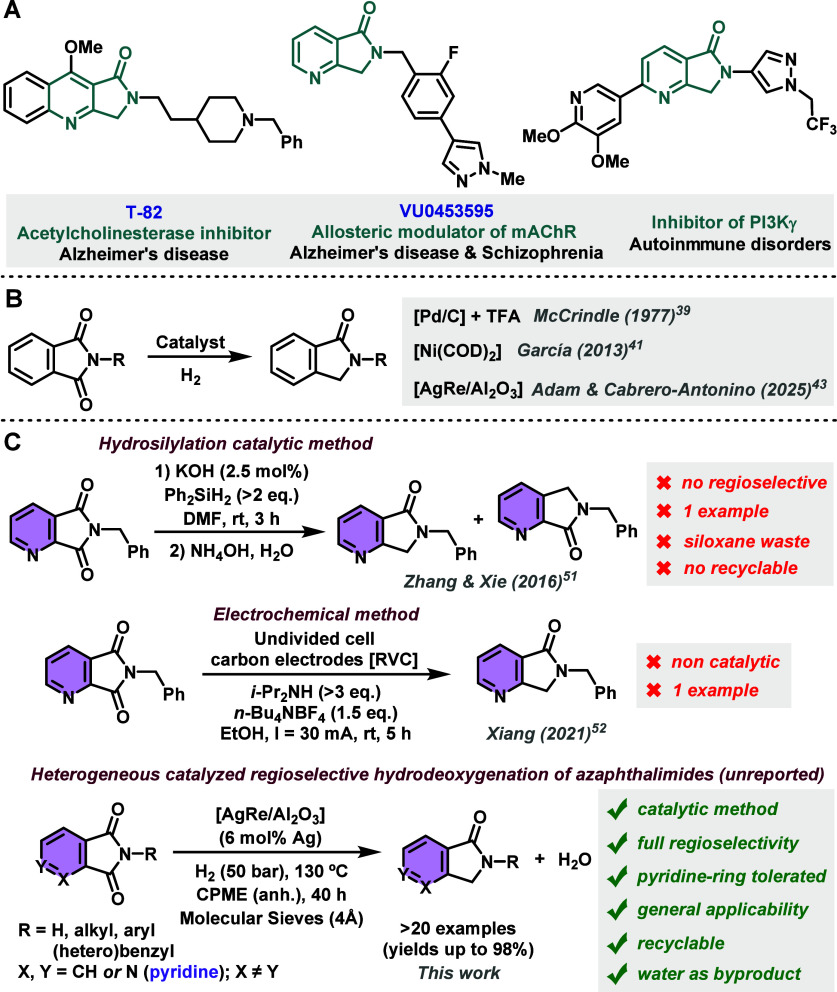
(A) Catalytic Hydrodeoxygenation of Phthalimides to Isoindolinones; (B) Examples of Biologically Active Azaisoindolinones; (C) Reduction of Azaphthalimides to Azaisoindolinones^a^

a Reported examples and this work. mAChR = muscarinic acetylcholine receptor. PI3Kγ = phosphoinositide 3-kinase γ complex.

In the current context in which reaching high levels of sustainability in the synthesis of relevant compounds, such as azaisoindolinones, is mandatory,^22–24^ strategies employing hydrogen and reusable catalysts are very interesting to explore. Thus, reactions like the hydrodeoxygenation (HDO) of carboxylic acid derivatives, for example, imides, esters, or amides, have emerged as powerful tools toward the synthesis of value-added compounds, only producing water as the sole byproduct.^25–30^ However, owing to its chemical stability and the poorly electrophilic character of the carbonyl group in these derivatives, its hydrogenative activation constitutes a large challenge.^31^ Despite this difficulty, the wide accessibility, low price, and robustness of carboxylic acid derivatives make their employment as starting materials very interesting for both industry and academia. In the last two decades, a successful strategy for the hydrodeoxygenation (HDO) of carboxylic acid derivatives has been built on the basis of the design of bimetallic nanomaterials containing a transition metal presenting an oxophilic character, and, hence, being able to activate the carbonyl group, combined with another metal capable of promoting H_2_ activation/dissociation.^25–30^ Hydrodeoxygenation of amides to amines using [PdRe/Graphite][Bibr ref32] or [PtV/HAP][Bibr ref33] and [PtMo/ZrO_2_]-catalyzed ester-to-ether hydrogenation^34^ constitute elegant examples following this approximation, among others.^25–30^

More specifically, in the hydrogenation of phthalimides to selectively achieve isoindolinones through a HDO reaction, an additional difficulty, apart from the inertness of the carbonyl group, is encountered related to selectivity.^28,29,35^ Phthalimides hydrogenation can potentially afford at least five possible products, only considering the possibilities of a partial hydrogenation^36–38^ or a hydrogenation following either a C–O^39–43^ or C–N^38,44–48^ pathway in one or both carbonyls. Therefore, to date, only three catalytic procedures have been described for the hydrogenation of phthalimides to isoindolinones (Scheme 1, B). Originally, the group of McCrindle demonstrated in 1977 that [Pd/C] catalyst in strong acidic conditions (trifluoroacetic acid) constitutes an active catalytic system for this process.^39^ In 2013, García and co-workers employed the [Ni­(COD)_2_] complex as a catalyst for the HDO of phthalimide to isoindolinone as the only substrate studied.^41^ Finally, this year, our group developed the first general catalytic protocol to promote the hydrodeoxygenation of a wide range of cyclic imides (mainly phthalimides) to lactams making use of a bimetallic supported [AgRe/Al_2_O_3_] nanomaterial (Scheme 1, B).^43^

As mentioned before, the development of direct and sustainable methodologies for synthesizing azaisoindolinones is a topic of interest. In this sense, the C–O hydrogenation or HDO of a single carbonyl of the corresponding azaphthalimide is an interesting approach that, to date, has not been reported. It is important to consider that, apart from the previously described challenges related to the hydrogenation of phthalimides, in the case of azaphthalimides containing a pyridine ring, additional selectivity problems can be found. On the one hand, pyridine, an electron poor heterocycle in comparison with benzene, presents a higher tendency for its hydrogenation. On the other hand, these cyclic imides are *per se* unsymmetrical phthalimides, in which both C=O groups can be distinguished, introducing another difficulty related to the control of the regioselectivity.^28,43^ Potentially, the pyridinic nitrogen atom could act as a directing group to selectively obtain one azalactam regioisomer.

Direct Clemmensen reductions of azaphthalimides with (over)­stoichiometric reagents such as Zn or Sn metal in the presence of strong acids have been described.^49,50^ Obviously, although these are practical methodologies, they are not desirable, as they generate a huge amount of waste. In addition, another two methodologies have been reported for the synthesis of azalactams through the direct reduction of azaphthalimides (Scheme 1, C). In 2016, Zhang, Xie, and co-workers developed a synthetic route to obtain one *N*-benzyl azaisoindolinone employing KOH as catalyst and an (over)­excess of Ph_2_SiH_2_ as the hydride source.^51^ This was not a general protocol; a huge amount of siloxane waste was generated in the process, and the reaction did not show regioselectivity. Five years later, a noncatalytic electrochemical method using an undivided cell with carbon electrodes was developed by the group of Xiang.^52^ By using this protocol, which requires a graphite anode and a reticulated vitreous carbon (RVC) cathode separated by an insulator, only one example of azalactam was obtained with full regioselectivity (Scheme 1, C).

In this work, our purpose is to study the potential application of our previously designed nanostructured [AgRe/Al_2_O_3_] solid nanocatalyst^43^ for promoting the regioselective hydrodeoxygenation of azaphthalimides to the corresponding azaisoindolinones with full preservation of the pyridine ring (Scheme 1, C). Our hypothesis to select this nanocatalyst is based on the fact that neither Ag nor Re are metals with a known tendency to hydrogenate aromatic rings. Moreover, we have previously shown that the material composed of a Ag–Re combination in an equimolar ratio over γ-Al_2_O_3_ demonstrated a remarkable bimetallic cooperativity for the selective hydrogenation of cyclic imides to lactams. In fact, we showed that Ag was very relevant to catalyze the first imide hydrogenation to the corresponding hemiamidal, and Re, due to its oxophilicity and large number of oxidation states, was key to promote the second hydrogenation step to afford the lactam, in cooperation with Ag. Therefore, in this Letter, we present the application of [AgRe/Al_2_O_3_] as a robust and reusable nanocatalyst able to produce azalactams via the regioselective hydrodeoxygenation of azaphthalimides, generating water as the only byproduct. Notably, the existence of supported AgReO_4_ nanoparticles on the alumina matrix as precatalytic sites that, under the reaction conditions, give a place to the active catalytic sites formed by Ag^0^ nanoparticles and highly dispersed ReO_*x*_ species in the material, is key for achieving an active and regioselective nanocatalyst in which the Ag–Re cooperativity results are optimal.

## Results and Discussion

As a starting point of our investigation, we selected, as the benchmark reaction, the hydrogenation of 6-benzyl-5*H*-pyrrolo­[3,4-*b*]­pyridine-5,7­(6*H*)-dione **1** in the presence of the previously reported^43^ [AgRe/Al_2_O_3_] nanomaterial (6 mol % Ag vs **1**) using anhydrous cyclopentyl methyl ether (CPME) as solvent. [AgRe/Al_2_O_3_] was prepared via wet impregnation followed by calcination, and Ag and Re contents were determined by ICP-AES to be 4.5 wt % of Ag and 7.5 wt % of Re, corresponding to an equimolar Ag:Re ratio (for more details, see Supporting Information Section 2.1). First, we studied the hydrogenation of **1** using the AgRe catalyst (6 mol % Ag) at 50 bar of H_2_ and three different temperatures (100, 130, and 150 °C) over 20 h (Table 1, entries 1–3). In the three reactions, a mixture of the azaisoindolinone **2** ^50,51^ and hemiamidal **3**,^50,53,54^ both coming from the regioselective hydrogenation of the more proximal carbonyl to the pyridinic nitrogen, were obtained as major products; traces of the C–N hydrogenation product **4** with full regioselectivity were also obtained.^44,55^ To our delight, at 150 °C, azaisoindolinone **2** was obtained in up to 80% yield and 80% selectivity with full conversion of **1** (Table 1, entry 3). Notably, any product coming from the pyridine ring reduction at azaimide **1** was observed. With the aim to improve product **2** yield, we conducted a catalytic test at higher H_2_ pressure, 70 bar, and 150 °C, but unfortunately, these conditions were detrimental for the formation of **2**, as its yield decreased to 62% (Table 1, entry 4). In addition, at these conditions, the formation of **4**, coming from C–N hydrogenation, increased (11%) and compound **5**, formed as a consequence of a concomitant hydrodeoxygenation of one C=O bond and a pyridine ring reduction, was detected in 17% yield for the first time. Then, the reaction at 130 °C and 50 bar of H_2_ in the presence of higher catalyst loadings (8 mol % Ag) was attempted. Although **2** was obtained in a higher yield than in the case of the reaction at the same conditions but at lower catalyst loadings (65% vs 84%, Table 1, entries 2 and 5), it was not a substantial improvement in comparison with the reaction performed at 150 °C and 6 mol % Ag (80%, Table 1, entry 3). Subsequently, we evaluated the influence in the catalytic process of longer reaction times at 130 and 150 °C under 50 bar of H_2_ and 6 mol % of Ag (Table 1, entries 6 and 7). Gratifyingly, at 130 °C and after 40 h, a quantitative yield of desired azaisoindolinone **2** (98% yield) was afforded with excellent regioselectivity and complete conversion of azaimide **1**. Moreover, a catalytic experiment was performed under the same reaction conditions but in the absence of molecular sieves, giving place to low conversions and yields of azaisoindolinone **2** (Table 1, entry 8). Hence, this confirmed the key role of molecular sieves in the reaction to absorb water and shift the equilibrium toward the azaisoindolinone **2** formation. Finally, the use of the optimized reaction conditions (50 bar of H_2_ and 130 °C) but applying a lower reaction time (30 h) or catalyst loading (4 mol % Ag) was detrimental for the catalytic activity of the system, since conversion of **1** and/or yield of **2** diminished (Table 1, entries 9 and 10, in comparison with entry 7).

**Table 1:**
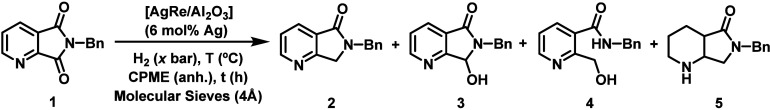
Optimization of Reaction Conditions in the [AgRe/Al_2_O_3_]-Catalyzed Hydrogenation of *N*-Benzyl Azaphthalimide **1**

Entry^a^	H_2_ (bar)	*T* (°C)	*t* (h)	Conv. **1** (%)^b^	**2** (%)^b^	**3** (%)^b^	**4** (%)^b^	**5** (%)^b^	Sel. **2** (%)^b^
1	50	100	20	75	30	41	3	n.d.	40
2	50	130	20	91	65	20	4	n.d.	71
3	50	150	20	>99	80	14	3	n.d.	80
4	70	150	20	98	62	9	11	17	63
5^c^	50	130	20	>99	84	2	5	7	83
6	50	150	40	>99	89	n.d.	5	4	89
**7**	**50**	**130**	**40**	**>99**	**98**	**n.d.**	**1**	**1**	**98**
8^d^	50	130	40	35	33	n.d.	1	1	93
9	50	130	30	>99	85	11	1	1	85
10^e^	50	130	40	80	77	n.d.	1	1	97

a Standard reaction conditions: azaphthalimide **1** (0.125 mmol), [AgRe/Al_2_O_3_] (6 mol % Ag, 4.5 wt % Ag in the material), H_2_ (50 bar), anh. CPME (1 mL), *n*-dodecane (20 μL), 4 Å molecular sieves (40 mg, previously dried under vacuum at 300 °C for 3 h) at the indicated temperature during the denoted time. n.d. = not detected. Bn = benzyl.

b Conversion of **1** and yield of products **2**, **3**, **4**, and **5** and selectivity to **2** were calculated by GC using *n*-dodecane as internal standard. In selected cases, regioselectivity obtained in the different hydrogenated products detected (**2** to **5**) was confirmed by ^1^H NMR analysis by using 1,3,5-trimethoxybenzene as external standard and DMSO-*d*_6_ as deuterated solvent.

c Run with 8 mol % Ag.

d Run without molecular sieves.

e Run with 4 mol % Ag.

Then, we decided to evaluate the solvent effect in the catalytic behavior of [AgRe/Al_2_O_3_] for the hydrogenation of **1** in the presence of 6 mol % Ag at 130 °C, 50 bar of H_2_ for 20 h (Table S1). To this end, we compared the effect of several aliphatic solvents such as *n*-heptane, ether-type solvents, including 2-Me-THF and 1,4-dioxane apart from CPME, arene-type solvents (mesitylene and toluene), and a protic solvent (methanol) (Table S1). Remarkably, under the specific reaction conditions tested, CPME was the solvent that afforded the best yield of azalactam **2** (65%, Table S1, entry 1). It is remarkable that *n*-heptane, 1,4-dioxane, and toluene also afforded compound **2** in moderate to good yields and selectivities (40%, 57%, and 45%, Table S1, entries 2, 4, and 6). In contrast, 2-Me-THF and methanol showed low conversions and selectivities to the desired compound **2** (Table S1, entries 3 and 7), while mesitylene afforded higher conversions but with an important decrease in the selectivity toward azalactam **2** and the significant observation of product **4**, coming from a C–N hydrogenolysis, in a 15% yield (Table S1, entry 5).

At this point, we were interested in better understanding the relationship between the catalyst composition and structure with the Ag–Re cooperativity and metal determination of regioselectivity (Table S2). With this aim, we evaluated the catalytic activity of the monometallic nanomaterials [Ag/Al_2_O_3_] and [Re/Al_2_O_3_] in the benchmark reaction at the optimized conditions of 20 h at 6 and 12 mol % of the corresponding metal (Table S2, entries 2–5). In the case of the reaction conducted using [Ag/Al_2_O_3_] as catalyst at 6 mol %, the corresponding hemiamidal **3** was the major product (53%, Table S2, entry 2),^50,51,53,54^ while at 12 mol % a mixture of **3** and **4**, coming from C–N hydrogenation, was detected (Table S2, entry 3). However, when the reaction was performed with [Re/Al_2_O_3_], a very low catalytic activity was observed and only very low yields of azalactam **2** were detected (Table S2, entries 4 and 5). It is known that Re monometallic catalysts can be potentially active for hydrogenation of carboxylic acid derivatives when they are prereduced.^43^ Hence, we explored the result of the test reaction employing a previously reduced [Re/Al_2_O_3_] at 280 °C; albeit in these cases, there was only a slight increase of the catalytic activity at 12 mol % (Table S2, entries 6 and 7). Therefore, these results clearly demonstrate that the cooperativity between Ag and Re is key for selectively obtaining the azalactam product and also suggest that Ag is in charge of the first imide hydrogenation to the hemiamidal, while Ag–Re cooperation is needed in the second step. Moreover, regarding regioselectivity, it can also be deduced from these results that both metals, Ag and Re, potentially coordinate with the pyridinic nitrogen to drive the selectivity to the hydrogenation of the proximal carbonyl. In addition, it is also interesting to note that, when the [Ag/Al_2_O_3_] and [Re/Al_2_O_3_] mixture was evaluated, the catalytic activity was clearly diminished with respect to [AgRe/Al_2_O_3_], confirming the key relevance of Ag–Re intimate contact (Table S2, entry 8).

Moreover, it was also interesting to evaluate the catalytic activity of other Ag–Re nanomaterials with different molar ratios. Thus, the hydrogenation of azaphtalimide **1** was tested at the optimized reaction conditions during 20 h using [AgRe_0.35_/Al_2_O_3_] (4.5 wt % Ag and 2.7 wt % Re) and [AgRe_2_/Al_2_O_3_] (4.1 wt % Ag and 14.0 wt % Re) as catalysts (Table S2, entries 9 and 10); Ag:Re molar ratios of 2 and 0.35, respectively. It has been demonstrated that, while the nanomaterial with equimolar Ag:Re ratio is composed of AgReO_4_ nanoparticles, AgRe_0.35_ material is composed of Ag_2_O nanoparticles and Re^7+^ oxides highly dispersed on the surface of the support and with a perrhenate geometry, and AgRe_2_ material is formed by AgReO_4_ species together with Re^7+^ oxides of perrhenate geometry.^43^ Under reaction conditions, implying H_2_ pressure and temperature, these materials became activated, and their composition is much more similar as they are all composed of Ag^0^ nanoparticles and ReO_*x*_ highly dispersed clusters. However, the precatalyst structure has a strong influence on the efficiency of the Ag–Re intimate contact, and the electronic and structural properties of the metallic species in the reduced materials present the optimal features when the bimetallic equimolar material is applied as catalyst for the hydrogenation of cyclic imides.^43^ In fact, for the hydrogenation of our azaphtalimide **1**, this was also the case, as AgRe_0.35_ and AgRe_2_ materials were demonstrated to be less active and selective than the AgRe nanocatalyst (Table S2, entries 9 and 10).

To gain more insight in the process, a yield–time kinetic profile was registered (Figure S1) at the optimized reaction conditions and using a prereduced version of [AgRe/Al_2_O_3_] to avoid a possible induction period caused by the catalyst hydrogenative activation.^43^ At these conditions, azahemiamidal **3** was detected at relatively short reaction times (25–30% yield at 1 h), confirming that this compound represents a central intermediate in the formation of azaisoindolinone **2**, as expected. Azahemiamidal **3** reached its maximum yield (41%) at around 4 h, indicating that its hydrogenative dehydration is not a fast process.

With the aim of investigating the heterogeneous nature of the AgRe bimetallic nanostructured system in the regioselective hydrodeoxygenation of **1** to **2**, a kinetic filtration test study was performed (Figure S2). This experiment confirmed that, after catalyst removal at 3 h, no increase in the yield of **2** was detected (35% yield of **2** was maintained up to the end). Moreover, ICP-AES analysis of the metal content present at the filtrated material after being used as catalyst for 20 h demonstrated its stability (see Supporting Information, Section 4.2). In addition, metal content in the filtrated reaction mixture coming from solid lixiviation was also discarded. Therefore, considering these results, we explored the potential reusability of [AgRe/Al_2_O_3_] nanocatalyst in the benchmark reaction (hydrodeoxygenation of **1** to **2** at 130 °C, 50 bar of H_2_, and 6 mol % Ag over 20 h). To remove organic residue and promote catalyst reactivation, we applied a calcination step to the material under an air flow at 300 °C for 3 h among each successive reaction cycle. Gratifyingly, [AgRe/Al_2_O_3_] catalyst could be used for five consecutive cycles without apparent loss of efficiency (68% yield of **2** at the first cycle to 65% at the fifth cycle, Figure S3). However, a loss of catalyst efficiency was detected at the sixth cycle of the reaction since a 47% yield of **2** was reached in this case.

Once the excellent robustness and stability of the [AgRe/Al_2_O_3_] nanocatalyst were demonstrated, we wanted to evaluate the effect of reaction conditions in the structure of the bimetallic nanocatalyst. Hence, we characterized both, the recovered and reactivated [AgRe/Al_2_O_3_] systems, by XRPD and UV–vis DRS (Figure S4 and S5). Interestingly, both techniques detected the presence of Ag^0^ nanoparticles^56–59^ in the used materials, which disappeared after calcination treatment at 300 °C. Complementarily, XRPD analysis of the recovered and then calcined material confirmed the regeneration of AgReO_4_ species^60,61^ after the reactivation step (Figure S4).

Considering all these observations, a plausible reaction mechanism for the regioselective hydrodeoxygenation of **1** to **2** using the [AgRe/Al_2_O_3_] nanocatalyst was proposed (Scheme 2). The initial step would entail the activation of the precatalytic nanomaterial, consisting of the dispersive hydrogenation of supported AgReO_4_ aggregates to Ag^0^ nanoparticles and highly dispersed ReO_*x*_ nanoclusters of high and low oxidation states.^43^ The next step involves the monohydrogenation of azaphthalimide **1** to azahemiamidal **3**, an intermediate detected at short reaction times (Figure S1). This is proposed to be mediated through the Ag^0^ catalyzed activation/dissociation of H_2_ in cooperation with both ReO_*x*_ oxophilic species or γ-Al_2_O_3_ acid sites able to promote C=O group coordination/activation.^43^ The involvement of ReO_*x*_ species in H_2_ activation in this step does not seem probable, as the [Re/Al_2_O_3_] prereduced catalyst showed very low catalytic activity. Taking into account the regioselectivity studies, here, we propose that both Ag and Re centers coordinate with the pyridinic nitrogen atom of **1**, hence driving the selectivity toward the reduction of the C=O group proximal to the pyridinic nitrogen.

**Scheme 2:**
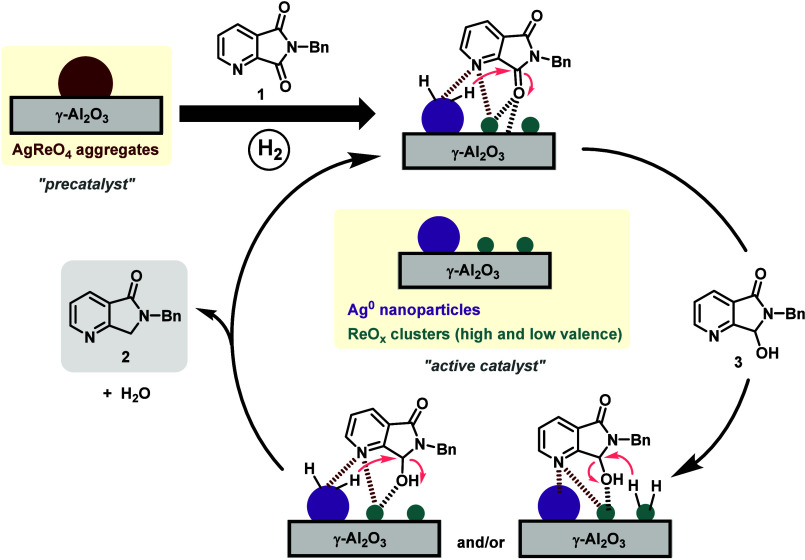
Proposed Reaction Mechanism for the [AgRe/Al_2_O_3_]-Catalyzed Regioselective Hydrodeoxygenation of Azaphthalimide **1** to Azaisoindolinone **2**

Subsequently, the C–OH hydrogenolysis of the azahemiamidal intermediate **3** to give azalactam **2** is proposed to occur by two possible pathways. In all of the possible routes, the role of Re is key, as [Ag/Al_2_O_3_] is not able to mediate this step by itself. One main path would involve a cooperative phenomenon in which Ag^0^ species activate H_2_ and ReO_*x*_ nanoclusters promote OH elimination by protonation/dehydration and water formation. In this sense, the stronger acidity of ReO_*x*_, in comparison with Ag^0^ species deposited on alumina, is key for performing such a dehydration step. The other mechanism proposes the hydrogen activation process to take place in low valent rhenium oxide nanoclusters (ReO_*x*_).^43^

In view of the good catalytic performance and robustness of [AgRe/Al_2_O_3_] nanomaterial for promoting the regioselective hydrodeoxygenation of **1** to **2**, at this stage, we became interested in exploring the synthetic applicability of our protocol for mediating the hydrodeoxygenation of several azaphthalimides (see Sections 2.3 and 2.4 of the Supporting Information for specific detailed information about its preparation) to the desired azaisoindolinones (Scheme 3). Under standard reaction conditions, *N*-benzyl azalactam **2** could be isolated in 95% yield. Note that, in almost all the other compounds involved in the substrate scope study, catalyst loading employed had to be increased up to 8 mol % Ag in order to improve azaphthalimide conversion and azalactam yields. Thus, a series of *N*-benzyl azalactams bearing diverse electron-donating and electron-withdrawing substituents (including −OMe, −OPh, −OCF_3_, −CF_3_, −F, −Cl, and −CF_3_) placed in the *ortho*, *meta*, and *para* positions of the benzene ring afforded the desired azalactams **6**–**13** in 67–90% isolated yields and with full regioselectivity (Scheme 3). Complementarily, three different disubstituted *N*-benzyl azalactams **14**–**16** containing either two F atoms in *ortho* positions, two CF_3_ groups in *meta* positions, or a F and Br in *ortho* and *para* positions, respectively, were produced in 53–83% isolated yields. Notably, azalactam **16** with a Br-substituted position, which is typically labile under hydrogen conditions, was obtained without any stability issues. It is important to note that **16** has been employed as a precursor of bioactive azalactam molecules^5^ by using a Suzuki coupling with *N*-methylpyrazol- or *N*-methylindazol-pinacol boronic esters (Scheme 1). Moreover, a variety of azalactams **17**–**20** featuring the nitrogen atom of the pyrrolidone fragment with naphthalene, 1,3-benzodioxolane, furan, or thiofuran aromatic ring substitution, were also accessed with moderate to very good isolated yields (42–79%). Thus, the synthesis of such chemically complex azalactams shows the interesting synthetic application of our developed catalytic strategy. In addition, *N*-phenyl as well as a variety of *N*-alkyl azaphthalimides, including primary (*n*-butyl, isopropyl, and cyclohexyl) and secondary alkyl-type groups at the nitrogen atom of the azaimide, were converted into the desired azalactams **21**–**24** in 40–79% yield. Remarkably, in the case of *N*-phenyl azalactam **21**, a small amount of aniline as additive (0.2 equiv vs azaimide) was required. This was needed to avoid the C–N hydrogenolysis pathway, occurring in a notable extension given the poor nucleophilic character of aniline in comparison with benzylamine. Interestingly, azalactam **25** presenting a biological active fragment^8^ was accessed in 70% yield. Finally, we explored our protocol toward the hydrogenation of an azaphtalimide with the pyridinic nitrogen atom placed in a different position, more distal from the carbonyl group (isopyridine ring). In this case, the corresponding azalactam **26** was obtained with 78% isolated yield and as an inseparable mixture of regioisomers in a 86:14 ratio. The exact nature of the major and minor regiosomer was unequivocally confirmed by NMR analysis employing HSQC, HMBC, and NOESY ^1^H–^1^H bidimensional experiments (see Section 8 of the Supporting Information). Certainly, this result was very interesting since it demonstrates that, if the nitrogen atom present at the pyridine fragment of the azaimide is placed in a far position, then the regioselectivity is slightly compromised. Therefore, this confirms the strong directing group effect that the pyridinic nitrogen atom plays in the regioselectivity of the overall process.

**Scheme 3:**
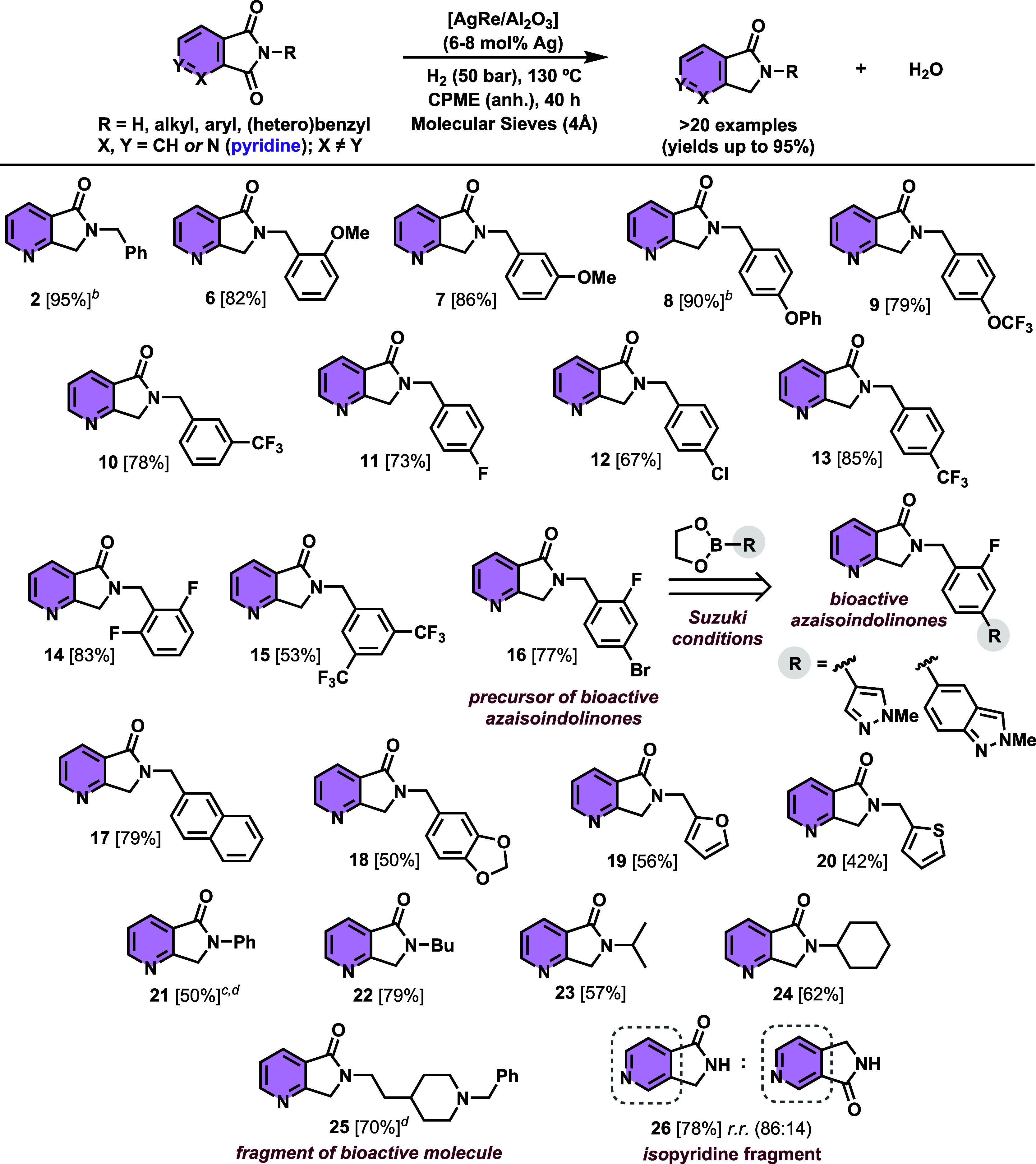
[AgRe/Al_2_O_3_]-Catalyzed Regioselective Hydrodeoxygenation of Azaphthalimides^a^

a Standard reaction conditions: azaphthalimide (0.125 mmol), [AgRe/Al_2_O_3_] nanomaterial (8 mol % Ag, 4.5 wt % Ag in the material), anh. CPME (1 mL), H_2_ (50 bar), 4 Å molecular sieves (40 mg), at 130 °C, 40 h. Isolated yields are given between brackets. r.r. = regioisomeric ratio.
b Run with 6 mol % of Ag.
c Run with 0.2 equiv of aniline.
d The yield was calculated by ^1^H NMR using 1,3,5-trimethoxybenzene as external standard.

## Conclusions

In conclusion, we developed a general heterogeneously catalyzed protocol for the regioselective hydrodeoxygenation of azaphthalimides to the corresponding azalactams with full preservation of the pyridine ring fragment. Using the [AgRe/Al_2_O_3_] nanomaterial, more than 20 azaimides could be efficiently synthesized with full regioselectivity and moderate to good yields. The Ag–Re material, composed of alumina-supported AgReO_4_ nanoaggregates as precatalyst, under the reaction conditions undergoes a dispersive hydrogenolysis to afford the real active catalyst constituted by Ag^0^ nanoparticles in combination with ReO_*x*_ clusters stabilized across the support. Here, we have demonstrated that, for an efficient hydrodeoxygenation process of azaphthalimides, the Ag–Re synergy is mandatory and this cooperativity depends on the existence of an intimate contact between both metals, which is optimal when Ag and Re are present in an equimolar ratio. Moreover, it has also been demonstrated that both metals are directly involved in driving the regioselectivity toward the reduction of C=O more proximal to the nitrogen atom of the pyridine ring through a directing group hydrogenative process. Remarkably, our catalyst showed a notable robustness and stability since it could be effectively used in up to five consecutive reaction cycles.

## Supplementary Material



## Notes

Additional experimental procedures, additional catalytic and kinetic experiments, materials characterization, characterization data, and NMR spectra of all the isolated organic compounds (PDF)

## †

Department of Organic Chemistry, University of Geneva, Geneva, 1211 Geneva 4, Switzerland

## ⊥

Department of Chemical Sciences, University of Padova, Padova 35131, Italy.

## §

Department of Chemistry, Stockholm University, Stockholm 114 18, Sweden.
