# Supplementary material for: Synthesis of Azaisoindolinones by Heterogeneous Catalyzed Regioselective Hydrodeoxygenation of N‑Heteroaromatic Phthalimides
Source: ACS Sustain Chem Eng. 2025 Jul 30;13(31):12328–36. doi: 10.1021/acssuschemeng.5c05996 (PMC12345408; doi:10.1021/acssuschemeng.5c05996)
Supplement: Supplementary file 1 [file sc5c05996_si_001.pdf]

SUPPORTING INFORMATION

Synthesis of Azaisoindolinones by Heterogeneous Catalyzed Regioselective Hydrodeoxygenation of *N*-Heteroaromatic Phthalimides

Carles Lluna-Galán,[a+] Luis Izquierdo-Aranda,[a+] Patricia de la Iglesia-Gómez,[a] Camille Béchet,[a] Pau Vidal-Puyuelo,[a] Juan Camilo Arango-Daza,[a] Rosa Adam[b,*] and Jose R. Cabrero-Antonino[a,*]

[a]Instituto de Tecnología Química (UPV-CSIC),
Universitat Politècnica de València-Consejo Superior de Investigaciones Científicas,
Avd. de los Naranjos s/n, València, 46022 (Spain).
[b]Departament de Química Orgànica,
Facultat de Farmàcia, Universitat de València,
Avd. Vicent Andrés Estellés s/n, Burjassot, València, 46100 (Spain).
[+]These authors contributed equally to this work

*E-mail: rosa.adam@uv.es; jcabrero@itq.upv.es

Content of the Supporting Information: 88 pages, 2 Schemes, 5 Figures and 2 Tables

1. GENERAL INFORMATION

2. GENERAL EXPERIMENTAL PROCEDURES

2.1. General procedure for the synthesis of [$AgRe_x/Al_2O_3$] bimetallic nanomaterials

2.2. General procedure for the synthesis of [Ag/Al_2O_3] and [Re/Al_2O_3] monometallic nanomaterials

2.3. General procedure for the synthesis of azaphthalimides (1, S1-S19)

Scheme S1

2.4. Procedure for the synthesis of azaphthalimide (S20)

Scheme S2

2.5. General procedure for the catalytic studies of the hydrogenation of 6-Benzyl-5*H*-pyrrolo[3,4-*b*]pyridine-5,7(6*H*)-dione (1) by using [$AgRe/Al_2O_3$] nanomaterial

2.6. Synthesis of azalactams from the regioselective hydrodeoxygenation of azaphthalimides by using [$AgRe/Al_2O_3$] nanomaterial

2.7. Procedure for the kinetic studies of 6-Benzyl-5*H*-pyrrolo[3,4-b]pyridine-5,7(6*H*)-dione (1) by using [$AgRe/Al_2O_3$] ex situ reduced nanomaterial

2.8. Procedure for the filtration test of [$AgRe/Al_2O_3$] nanomaterial

2.9. Procedure for the recycling studies of [$AgRe/Al_2O_3$] nanomaterial

3. COMPLEMENTARY CATALYTIC AND KINETIC STUDIES

Table S1

Table S2

Figure S1

4. [$AgRe/Al_2O_3$] SYSTEM HETEROGENEITY EXPERIMENTS

4.1. Catalyst filtration test

Figure S2

4.2. Additional [$AgRe/Al_2O_3$] heterogeneity experiments

4.3. Recycling studies of [$AgRe/Al_2O_3$] nanomaterial

Figure S3

5. CHARACTERIZATION OF THE MATERIALS

5.1. X-Ray Powder Diffraction (XRPD)

Figure S4

5.2. Diffuse Reflectance UV-Vis Spectroscopy (DR UV-Vis)

Figure S5

6. CHARACTERIZATION DATA OF THE ISOLATED ORGANIC COMPOUNDS

7. REFERENCES

8. NMR SPECTRA OF THE ISOLATED ORGANIC COMPOUNDS

1. GENERAL INFORMATION

All the chemicals were obtained from commercial sources and were used without further purification otherwise indicated. 4Å Molecular sieves (MS) were activated at 300 °C under vacuum for 3 h before use. Metallic precursors [Ag(acac)] and [NH_4ReO_4] were purchased from Sigma-Aldrich and γ-Al_2O_3 support was purchased from ABCR (ref. AB255279). 4Å Molecular sieves (MS), and all the screened anhydrous solvents (CPME, *n*-heptane, 2-MeTHF, 1,4-dioxane, mesitylene, toluene and MeOH) were purchased from Sigma-Aldrich. Unless otherwise indicated, for the catalytic evaluation and characterization of all the materials they were used in its freshly prepared form. All the isolated organic compounds were characterized by GC-MS, ^{1}H and ^{13}C NMR, DEPT, bidimensional HSQC, HMBC and NOESY (when considered necessary), ^{19}F RMN (when required) and HRMS (if the compound has not been previously described in the literature). When available, the characterization given in the literature was used for comparison. Gas chromatographic analysis of the catalytic experiments was performed in a Bruker 430-GC equipped with a 25 m capillary column of 5% phenylmethylsilicone. GC-MS analyses were acquired on Agilent 6890 Network gas chromatograph equipped with an HP-5 column (30 m, 0.32 mm, 0.25 µm) coupled to an Agilent 5973 Network mass selective detector. ^{1}H NMR, ^{13}C NMR, ^{19}F, and bidimensional HSQC, HMBC and NOESY NMR spectra were recorded on a Bruker 300 or 400 spectrometer. All chemical shifts (δ) are reported in parts per million (ppm) and coupling constants (*J*) in hertz (Hz). Abbreviations used in the reported NMR experiments are: b, broad; s, singlet; d, doublet; t, triplet; q, quartet; m, multiplet. All chemical shifts are reported relative to residual proton solvents i.e., to CD_3CN (deuterated acetonitrile) peaks δ 1.94 for ^{1}H NMR and δ 1.32 and 118.26 ^{13}C NMR or CD_3OD (deuterated methanol) peaks δ 3.31 for ^{1}H NMR and δ 49.00 ^{13}C NMR. All measurements were carried out at room temperature. All the products were isolated by silica and alumina gel column chromatography using as eluent *n*-hexane/EtOAc mixtures. HRMS measurements of all isolated products were performed using the electrospray ionization (ESI) and atmospheric pressure chemical ionization (APCI) technique in UHPLC TRIPLETOFT6600+/Exion LC AD Pump (SCIEX) equipment. The real amount of metal (wt%) contained in the fresh and used materials was determined by Inductively Coupled Plasma-Atomic Emission Spectroscopy (ICP-AES) using Varian 715-ES after the dissolution of the solid samples in HCl/HNO_3 solution (3:1 vol). X-Ray Powder Diffraction (XRPD) measurements were performed in Bragg-Brentano geometry using a PANalytical CUBIX diffractometer equipped with an X-Celerator detector using Cu Kα (λ1 = 1.5406 Å, λ2 = 1.5444 Å, I2/I1 = 0.5) radiation. The applied tube voltage and intensity were 45 kV and 40 mA, respectively. The length of the goniometer arm is 200 mm, and a fixed divergence slit with a 1/8º aperture was applied. The scanning range was from 3.5° to 90.0° (2θ), with a step of 0.020° (2θ) and an acquisition time of 35 seconds per step. The measurement was performed at 25 °C while the sample was rotated at 0.5 revolutions per second. UV-Vis Diffuse Reflectance spectra were obtained on a Cary 5000 (Agilent Technology) instrument equipped with a Harrick "Praying Mantis" cell, using $BaSO_4$ as a reference for reflectance.

2. GENERAL EXPERIMENTAL PROCEDURES

2.1. General procedure for the synthesis of [$AgRe_x/Al_2O_3$] bimetallic nanomaterials.[1]

A 100 mL round-bottomed flask containing a stirring bar was sequentially charged with [Ag(acac)] (0.4 mmol) and [NH_4ReO_4] (0.2, 0.6 or 3.2 mmol, depending of the $AgRe_x$ nanomaterial, where x = 0.35, 1 or 2) and dissolved in 50 mL of acetone. After 10 min of stirring, γ-Al_2O_3 support (1 g) was added, and the mixture was stirred during 4 h. Once the impregnation was completed, the solvent was evaporated under vacuum and dried in an oven at 100 ºC overnight. Finally, the solid was morturated in a mortar and calcined under air flow at 500 ºC during 3 h (with a heating ramp of 2 ºC/min up to reach 500 ºC). When required, the reduction process of the material was as it follows: a quartz tube containing the fresh calcined material was introduced into a tubular furnace. At this point, a temperature controller and a system to allow gas flow to enter and exit were connected. Once the system was set up, the furnace was programmed to heat (with a heating ramp of 2 ºC/min) up to the noted final temperature (150 or 280 ºC). All the heating processes were done under inert gas flow (N_2 or Ar, 100 mL/min). When the maximum temperature was reached, the gas was changed to H_2 (100 mL/min) and was maintained during 2 h. Then, the furnace was switched off and the catalyst was cooled to room temperature under inert gas flow (N_2 or Ar). Finally, the real metal content at the material was determined by ICP-AES: 4.5 wt% Ag and 2.7 wt% Re for [$AgRe_{0.35}/Al_2O_3$], 4.2 wt% Ag and 7.5 wt% Re for [$AgRe/Al_2O_3$] and 4.1 wt% Ag and 14.0 wt% Re for [$AgRe_2/Al_2O_3$] material. In the case of ex situ hydrogenated materials, both wt% of silver and rhenium metals (measured by ICP-AES) is the same to the ones measured at the just calcined materials.

2.2. General procedure for the synthesis of [Ag/Al_2O_3] and [Re/Al_2O_3] monometallic nanomaterials.[1]

The preparation of [Ag/Al_2O_3] and [Re/Al_2O_3] materials was performed employing the same procedure described above for the bimetallic ones but employing just [Ag(acac)] (0.4 mmol) or [NH_4ReO_4] (1.2 mmol), respectively. Notably, due to the tendency of Re oxidized species to sublimate at high temperatures under an air atmosphere (factor which is more notable in the absence of silver as the other metal of the material) the theoretical amount of rhenium precursor used for synthetizing [Re/Al_2O_3] had to be increased up to double in this case. Finally, the real metal content at the material was determined by ICP-AES: 4.2 wt% Ag for [Ag/Al_2O_3] and 7.5wt% Re for [Re/Al_2O_3] material.

2.3. General procedure for the synthesis of azaphthalimides (1, S1-S19).

The synthesis of azaphthalimide starting materials **1**, **S1-19** was performed following a previous procedure reported in the literature but with slight modifications (Scheme S1):[2] In a 50 mL round-bottomed flask, furo[3,4-*b*]pyridine-5,7-dione (596.5 mg, 4 mmol) was dissolved in glacial acetic acid (8 mL, ACS reagent, ≥99.7%). Then, the corresponding amine (4 mmol) was added dropwise and the reaction mixture was kept under stirring at 130 ºC during 24 h. Once completed this time, the reaction mixture was cooled down to room temperature and treated with Na_2CO_3 or NaOH (2 M) aqueous solution where a precipitated was formed, which was filtered under vacuum and washed with water affording the corresponding desired imide **1** or **S1** to **S19** as a solid. In some cases, in which the precipitation step was not efficient enough a liquid-liquid extraction employing EtOAc from aqueous neutral phase was performed. After the extraction process, the organic layers were dried with anhydrous sodium sulfate and evaporated under vacuum, and the obtained residue was purified by silica gel column chromatography using *n*-hexane/EtOAc mixtures as eluent to afford the desired azaimide. Isolated yields are shown below between brackets (see Scheme S1).

Scheme S1. Synthesis of azaphthalimides **1**, **S1-S19** from the corresponding amines and azaphthalic anhydride.

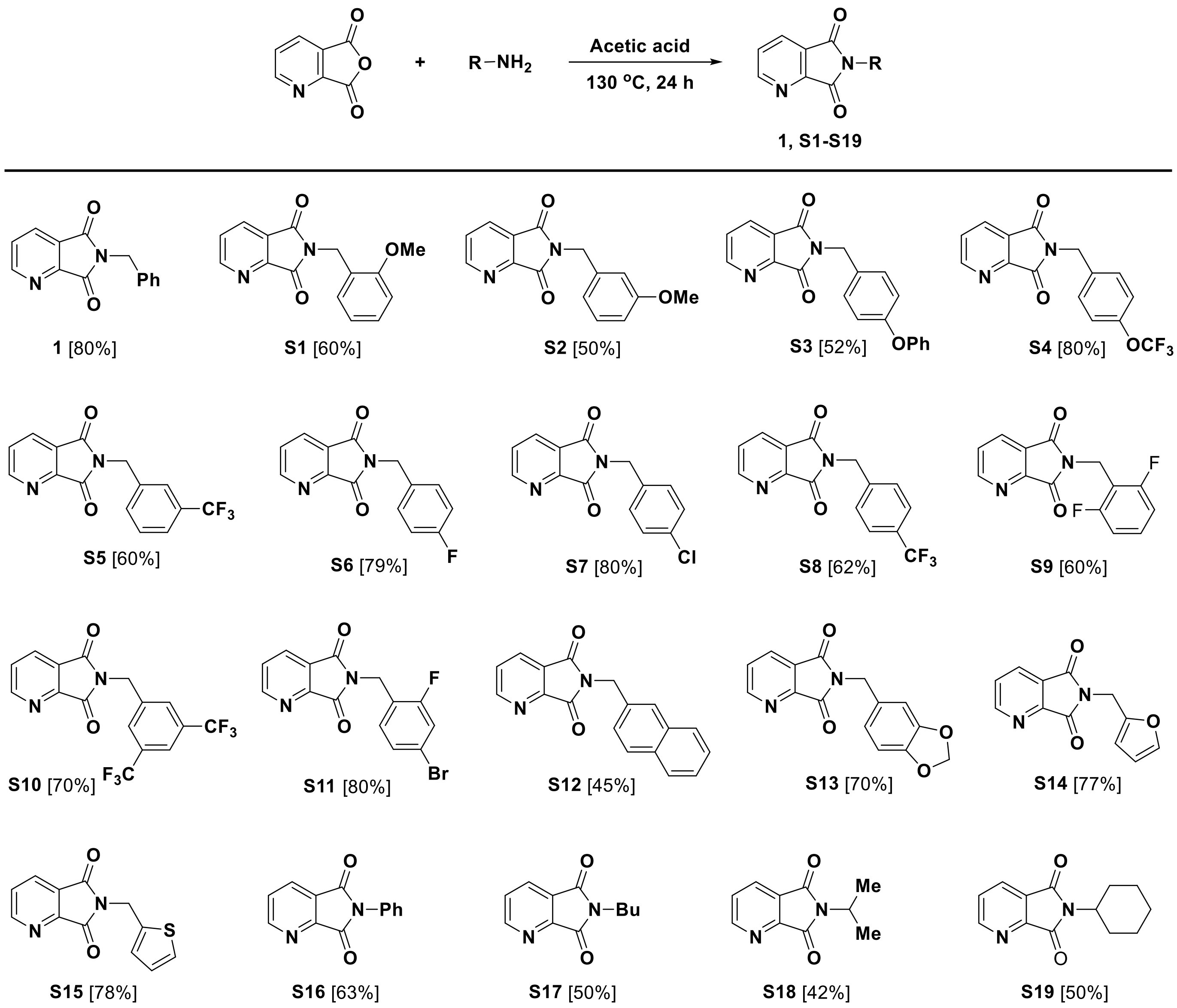

2.4. Procedure for the synthesis of azaphthalimide (S20)

The synthesis of azaimide **S20** was performed following a previous reported procedure in the literature but with slight modifications (Scheme S2):[3] A mixture of 2-(1-benzylpiperidin-4-yl)ethan-1-amine (1.03 g, 4.7 mmol) and furo[3,4-*b*]pyridine-5,7-dione (775.4 mg, 5.2 mmol, 1.1 eq.) in dioxane (30 mL) was refluxed for 6 h. Then, 50 mL of distilled water was added and the reaction mixture was extracted three times with $CHCl_3$. The organic layers were dried with anhydrous sodium sulfate and evaporated under vacuum, and the obtained residue was purified by silica gel column chromatography using *n*-hexane/EtOAc mixtures as eluent affording azalactam **S20** in 85% isolated yield (Scheme S2).

Scheme S2. Synthesis of azaphthalimide **20** from the corresponding amine and azaphthalic anhydride.

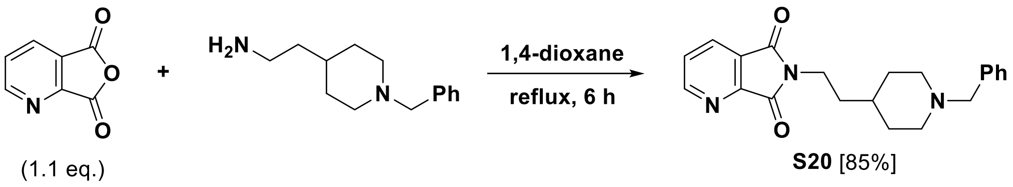

2.5. General procedure for the catalytic studies of the hydrogenation of 6-Benzyl-5*H*-pyrrolo[3,4-*b*]pyridine-5,7(6*H*)-dione (1) by using [AgRe/Al_2O_3] nanomaterial.

A 8 mL vial equipped with a stirring bar, was sequentially charged with 6-Benzyl-5*H*-pyrrolo[3,4-b]pyridine-5,7(6*H*)-dione **1** (29.8 mg, 0.125 mmol), [AgRe/Al_2O_3] (19.3 mg, 6 mol% Ag, 4.2 wt% of Ag at the material), molecular sieves 4Å (40 mg, preactivated at 300 ºC under vacuum during 3 h), *n*-dodecane (20 µL) as an internal standard and anhydrous CPME (1 mL) as solvent. Afterwards, the reaction vial was closed with a screw cap with a septum and introduced into a 300 mL vessel. Then, the septum was perforated with a needle and the vessel was closed and pressurized with H_2 (50 bar) after three purges. Finally, the vessel was placed into an aluminum block preheated at 100, 130 or 150 ºC for 20 or 40 h. Once the desired time was completed, the autoclave was cooled down using an ice bath, carefully depressurized and opened. Finally, the reaction mixture was diluted with EtOAc, centrifuged and the supernatant was analyzed by GC. In some selected cases, the reaction was also analyzed by ^{1}H NMR using 1,3,5-trimethoxybenzene as external standard and DMSO-d_6 as deuterated solvent.

2.6. Synthesis of azalactams from the regioselective hydrodeoxygenation of azaphthalimides by using [AgRe/Al_2O_3] nanomaterial.

A 8 mL glass vial containing a stirring bar was sequentially charged with the corresponding azaphthalimide (0.125 mmol), [AgRe/Al_2O_3] material (6-8 mol% Ag), molecular sieves 4Å (40 mg, pretreated at 300 °C under vacuum during 3 h) and anhydrous CPME (1 mL) as solvent. Then, the reaction vial was closed with a screw cap with a septum and introduced into a 300 mL vessel. Next, the septum was perforated with a needle and the vessel was closed and pressurized with H_2 (50 bar) after three purges. Finally, the vessel was placed into an aluminum block preheated at 130 ºC during 40 h. Once the time was completed, the autoclave was cooled down with the help of an ice bath, carefully depressurized and opened. The reaction mixture was diluted with EtOAc, centrifuged and the supernatant was analyzed by GC and GC-MS. Once the formation of the azalactam was identified, its purification was carried out by using column chromatography on silica or preparative TLC employing *n*-hexane/EtOAc mixtures as eluent. Note that in the case of azalactam compounds **21** and **25**, purification via column chromatography was not performed and ^{1}H NMR yield of the product was calculated from the crude of the hydrogenation reaction using 1,3,5-trimethoxybenzene as external standard.

2.7. Procedure for the kinetic studies of 6-Benzyl-5*H*-pyrrolo[3,4-*b*]pyridine-5,7(6*H*)-dione (1) by using [AgRe/Al_2O_3] ex situ reduced nanomaterial.

Fixed-point data for the kinetic study was performed by a slightly modified standard reaction procedure. A 8 mL vial containing a stirring bar was sequentially charged with 6-Benzyl-5*H*-pyrrolo[3,4-*b*]pyridine-5,7(6*H*)-dione **1** (29.8 mg, 0.125 mmol), [AgRe/Al_2O_3] (19.3 mg, 6 mol% Ag) ex situ reduced material at 150 ºC during 2 h using a heating ramp of 2 ºC/min, molecular sieves 4Å (40 mg, pretreated at 300 ºC under vacuum during 3 h) and anhydrous CPME (1 mL) as solvent. Afterwards, the reaction vial was closed with a screw cap with a septum and introduced into a 300 mL vessel. Then, the septum was perforated with a needle and the vessel was closed and pressurized with H_2 (50 bar) after three purges. Finally, the vessel was placed into an aluminum block preheated at 130 ºC for the required time, 0.5, 1, 2, 4, 6 and 24 h (each reaction at different time was performed in a separate autoclave). Once the desired reaction time was completed, the autoclave was cooled down using an ice bath, carefully depressurized and opened. Finally, an aliquot of the reaction mixture was evaporated and analyzed by ^{1}H NMR using 1,3,5-trimethoxybenzene as external standard and DMSO-d_6 as deuterated solvent (see Figure S1).

2.8. Procedure for the filtration test of [AgRe/Al_2O_3] nanomaterial.

A 8 mL glass vial containing a stirring bar was sequentially charged with 6-benzyl-5*H*-pyrrolo[3,4-*b*]pyridine-5,7(6*H*)-dione **1** (29.8 mg, 0.125 mmol), [AgRe/Al_2O_3] material (19.3 mg, 6 mol% Ag), molecular sieves 4Å (40 mg, pretreated

at 300 ºC under vacuum during 3 h), and anhydrous CPME (1 mL) as solvent. Then, the reaction vial was closed with a screw cap with a septum and introduced into a 300 mL vessel. Then, the septum was perforated with a needle and the vessel was closed and pressurized with H_2 (50 bar) after three purges. Finally, the vessel was placed into an aluminum block preheated at 130 ºC and the reaction was maintained for 3 h. After this time, the autoclave was cooled down in an ice bath, carefully depressurized and opened. The reaction mixture was then centrifuged and the supernatant was evaporated and analyzed by ^1H NMR using 1,3,5-trimethoxybenzene as external standard and DMSO-d_6 as deuterated solvent. Then, the liquid mixture without catalyst was poured into a new glass vial equipped with a stirring bar, which was closed with a screw cap with a septum and introduced into a new 300 mL vessel. Then, the septum was perforated with a needle and the vessel was closed and pressurized with H_2 (50 bar) after three purges. The vessel was placed into an aluminum block preheated at 130 ºC and maintained for the required additional time of 1, 3 and 21 h (each reaction at different time was performed in a separate autoclave). Once the desired reaction time was completed, the autoclave was cooled down using an ice bath, carefully depressurized and opened. Finally, the reaction mixture was evaporated and analyzed by ^1H NMR using 1,3,5-trimethoxybenzene as external standard and DMSO-d_6 as deuterated solvent (see Figure S2).

2.9. Procedure for the recycling studies of [AgRe/Al_2O_3] nanomaterial.

Recycling experiments for [AgRe/Al_2O_3] system in the hydrogenation of azaphthalimide **1** were carried out by 6-fold scaling up the above-mentioned general reaction procedure. A 25 mL vial containing a stirring bar was sequentially charged with 6-Benzyl-5*H*-pyrrolo[3,4-*b*]pyridine-5,7(6*H*)-dione **1** (168.8 mg, 0.75 mmol), [AgRe/Al_2O_3] material (6 mol% Ag), molecular sieves 4Å (150 mg, preactivated at 300 ºC under vacuum during 3 h) and anhydrous CPME (5 mL) as solvent. Afterwards, the reaction vial was closed with a screw cap performed with a needle and introduced into the 300 mL autoclave. Then, the autoclave was closed and pressurized with H_2. After three purges, the autoclave was pressurized with 50 bar of H_2 and placed into an aluminium block preheated at 130 ºC. After 20 h, the autoclave was cooled down in an ice bath, carefully depressurized and opened. Then, an aliquot of the reaction mixture was diluted with EtOAc, centrifuged and the supernatant was evaporated and analysed by ^1H NMR using 1,3,5-trimethoxybenzene as external standard and DMSO-d_6 as deuterated solvent. The recovered catalyst after use in reaction was filtered under vacuum, washed with AcOEt, *n*-hexane and acetone and dried in an oven at 100 ºC overnight. Finally, prior its use in a subsequent cycle the used material was calcined under air flow at 300 ºC during 3 h with a heating ramp of 2 ºC/min. After each reaction cycle, the metal content of the catalytic material was determined by ICP-AES (see Figure S3).

3. COMPLEMENTARY CATALYTIC AND KINETIC STUDIES

Table S1. Study of the solvent effect in the $[AgRe/Al_2O_3]$-catalyzed hydrogenation of *N*-benzyl azaphthalimide **1**.

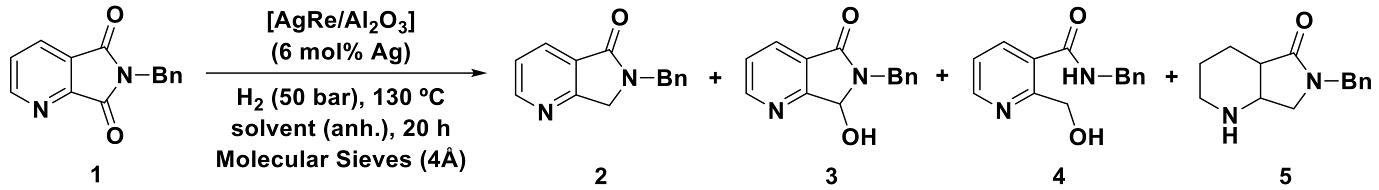

Entry[a]	Solvent (anh.)	Conv. **1** (%)[b]	**2** (%)[b]	**3** (%)[b]	**4** (%)[b]	**5** (%)[b]
1	CPME	91	65	20	4	n.d.
2	*n*-Heptane	55	40	n.d.	4	3
3	2-Me-THF	10	5	n.d.	2	n.d.
4	1,4-Dioxane	77	57	12	2	2
5	Mesitylene	60	18	5	15	6
6	Toluene	64	45	n.d.	9	5
7	MeOH	20	12	4	n.d.	n.d.

[a]Standard reaction conditions: *N*-benzyl azaphthalimide **1** (0.125 mmol), $[AgRe/Al_2O_3]$ (6 mol% Ag), H_2 (50 bar), anhydrous solvent (1 mL), *n*-dodecane (20 μL), molecular sieves 4Å (40 mg, previously dried under vacuum at 300 ºC during 3 h) at 130 °C during 20 h. [b]Conversion of **1** and yield of products **2**, **3**, **4** and **5** were calculated by GC using *n*-dodecane as internal standard. N.d. = no detected. Bn = Benzyl.

Table S2. Catalytic evaluation (cooperativity and regioselectivity studies) of bimetallic [$AgRe_x/Al_2O_3$] and monometallic [Ag/Al_2O_3] and [Re/Al_2O_3] materials in the hydrogenation of *N*-benzyl azaphthalimide **1**.

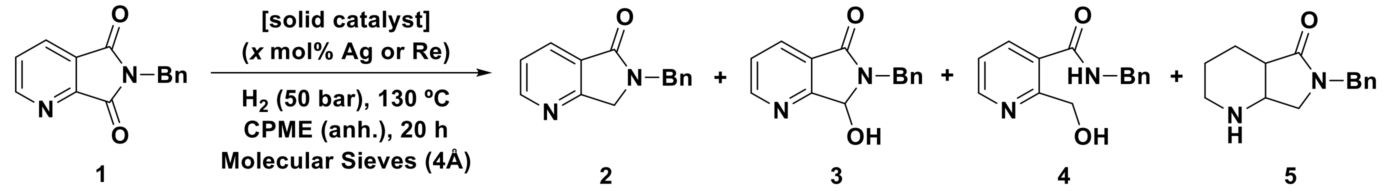

Entry[a]	[solid catalyst] (mol% Ag or Re)	Conv. **1** (%)[b]	**2** (%)[b]	**3** (%)[b]	**4** (%)[b]	**5** (%)[b]
1	[$AgRe/Al_2O_3$] (6, Ag)	91	65	20	4	n.d.
2	[Ag/Al_2O_3] (6)	56	n.d.	53	2	n.d.
3	[Ag/Al_2O_3] (12)	64	n.d.	29	26	n.d.
4	[Re/Al_2O_3] (6)	<5	2	n.d.	1	n.d.
5	[Re/Al_2O_3] (12)	<5	2	n.d.	1	n.d.
6[c]	[Re/Al_2O_3]-H_2-280 (6)	<5	2	n.d.	1	n.d.
7[c]	[Re/Al_2O_3]-H_2-280 (12)	10	8	n.d.	2	n.d.
8	[Ag/Al_2O_3] (6) + [Re/Al_2O_3] (6)	41	30	4	2	n.d.
9	[$AgRe_{0.35}/Al_2O_3$] (6, Ag)	50	21	20	7	n.d.
10	[$AgRe_2/Al_2O_3$] (6, Ag)	37	16	n.d.	12	2

[a]Standard reaction conditions: *N*-benzyl azaphthalimide **1** (0.125 mmol), [solid catalyst] (*x* mol% Ag or Re), H_2 (50 bar), anh. CPME (1 mL), *n*-dodecane (20 µL), molecular sieves 4Å (40 mg, previously dried under vacuum at 300 ºC during 3 h) at 130 °C during 18 h. [b]Conversion of **1** and yield of products **2**, **3**, **4** and **5** were calculated by GC using *n*-dodecane as internal standard. Complementarily, ^1H NMR analysis by using 1,3,5-trimethoxybenzene as external standard and DMSO-d_6 as deuterated solvent was also employed in order to confirm the exact regioisomer obtained in each case, the conversion of azaimide **1** and the yields of the hydrogenated products **2** to **5**. In all cases, full regioselectivity towards hydrogenation of the C=O bond spatially closer to nitrogen pyridine atom at the azaimide **1** was observed. [c]In this case, [Re/Al_2O_3] material was reduced ex situ at 280 ºC during 3 h with a heating ramp of 2 ºC/min prior its use in the catalytic hydrogenation test. N.d. = no detected. Bn = Benzyl.

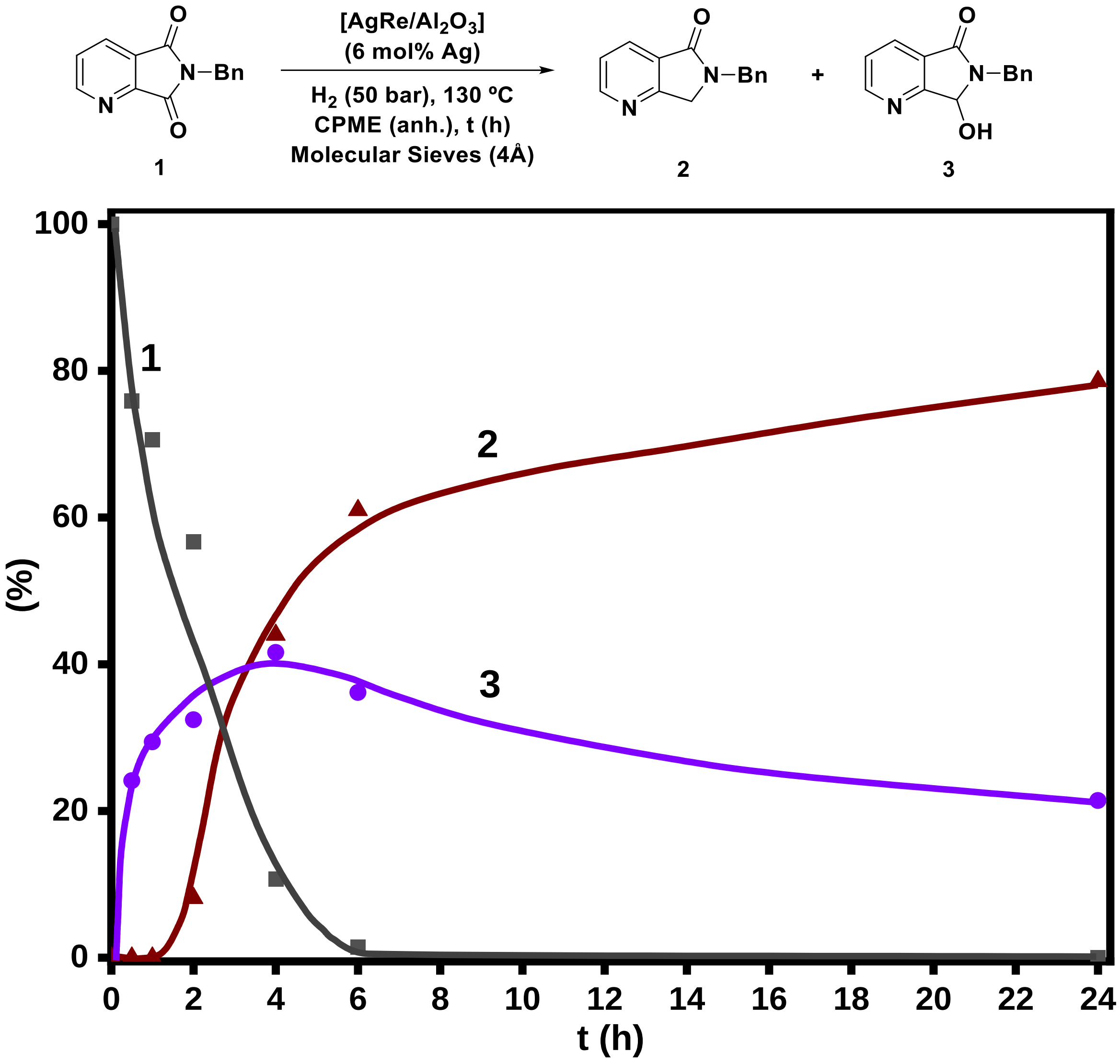

Figure S1. Yield/time kinetic profile for the hydrogenation of *N*-benzyl azaphthalimide **1** in the presence of [AgRe/Al_2O_3] nanocatalyst. Reaction conditions: *N*-benzyl azaphthalimide **1** (0.125 mmol), [AgRe/Al_2O_3] (6 mol% Ag), 4Å molecular sieves (MS) (pretreated at 300 °C under vacuum during 3 h, 40-50 mg), H_2 (50 bar), anh. CPME (1 mL) at 130 °C during 0.5, 1, 2, 4, 6 and 24 h (due to experimental reasons, each catalytic test carried out at the indicated time was performed as a separated experiment). In all cases, prior its use in the catalytic test, [AgRe/Al_2O_3] material was reduced ex situ under H_2 flow at 150 °C during 2 h with a heating ramp of 2 °C/min to avoid the kinetic induction period.[1] The disappearance of azaimide **1** and yields of products **2** and **3** were determined by ^{1}H NMR using 1,3,5-trimethoxybenzene as an external standard and DMSO-d$_6$ as deuterated solvent.

4. [AgRe/Al_2O_3] SYSTEM HETEROGENEITY EXPERIMENTS

4.1. Catalyst filtration test

These experiments were performed following the procedure described in section 2.8 of the SI.

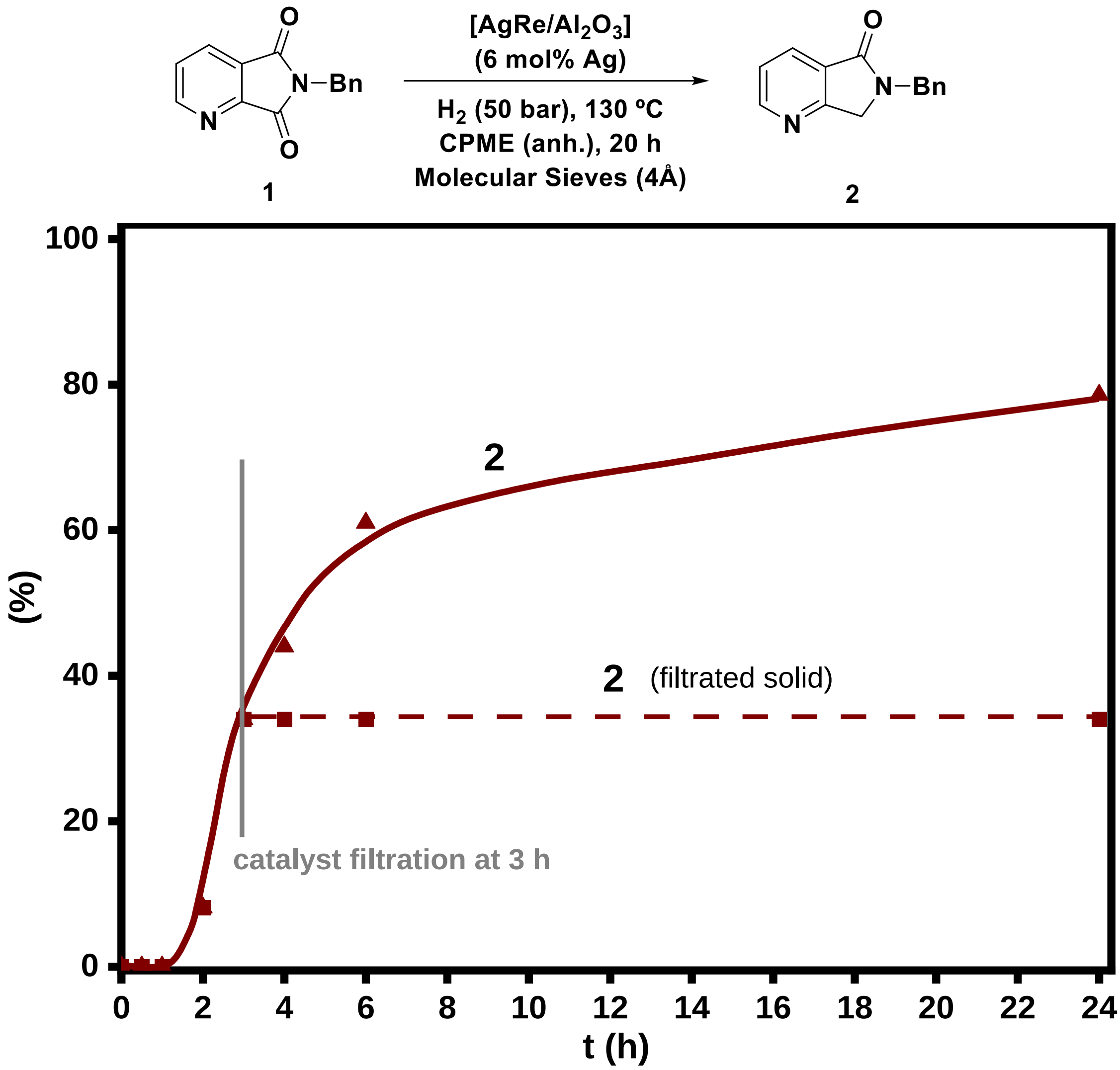

Figure S2. [AgRe/Al_2O_3] filtration test for the hydrogenation of *N*-benzyl azaphthalimide **1**. Yield/time kinetic profile for the formation of product **2** for standard reaction (—) and for the reaction filtrated at 3 h (– – –). Reaction conditions: *N*-benzyl azaphthalimide **1** (0.125 mmol), [AgRe/Al_2O_3] (6 mol% Ag), 4Å molecular sieves (MS) (pretreated at 300 °C under vacuum during 3 h, 40-50 mg), H_2 (50 bar), anh. CPME (1 mL) at 130 °C during 0.5, 1, 2, 4, 6 and 24 h (due to experimental reasons, each catalytic test carried out at the indicated time was performed as a separated experiment). In all cases, prior its use in the catalytic test, [AgRe/Al_2O_3] material was reduced ex situ under H_2 flow at 150 °C during 2 h with a heating ramp of 2 °C/min to avoid the kinetic induction period.[1] Yield of product **2** was determined by ^1H NMR using 1,3,5-trimethoxybenzene as external standard and DMSO-d_6 as deuterated solvent.

4.2. Additional [AgRe/Al_2O_3] heterogeneity experiments

Moreover, the possible leaching of metal species to the reaction mixture was studied analyzing the metal content of both the final catalyst material and the reaction mixture. With this purpose, at the end of the filtration test performed in the previously exposed conditions for the hydrogenation of **1**, the reaction mixture was evaporated to dryness and the residue was dissolved with *aqua regia* ($HCl:HNO_3$, 3:1 in volume) and analyzed by ICP-AES. At the reaction mixture, no presence of Ag and/or Re was detected. For material metal content after being used under optimized reaction conditions during 20 h, the wt% of Ag and Re was just slightly lower than the original one (4.4% for Ag respect to 4.5% and 7.4% for Re respect to 7.5%, determined by ICP-AES), being maintained the molar relationship (Ag:Re = 1:1).

4.3. Recycling studies of [AgRe/Al_2O_3] nanomaterial

These experiments were performed following the procedure described in section 2.9 of the SI.

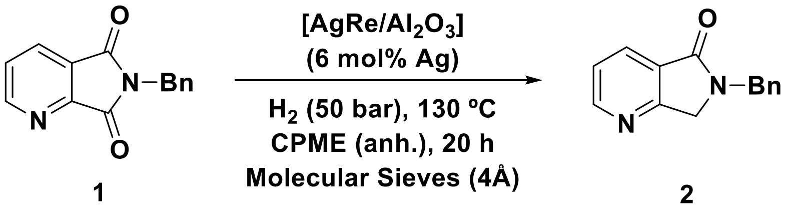

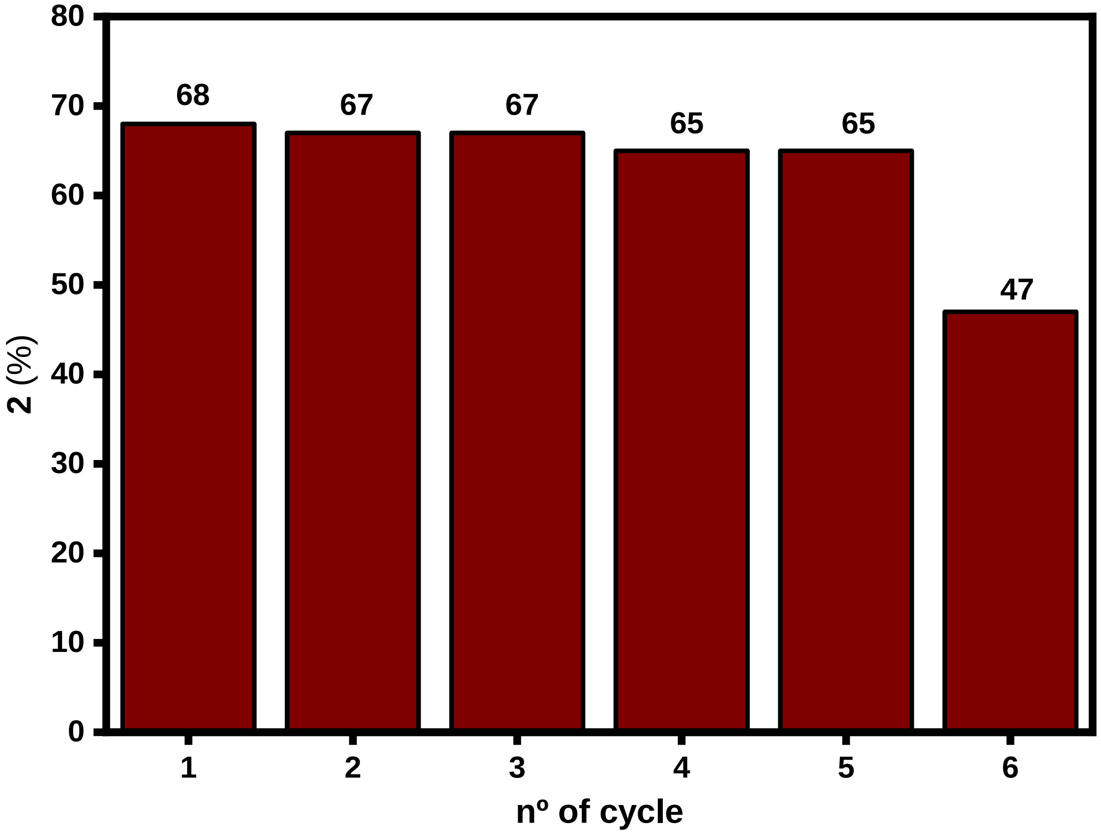

Figure S3. Recycling study of [AgRe/Al_2O_3] nanomaterial in the hydrodeoxygenation of *N*-benzyl azaphthalimide **1** to azaisoindolinone **2**. Reaction conditions: *N*-benzyl azaphthalimide **1** (0.75 mmol), [AgRe/Al_2O_3] (6 mol% Ag), H_2 (50 bar), anh. CPME (2 mL), molecular sieves 4Å (40 mg, previously dried under vacuum at 300 ºC during 3 h) at 130 °C during 20 h. Prior its use in a successive reaction cycle, the recovered [AgRe/Al_2O_3] material was calcined at 300 ºC under air flow during 4 h. Yield of product **2** was determined by ^1H NMR using 1,3,5-trimethoxybenzene as external standard and DMSO-d_6 as deuterated solvent.

5. CHARACTERIZATION OF THE MATERIALS

5.1. X-Ray Powder Diffraction (XRPD)

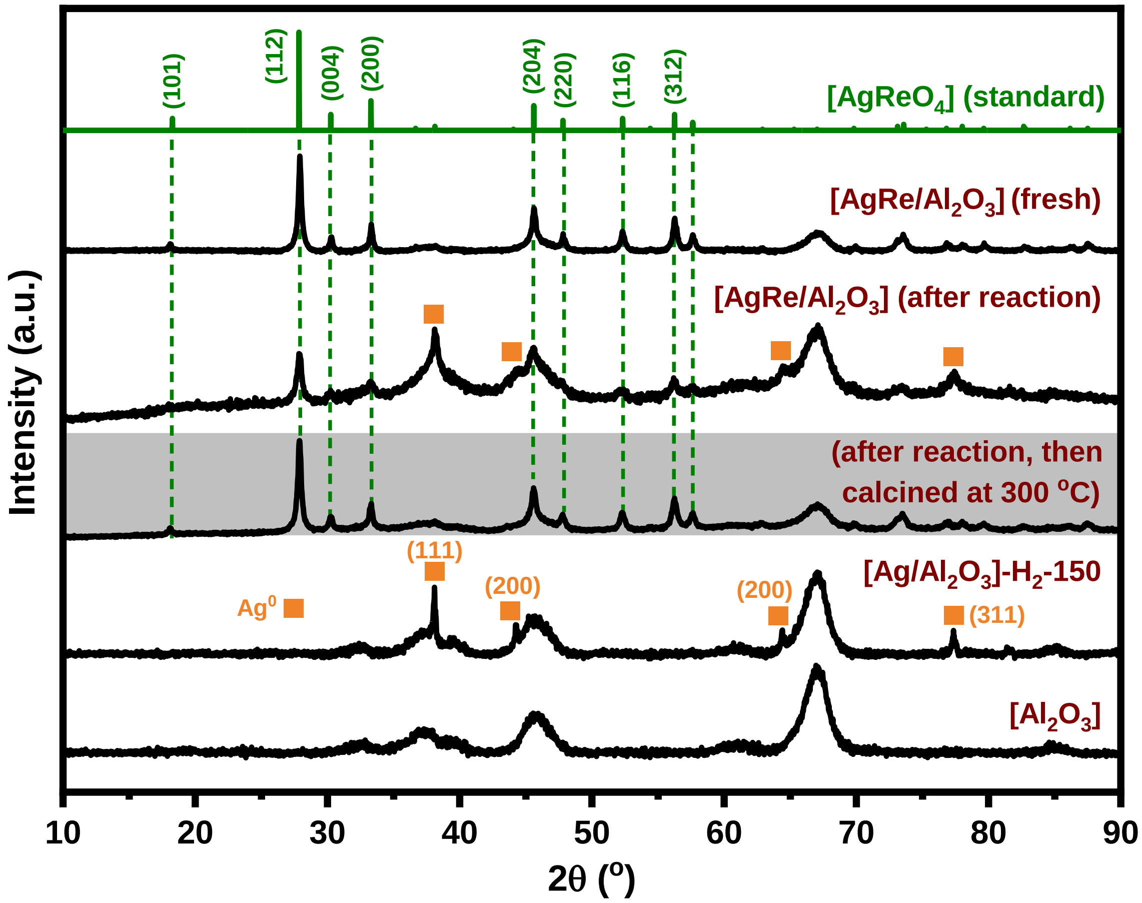

Figure S4. XRPD pattern of freshly prepared [AgRe/Al_2O_3] material, [AgRe/Al_2O_3] material after being used in a reaction cycle, [AgRe/Al_2O_3] material after being used in a reaction cycle and then calcined at 300 ºC during 2 h with a heating ramp of 2 ºC/min, freshly reduced [Ag/Al_2O_3] material at 150 ºC, γ-Al_2O_3 support and $AgReO_4$ standard with scheelite structure used for comparison (ICDDN 00-008-095). Miller indexes of the most representative diffractions of [$AgReO_4$] are shown between parentheses,[4, 5] and diffraction patterns corresponding to Ag(0) species are represented by orange squares.[6, 7]

5.2. Diffuse Reflectance UV-Vis Spectroscopy (DR UV-Vis)

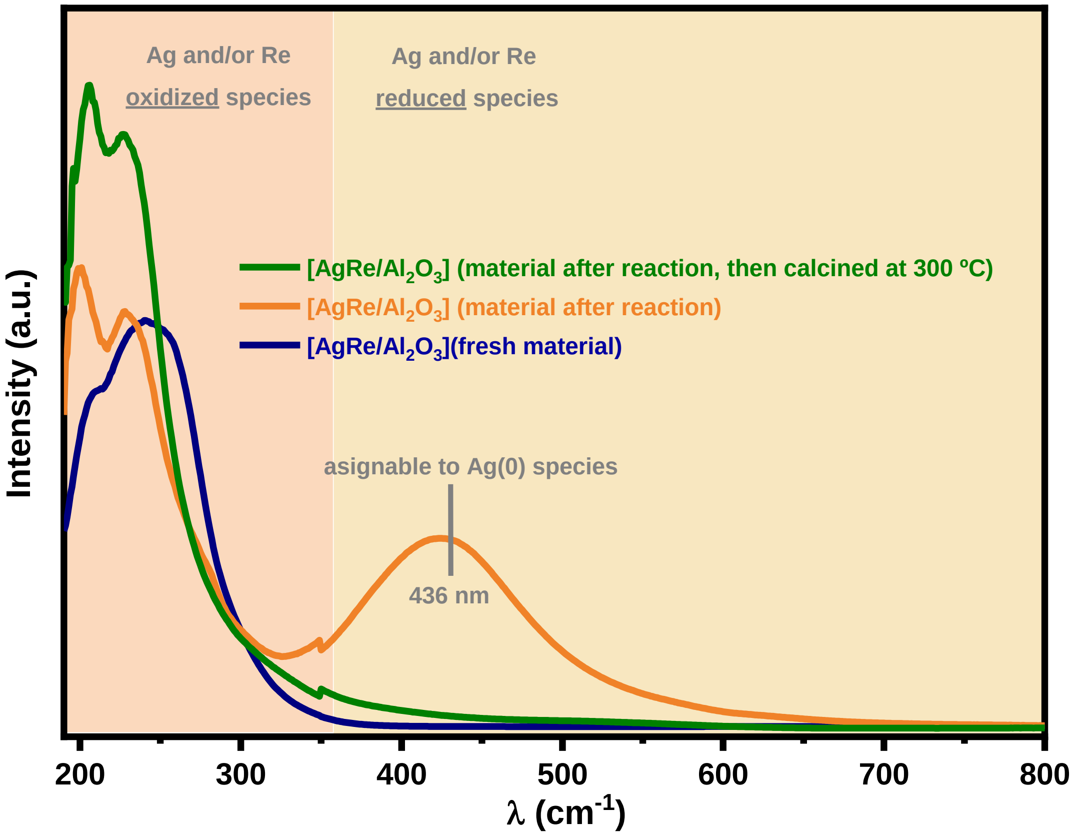

Figure S5. Diffuse reflectance UV-Vis spectra of freshly prepared [AgRe/Al_2O_3] nanomaterial, [AgRe/Al_2O_3] nanomaterial recovered after being used in a reaction cycle and [AgRe/Al_2O_3] nanomaterial recovered after being used in a reaction cycle and then calcined at 300 ºC during 3 h with a heating ramp of 2 ºC/min. It has been well stablished in the literature that Ag^+ ions and $Ag^{\delta+}$ clusters as well as Re^{7+} species absorb in the 200-300 nm region, while Ag^0 and reduced Re species absorb at higher wavelengths between 330-800.[8-14]

6. CHARACTERIZATION DATA OF THE ISOLATED ORGANIC COMPOUNDS

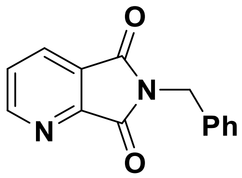

6-Benzyl-5*H*-pyrrolo[3,4-*b*]pyridine-5,7(6*H*)-dione (1).[15] Isolated yield: 80%. GC-MS (m/z, $M^{+\cdot}$ 238), major peaks found: 238 (63%), 207 (22%), 181 (52%), 155 (17%), 79 (100%), 50 (21%). The NMR spectrum is consistent with literature. ^{1}H NMR (400 MHz, $CDCl_3$) *δ*: 8.92 (dd, *J* = 5.0, 1.5 Hz, 1H), 8.12 (dd, *J* = 7.6, 1.5 Hz, 1H), 7.56 (dd, *J* = 7.7, 4.9 Hz, 1H), 7.45 – 7.36 (m, 2H), 7.33 – 7.21 (m, 3H), 4.88 (s, 2H). ^{13}C NMR (100 MHz, $CDCl_3$) *δ*: 166.1, 166.0, 155.4, 152.0, 136.0, 131.4, 129.0, 128.9, 128.2, 127.6, 127.5, 42.0.

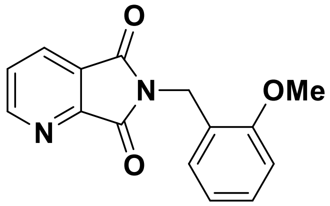

6-(2-Methoxybenzyl)-5*H*-pyrrolo[3,4-*b*]pyridine-5,7(6*H*)-dione. **(S1).** Isolated yield: 60%. GC-MS (m/z, $M^{+\cdot}$ 268), major peaks found: 268.0 (100%), 240 (26%), 225 (38%), 181 (33%), 91 (26%), 79 (40%). ^{1}H NMR (400 MHz, CD_3CN) *δ*: 8.95 (dd, *J* = 5.0, 1.5 Hz, 1H), 8.19 (dd, *J* = 7.7, 1.5 Hz, 1H), 7.70 (dd, *J* = 7.7, 5.0 Hz, 1H), 7.28 (td, *J* = 7.9, 1.8 Hz, 1H), 7.15 (dd, *J* = 7.7, 1.5 Hz, 1H), 6.98 (dd, *J* = 8.3, 1.1 Hz, 1H), 6.88 (td, *J* = 7.5, 1.1 Hz, 1H), 4.84 (s, 2H), 3.83 (s, 3H). ^{13}C NMR (100 MHz, CD_3CN) *δ*: 167.5, 167.3, 158.0, 156.0, 152.9, 132.0, 129.8, 128.9, 128.7, 128.6, 124.9, 121.2, 111.7, 56.2, 37.7. HRMS (ESI+) [M+H$^+$; calculated for $C_{15}H_{13}N_2O_3$: 269.0921] found m/z 269.0920.

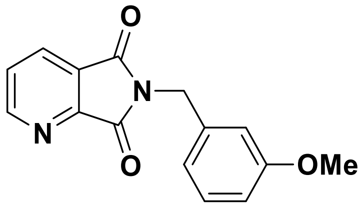

6-(3-Methoxybenzyl)-5*H*-pyrrolo[3,4-*b*]pyridine-5,7(6*H*)-dione (S2). Isolated yield: 50%. GC-MS (m/z, $M^{+\cdot}$ 268), major peaks found: 268 (100%), 240 (40%), 211 (39%), 79 (57%). ^{1}H NMR (400 MHz, CD_3CN) *δ*: 8.93 (dd, *J* = 5.0, 1.5 Hz, 1H), 8.17 (dd, *J* = 7.7, 1.5 Hz, 1H), 7.68 (dd, *J* = 7.7, 5.0 Hz, 1H), 7.24 (t, *J* = 7.9 Hz, 1H), 6.94 – 6.91 (m, 2H), 6.85 – 6.81 (m, 1H), 4.81 (s, 2H), 3.75 (s, 3H). ^{13}C NMR (100 MHz, CD_3CN) *δ*: 167.6, 167.3, 160.9, 156.1, 152.9, 139.1, 132.1, 130.7, 128.7, 128.6, 120.8, 114.4, 114.0, 55.9, 42.2. HRMS (ESI+) [M+H$^+$; calculated for $C_{15}H_{13}N_2O_3$: 269.0921] found m/z 269.0932.

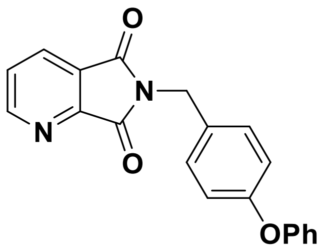

6-(4-Phenoxybenzyl)-5*H*-pyrrolo[3,4-*b*]pyridine-5,7(6*H*)-dione (S3). Isolated yield: 52%. GC-MS (m/z, $M^{+\cdot}$ 330), major peaks found: 330 (100%), 302 (42%), 273 (19%), 209 (23%), 79 (32%). ^{1}H NMR (400 MHz, CD_3CN) *δ*: 8.93 (dd,

J = 5.0, 1.5 Hz, 1H), 8.18 (dd, J = 7.7, 1.5 Hz, 1H), 7.69 (dd, J = 7.7, 5.0 Hz, 1H), 7.39 – 7.34 (m, 4H), 7.15 – 7.11 (m, 1H), 7.00 – 6.93 (m, 4H), 4.83 (s, 2H). ^{13}C NMR (75 MHz, CD_3CN) *δ:* 167.6, 167.4, 158.0, 157.9, 156.1, 153.0, 132.5, 132.1, 131.0, 130.7, 128.7, 124.6, 119.9, 119.6, 41.7. HRMS (ESI+) [M+H$^+$; calculated for $C_{20}H_{15}N_2O_3$: 331.1077] found m/z 331.1082.

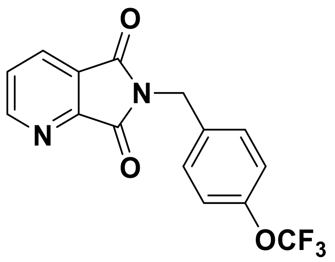

6-(4-(Trifluoromethoxy)benzyl)-5H-pyrrolo[3,4-*b*]pyridine-5,7(6*H*)-dione (S4). Isolated yield: 80%. GC-MS (m/z, $M^{+\cdot}$ 322), major peaks found: 322 (100%), 294 (30%), 265 (48%), 239 (22%), 79 (75%). ^{1}H NMR (400 MHz, CD_3CN) *δ*: 8.93 (dd, J = 5.0, 1.5 Hz, 1H), 8.19 (dd, J = 7.7, 1.5 Hz, 1H), 7.69 (dd, J = 7.7, 5.0 Hz, 1H), 7.48 (d, J = 7.8 Hz, 2H), 7.25 (d, J = 7.8 Hz, 2H), 4.86 (s, 2H). ^{13}C NMR (100 MHz, CD_3CN) *δ:* 167.5, 167.3, 156.2, 152.9, 149.5 – 149.4 (m), 136.8, 132.3, 130.9, 128.8, 128.7, 122.2, 121.1 (q, J^1_{C-F} = 245 Hz), 41.6. ^{19}F NMR (282 MHz, CD_3CN) *δ*: -58.67 (s, 3F). HRMS (ESI+) [M+H$^+$; calculated for $C_{15}H_{10}F_3N_2O_3$: 323.0638] found m/z 323.0632.

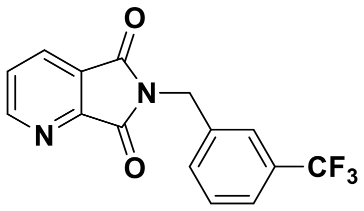

6-(3-(Trifluoromethyl)benzyl)-5*H*-pyrrolo[3,4-*b*]pyridine-5,7(6*H*)-dione (S5). Isolated yield: 60%. GC-MS (m/z, $M^{+\cdot}$ 306), major peaks found: 306 (100%), 307 (24%), 249 (42%), 223 (22%), 79 (83%), 77 (26%). ^{1}H NMR (400 MHz, CD_3CN) *δ*: 8.94 (dd, J = 5.0, 1.5 Hz, 1H), 8.19 (dd, J = 7.7, 1.5 Hz, 1H), 7.71 -7.50 (m, 5H), 4.92 (s, 2H). ^{13}C NMR (75 MHz, CD_3CN) *δ:*167.5, 167.3, 156.2, 152.9, 138.8, 132.9 (d, J^4_{C-F} = 1.0 Hz), 132.2, 131.2 (q, J^2_{C-F} = 32.1 Hz), 130.5, 128.8, 128.7, 125.7 (q, J^3_{C-F} = 3.8 Hz), 125.5 (q, J^3_{C-F} = 3.8 Hz), 125.3 (q, J^1_{C-F} = 271.5 Hz), 41.9. ^{19}F NMR (282 MHz, CD_3CN) *δ*: -63.09 (s, 3F). HRMS (ESI+) [M+H$^+$; calculated for $C_{15}H_{10}F_3N_2O_2$: 307.0655] found m/z 307.0651.

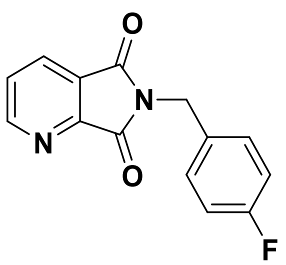

6-(4-Fluorobenzyl)-5*H*-pyrrolo[3,4-*b*]pyridine-5,7(6*H*)-dione. **(S6).**[16] Isolated yield: 79%. GC-MS (m/z, $M^{+\cdot}$ 256), major peaks found: 256 (100%), 228 (29%), 199 (68%), 109 (29%), 107 (25%), 79 (72%). The NMR spectrum is consistent with literature. ^{1}H NMR (400 MHz, CD_3CN) *δ*: 8.93 (dd, J = 5.0, 1.5 Hz, 1H), 8.18 (dd, J = 7.7, 1.5 Hz, 1H), 7.68 (dd, J = 7.7, 5.0 Hz, 1H), 7.41 (dd, J = 8.9, 5.4 Hz, 1H), 7.07 (t, J = 8.9 Hz, 1H), 4.82 (s, 1H). ^{13}C NMR (75 MHz, CD_3CN) *δ* 167.5, 167.3, 163.2 (d, J^1_{C-F} = 243.9 Hz), 156.1, 152.9, 133.7 (d, J^4_{C-F} = 3.2 Hz), 132.1, 131.1 (d, J^3_{C-F} = 8.3 Hz), 128.7, 128.6, 116.3 (d, J^2_{C-F} = 21.9 Hz), 41.6. ^{19}F NMR (282 MHz, CD_3CN) *δ*: -116.48 (tt, J = 9.0, 5.4 Hz, 1F).

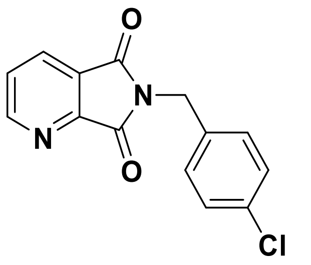

6-[(4-Chlorophenyl)methyl]-5*H*-pyrrolo[3,4-*b*]pyridine-5,7(6*H*)-dione (S7).[17] Isolated yield: 80%. GC-MS (m/z, $M^{+\cdot}$ 273), major peaks found: 274 (28%), 272 (88%), 246 (8%), 244 (27%), 217 (11%), 215 (32%), 181 (18%), 107 (21%), 79 (100 %), 50 (14 %). The NMR spectrum is consistent with literature. ^{1}H NMR (300 MHz, CD_3CN) *δ*: 8.94 (dd, *J* = 4.9, 1.5 Hz, 1H), 8.18 (dd, *J* = 7.7, 1.5 Hz, 1H), 7.69 (dd, *J* = 7.7, 4.9 Hz, 1H), 7.43 – 7.30 (m, 4H), 4.83 (s, 2H). ^{13}C RMN (75 MHz, CD_3CN) *δ*: 167.4, 167.2, 156.1, 152.3, 136.4, 133.9, 132.1, 130.7, 129.5, 128.7, 128.6, 41.6. HRMS (ESI+) [M+H+; calculated for $C_{14}H_{10}ClN_2O_2$ (^{35}Cl): 273.0353] found m/z 273.0433, [M+H+; calculated for $C_{14}H_{10}ClN_2O_2$ (^{37}Cl): 275.0323] found m/z 275.0406.

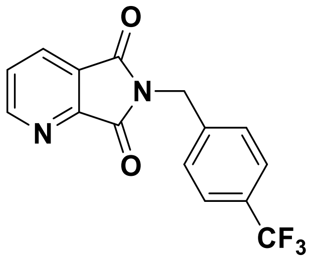

6-(4-(Trifluoromethyl)benzyl)-5*H*-pyrrolo[3,4-*b*]pyridine-5,7(6*H*)-dione (S8). Isolated yield: 62%. GC-MS (m/z, $M^{+\cdot}$ 306), major peaks found: 306 (100%), 249 (51%), 223 (23%), 79 (84%), 77 (28%). ^{1}H NMR (400 MHz, CD_3CN) *δ*: 8.95 (dd, *J* = 5.0, 1.5 Hz, 1H), 8.20 (dd, *J* = 7.7, 1.5 Hz, 1H), 7.70 (dd, *J* = 7.7, 5.0 Hz, 1H), 7.66 (d, *J* = 7.9 Hz, 2H), 7.56 (d, *J* = 8.1 Hz, 2H), 4.92 (s, 2H). ^{13}C NMR (75 MHz, CD_3CN) *δ:*167.5, 167.3, 156.2, 152.9, 142.0, 132.2, 129.5, 128.8, 128.7, 128.5 (q, $J^{1}_{C\text{-}F}$ = 271.2 Hz), 126.5 (q, *J* = 3.8 Hz), 41.9. ^{19}F NMR (282 MHz, CD_3CN) *δ*: -63.08 (s, 3F). HRMS (ESI+) [$M+H^+$; calculated for $C_{15}H_{10}F_3N_2O_2$: 307.0689] found m/z 307.0696.

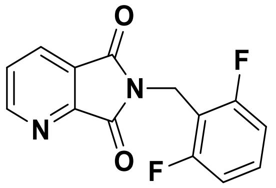

6-(2,6-Difluorobenzyl)-5*H*-pyrrolo[3,4-*b*]pyridine-5,7(6*H*)-dione (S9). Isolated yield: 60%. GC/MS (m/z, $M^{+\cdot}$ 274), major peaks found: 274 (100%), 199 (20%), 127 (33%), 106 (24%), 79 (92%), 50 (17%). ^{1}H NMR (300 MHz, CD_3CN) *δ*: 8.92 (dd, *J* = 4.9, 1.5 Hz, 1H), 8.16 (dd, *J* = 7.7, 1.5 Hz, 1H), 7.68 (dd, *J* = 7.7, 5.0 Hz, 1H), 7.36 (tt, *J* = 8.5, 6.5 Hz, 1H), 7.05 – 6.90 (m, 2H), 4.92 (s, 2H). ^{13}C NMR (75 MHz, CD_3CN) *δ*: 166.8, 166.6, 162.6 (d, $J^{1}_{C\text{-}F}$ = 249.0 Hz), 162.5 (d, $J^{1}_{C\text{-}F}$ = 249.1 Hz), 156.1, 152.7, 132.1, 131.4 (t, $J^{3}_{C\text{-}F}$ = 10.5 Hz), 128.7, 128.4, 112.7 (t, $J^{2}_{C\text{-}F}$ = 18.5 Hz), 112.4 (d, $J^{2}_{C\text{-}F}$ = 17.8 Hz), 112.3 (d, $J^{2}_{C\text{-}F}$ = 17.6 Hz), 30.8 (t, $J^{3}_{C\text{-}F}$ = 4.5 Hz). ^{19}F NMR (282 MHz, CD_3CN) *δ*: -115.10-(-115.19) (m, 2F). HRMS (ESI+) [$M+H^+$; calculated for $C_{14}H_9F_2N_2O_2$: 275.0554] found m/z 275.0635.

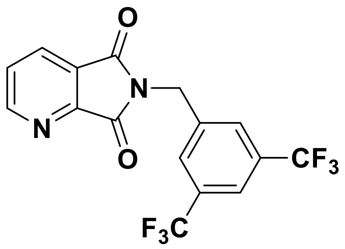

6-(3,5-Bis(trifluoromethyl)benzyl)-5*H*-pyrrolo[3,4-*b*]pyridine-5,7(6*H*)-dione (S10). Isolated yield: 70%. GC-MS (m/z, $M^{+\cdot}$ 374), major peaks found: 374 (90%), 317 (21%), 291 (16%), 105 (19%), 79 (100%), 50 (12%). ^{1}H NMR (400 MHz, CD_3CN) *δ*: 8.95 (dd, *J* = 5.0, 1.5 Hz, 1H), 8.20 (dd, *J* = 7.7, 1.5 Hz, 1H), 7.96 (s, 2H), 7.94 (s, 1H), 7.71 (dd, *J* = 7.7, 5.0 Hz, 1H), 4.99 (s, 2H). ^{13}C NMR (75 MHz, CD_3CN) *δ*: 167.4, 167.3, 156.2, 152.9, 140.4, 132.2, 132.2, 129.8, 128.7, 128.6, 124.5, 122.7, 41.5. ^{19}F NMR (377 MHz, CD_3CN) δ: -63.4 (s, 6F). HRMS (ESI+) [M+H$^+$; calculated for $C_{16}H_9F_6N_2O_2$: 375.0490] found m/z 375.0575.

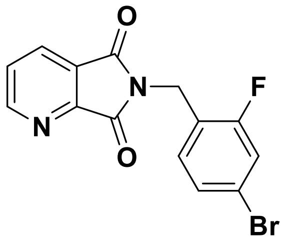

6-(4-Bromo-2-fluorobenzyl)-5*H*-pyrrolo[3,4-*b*]pyridine-5,7(6*H*)-dione (S11).[18] Isolated yield: 80%. GC-MS (m/z, $M^{+\cdot}$ 335), major peaks found: 336 (59%), 334 (61%), 279 (14%), 277 (14%), 199 (16%), 107 (45%), 79 (100%), 50 (20%). The NMR spectrum is consistent with literature. ^{1}H NMR (400 MHz, CD_3CN) *δ*: 8.93 (dd, *J* = 5.0, 1.5 Hz, 1H), 8.17 (dd, *J* = 7.7, 1.5 Hz, 1H), 7.68 (dd, *J* = 7.7, 5.0 Hz, 1H), 7.45 – 7.27 (m, 3H), 4.85 (s, 2H). ^{13}C NMR (75 MHz, CD_3CN) *δ*: 167.2, 167.0, 161.3 (d, $J^{1}_{C\text{-}F}$ = 250.9 Hz), 156.2, 152.8, 132.9 (d, $J^{3}_{C\text{-}F}$ = 4.7 Hz), 128.7, 128.51 (d, $J^{4}_{C\text{-}F}$ = 3.3 Hz), 123.7 (d, $J^{3}_{C\text{-}F}$ = 14.9 Hz), 122.4 (d, $J^{2}_{C\text{-}F}$ = 9.6 Hz), 119.8 (d, $J^{2}_{C\text{-}F}$ = 25.2 Hz), 35.8 (d, $J^{3}_{C\text{-}F}$ = 4.5 Hz). ^{19}F NMR (377 MHz, CD_3CN) *δ*: -115.71-(-115.90) (m, 1F).

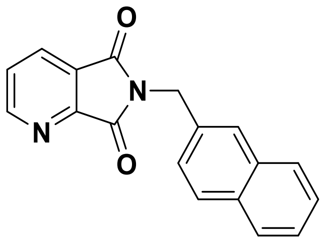

6-(Naphthalen-2-ylmethyl)-5*H*-pyrrolo[3,4-*b*]pyridine-5,7(6*H*)-dione (S12). Isolated yield: 45%. GC-MS (m/z, $M^{+\cdot}$ 288), major peaks found: 288 (100%), 260 (49%) 231 (59%), 79 (34%). ^{1}H NMR (400 MHz, CD_3CN) *δ*: 8.94 (dd, *J* = 5.0, 1.5 Hz, 1H), 8.19 (dd, *J* = 7.7, 1.5 Hz, 1H), 7.89 – 7.82 (m, 4H), 7.69 (dd, *J* = 7.7, 5.0 Hz, 1H), 7.52 (dd, *J* = 8.4, 1.8 Hz, 1H), 7.50 – 7.46 (m, 2H), 5.01 (s, 2H). ^{13}C NMR (100 MHz, CD_3CN) *δ*: 167.6, 167.3, 156.1, 152.9, 135.0, 134.2, 133.7, 132.1, 129.3, 128.7, 128.6, 128.5, 127.3, 127.2, 127.1, 126.9, 42.4. HRMS (ESI+) [M+H$^+$; calculated for $C_{18}H_{13}N_2O_2$: 289.0972] found m/z 289.0980.

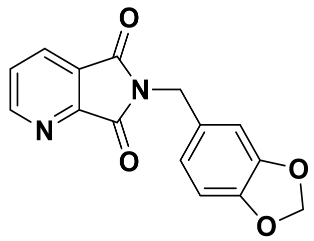

6-(Benzo[*d*][1,3]dioxol-5-ylmethyl)-5*H*-pyrrolo[3,4-*b*]pyridine-5,7(6*H*)-dione (S13).[19] Isolated yield: 70%. GC-MS (m/z, $M^{+\cdot}$ 282), major peaks found: 282 (100%), 254 (33%), 225 (31%), 135 (13%), 79 (51%), 51 (14%). The NMR spectrum is consistent with literature. ^{1}H NMR (400 MHz, CD_3CN) *δ*: 8.93 (dd, *J* = 5.0, 1.5 Hz, 1H), 8.17 (dd, *J* = 7.7, 1.5 Hz, 1H), 7.68 (dd, *J* = 7.7, 5.0 Hz, 1H), 6.90 – 6.83 (m, 2H), 6.82 – 6.75 (m, 1H), 5.93 (s, 2H), 4.75 (s, 2H). ^{13}C NMR (100 MHz, CD_3CN) *δ*: 167.5, 167.3, 156.0, 153.0, 148.8, 148.1, 132.0, 131.3, 128.6, 122.4, 121.9, 109.2, 102.4, 42.0. HRMS (ESI+) [M+H$^+$; calculated for $C_{15}H_{11}N_2O_4$: 283.0641] found m/z 283.0717.

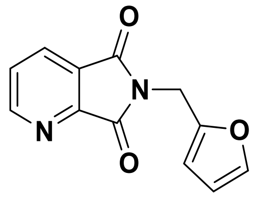

6-(Furan-2-ylmethyl)-5*H*-pyrrolo[3,4-*b*]pyridine-5,7(6*H*)-dione (S14).[20] Isolated yield: 77%. GC-MS (m/z, $M^{+\cdot}$ 228), major peaks found: 228 (100%), 200 (34%), 1711 (57%), 144 (24%), 79 (40%). The NMR spectrum is consistent with literature. ^{1}H NMR (400 MHz, CD_3CN) *δ*: 8.93 (dd, *J* = 4.9, 1.5 Hz, 1H), 8.18 (dd, *J* = 7.7, 1.5 Hz, 1H), 7.69 (dd, *J* = 7.7, 5.0 Hz, 1H), 7.42 (dd, *J* = 1.7, 1.0 Hz, 1H), 6.39 – 6.35 (m, 2H), 4.84 (s, 2H). ^{13}C NMR (100 MHz, CD_3CN) *δ*: 167.0, 166.8, 156.1, 152.7, 150.5, 143.5, 132.1, 128.7, 128.5, 111.5, 109.4, 35.2.

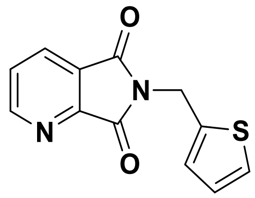

6-(Thiophen-2-ylmethyl)-5*H*-pyrrolo[3,4-*b*]pyridine-5,7(6*H*)-dione (S15).[21] Isolated yield: 78%. GC-MS (m/z, $M^{+\cdot}$ 245), major peaks found: 244 (100%), 216 (23%), 187 (42%), 161 (18%), 79 (69%), 50 (11%). The NMR spectrum is consistent with literature. ^{1}H NMR (400 MHz, CD_3CN) δ: 8.93 (dd, *J* = 5.0, 1.5 Hz, 1H), 8.18 (dd, *J* = 7.7, 1.5 Hz, 1H), 7.68 (dd, *J* = 7.7, 5.0 Hz, 1H), 7.32 (dd, *J* = 5.1, 1.2 Hz, 1H), 7.12 (dd, *J* = 3.5, 1.1 Hz, 1H), 6.96 (dd, *J* = 5.1, 3.5 Hz, 1H), 5.01 (s, 2H). ^{13}C NMR (75 MHz, CD_3CN) *δ*: 167.0, 166.8, 156.1, 152.8, 139.4, 132.2, 128.7, 128.5, 128.4, 127.9, 127.0, 36.7. HRMS (ESI+) [M+H$^+$; calculated for $C_{12}H_9N_2O_2S$: 245.0306] found m/z 245.0392.

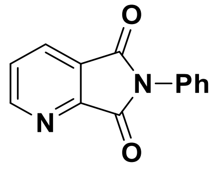

6-Phenyl-5*H*-pyrrolo[3,4-*b*]pyridine-5,7(6*H*)-dione (S16).[22] Isolated yield: 63%. GC-MS (m/z, $M^{+\cdot}$ 224), major peaks found: 224 (100%), 180 (41%), 105 (28%), 77 (63%), 51 (17%). The NMR spectrum is consistent with literature. ^{1}H NMR (300 MHz, $CDCl_3$) *δ*: 9.06 (dd, *J* = 5.0, 1.5 Hz, 1H), 8.29 (dd, *J* = 7.7, 1.5 Hz, 1H), 7.70 (dd, *J* = 7.7, 5.0 Hz, 1H),

7.56 – 7.50 (m, 2H), 7.48 – 7.41 (m, 3H).^{13}C NMR (75 MHz, $CDCl_3$) *δ*: 165.4, 165.3, 156.0, 151.4, 131.8, 131.2, 129.4, 128.7, 127.9, 127.2, 126.7.

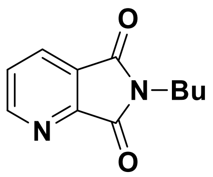

6-Butyl-5*H*-pyrrolo[3,4-*b*]pyridine-5,7(6*H*)-dione (S17).[23] Isolated yield: 20%. GC-MS (m/z, $M^{+\cdot}$ 204), major peaks found: 204 (37%), 161 (100%), 134 (17%), 106 (43%), 78 (39%), 50 (11%). The NMR spectrum is consistent with literature. ^{1}H NMR (300 MHz, CD_3CN) *δ*: 8.91 (dd, *J* = 5.0, 1.5 Hz, 1H), 8.15 (dd, *J* = 7.7, 1.5 Hz, 1H), 7.66 (dd, *J* = 7.6, 5.0 Hz, 1H), 3.66 (t, *J* = 7.1 Hz, 2H), 1.68 – 1.58 (m, 2H), 1.42 – 1.29 (m, 2H), 0.93(t, *J* = 7.3 Hz, 3H). ^{13}C NMR (75 MHz, CD_3CN) *δ*: 167.8, 167.6, 155.9, 153.1, 131.8, 128.6, 128.5, 38.6, 31.2, 20.8, 13.9.

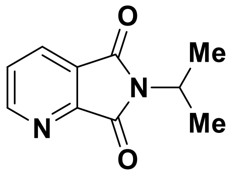

6-Isopropyl-5*H*-pyrrolo[3,4-*b*]pyridine-5,7(6*H*)-dione (S18).[24] Isolated yield: 42%. GC-MS (m/z, $M^{+\cdot}$ 190), major peaks found: 190 (52%), 175 (100%), 162 (17%), 148 (38%), 131 (24%), ^{1}H NMR (400 MHz, CD_3CN) *δ*: 8.90 (dd, *J* = 5.0, 1.5 Hz, 1H), 8.13 (dd, *J* = 7.7, 1.5 Hz, 1H), 7.66 (dd, *J* = 7.7, 5.0 Hz, 1H), 4.50 (h, *J* = 6.9 Hz, 1H), 1.46 (d, *J* = 6.9 Hz, 6H). ^{13}C NMR (75 MHz, CD_3CN) *δ*: 167.6, 163.4, 155.9, 152.8, 131.7, 128.4, 44.0, 20.3.

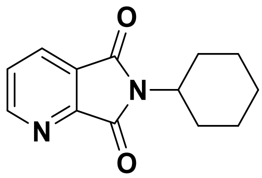

6-Cyclohexyl-5*H*-pyrrolo[3,4-*b*]pyridine-5,7(6*H*)-dione (S19).[24] Isolated yield: 50%. GC-MS (m/z, $M^{+\cdot}$ 230), major peaks found: 230 (43%), 187 (25%), 149 (100%), 131 (26%). The NMR spectrum is consistent with literature. ^{1}H NMR (400 MHz, CD_3CN) *δ*: 8.90 (dd, *J* = 5.0, 1.5 Hz, 1H), 8.12 (dd, *J* = 7.7, 1.5 Hz, 1H), 7.65 (dd, *J* = 7.7, 5.0 Hz, 1H), 4.10 (tt, *J* = 12.4, 2.9 Hz, 1H), 2.21 – 2.07 (m, 2H), 1.89 – 1.66 (m, 5H), 1.47 – 1.17 (m, 3H). ^{13}C NMR (75 MHz, CD_3CN) *δ*: 167.6, 163.5, 155.9, 152.7, 131.7, 128.5, 128.4, 51.9, 30.6, 26.7, 26.1.

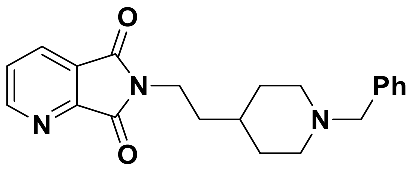

6-(2-(1-Benzylpiperidin-4-yl)ethyl)-5H-pyrrolo[3,4-b]pyridine-5,7(6H)-dione (S20). Isolated yield: 85%. GC-MS (m/z, $M^{+\cdot}$ 349), major peaks found: 349 (85%), 272 (14%), 258 (32%), 188 (41%), 91 (100%). ^{1}H NMR (400 MHz, $CDCl_3$) *δ*: 8.96 (dd, *J* = 4.9, 1.5 Hz, 1H), 8.15 (dd, *J* = 7.6, 1.5 Hz, 1H), 7.60 (dd, *J* = 7.6, 5.0 Hz, 1H), 7.32 – 7.28 (m, 4H), 7.25 – 7.21 (m, 1H), 3.78 (d, *J* = 7.5 Hz, 2H), 3.48 (s, 2H), 2.93 – 2.81 (m, 2H), 1.99 – 1.87 (m, 2H), 1.79 – 1.59 (m, 5H), 1.34 – 1.26 (m, 2H). ^{13}C NMR (100 MHz, $CDCl_3$) *δ*: 166.4, 166.3, 155.3, 153.0, 138.5, 131.2, 129.4, 128.3, 127.5, 127.4, 127.1, 63.6, 53.8, 36.3, 35.2, 33.6, 32.1. HRMS (ESI+) [M+H^{+}; calculated for $C_{21}H_{24}N_3O_2$: 350.1870] found m/z 350.1863.

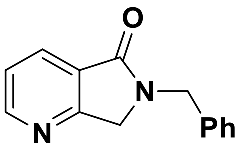

6,7-Dihydro-6-(phenylmethyl)-5*H*-pyrrolo[3,4-*b*]pyridin-5-one (2).[25] Isolated yield: 95%. GC-MS (m/z, $M^{+\cdot}$ 224), major peaks found: 224 (100%), 147 (20%), 120 (32%), 91 (77%), 65 (30%), 39 (8%). (The NMR spectrum is consistent with the reported data). ^{1}H NMR (400 MHz, CD_3CN) *δ*: 8.69 (dd, *J* = 5.0, 1.6, 1H), 8.06 (dd, *J* = 7.7, 1.6, 1H), 7.44 (dd, *J* = 7.7, 5.0, 1H), 7.40-7.25 (m, 5H), 4.78 (s, 2H), 4.34 (s, 2H). ^{13}C NMR (100 MHz, CD_3CN) *δ*: 167.5, 163.7, 153.5, 138.4, 132.1, 129.7, 128.9, 128.5, 127.1, 124.2, 52.1, 46.6.

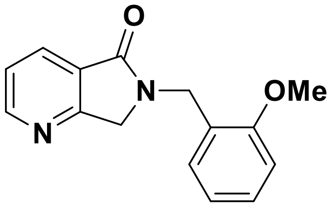

6-(2-Methoxybenzyl)-6,7-dihydro-5*H*-pyrrolo[3,4-*b*]pyridin-5-one (6). Isolated yield: 82%. GC-MS (m/z, $M^{+\cdot}$ 254), major peaks found: 254 (100%), 225 (55%), 121 (85%), 91 (68%). ^{1}H NMR (400 MHz, CD_3CN) *δ*: 8.69 (dd, *J* = 5.0, 1.6 Hz, 1H), 8.04 (dd, *J* = 7.7, 1.6 Hz, 1H), 7.44 (dd, *J* = 7.7, 5.0 Hz, 1H), 7.29 (td, *J* = 7.8, 1.8 Hz, 1H), 7.20 (dd, *J* = 7.5, 1.8 Hz, 1H), 7.00 (dd, J = 8.3, 1.1 Hz, 1H), 6.91 (td, J = 7.4, 1.1 Hz, 1H), 4.76 (s, 2H), 4.38 (s, 2H), 3.85 (s, 3H).^{13}C NMR (100 MHz, CD_3CN) *δ*: 167.5, 163.8, 158.6, 153.4, 132.0, 130.3, 130.0, 127.2, 126.0, 124.1, 121.5, 111.8, 56.2, 52.6, 41.9. HRMS (ESI+) [M+H$^+$; calculated for $C_{15}H_{15}N_2O_2$: 255.1128] found m/z 255.1125.

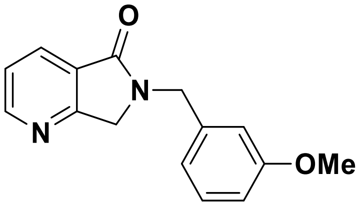

6-(3-Methoxybenzyl)-6,7-dihydro-5*H*-pyrrolo[3,4-*b*]pyridin-5-one (7). Isolated yield: 86%. GC-MS (m/z, $M^{+\cdot}$ 254), major peaks found: 253 (84%), 121 (100%), 92 (52%), 147 (40%). ^{1}H NMR (400 MHz, CD_3CN) *δ*: 8.70 (dd, *J* = 5.0, 1.6 Hz, 1H), 8.06 (dd, *J* = 7.7, 1.6 Hz, 1H), 7.44 (dd, *J* = 7.7, 5.0 Hz, 1H), 7.29 – 7.25 (m, 1H), 6.98 – 6.77 (m, 3H), 4.74 (s, 2H), 4.35 (s, 2H), 3.76 (s, 3H).^{13}C NMR (100 MHz, CD_3CN) *δ*: 167.5, 163.7, 161.0, 153.5, 140.0, 132.2, 130.8, 127.1, 124.1, 121.0, 114.4, 113.9, 55.9, 52.1, 46.6. HRMS (ESI+) [M+H$^+$; calculated for $C_{15}H_{15}N_2O_2$: 255.1128] found m/z 255.1137.

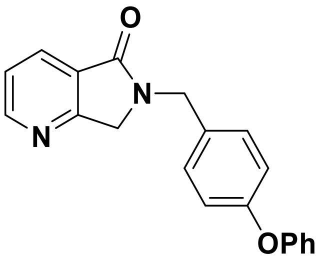

6-(4-Phenoxybenzyl)-6,7-dihydro-5*H*-pyrrolo[3,4-*b*]pyridin-5-one (8). Isolated yield: 90%. GC/MS (m/z, $M^{+\cdot}$ 316), major peaks found: 316 (100%), 223 (40%), 183 (39%), 120 (32%). ^{1}H NMR (400 MHz, CD_3CN) *δ*: 8.69 (dd, *J* = 5.0, 1.6 Hz, 1H), 8.05 (dd, *J* = 7.7, 1.6 Hz, 1H), 7.44 (dd, *J* = 7.7, 5.0 Hz, 1H), 7.40 – 7.29 (m, 4H), 7.13 (t, *J* = 7.4 Hz, 1H), 7.04 – 6.92 (m, 4H), 4.75 (s, 2H), 4.35 (s, 2H). ^{13}C NMR (75 MHz, CD_3CN) *δ*: 167.5, 163.7, 158.2, 157.7, 153.5, 133.4,

132.2, 130.6, 127.1, 124.5, 124.2, 119.9, 119.7, 52.0, 46.0. HRMS (ESI+) [M+H$^+$; calculated for $C_{20}H_{17}N_2O_2$: 317.1285] found m/z 317.1283.

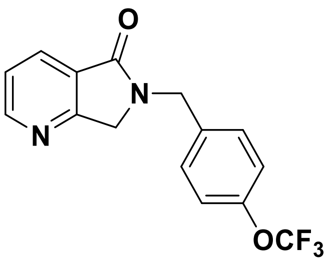

6-(4-(Trifluoromethoxy)benzyl)-6,7-dihydro-5*H*-pyrrolo[3,4-*b*]pyridin-5-one (9). Isolated yield: 79%. GC-MS (m/z, M$^{+\cdot}$ 308), major peaks found: 308 (100%), 223 (29%), 175 (46%), 120 (42%), 92 (28%). The NMR spectrum is consistent with the reported data ^{1}H NMR (400 MHz, CD_3CN) *δ:* 8.71 (dd, *J* = 5.0, 1.6 Hz, 1H), 8.07 (dd, *J* = 7.7, 1.6 Hz, 1H), 7.45 (dd, *J* = 7.7, 5.0 Hz, 1H), 7.42 (d, *J* = 7.7 Hz, 2H), 7.27 (d, *J* = 7.7 Hz, 2H), 4.79 (s, 2H), 4.37 (s, 2H).^{13}C NMR (100 MHz, CD_3CN) *δ*: 167.5, 163.7, 153.6, 137.8, 132.2, 130.6, 127.0, 124.2, 122.3, 52.1, 45.9. ^{19}F NMR (282 MHz, CD_3CN) *δ*: -58.67 (s, 3F). HRMS (ESI+) [M+H$^+$; calculated for $C_{15}H_{12}F_3N_2O_2$: 309.0845] found m/z 309.0840.

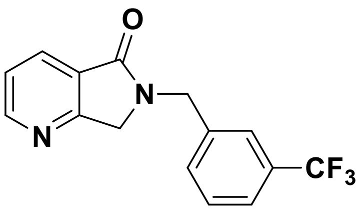

6-(3-(Trifluoromethyl)benzyl)-6,7-dihydro-5*H*-pyrrolo[3,4-*b*]pyridin-5-one (10). Isolated yield: 78%. GC-MS (m/z, M$^{+\cdot}$ 292), major peaks found: 292 (100%), 147 (35%), 133 (31%), 120 (53%), 92 (34%). ^{1}H NMR (300 MHz, CD_3CN) *δ*: 8.71 (dd, *J* = 5.0, 1.6 Hz, 1H), 8.08 (dd, *J* = 7.7, 1.6 Hz, 1H), 7.73 – 7.50 (m, 5H), 7.46 (dd, *J* = 7.7, 5.0 Hz, 1H), 4.85 (s, 2H), 4.38 (s, 2H).^{13}C NMR (75 MHz, CD_3CN) *δ*: 167.8, 163.7, 153.6, 139.9, 132.8, 132.3, 131.2 (d, $J^2_{C\text{-}F}$ = 22.5 Hz), 130.6, 128.8 (q, $J^1_{C\text{-}F}$ = 252), 125.6 (d, $J^3_{C\text{-}F}$ = 4.0 Hz), 125.3 (d, $J^3_{C\text{-}F}$ = 4.0 Hz), 124.2, 52.2, 46.3. ^{19}F NMR (282 MHz, CD_3CN) *δ*: -63.07 (s, 3F). HRMS (ESI+) [M+H$^+$; calculated for $C_{15}H_{12}F_3N_2O$: 293.0899] found m/z 293.0912.

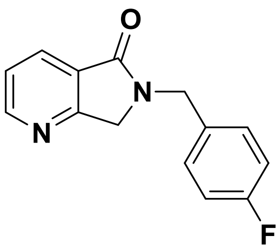

6-(4-Fluorobenzyl)-6,7-dihydro-5*H*-pyrrolo[3,4-*b*]pyridin-5-one (11).[26] Isolated yield: 73%. GC-MS (m/z, M$^{+\cdot}$ 242), major peaks found: 242 (100%), 120 (56%), 109 (60%), 92 (34%). The NMR spectrum is consistent with the reported data. ^{1}H NMR (400 MHz, CD_3CN) *δ*: 8.70 (dd, *J* = 4.9, 1.6 Hz, 1H), 8.06 (dd, *J* = 7.7, 1.6 Hz, 1H), 7.45 (dd, *J* = 7.7, 4.9 Hz, 1H), 7.35 (dd, *J* = 8.9, 5.6 Hz, 2H), 7.09 (t, J = 8.9 Hz, 2H), 4.75 (s, 2H), 4.34 (s, 2H).^{13}C NMR (100 MHz, CD_3CN) *δ*: 167.5, 163.7, 163.1 (d, $J^1_{C\text{-}F}$ = 243.3 Hz), 153.5, 134.5 (d, $J^4_{C\text{-}F}$ = 3.1 Hz), 132.2, 130.9 (d, $J^3_{C\text{-}F}$ = 8.3 Hz), 127.0, 124.2, 116.3 (d, $J^2_{C\text{-}F}$ = 21.6 Hz), 52.0, 45.9. ^{19}F NMR (377 MHz, CD_3CN) *δ*: -116.90 - (-116.98) (m, 1F).

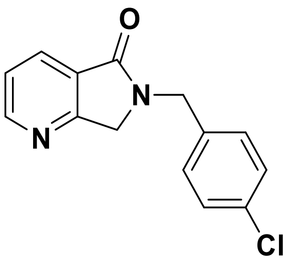

6-(4-Chlorobenzyl)-6,7-dihydro-5*H*-pyrrolo[3,4-*b*]pyridin-5-one (12).[26] Isolated yield: 67%. GC-MS (m/z, M+· 258), major peaks found: 258 (100 %), 223 (38 %), 147 (15 %), 127 (12 %), 125 (39 %), 92 (27 %), 65 (27 %), 39 (11 %). The NMR spectrum is consistent with the reported data. ^1H NMR (300 MHz, CD_3CN) δ: 8.68 (dd, J = 5.0, 1.6 Hz, 1H), 8.05 (dd, J = 7.7, 1.6 Hz, 1H), 7.43 (dd, J = 7.7, 5.0 Hz, 1H), 7.38 – 7.25 (m, 4H), 4.74 (s, 2H), 4.33 (s, 2H). ^{13}C NMR (75 MHz, CD_3CN) δ: 167.6, 163.6, 153.5, 137.3, 133.7, 132.2, 130.6, 129.6, 126.9, 124.1, 52.0, 45.9. HRMS (ESI+) [M+H$^+$; calculated for $C_{14}H_{12}ClN_2O$ (^{35}Cl): 259.0560] found m/z 259.0633, HRMS (ESI+) [M+H$^+$; calculated for $C_{14}H_{12}ClN_2O$ (^{37}Cl): 261.0530] found m/z 261.0603.

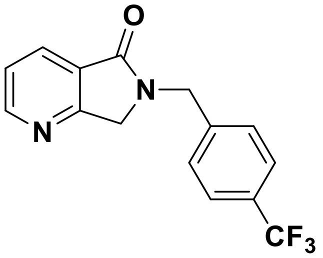

6-(4-(Trifluoromethyl)benzyl)-6,7-dihydro-5*H*-pyrrolo[3,4-*b*]pyridin-5-one (13). Isolated yield: 85%. GC-MS (m/z, $M^{+\cdot}$ 292), major peaks found: 292 (100%), 147 (31%), 133 (34%), 120 (43%). ^1H NMR (300 MHz, CD_3CN) δ: 8.72 (dd, J = 5.0, 1.6 Hz, 1H), 8.08 (dd, J = 7.7, 1.6 Hz, 1H), 7.67 (d, J = 8.0 Hz, 2H), 7.50 (d, J = 8.0 Hz, 2H), 7.46 (dd, J = 7.7, 5.0 Hz, 1H), 4.86 (s, 2H), 4.38 (s, 2H). ^{13}C NMR (75 MHz, CD_3CN) δ: 153.6, 132.2, 129.4, 126.5, 124.2, 52.2, 46.2. ^{19}F NMR (282 MHz, CD_3CN) δ: -62.99 (s, 3F). HRMS (ESI+) [M+H$^+$; calculated for $C_{15}H_{12}F_3N_2O$: 293.0896] found m/z 293.0904.

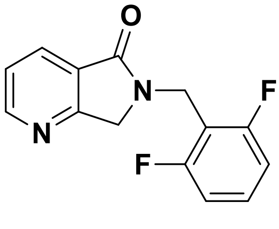

6-(2,6-Difluorobenzyl)-6,7-dihydro-5*H*-pyrrolo[3,4-*b*]pyridin-5-one (14).[26] Isolated yield: 83%. GC/MS (m/z, M+· 260), major peaks found: 260 (100 %), 147 (12 %), 120 (47 %), 92 (26 %), 65 (6 %). The NMR spectrum is consistent with the reported data. ^1H NMR (400 MHz, CD_3CN) δ: 8.67 (dd, J = 5.0, 1.6 Hz, 1H), 8.02 (dd, J = 7.7, 1.6 Hz, 1H), 7.41 (dd, J = 7.7, 5.0, 1H), 7.39 – 7.34 (m, 1H), 7.04 – 6.97 (m, 2H), 4.87 (s, 2H), 4.33 (s, 2H). ^{13}C NMR (75 MHz, CD_3CN) δ: 166.9, 163.5, 162.7 (d, $J^1_{C\text{-}F}$ = 248.1 Hz), 162.6 (d, $J^1_{C\text{-}F}$ = 248.0 Hz), 153.5, 132.1, 131.4 (t, $J^3_{C\text{-}F}$ = 10.5 Hz), 126.7, 124.1, 113.3 (t, $J^2_{C\text{-}F}$ = 19.8 Hz), 112.5 (d, $J^2_{C\text{-}F}$ = 17.8 Hz), 112.4 (d, $J^2_{C\text{-}F}$ = 17.6 Hz), 51.8, 34.2 (t, $J^3_{C\text{-}F}$ = 4.1 Hz). ^{19}F NMR (282 MHz, CD_3CN) δ: -115.86-(-115.94) (m, 2F). HRMS (ESI+) [M+H$^+$; calculated for $C_{14}H_{11}F_2N_2O$: 261.0761] found m/z 261.0837.

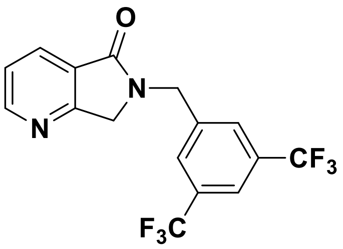

6-[[3,5-Bis(trifluoromethyl)phenyl]methyl]-5*H*-pyrrolo[3,4-*b*]pyridine-5-one (15). Isolated yield: 53%. GC-MS (m/z, $M^{+\cdot}$ 360), major peaks found: 360 (100%), 291 (11%), 227 (17%), 147 (21%), 120 (40%), 92 (29%). ^{1}H NMR (400 MHz, CD_3CN) δ: 8.72 (dd, J = 5.0, 1.6 Hz, 1H), 8.09 (dd, J = 7.7, 1.6 Hz, 1H), 7.94 (s, 1H), 7.92 (s, 2H), 7.47 (dd, J = 7.7, 5.0 Hz, 1H), 4.91 (s, 2H), 4.41 (s, 2H). ^{13}C NMR (100 MHz, CD_3CN) δ: 168.1, 163.8, 153.7, 141.8, 132.3, 132.2 (q, $J^{2}_{C\text{-}F}$ = 33.1 Hz), 129.6 (q, $J^{3}_{C\text{-}F}$ = 2.9 Hz), 126.7, 124.4 (q, $J^{1}_{C\text{-}F}$ = 271.5 Hz), 124.3, 122.4 (q, $J^{3}_{C\text{-}F}$ = 4.5 Hz), 52.4, 46.0. ^{19}F NMR (377 MHz, CD_3CN) δ: -63.33 (s, 6F). HRMS (ESI+) [M+H^{+}; calculated for $C_{16}H_{11}F_6N_2O$: 361.0697] found m/z 361.0770.

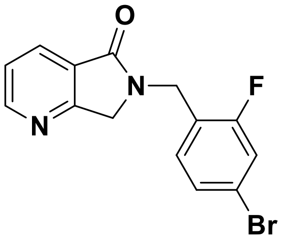

6-(4-Bromo-2-fluorobenzyl)-6,7-dihydro-5*H*-pyrrolo[3,4-*b*]pyridin-5-one (16).[18] Isolated yield: 77% GC-MS (m/z, $M^{+\cdot}$ 321), major peaks found: 322 (92 %), 320 (100 %), 241 (44 %), 189 (29 %), 187 (31 %), 147 (21 %), 120 (76 %), 92 (45 %), 65 (14 %). The NMR spectrum is consistent with the reported data. ^{1}H NMR (400 MHz, CD_3CN) δ: 8.70 (dd, J = 4.9, 1.6 Hz, 1H), 8.05 (dd, J = 7.6, 1.6 Hz, 1H), 7.44 (dd, J = 7.6, 4.9, 1H), 7.38 (dd, J = 9.6, 1.9 Hz, 1H), 7.34 (dd, J = 8.2, 1.9 Hz, 1H), 7.30 – 7.26 (m, 1H), 4.79 (s, 2H), 4.38 (s, 2H). ^{13}C NMR (100 MHz, CD_3CN) δ: 167.6, 163.7, 161.7 (d, $J^{1}_{C\text{-}F}$ = 250.0 Hz), 153.6, 132.9 (d, $J^{3}_{C\text{-}F}$ = 5.0 Hz), 132.2, 128.7 (d, $J^{4}_{C\text{-}F}$ = 3.7 Hz), 126.8, 124.7 (d, $J^{3}_{C\text{-}F}$ = 15.3 Hz), 124.1, 122.2 (d, $J^{2}_{C\text{-}F}$ = 9.5 Hz), 119.9 (d, $J^{2}_{C\text{-}F}$ = 25.4 Hz), 52.2, 40.2 (d, $J^{3}_{C\text{-}F}$ = 4.2). ^{19}F NMR (377 MHz, CD_3CN) δ: -116.76-(-116.90) (m, 1F). HRMS (ESI+) [M+H^{+}; calculated for $C_{14}H_{11}BrFN_2O$ (^{79}Br): 320.9961] found m/z 321.0026, [M+H^{+}; calculated for $C_{14}H_{11}BrFN_2O$ (^{81}Br): 322.9940] found m/z 323.0029.

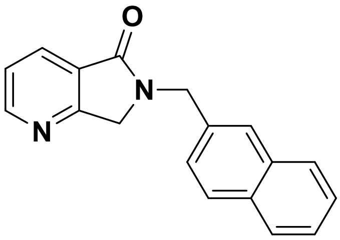

6-(Naphthalen-2-ylmethyl)-6,7-dihydro-5*H*-pyrrolo[3,4-*b*]pyridin-5-one (17). Isolated yield: 79%. GC-MS (m/z, $M^{+\cdot}$ 274), major peaks found: 274 (96%), 141 (100%), 115 (21%). ^{1}H NMR (400 MHz, CD_3CN) δ: 8.70 (dd, J = 5.0, 1.6 Hz, 1H), 8.09 (dd, J = 7.7, 1.6 Hz, 1H), 7.99 – 7.82 (m, 3H), 7.80 (s, 1H), 7.66 – 7.31 (m, 4H), 4.94 (s, 2H), 4.39 (s, 2H).^{13}C NMR (75 MHz, CD_3CN) δ: 167.6, 163.8, 153.5, 136.0, 134.4, 133.8, 132.2, 129.5, 128.7, 128.6, 127.4, 127.3, 127.1, 127.0, 126.9, 124.2, 52.1, 46.8. HRMS (ESI+) [M+H^{+}; calculated for $C_{18}H_{15}N_2O$: 275.1179] found m/z 275.1181.

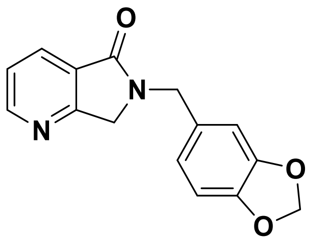

6-(1,3-Benzodioxol-5-ylmethyl)-5*H*-pyrrolo[3,4-*b*]pyridine-5-one (18). Isolated yield: 50%. GC-MS (m/z, $M^{+\cdot}$ 268), major peaks found: 268 (99%), 210 (9%), 181 (9%), 135 (100%), 105 (10%), 77 (23%), 51 (11%). ^{1}H NMR (400 MHz, CD_3CN) *δ*: 8.68 (dd, *J* = 4.9, 1.6 Hz, 1H), 8.05 (dd, *J* = 7.7, 1.6 Hz, 1H), 7.43 (dd, *J* = 7.7, 4.9 Hz, 1H), 6.83 – 6.80 (m, 3H), 5.92 (s, 2H), 4.65 (s, 2H), 4.32 (s, 2H). ^{13}C NMR (75 MHz, CD_3CN) *δ*: 167.5, 163.7, 153.5, 149.1, 148.1, 132.2, 132.1, 127.1, 124.2, 122.4, 109.4, 109.2, 102.4, 51.9, 46.4. HRMS (ESI+) [M+H$^+$; calculated for $C_{15}H_{13}N_2O_3$: 269.0848] found m/z 269.0921.

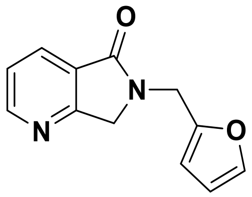

6-(Furan-2-ylmethyl)-6,7-dihydro-5H-pyrrolo[3,4-b]pyridin-5-one (19).[16] Isolated yield: 56%. GC-MS (m/z, $M^{+\cdot}$ 214), major peaks found: 214 (100%), 197 (26%), 185 (41%), 157 (35%), 133 (39%), 81 (34%). (The NMR spectrum is consistent with the reported data). ^{1}H NMR (400 MHz, CD_3CN) *δ*: 8.70 (dd, *J* = 4.9, 1.6 Hz, 1H), 8.04 (dd, *J* = 7.7, 1.6 Hz, 1H), 7.46 – 7.42 (m, 2H), 6.39 – 6.37 (m, 2H), 4.76 (s, 2H), 4.39 (s, 2H). ^{13}C NMR (100 MHz, CD_3CN) *δ*: 167.2, 163.6, 153.6, 151.6, 143.8, 132.2, 126.9, 124.2, 111.5, 109.4, 52.1, 39.4.

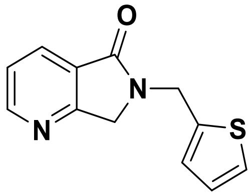

6-(2-Thienylmethyl)-5*H*-pyrrolo[3,4-*b*]pyridine-5-one (20). Isolated yield: 42%. GC-MS (m/z, $M^{+\cdot}$ 230), major peaks found: 230 (100%), 197 (21%), 146 (4%), 120 (20%), 97 (40%), 65 (7%), 39 (4%). ^{1}H NMR (400 MHz, CD_3CN) *δ*: 8.70 (dd, *J* = 5.0, 1.6 Hz, 1H), 8.05 (dd, *J* = 7.7, 1.6 Hz, 1H), 7.44 (dd, *J* = 7.7, 5.0 Hz, 1H), 7.33 (dd, *J* = 5.1, 1.2 Hz, 1H), 7.09 (dd, *J* = 3.5, 1.2 Hz, 1H), 6.99 (dd, J = 5.1, 3.5 Hz, 1H), 4.94 (s, 2H), 4.40 (s, 2H). ^{13}C NMR (100 MHz, CD_3CN) *δ*: 167.2, 163.6, 153.6, 140.8, 132.3, 128.1, 127.9, 126.9, 126.8, 124.2, 51.8, 41.2. HRMS (ESI+) [M+H$^+$; calculated for $C_{12}H_{11}N_2OS$: 231.0514] found m/z 231.0589.

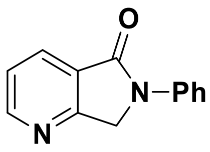

6-Phenyl-6,7-dihydro-5*H*-pyrrolo[3,4-*b*]pyridin-5-one (21). ^{1}H NMR yield: 50%. GC-MS (m/z, $M^{+\cdot}$ 210), major peaks found: 210 (100%), 181 (26%), 77 (19%). ^{1}H NMR (400 MHz, CD_3OD) *δ*: 8.81 (dd, *J* = 5.0, 1.5 Hz, 1H), 8.26 (dd, *J* = 7.7, 1.5 Hz, 1H), 7.89 (d, *J* = 7.7 Hz, 2H), 7.61 (dd, *J* = 7.7, 5.0 Hz, 1H), 7.50 – 7.46 (m, 2H), 7.7.30 – 7.24 (m, 1H), 5.07 (s, 2H).

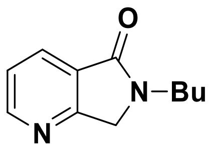

6-Butyl-5*H*-pyrrolo[3,4-*b*]pyridine-5-one (22). Isolated yield: 79%. GC-MS (m/z, $M^{+\cdot}$ 190), major peaks found: 190 (27%), 168 (4%), 147 (100%), 120 (15%), 92 (17%), 65 (8%), 39 (3%). ^{1}H NMR (300 MHz, CD_3CN) δ: 8.69 (dd, J = 4.9, 1.6 Hz, 1H), 8.00 (dd, J = 7.7, 1.6 Hz, 1H), 7.42 (dd, J = 7.7, 4.9 Hz, 1H), 4.41 (s, 2H), 3.58 (t, J = 7.2 Hz, 2H), 1.72 – 1.57 (m, 2H), 1.42 – 1.27 (m, 2H), 0.94 (t, J = 7.3 Hz, 3H). ^{13}C NMR (75 MHz, CD_3CN) δ: 167.3, 163.8, 153.2, 131.9, 127.5, 124.1, 52.1, 42.4, 31.0, 20.7, 14.0. HRMS (ESI+) [M+H$^+$; calculated for $C_{11}H_{15}N_2O$: 191.1106] found m/z 191.1176.

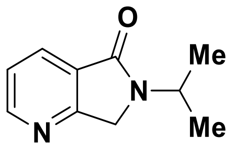

6-Isopropyl-6,7-dihydro-5*H*-pyrrolo[3,4-*b*]pyridin-5-one (23). Isolated yield: 57%. GC-MS (m/z, $M^{+\cdot}$ 176), major peaks found: 176 (22 %), 161 (100%), 120 (39%), 92 (32%), 65 (10%). ^{1}H NMR (400 MHz, CD_3CN) δ: 8.69 (dd, J = 5.0, 1.6 Hz, 1H), 8.00 (dd, J = 7.7, 1.6 Hz, 1H), 7.42 (dd, J = 7.7, 5.0 Hz, 1H), 4.56 (hept, J = 6.8 Hz, 1H), 4.39 (s, 2H), 1.28 (d, J = 6.8 Hz, 6H). ^{13}C NMR (75 MHz, CD_3CN) δ: 166.7, 163.9, 153.2, 131.8, 127.7, 124.0, 47.6, 43.5, 20.8. HRMS (ESI+) [M+H$^+$; calculated for $C_{10}H_{13}N_2O$: 177.1022] found m/z 177.1023.

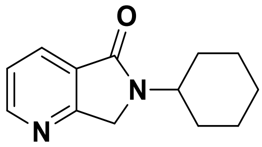

6-Cyclohexyl-6,7-dihydro-5H-pyrrolo[3,4-b]pyridin-5-one (24).[16] Isolated yield: 62%. GC-MS (m/z, $M^{+\cdot}$ 216), major peaks found: 216 (69%), 173 (100%), 135 (91%). ^{1}H NMR (400 MHz, CD_3CN) δ: 8.69 (dd, J = 5.0, 1.6 Hz, 1H), 8.00 (dd, J = 7.7, 1.6 Hz, 1H), 7.42 (dd, J = 7.7, 5.0 Hz, 1H), 4.40 (s, 2H), 4.13 (tt, J = 11.8, 3.7 Hz, 1H), 1.88 – 1.80 (m, 4H), 1.73 – 1.38 (m, 6H). ^{13}C NMR (100 MHz, CD_3CN) δ: 166.7, 164.0, 153.1, 131.8, 127.7, 124.0, 51.6, 48.6, 31.9, 26.4, 26.2.

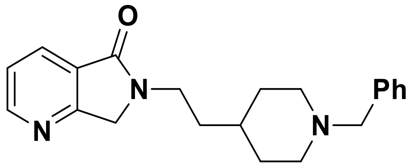

6-(2-(1-Benzylpiperidin-4-yl)ethyl)-6,7-dihydro-5H-pyrrolo[3,4-b]pyridin-5-one (26).[27] ^{1}H NMR yield: 70%. GC-MS (m/z, $M^{+\cdot}$ 335), major peaks found: 335 (16%), 188 (19%), 244 (100%), 91 (56%). ^{1}H NMR (400 MHz, CD_3OD) δ: 8.72 (dd, J = 5.0, 1.5 Hz, 1H), 8.14 (dd, J = 7.7, 1.5 Hz, 1H), 7.54 (dd, J = 7.7, 5.0 Hz, 1H), 7.32 – 7.29 (m, 5H), 4.54 (s, 2H), 3.70 (t, J = 7.4 Hz, 2H), 3.49 (s, 2H), 2.94 – 2.84 (m, 2H), 1.99 – 1.87 (m, 2H), 1.85 – 1.65 (m, 5H), 1.40 – 1.30 (m, 2H).

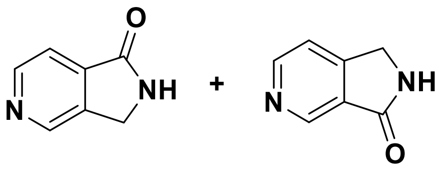

(86%) **(14%)**

2,3-Dihydro-1*H*-pyrrolo[3,4-*c*]pyridine-1-one/1,2-dihydro-3*H*-pyrrolo[3,4-*c*]pyridin-3-one[28] **(25).** Isolated yield: 74%. Both compounds were obtained as an inseparable mixture of regioisomers with a (86:14) ratio. GC-MS (m/z, $M^{+\cdot}$ 134), major peaks found (only one peak was detected by GC for major and minor regioisomer): 135 (100%), 106 (73%), 78 (50%), 50 (29%), 28 (9%). HRMS (ESI+) [M+H^+; calculated for $C_7H_7N_2O$: 135.0560] found m/z 135.0563 (is the same for both regioisomers).

For major regioisomer: ^{1}H NMR (400 MHz, CD_3OD) δ: 9.07 (d, J = 1.0, 1H), 8.83 (d, J = 5.3, 1H), 7.73 (dd, J = 5.2, 1.0, 1H), 5.47 (s, 2H). ^{13}C NMR (100 MHz, CD_3OD) δ: 171.2, 158.0, 153.9, 148.2, 123.9, 119.6, 71.2.

For minor regioisomer: ^{1}H NMR (400 MHz, CD_3OD) δ: 9.01 (s, 1H), 8.69 (d, J = 5.3, 1H), 7.84 (dd, J = 5.2, 0.8, 1H), 5.02 (s, 2H). ^{13}C NMR (100 MHz, CD_3OD) δ: 167.2, 156.1, 153.4, 151.5, 125.0, 122.6, 62.2.

7. REFERENCES

1. Lluna-Galán, C.; Arango-Daza, J. C.; Gómez, D.; Concepción, P.; Sun, R.; Calvino, J. J.; Simonelli, L.; Adam, R.; Cabrero-Antonino, J. R. Building Lactams by Highly Selective Hydrodeoxygenation of Cyclic Imides Using an Alumina-Supported AgRe Bimetallic Nanocatalyst. *Nat. Commun.* **2025**, *16*, 4119.

2. Wu, C.; Wang, J.; Zhang, X.; Zhang, R.; Ma, B. Highly Chemoselective Hydrogenation of Cyclic Imides to ω-Hydroxylactams or ω-Hydroxyamides Catalyzed by Iridium Catalysts. *Org. Chem. Front.* **2021**, *8*, 6530-6534.

3. Sugimoto, H.; Tsuchiya, Y.; Sugumi, H.; Higurashi, K.; Karibe, N.; Iimura, Y.; Sasaki, A.; Araki, S.; Yamanishi, Y.; Yamatsu, K. Synthesis and Structure-Activity Relationships of Acetylcholinesterase Inhibitors: 1-Benzyl-4-(2-Phthalimidoethyl)Piperidine, and Related Derivatives. *J. Med. Chem.* **1992**, *35*, 4542-4548.

4. Brütsch, L.; Feldmann, C. Synthesis and Morphology of $AgReO_4$ Plates, Rods, and Stars. *Z. anorg. allg. Chem.* **2017**, *643*, 789-792.

5. Otto, J. W.; Vassiliou, J. K.; Porter, R. F.; Ruoff, A. L. Raman Study of $AgReO_4$ in the Scheelite Structure Under Pressure. *Phys. Rev. B* **1991**, *44*, 9223-9227.

6. Goudarzi, M.; Mir, N.; Mousavi-Kamazani, M.; Bagheri, S.; Salavati-Niasari, M. Biosynthesis and Characterization of Silver Nanoparticles Prepared from Two Novel Natural Precursors by Facile Thermal Decomposition Methods. *Sci. Rep.* **2016**, *6*, 32539.

7. Ali, M. H.; Azad, M. A. K.; Khan, K. A.; Rahman, M. O.; Chakma, U.; Kumer, A. Analysis of Crystallographic Structures and Properties of Silver Nanoparticles Synthesized Using PKL Extract and Nanoscale Characterization Techniques. *ACS Omega* **2023**, *8*, 28133-28142.

8. She, X.; Flytzani-Stephanopoulos, M. The Role of AgOAl Species in Silver–Alumina Catalysts for the Selective Catalytic Reduction of NO_x with Methane. *J. Catal.* **2006**, *237*, 79-93.

9. Lwin, S.; Keturakis, C.; Handzlik, J.; Sautet, P.; Li, Y.; Frenkel, A. I.; Wachs, I. E. Surface ReO_x Sites on Al_2O_3 and their Molecular Structure–Reactivity Relationships for Olefin Metathesis. *ACS Catal.* **2015**, *5*, 1432-1444.

10. López-Hernández, I.; García, C.; Truttmann, V.; Pollitt, S.; Barrabés, N.; Rupprechter, G.; Rey, F.; Palomares, A. E. Evaluation of the Silver Species Nature in Ag-ITQ2 Zeolites by the CO Oxidation Reaction. *Catal. Today* **2020**, *345*, 22-26.

11. Zhang, B.; Lwin, S.; Xiang, S.; Frenkel, A. I.; Wachs, I. E. Tuning the Number of Active Sites and Turnover Frequencies by Surface Modification of Supported $ReO_4/(SiO_2–Al_2O_3)$ Catalysts for Olefin Metathesis. *ACS Catal.* **2021**, *11*, 2412-2421.

12. Zhang, B.; Wachs, I. E. Identifying the Catalytic Active Site for Propylene Metathesis by Supported ReO_x Catalysts. *ACS Catal.* **2021**, *11*, 1962-1976.

13. Izquierdo-Aranda, L.; Adam, R.; Cabrero-Antonino, J. R. Silver Supported Nanoparticles on [Mg_4Al-LDH] as an Efficient Catalyst for the α-Alkylation of Nitriles, Oxindoles and Other Carboxylic Acid Derivatives with Alcohols. *ChemSusChem* **2023**, *16*, e202300818.

14. Yang, X.; Wang, A.; Guo, J.; Guo, Y.; Guo, Y.; Wang, L.; Zhan, W. γ-Al_2O_3 Supported Silver Nanoparticle Applied in C_3H_8-SCR: Nanosphere and Nanoflake. *Catalysis Commun.* **2023**, *176*, 106634.

15. Wu, X.; Ding, G.; Yang, L.; Lu, W.; Li, W.; Zhang, Z.; Xie, X. Alkoxide-Catalyzed Hydrosilylation of Cyclic Imides to Isoquinolines via Tandem Reduction and Rearrangement. *Org. Lett.* **2018**, *20*, 5610-5613.

16. Nammalwar, B.; Muddala, N. P.; Watts, F. M.; Bunce, R. A. Efficient Conversion of Acids and Esters to Amides and Transamidation of Primary Amides Using OSU-6. *Tetrahedron* **2015**, *71*, 9101-9111.

17. Abdel-Aziz, A. A. M. Novel and Versatile Methodology for Synthesis of Cyclic Imides and Evaluation of their Cytotoxic, DNA Binding, Apoptotic Inducing Activities and Molecular Modeling Study. *Eur. J. Med. Chem.* **2007**, *42*, 614-626.

18. Lindsley, C. W.; Engers, D. W.; Engers, J. L.; Bungard, J. D.; Li, J.; Long, M. F.; Han, C.; Dodd, C. J. Positive Allosteric Modulators of the Muscarinic Acetylcholine Receptor M1. Patent WO2024086570, 2024.

19. Goldfarb, D. S. Method Using Lifespan-Altering Compounds for Altering the Lifespan of Eukaryotic Organisms, and Screening for Such Compounds. Patent US20090163545, 2009.

20. Sondhi, S. M.; Rani, R. A Convenient, Solvent Free and High Yielding Synthesis of Bicyclo- Heterocyclic Compounds. *Lett. Org. Chem.* **2008**, *5*, 51-54.

21. Hitchings, G. J.; Vernon, J. M. Regioselective Formation of Hydroxy Lactams from Pyridine-2,3-Dicarboximides and Their Cyclodehydration to Pyrido[2′,3′:3,4]Pyrrolo-Fused Heterocyclic Systems. *J. Chem. Soc., Perkin Trans. 1* **1990**, 1757-1763.

22. Kajita, Y.; Matsubara, S.; Kurahashi, T. Nickel-Catalyzed Decarbonylative Addition of Phthalimides to Alkynes. *J. Am. Chem. Soc* **2008**, *130*, 6058-6059.

23. Kon'shin, M. E.; Syropyatov, B. Y.; Vakhrin, M. I.; Neifel'd, P. G.; Feshin, V. P.; Shurov, S. N.; Odegova, T. F. Synthesis and Pharmacological Activity of 3-Carboxypicolinic Acid Amides. *Pharm. Chem. J.* **2010**, *44*, 476-479.

24. Blanco, M. M.; Levin, G. J.; Schapira, C. B.; Perillo, I. A. Improved Synthesis of N-Substituted 2,3-Pyridinedicarboximides with Microwave Irradiation. *Heterocycles* **2002**, *57*, 1881-1890.

25. Ding, G.; Li, C.; Shen, Y.; Lu, B.; Zhang, Z.; Xie, X. Potassium Hydroxide-Catalyzed Chemoselective Reduction of Cyclic Imides with Hydrosilanes: Synthesis of ω-Hydroxylactams and Lactams. *Adv. Synth. Catal.* **2016**, *358*, 1241-1250.

26. Unverferth, K.; Arnold, T.; Lankau, H.-j.; Rostock, A.; Tober, C.; Dost, R.; Rundfeldt, C.; Gasparic, A. Preparation of 6,7-Dihydro-Pyrrolo[3,4-*b*]Pyridin-5-Ones as Anticonvulsants. Patent WO2002018381, 2002.

27. Sugimoto, H.; Tsuchiya, Y.; Higurashi, K.; Karibe, N.; Iimura, Y.; Sasaki, A.; Yamanashi, Y.; Ogura, H.; Araki, S. Preparation of 1-Benzyl-4-(Substituted Alkyl)Piperidines and Analogs as Acetylcholinesterase Inhibitors. Patent EP296560, 1988.

28. Galli, U.; Mesenzani, O.; Coppo, C.; Sorba, G.; Canonico, P. L.; Tron, G. C.; Genazzani, A. A. Identification of a Sirtuin 3 Inhibitor that Displays Selectivity over Sirtuin 1 and 2. *Eur. J. Med. Chem.* **2012**, *55*, 58-66.

8. NMR SPECTRA OF THE ISOLATED ORGANIC COMPOUNDS

^{1}H NMR (Compound **1**)

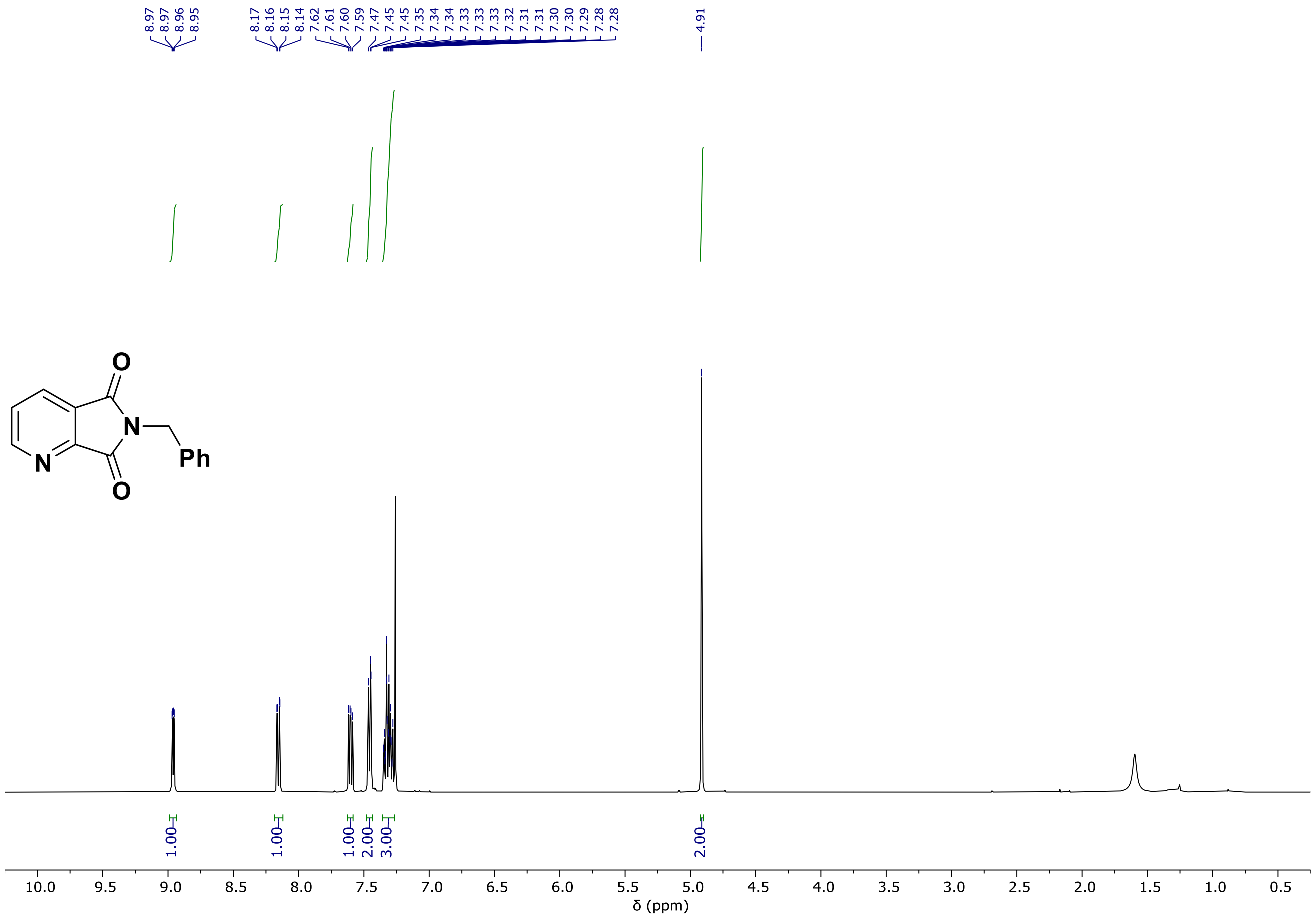

^{13}C NMR (Compound **1**)

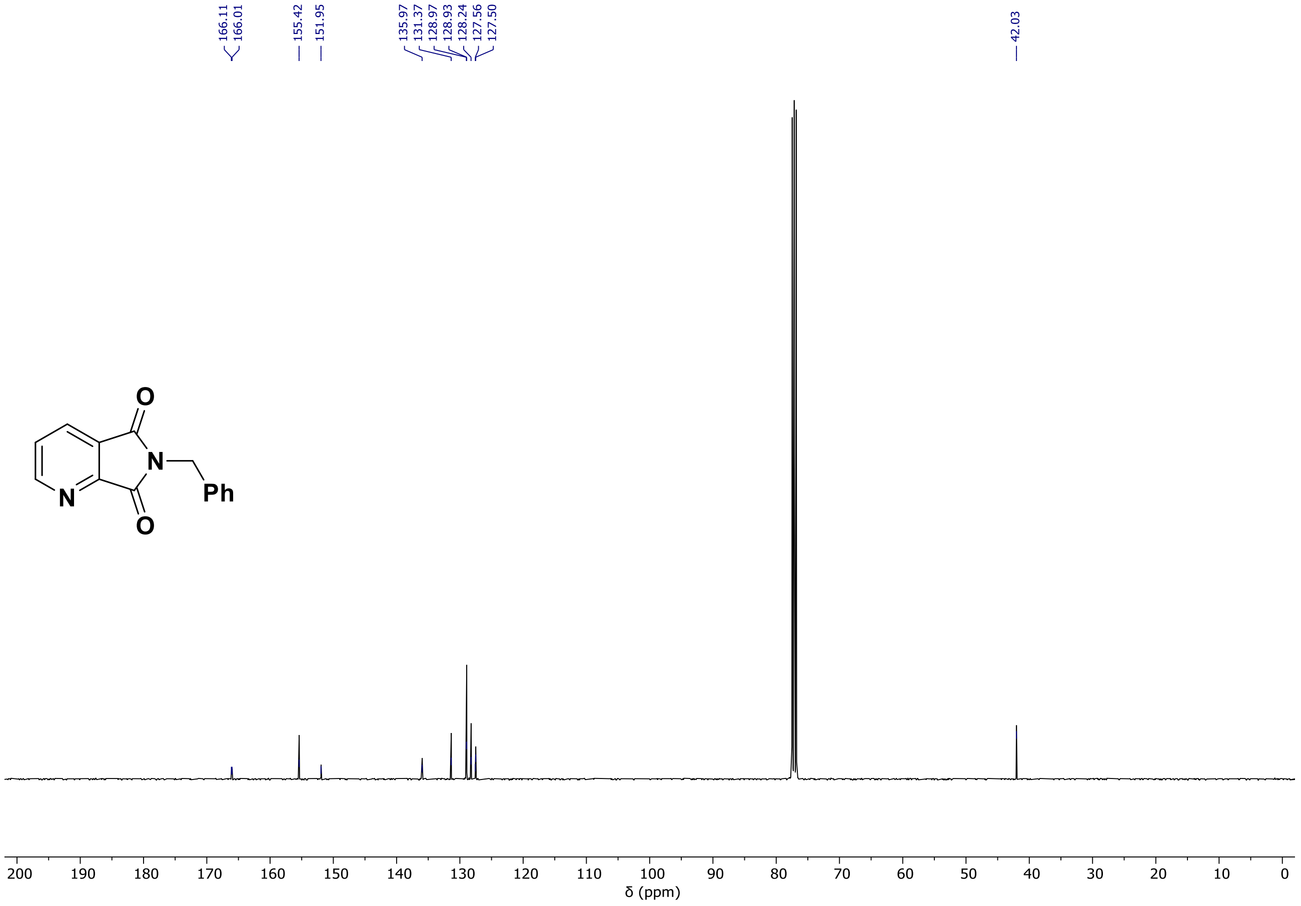

^{1}H NMR (Compound **S1**)

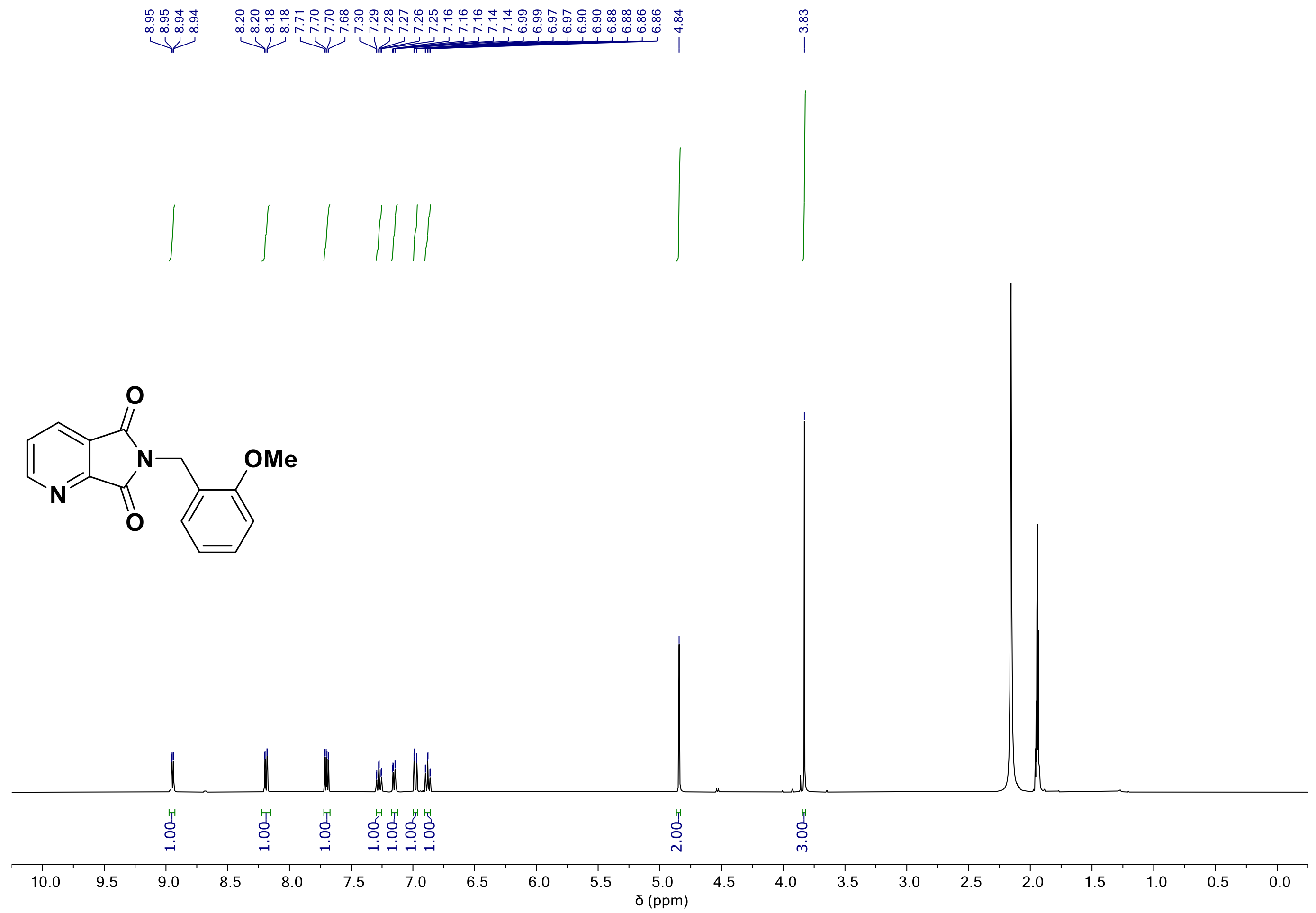

^{13}C NMR (Compound **S1**)

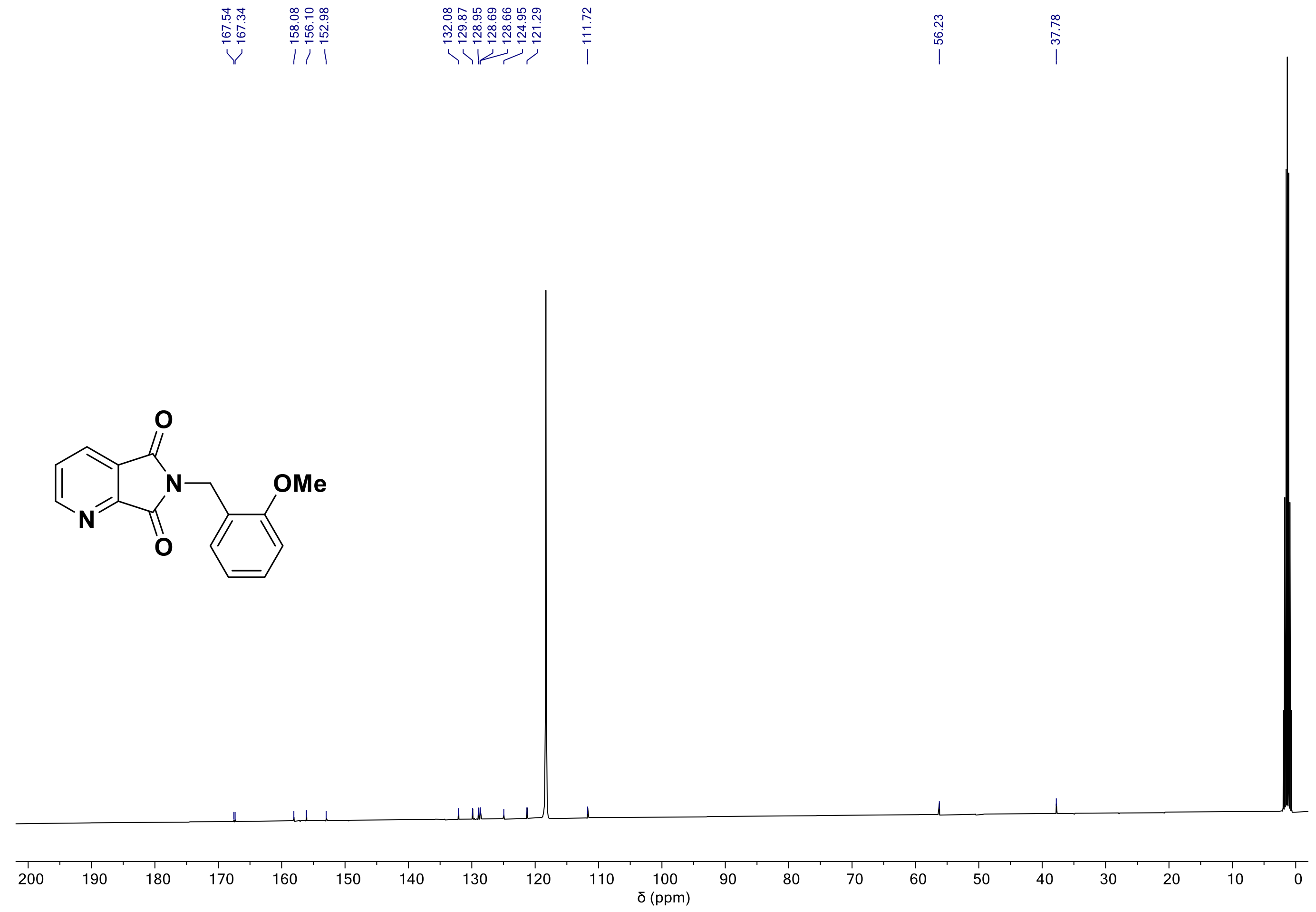

^{1}H NMR (Compound **S2**)

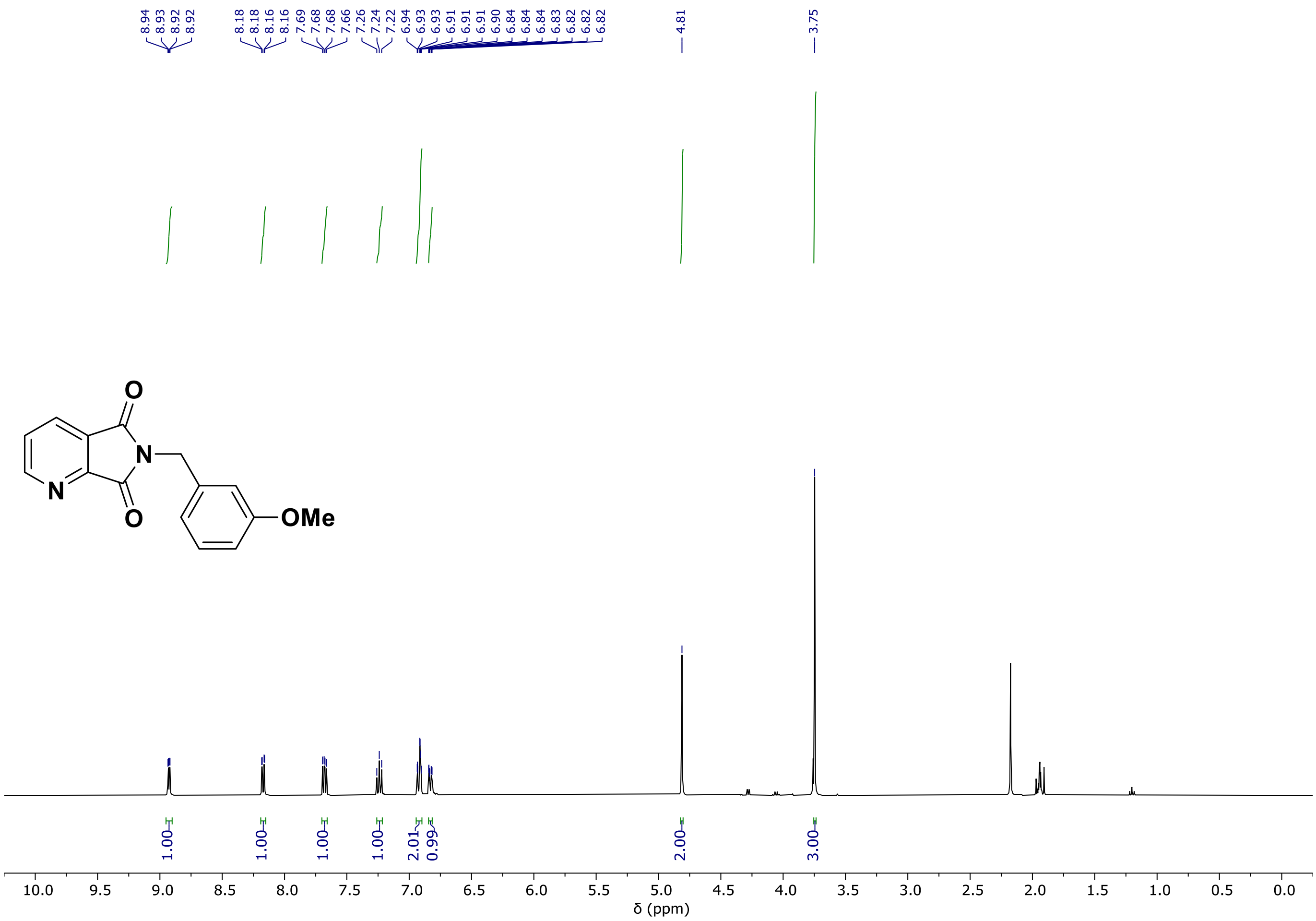

^{13}C NMR (Compound **S2**)

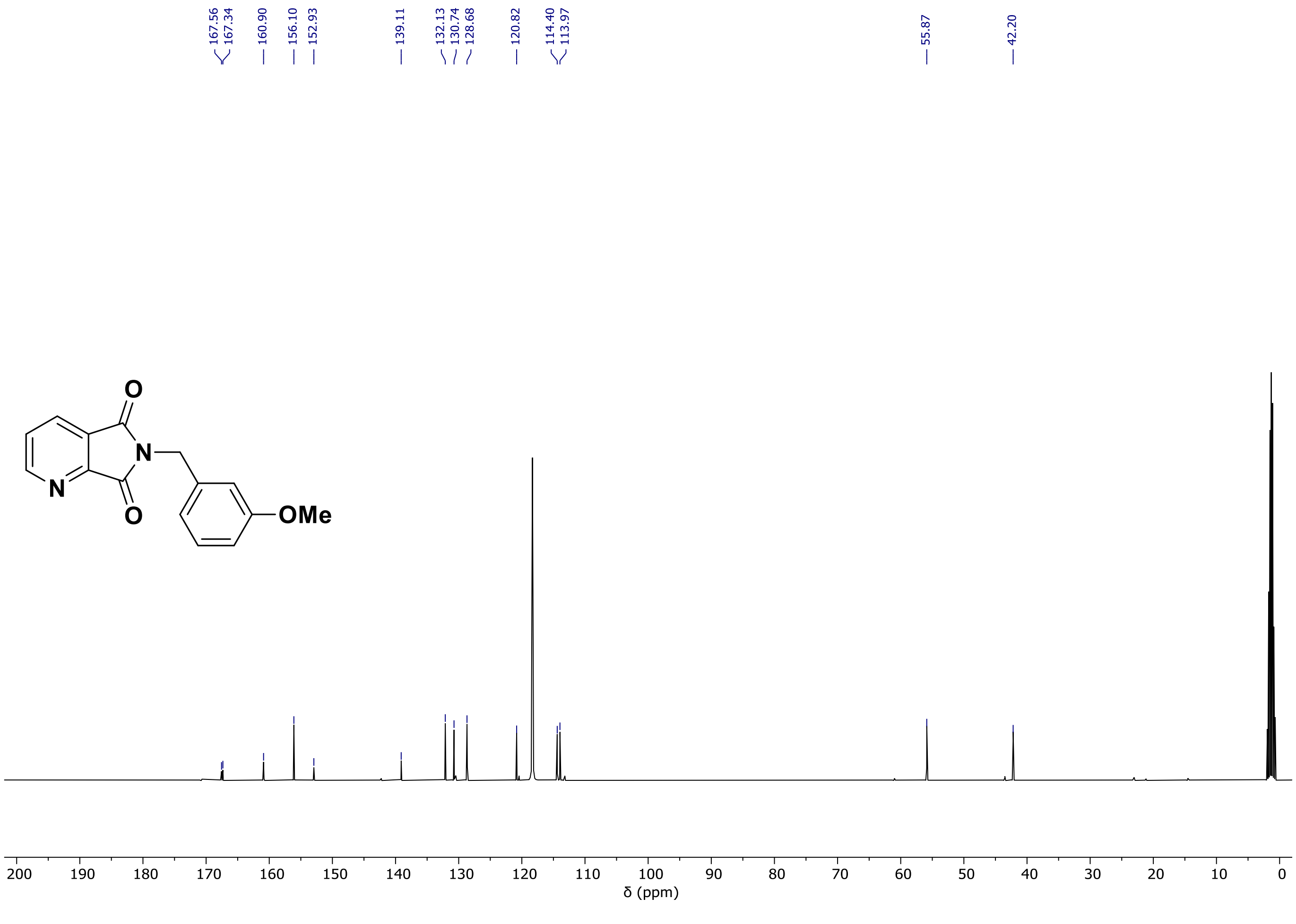

^{1}H NMR (Compound **S3**)

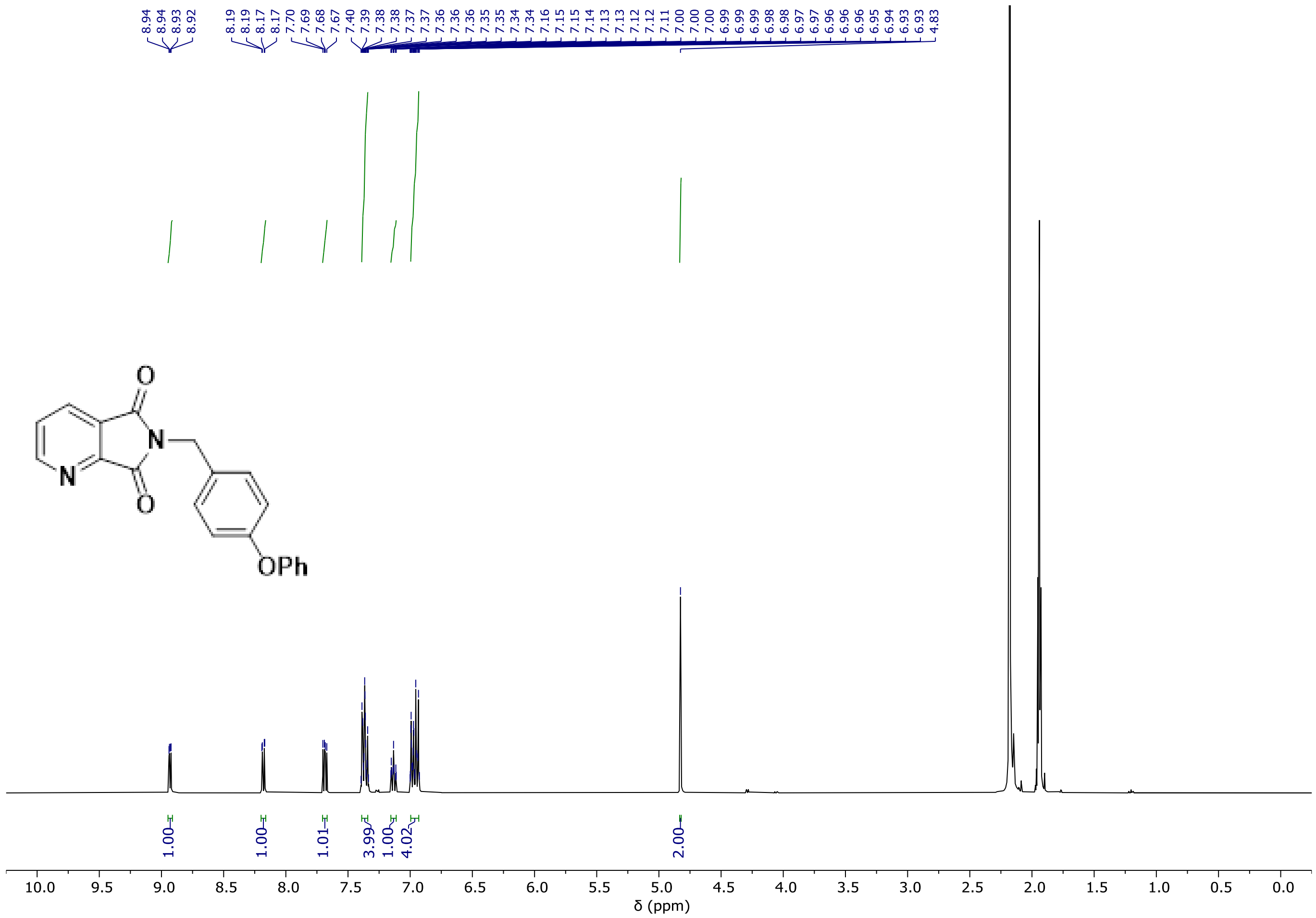

^{13}C NMR (Compound **S3**)

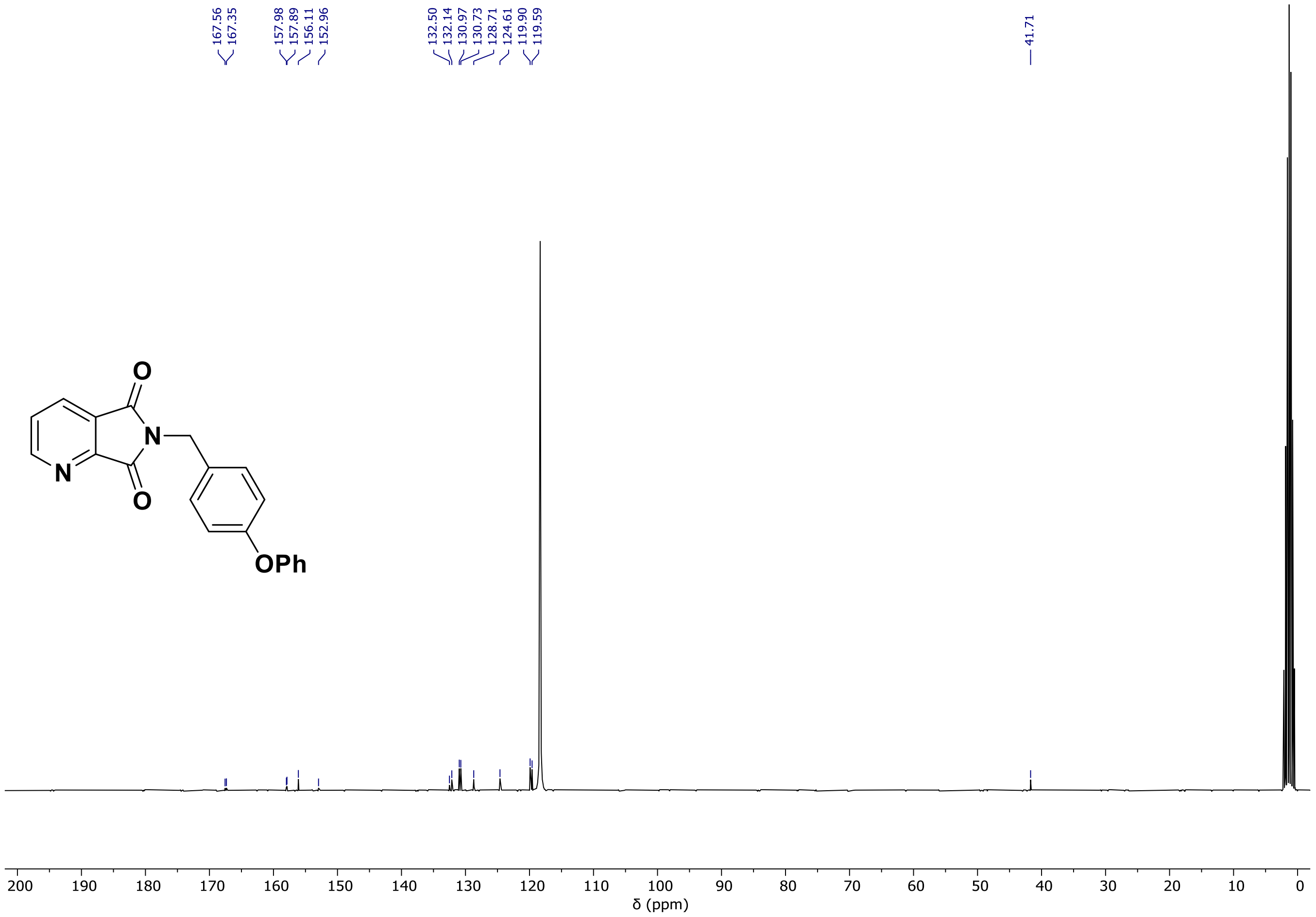

^{1}H NMR (Compound **S4**)

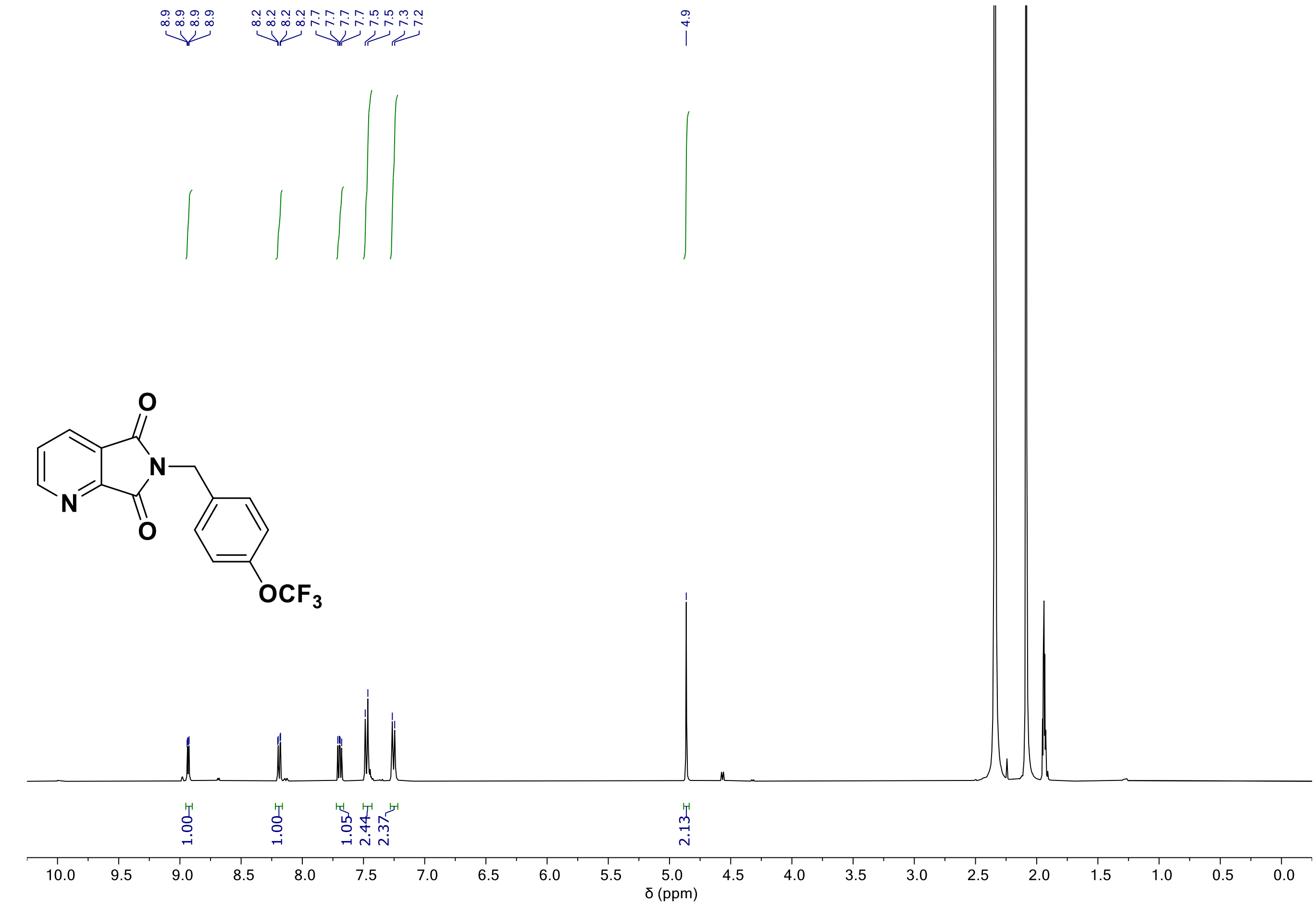

^{13}C NMR (Compound **S4**)

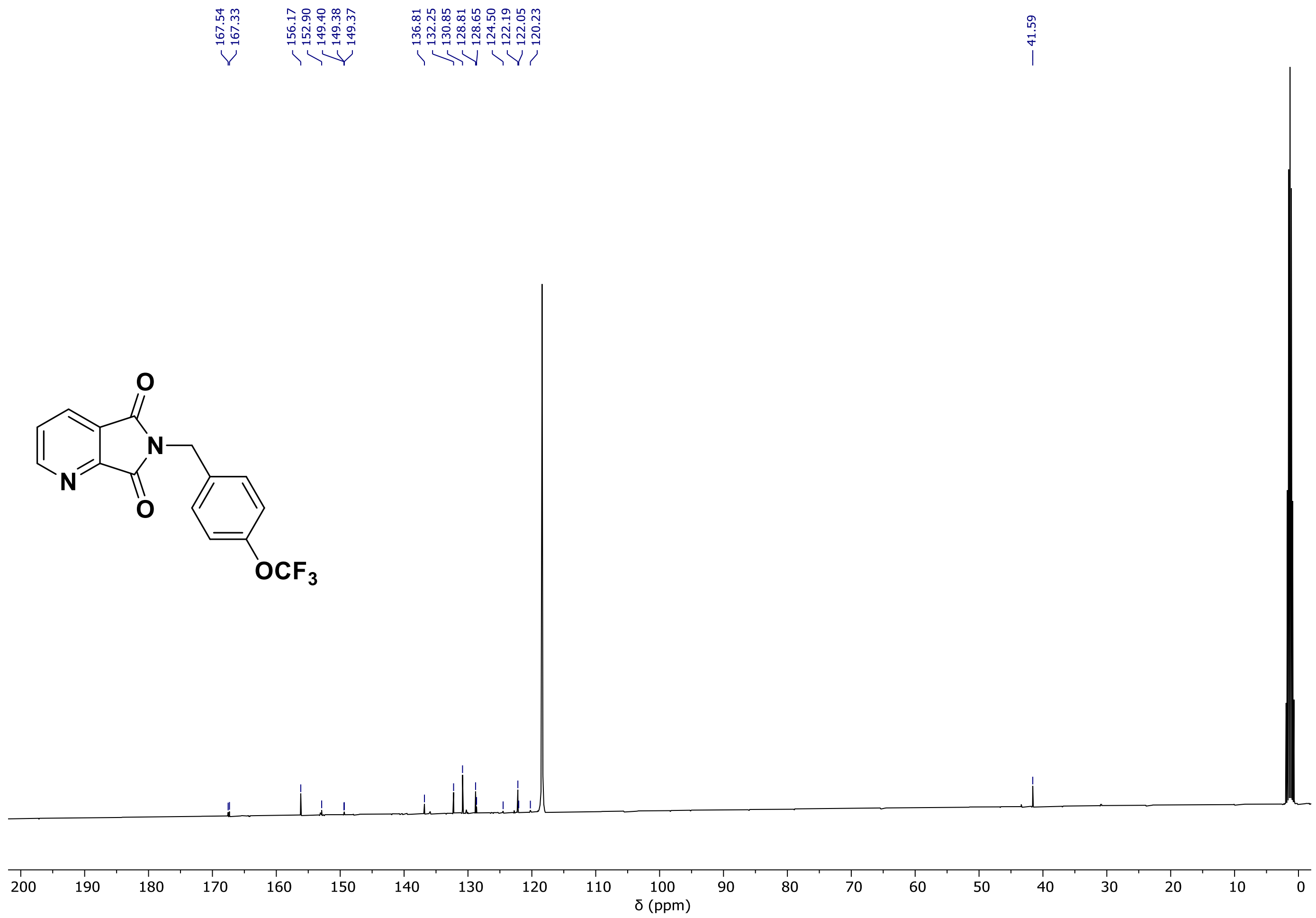

^{19}F NMR (Compound **S4**)

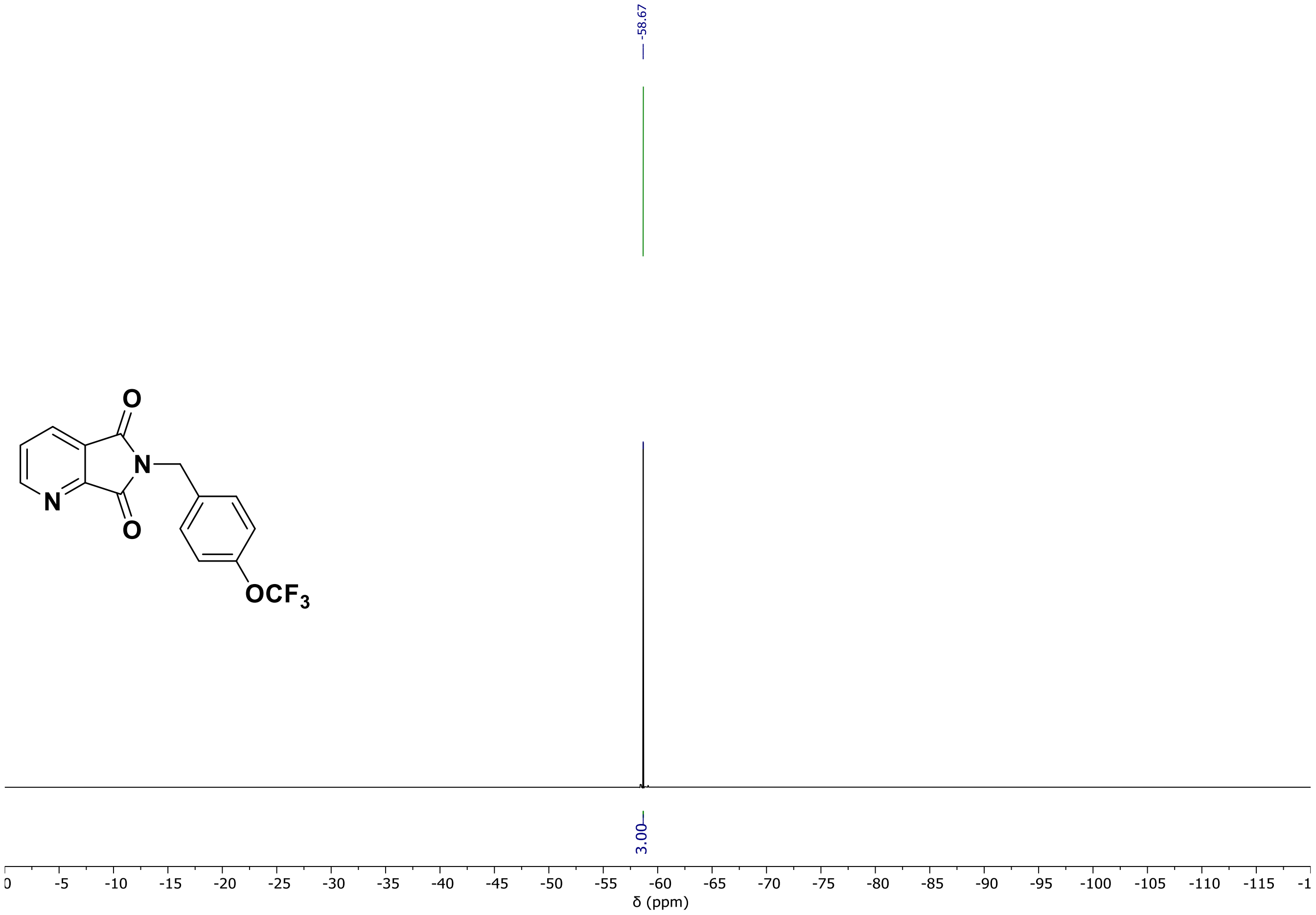

^{1}H NMR (Compound **S5**)

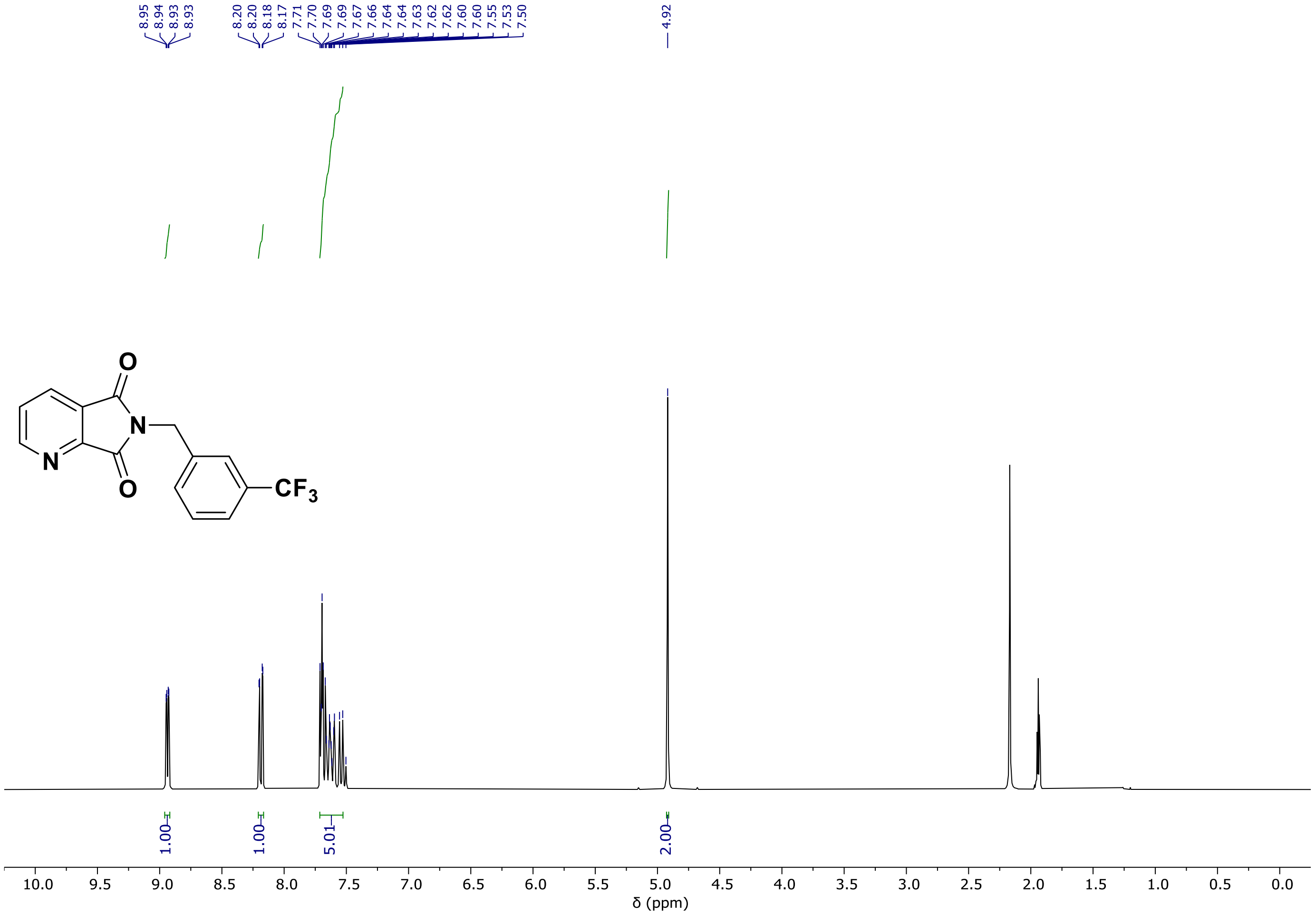

^{13}C NMR (Compound **S5**)

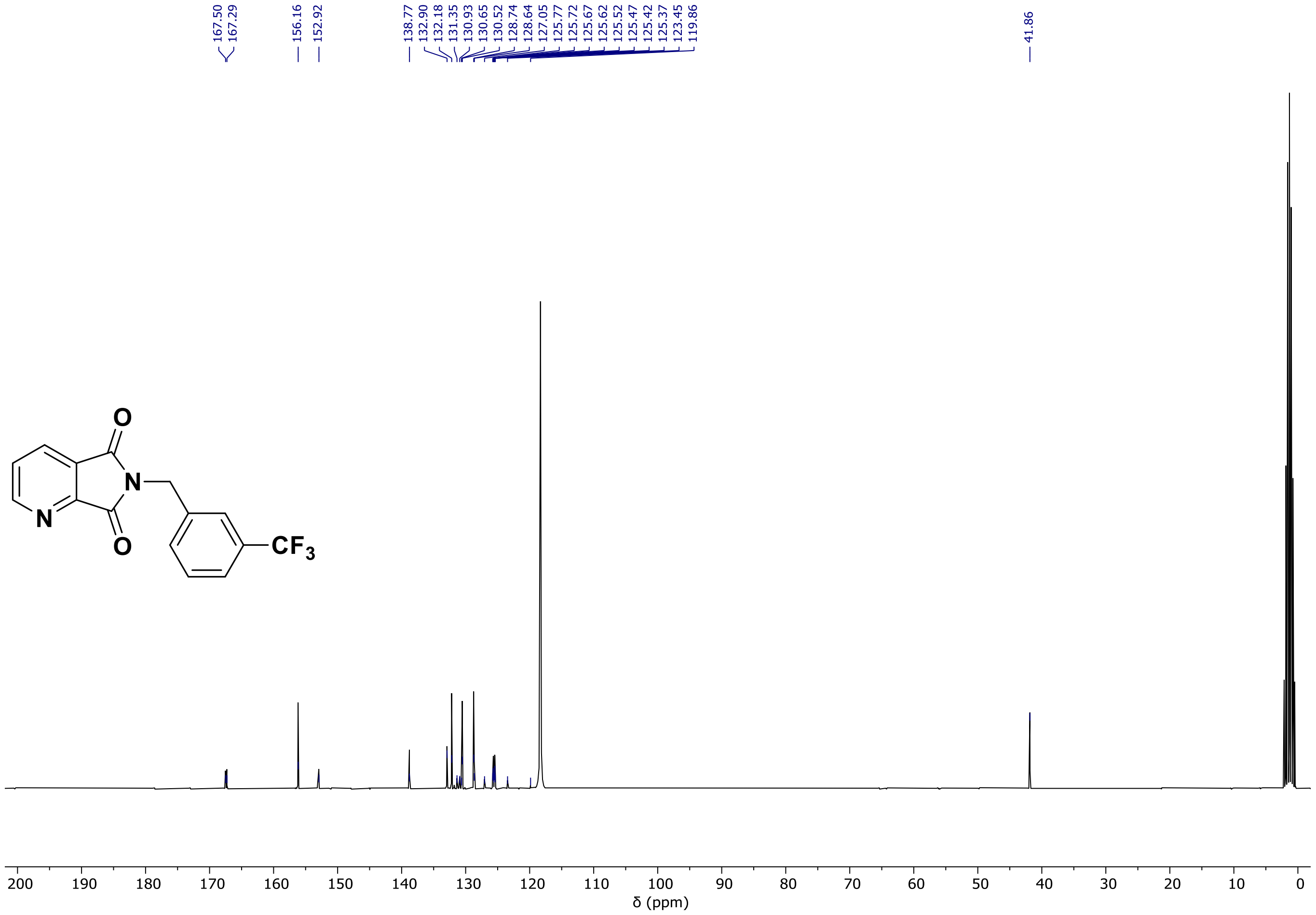

^{19}F NMR (Compound **S5**)

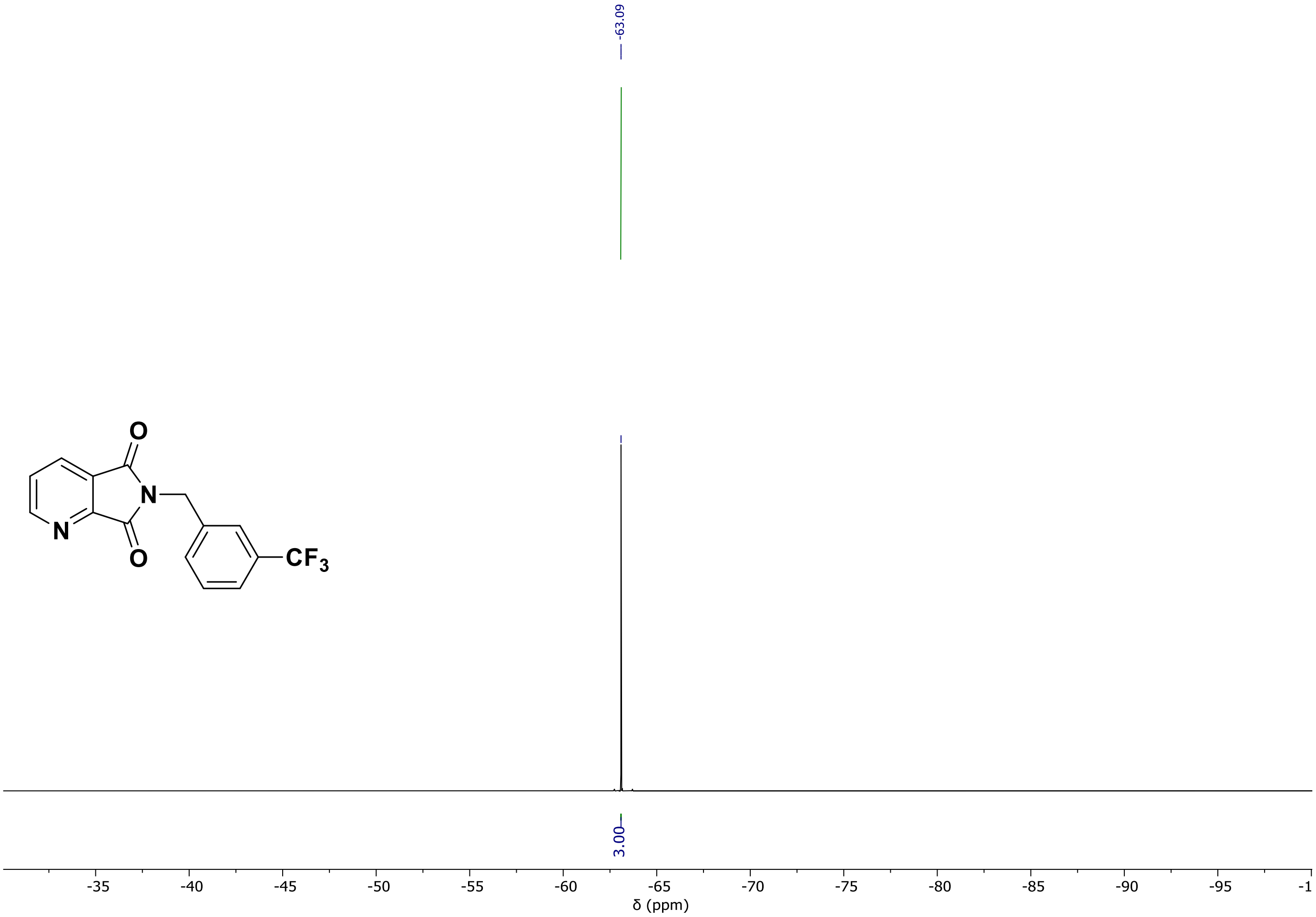

^{1}H NMR (Compound S6)

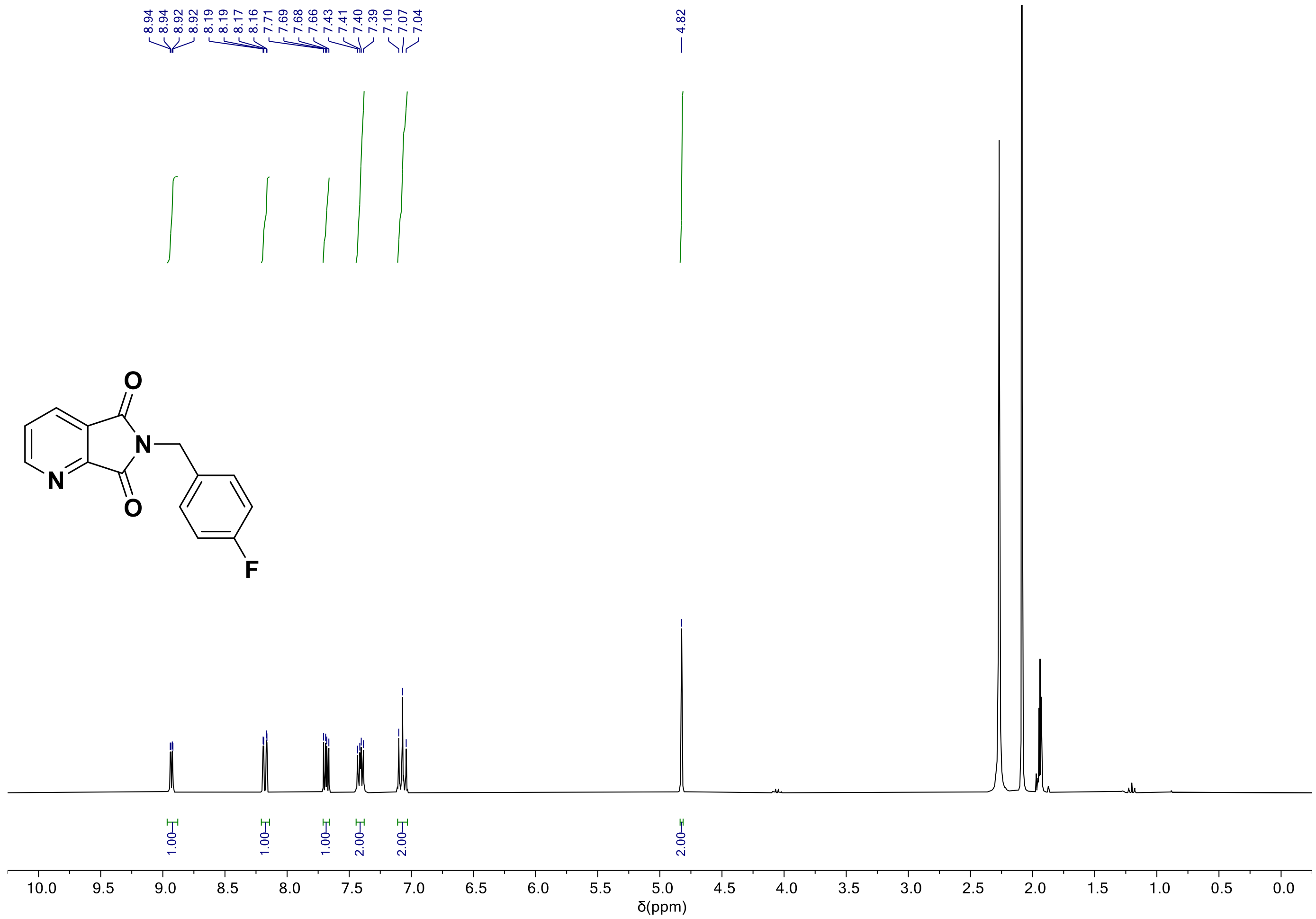

^{13}C NMR (Compound S6)

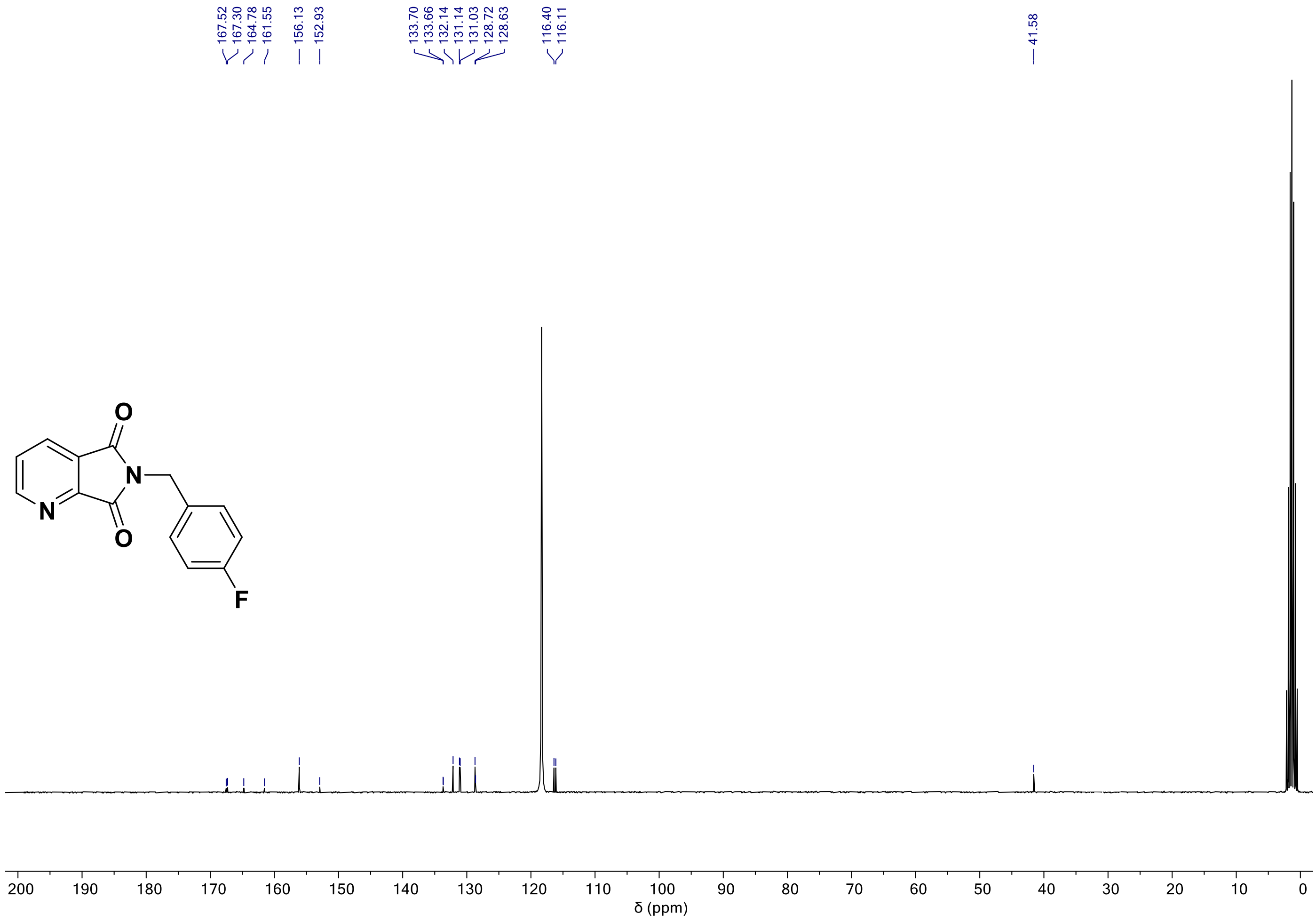

^{19}F NMR (Compound **S6**)

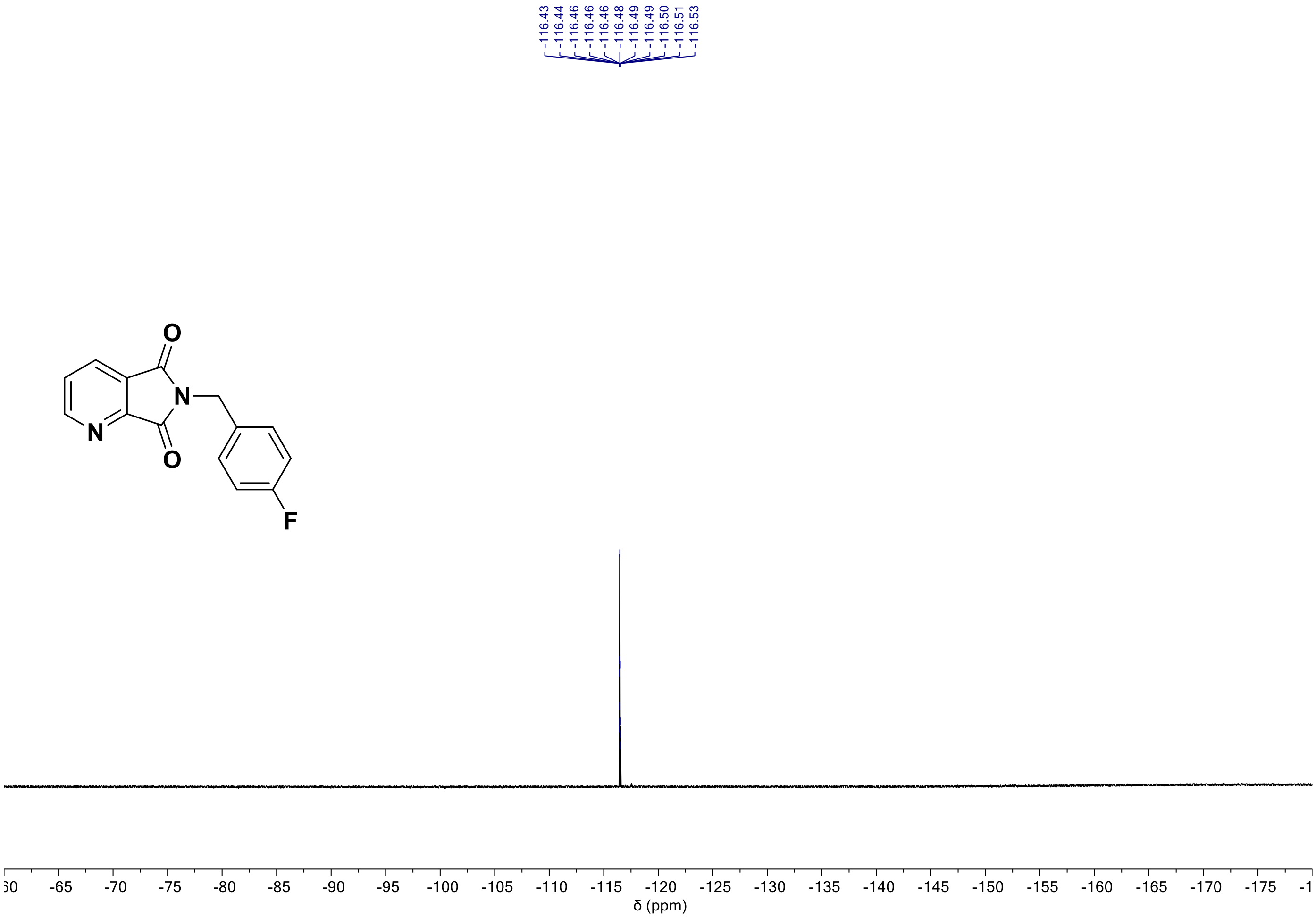

^{1}H NMR (Compound **S7**)

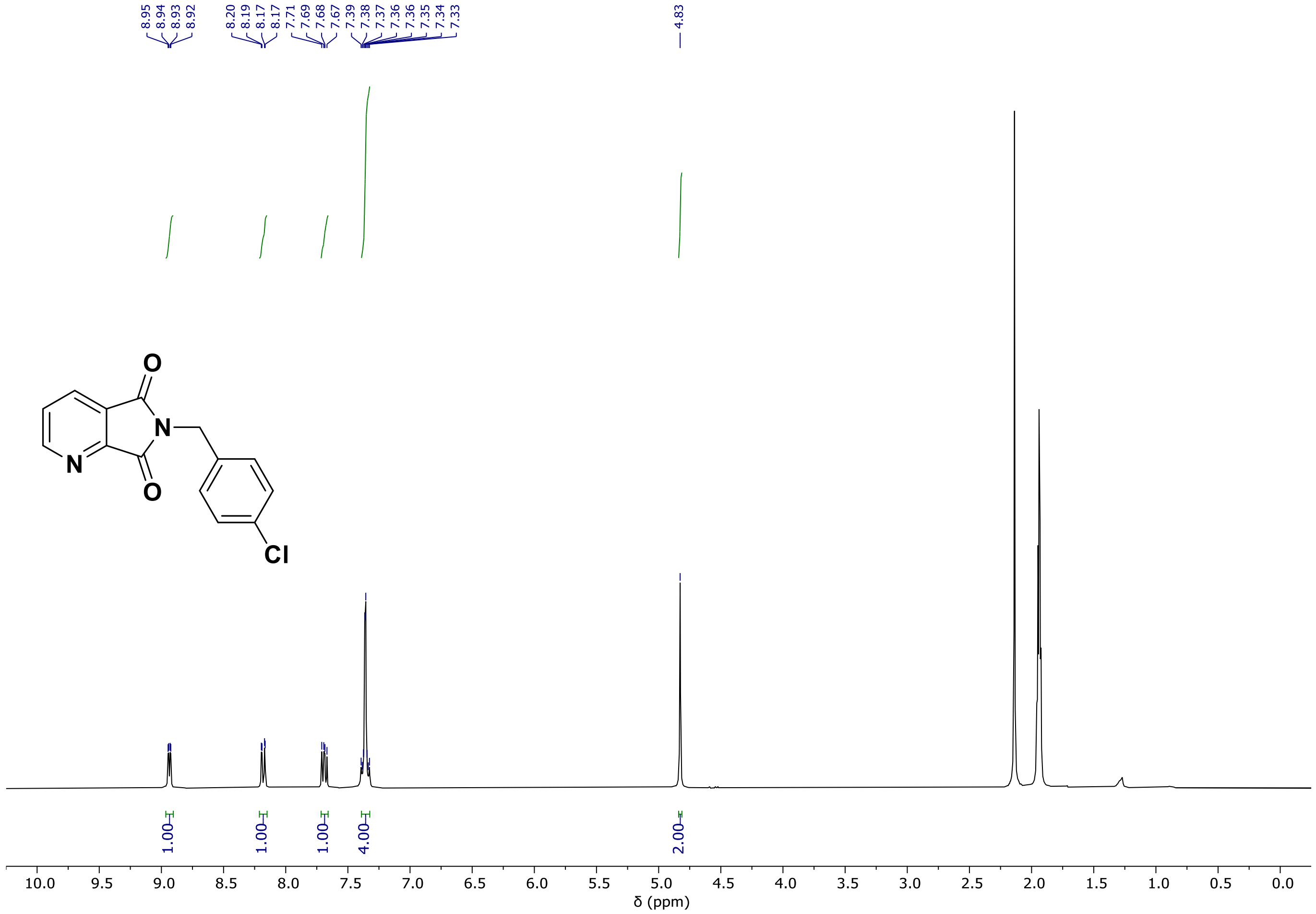

^{13}C NMR (Compound **S7**)

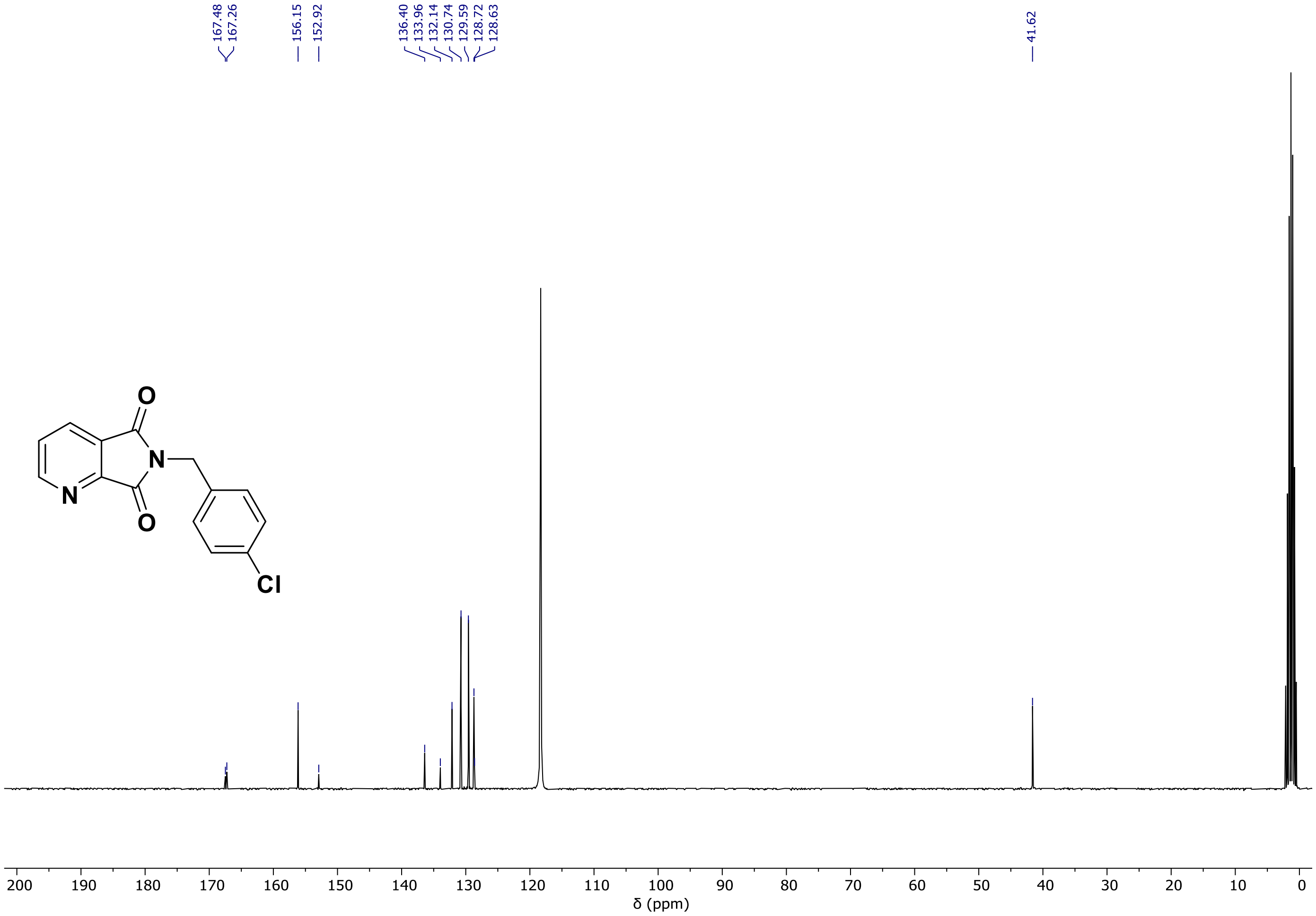

^{1}**H NMR** (Compound **S8**)

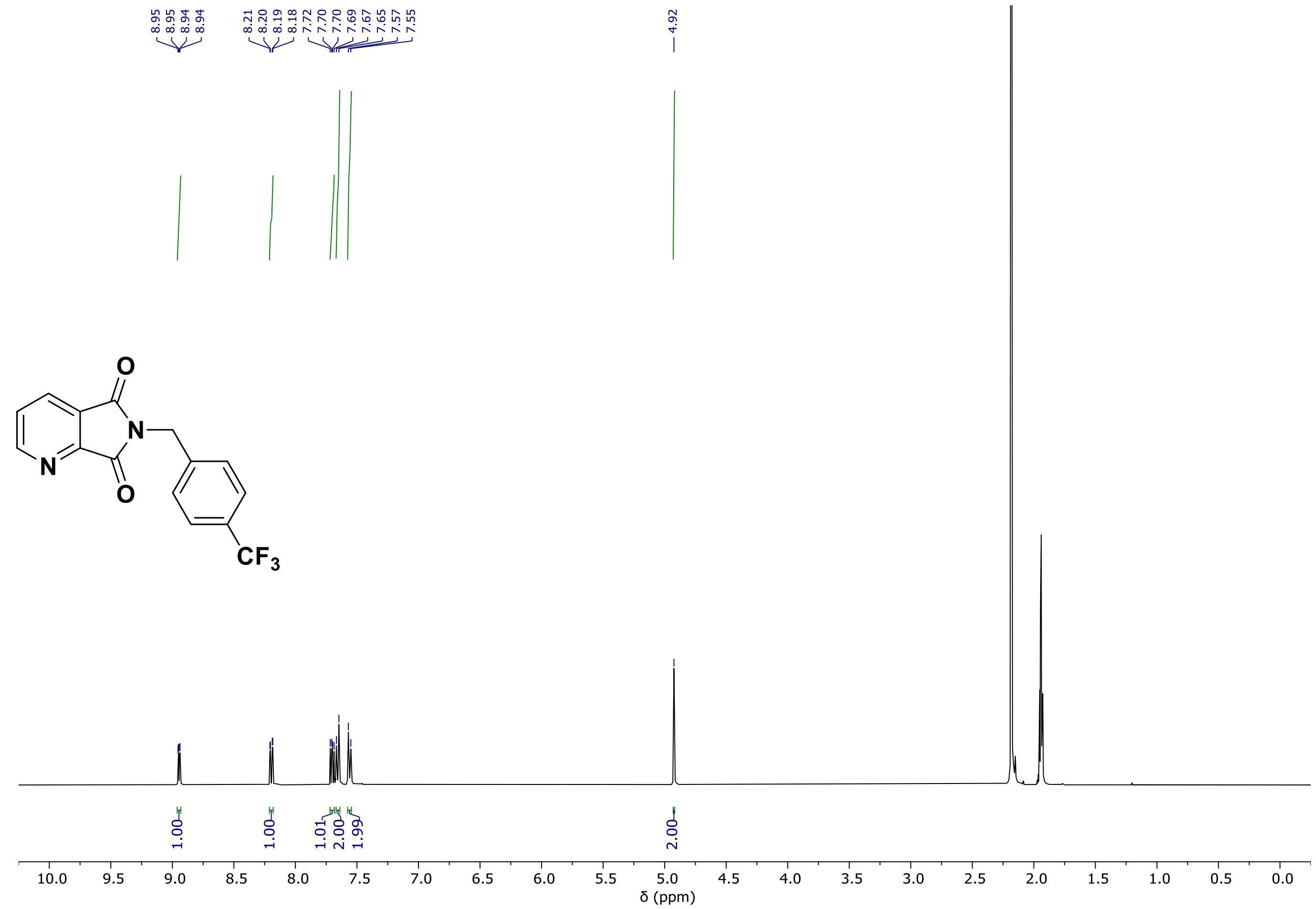

^{13}**C NMR** (Compound **S8**)

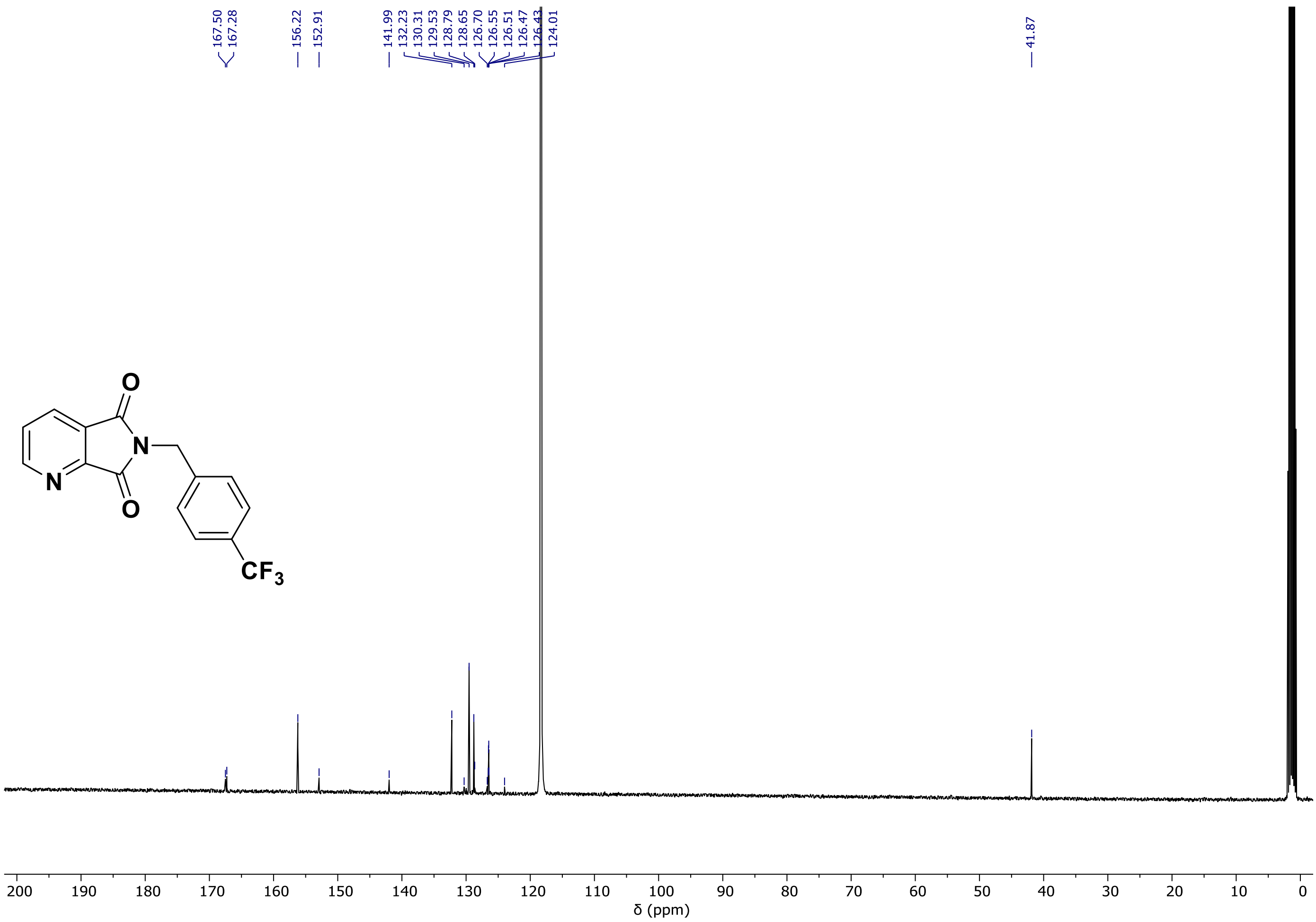

^{19}F NMR (Compound **S8**)

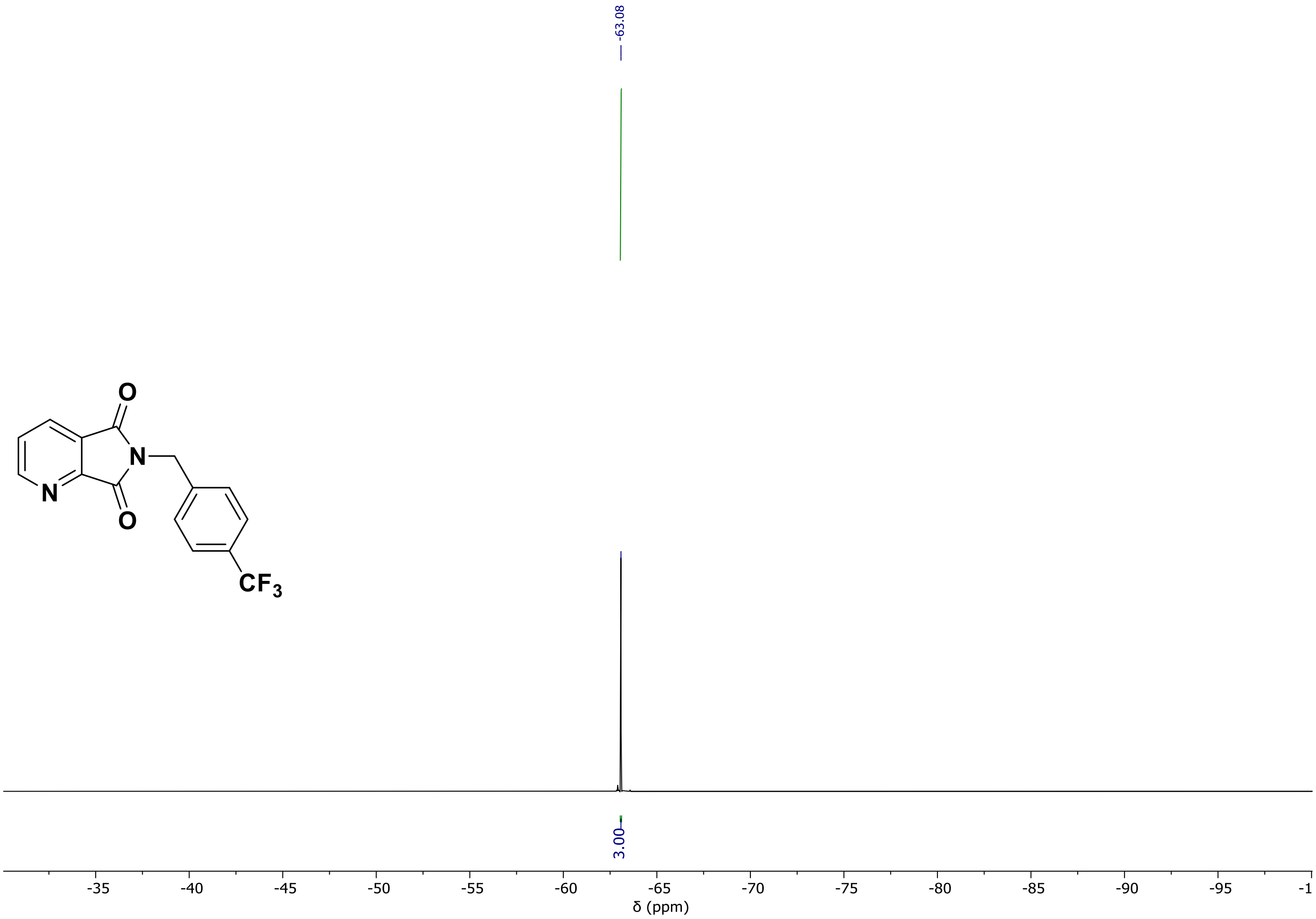

^{1}H NMR (Compound **S9**)

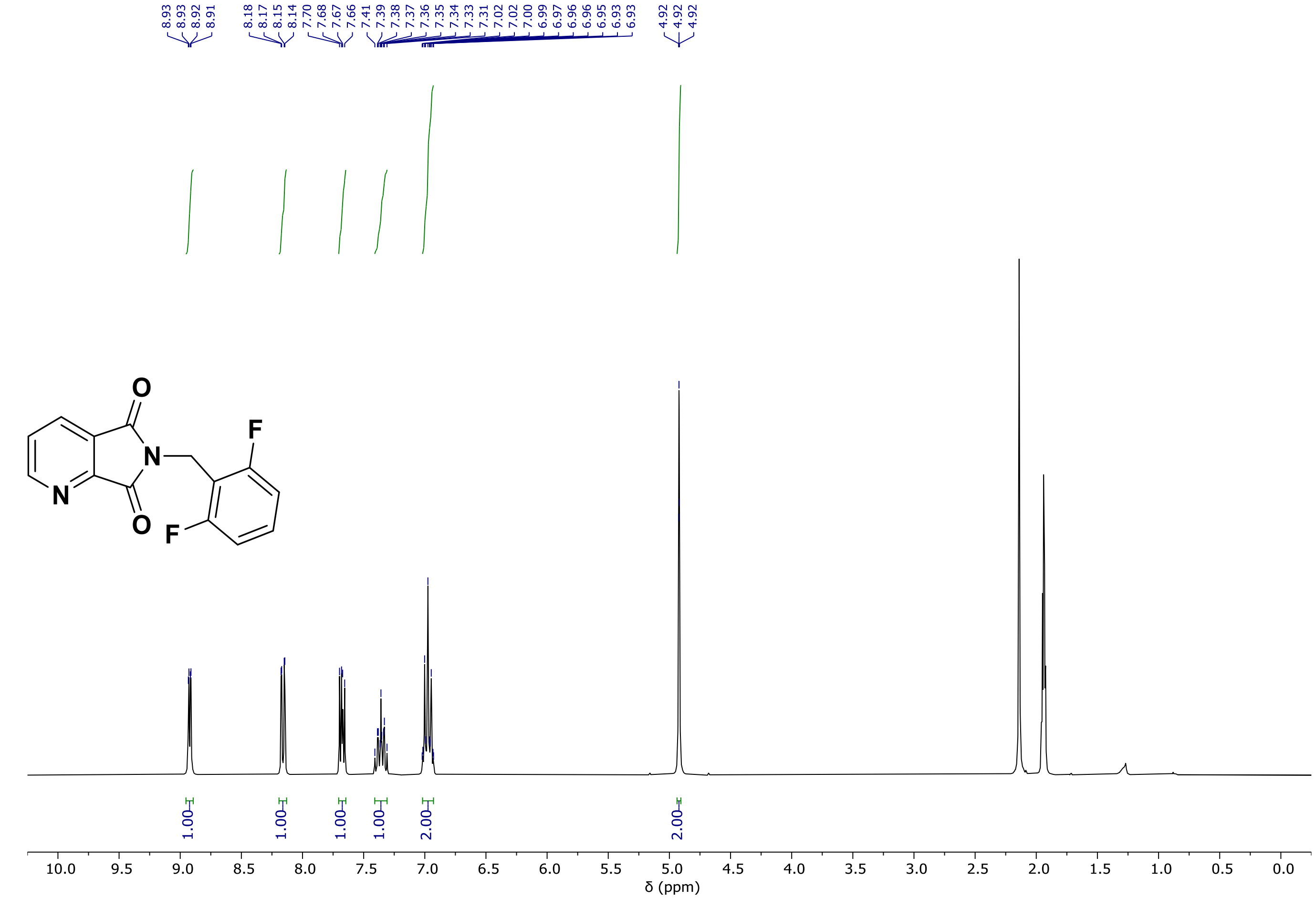

^{13}C NMR (Compound **S9**)

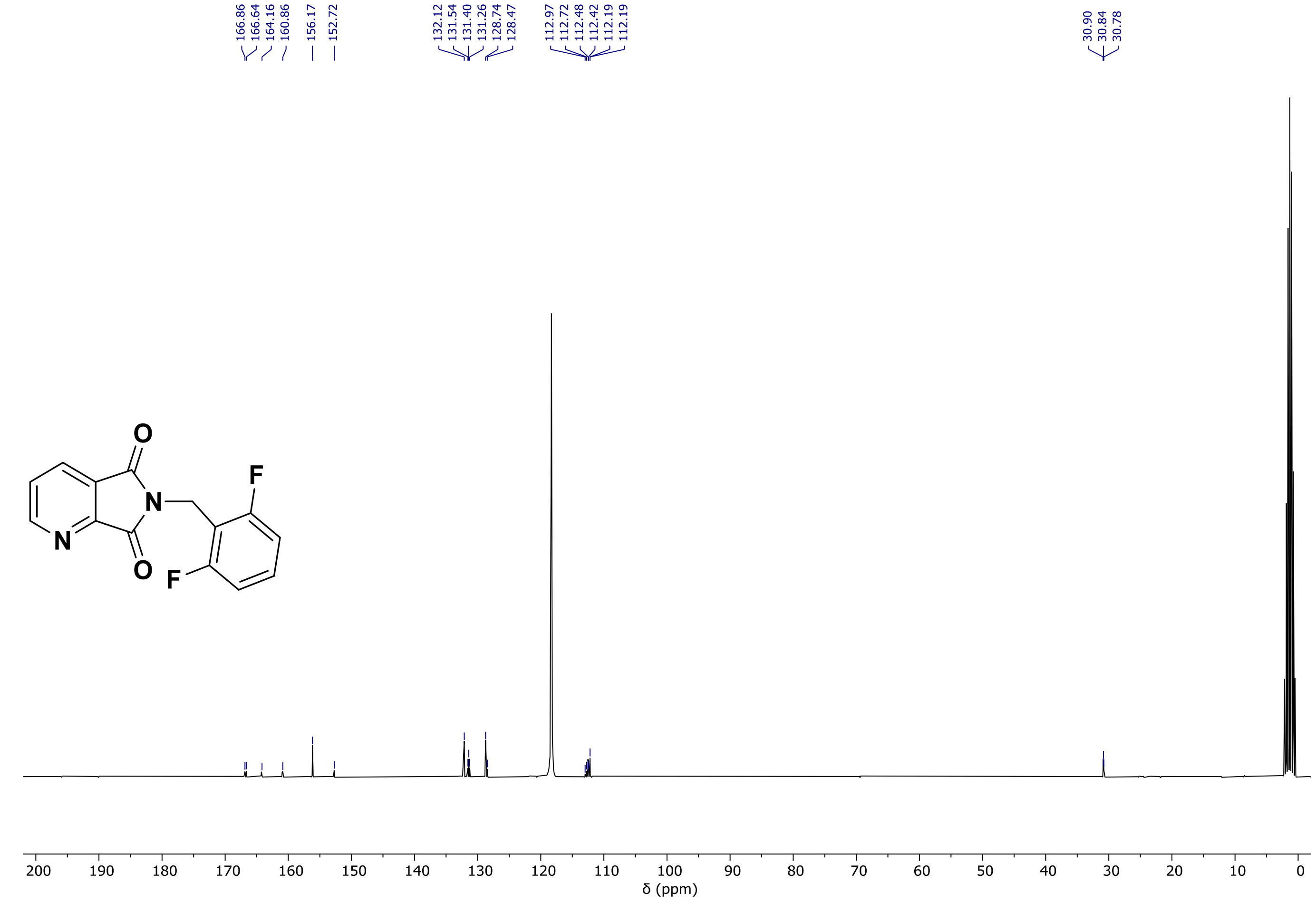

^{19}F NMR (Compound **S9**)

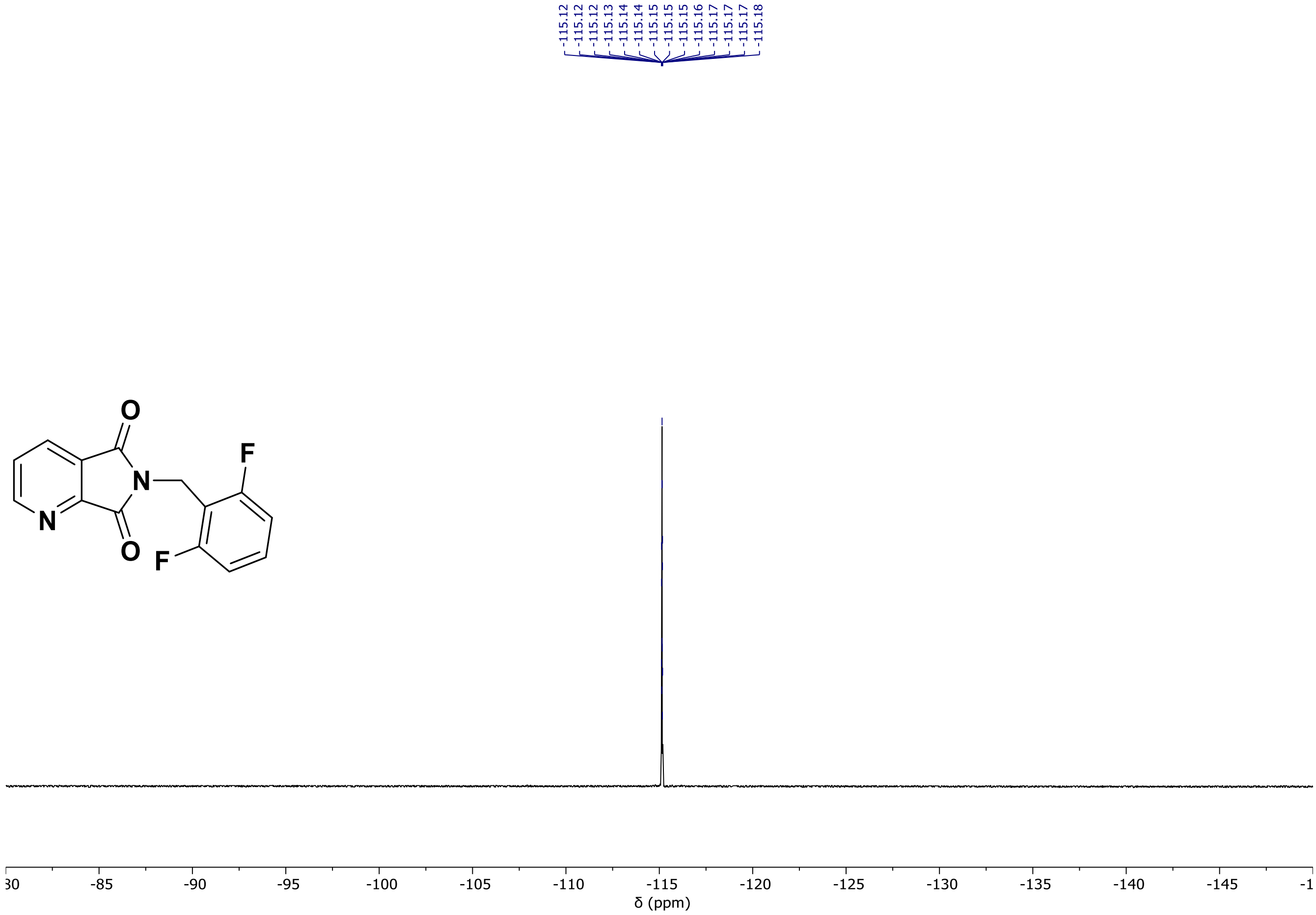

^{1}H NMR (Compound **S10**)

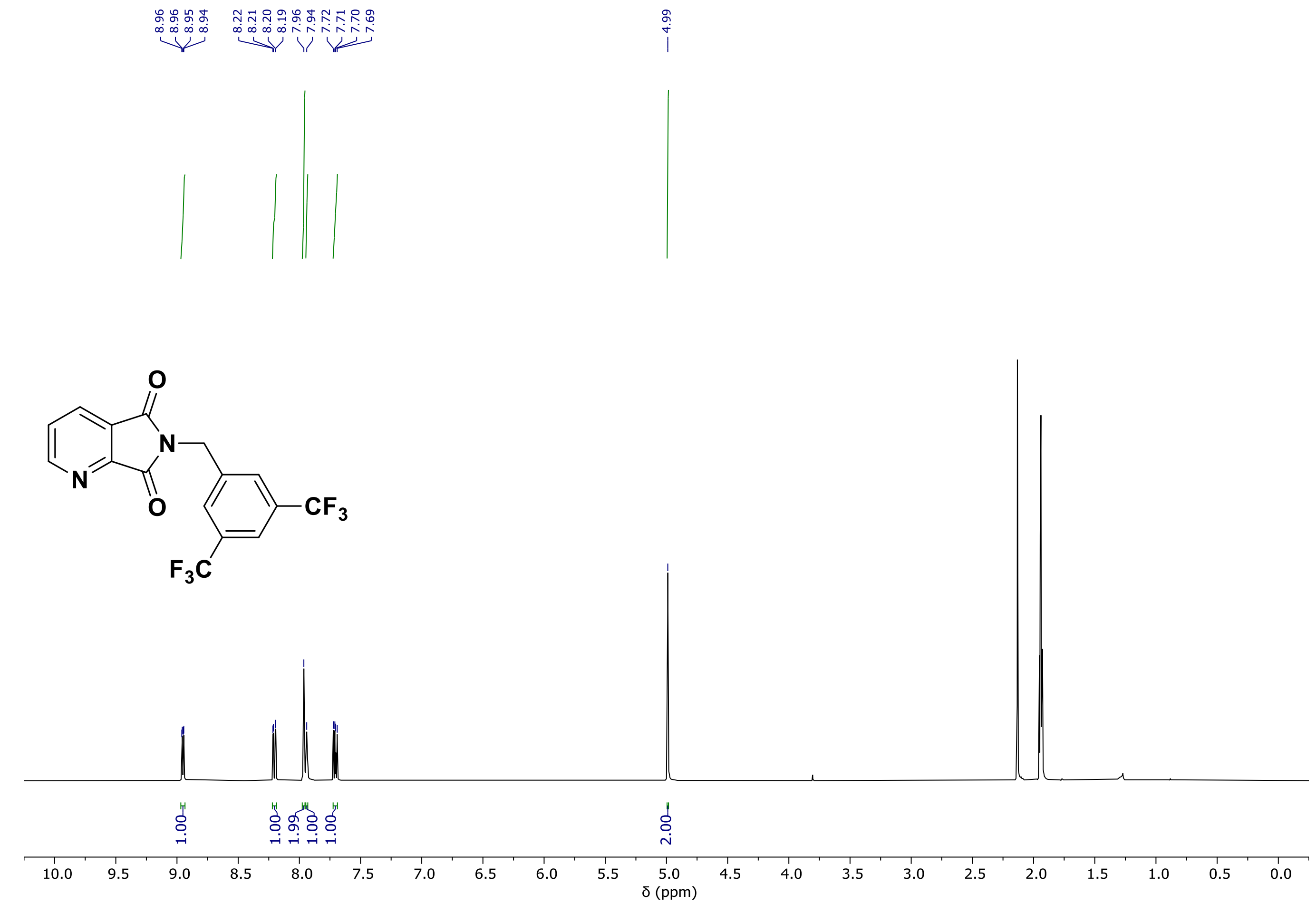

^{13}C NMR (Compound **S10**)

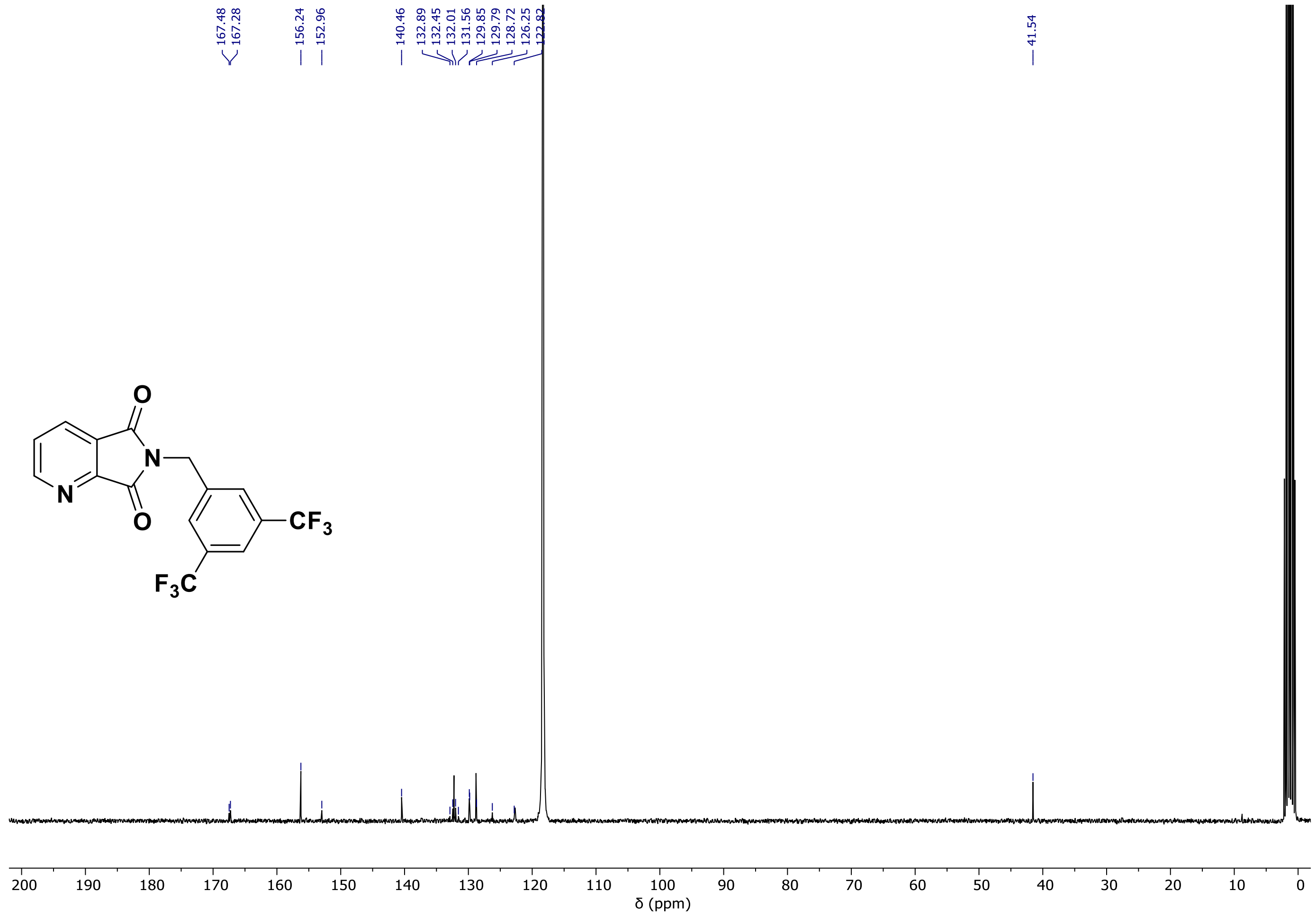

^{19}F NMR (Compound **S10**)

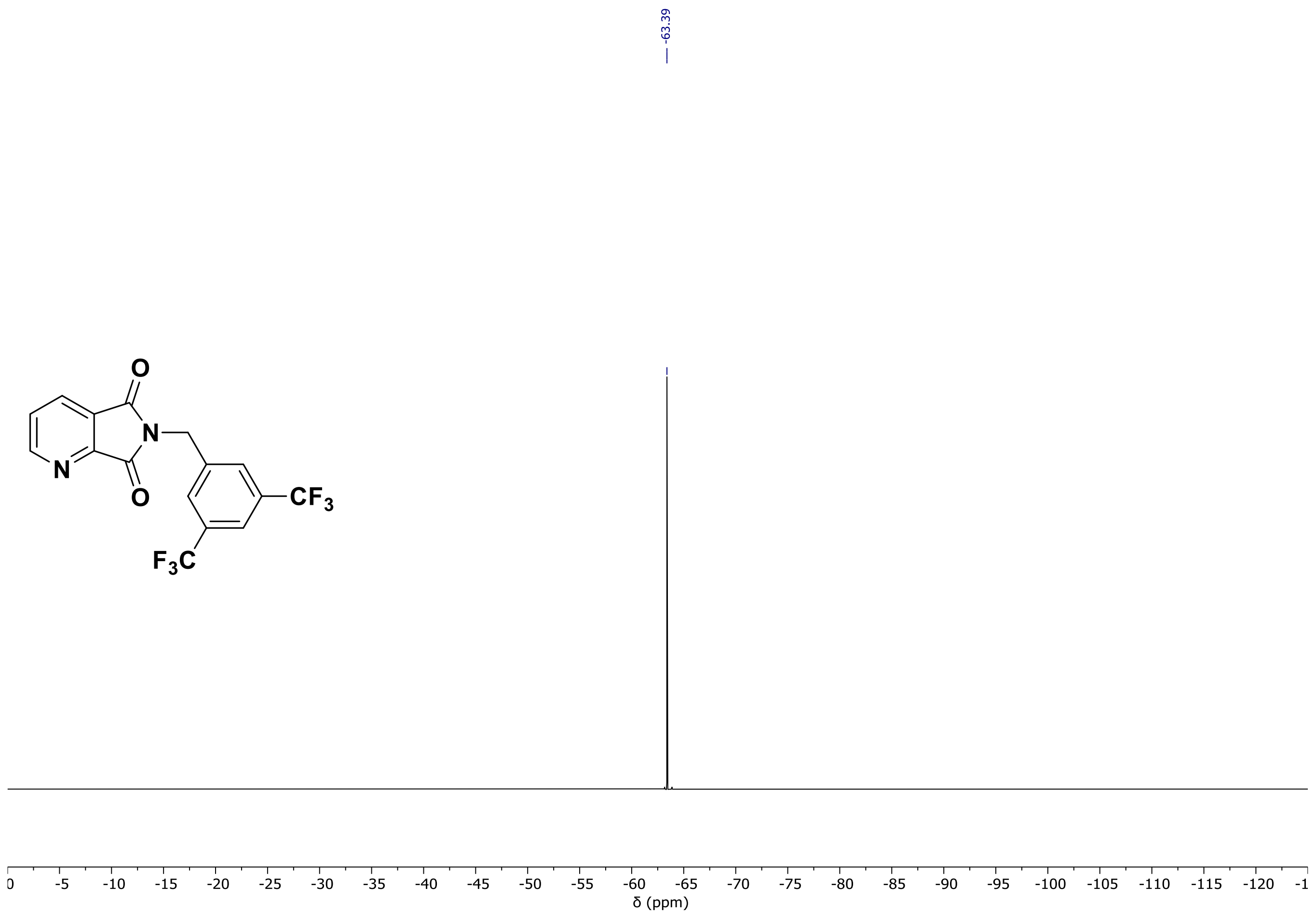

^{1}H NMR (Compound **S11**)

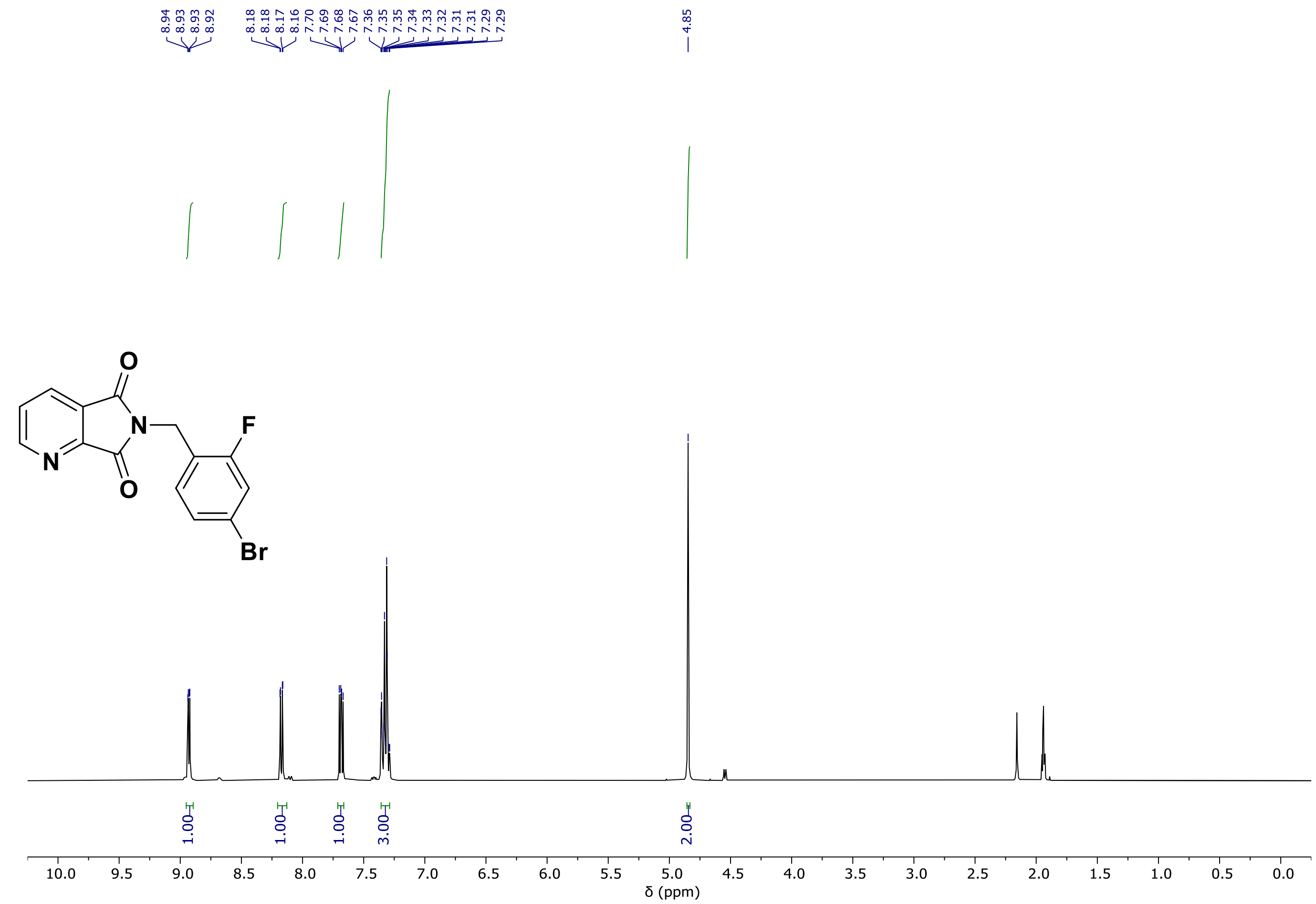

^{13}C NMR (Compound **S11**)

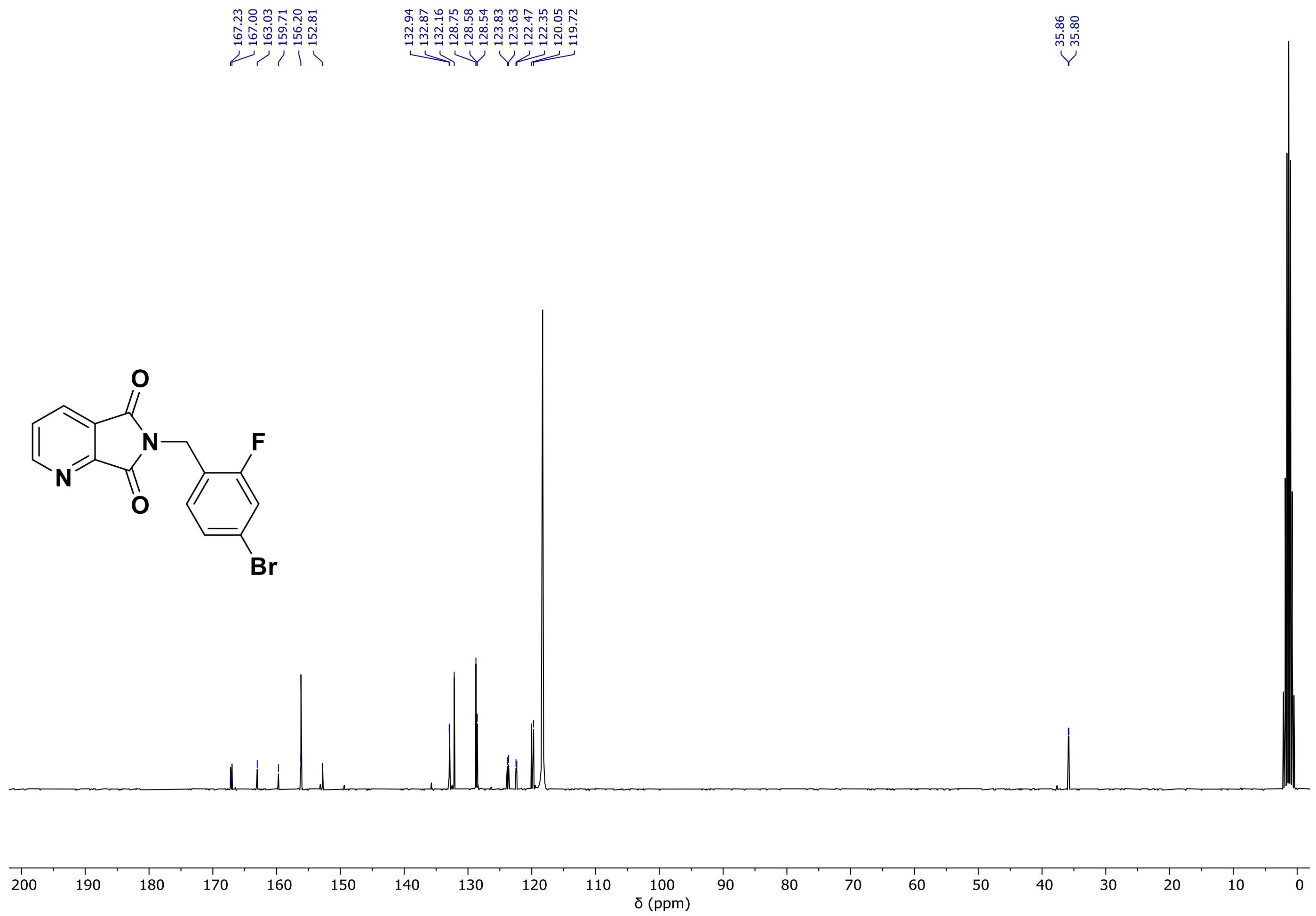

^{19}F NMR (Compound **S11**)

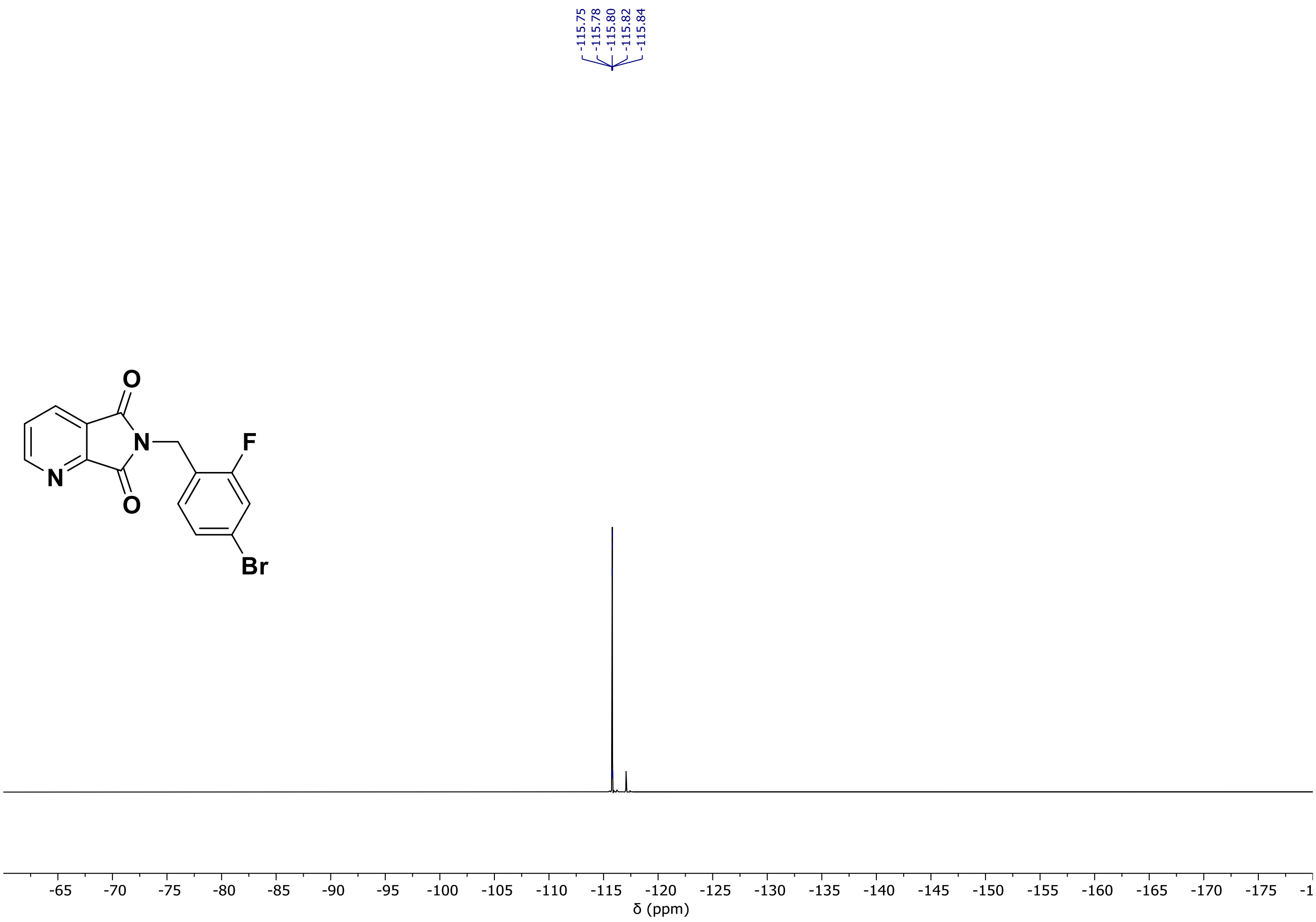

^{1}H NMR (Compound **S12**)

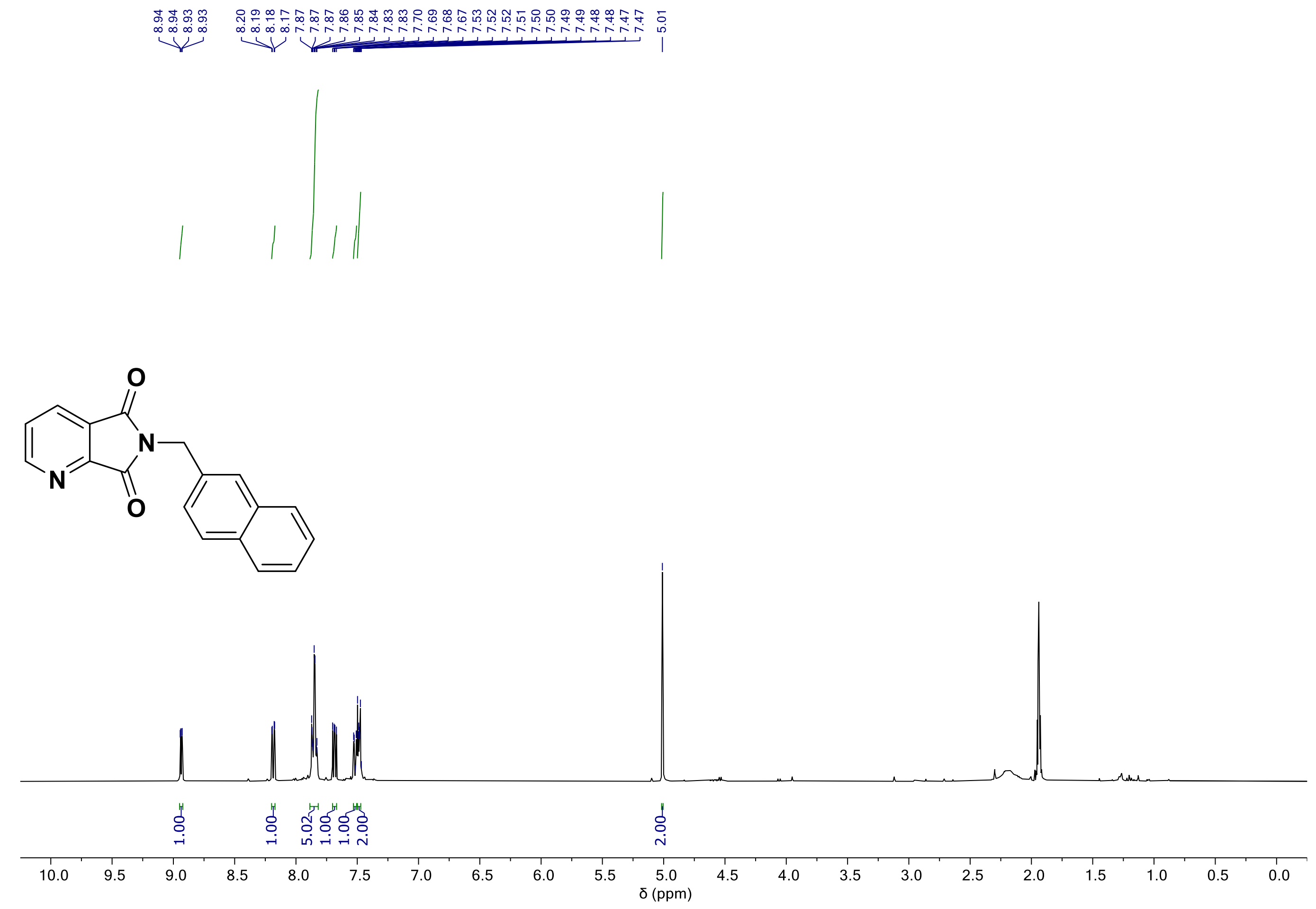

^{13}C NMR (Compound **S12**)

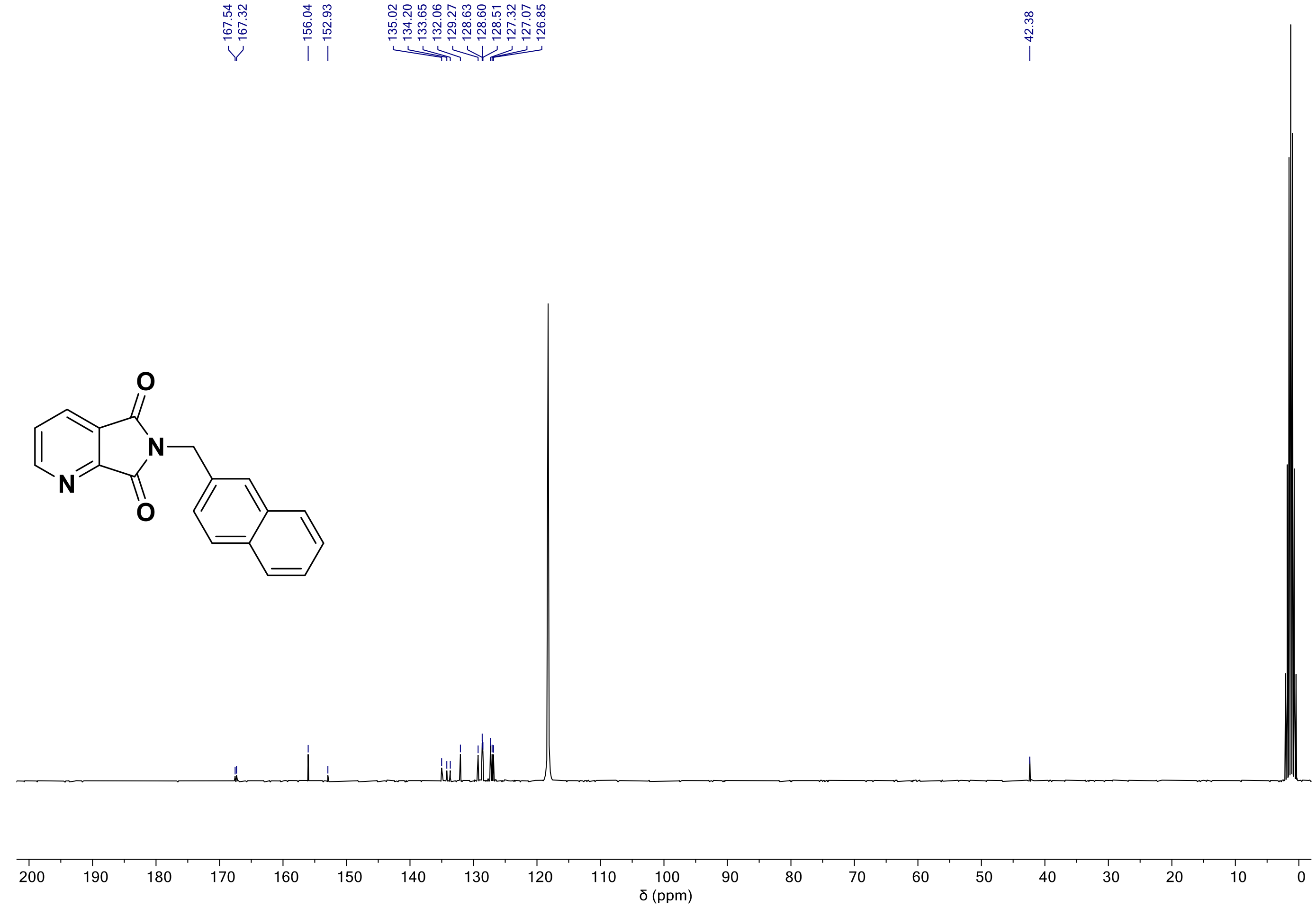

^{1}H NMR (Compound **S13**)

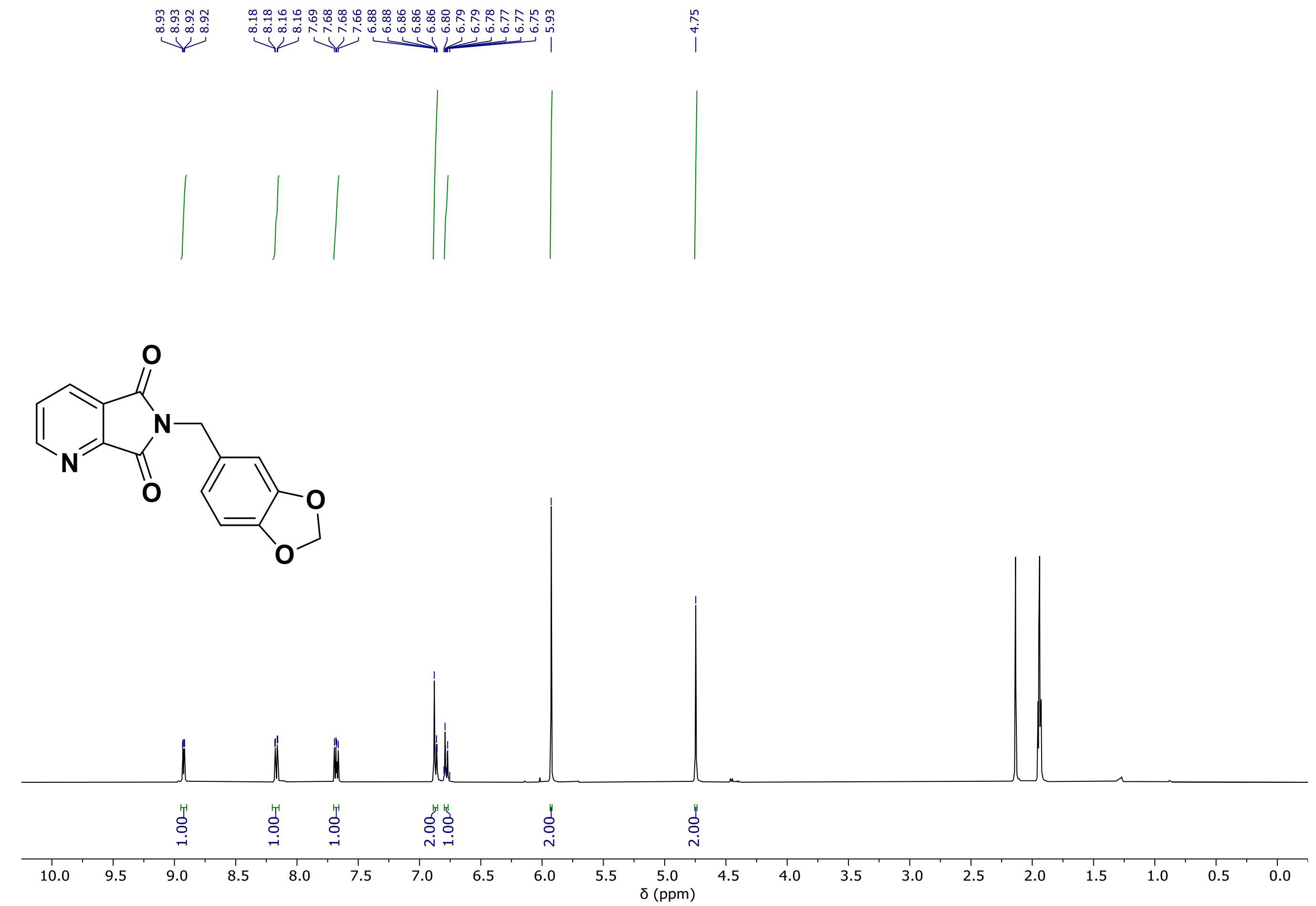

^{13}C NMR (Compound **S13**)

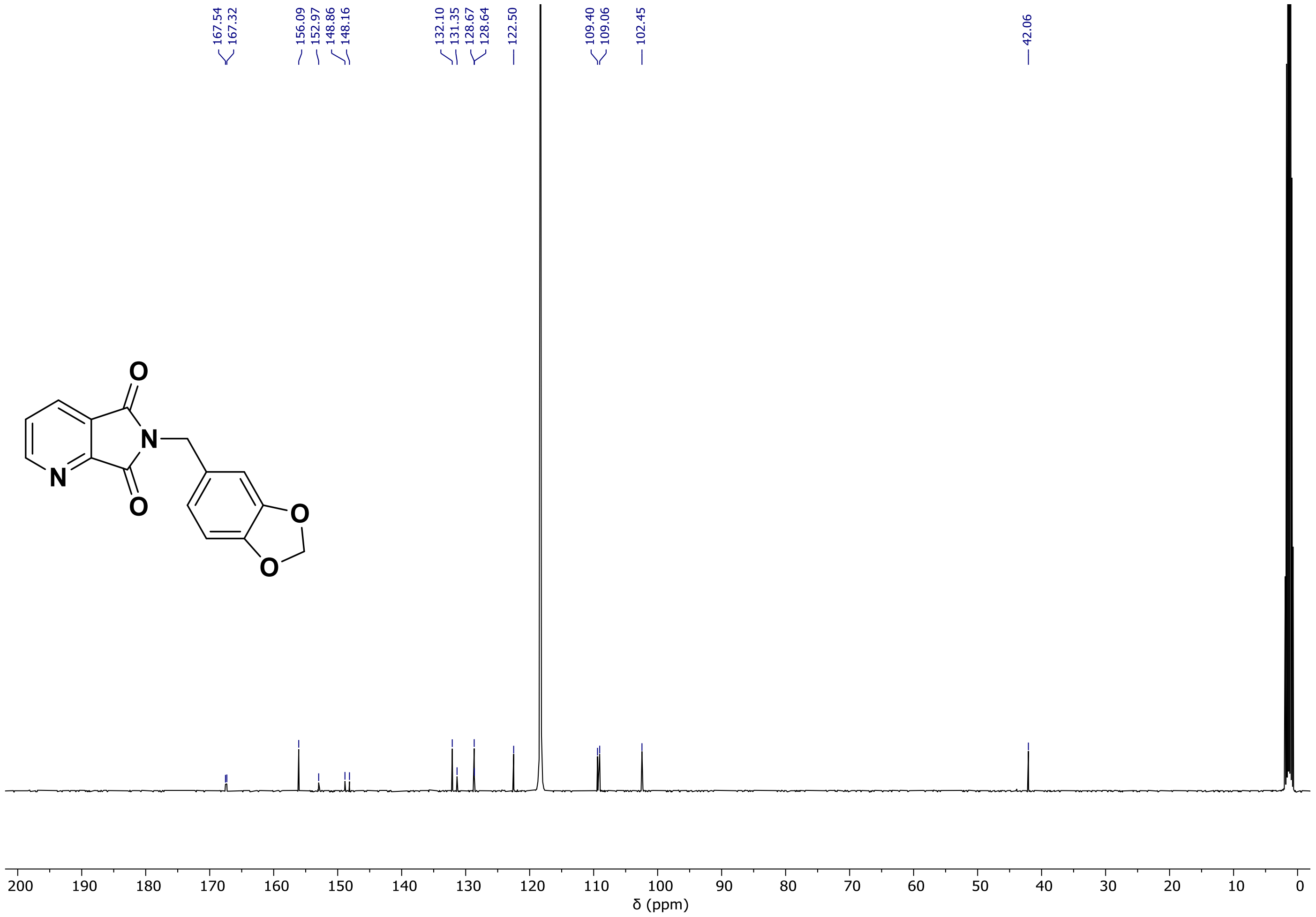

^{1}H NMR (Compound **S14**)

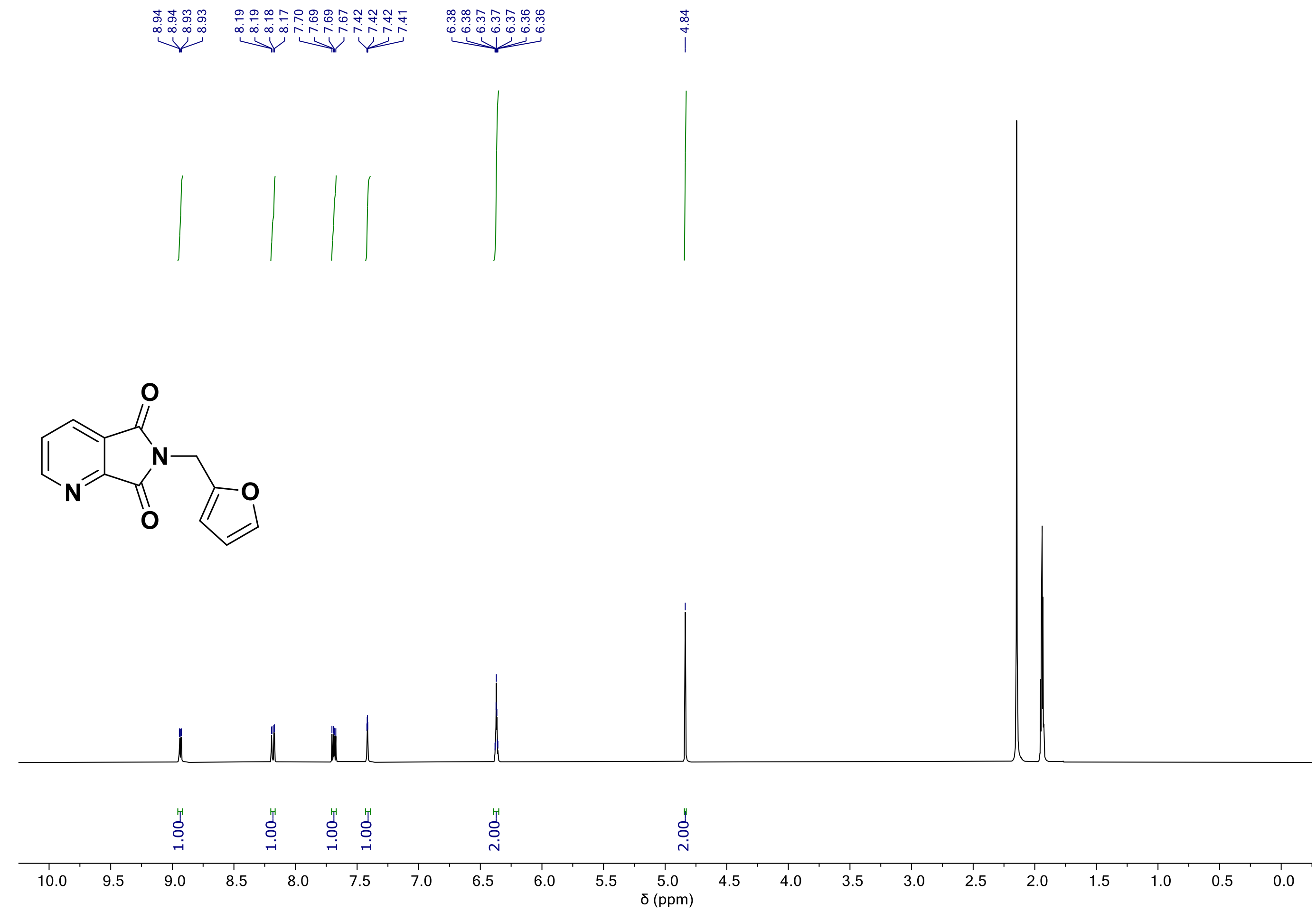

^{13}C NMR (Compound **S14**)

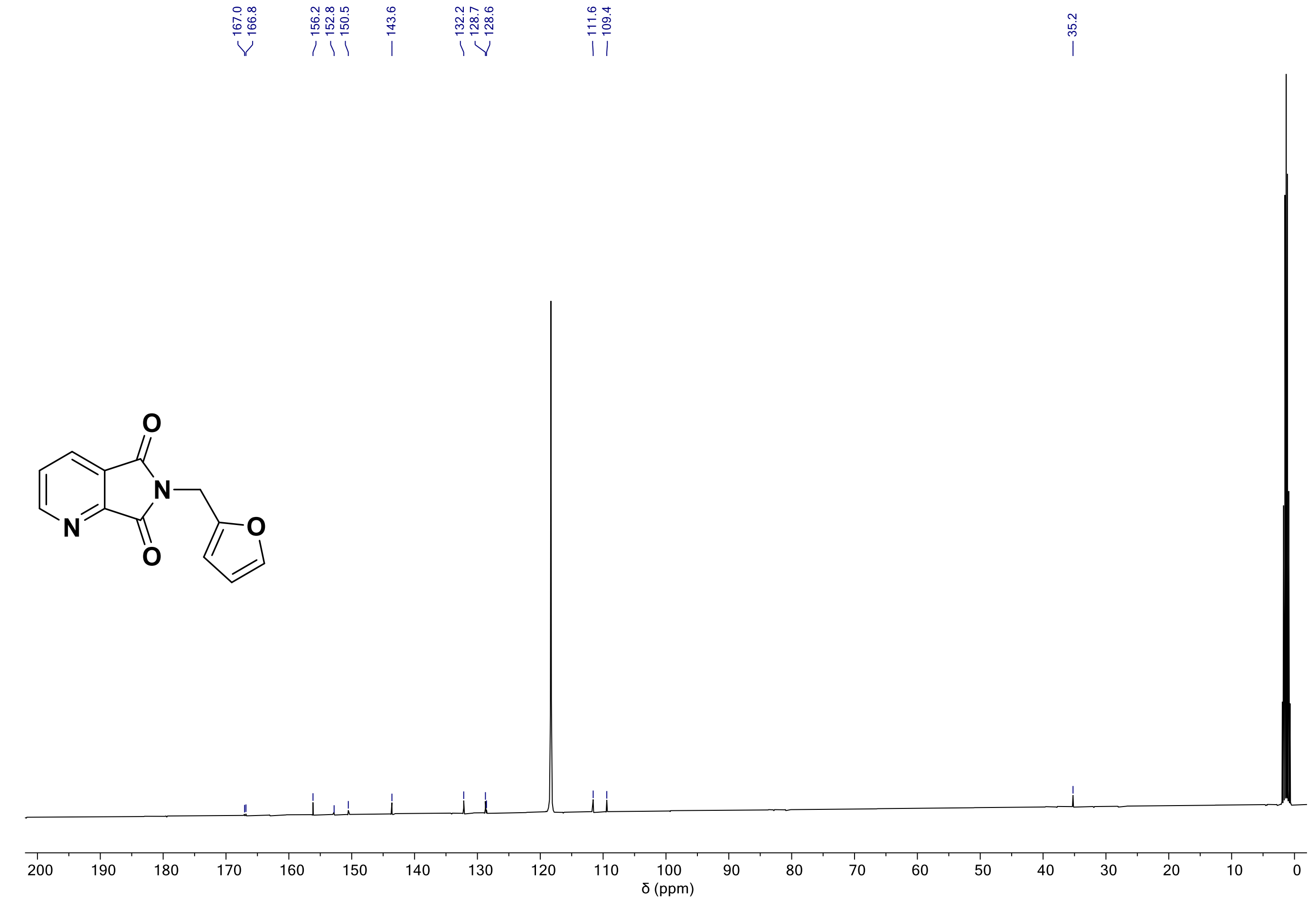

^{1}H NMR (Compound **S15)**

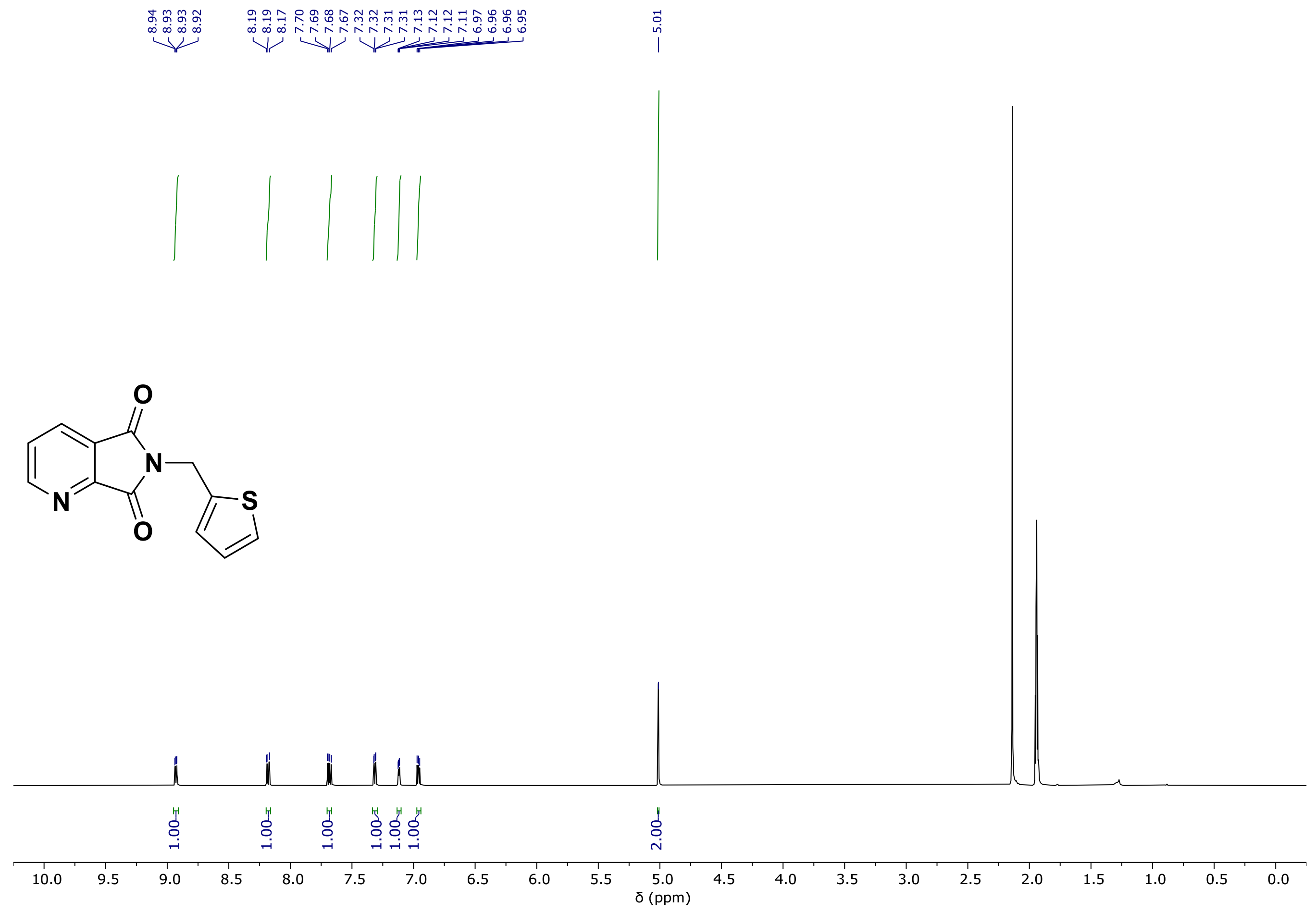

^{13}C NMR (Compound **S15**)

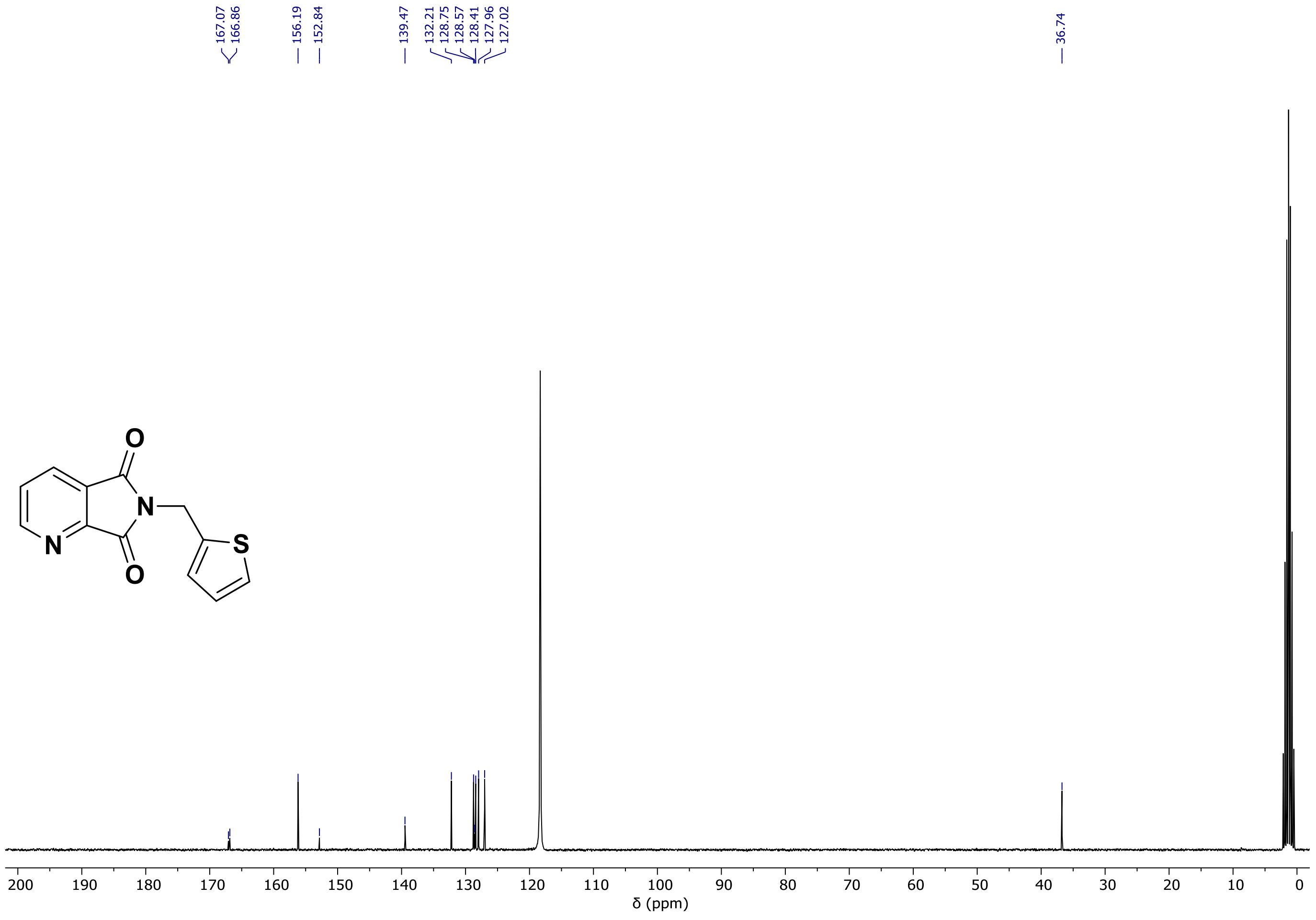

^{1}H NMR (Compound **S16)**

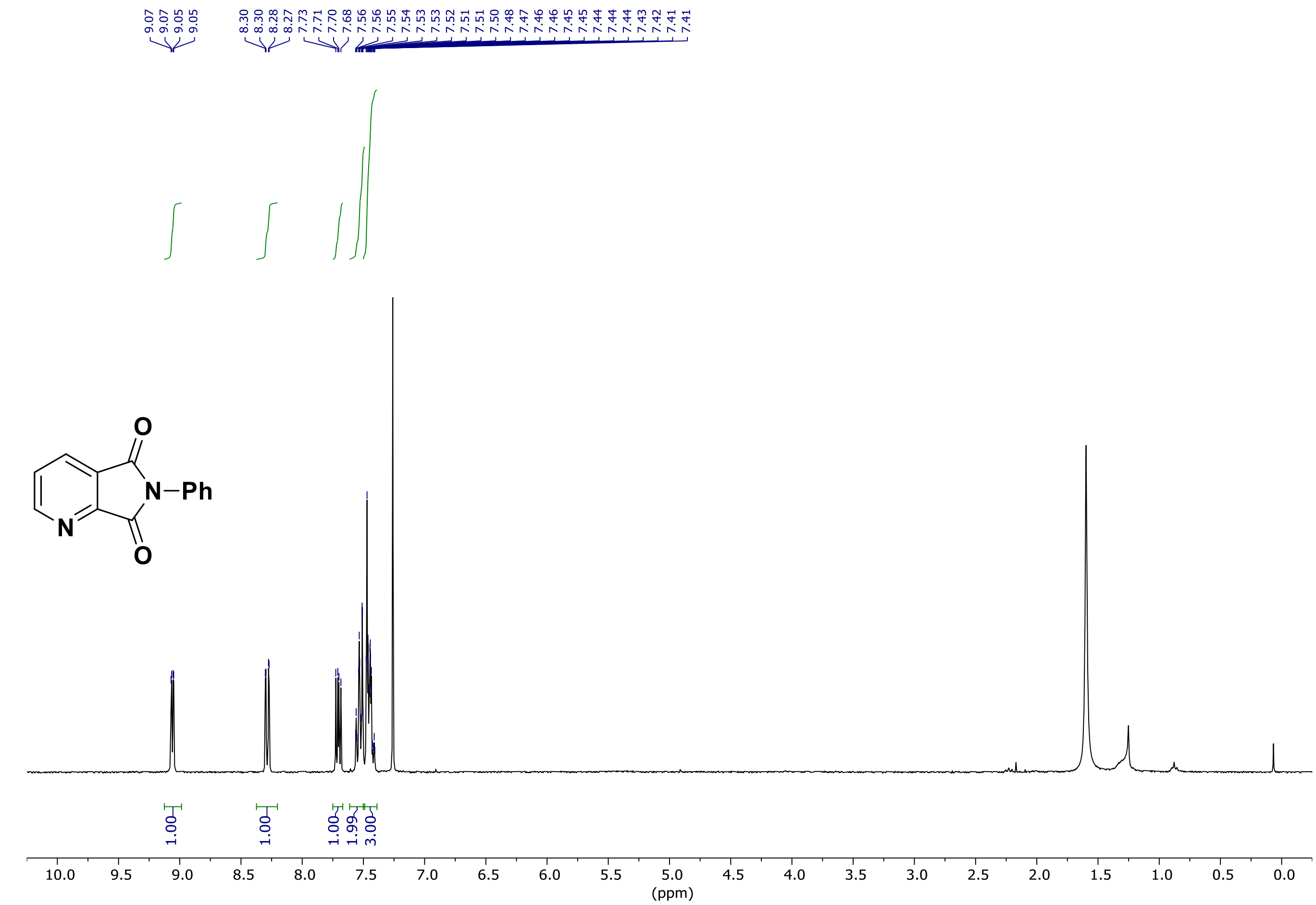

^{13}C NMR (Compound **S16**)

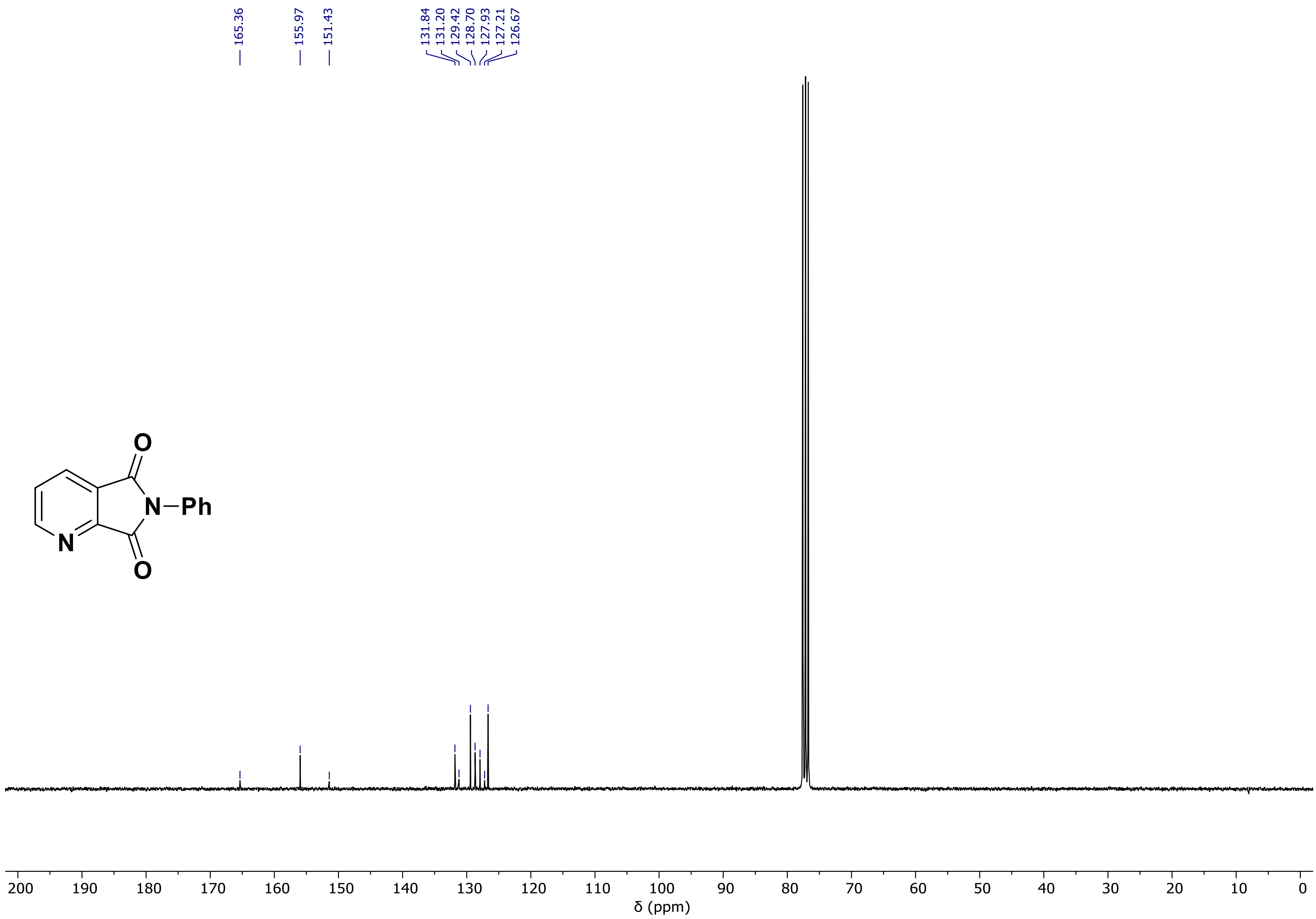

^{1}H NMR (Compound **S17**)

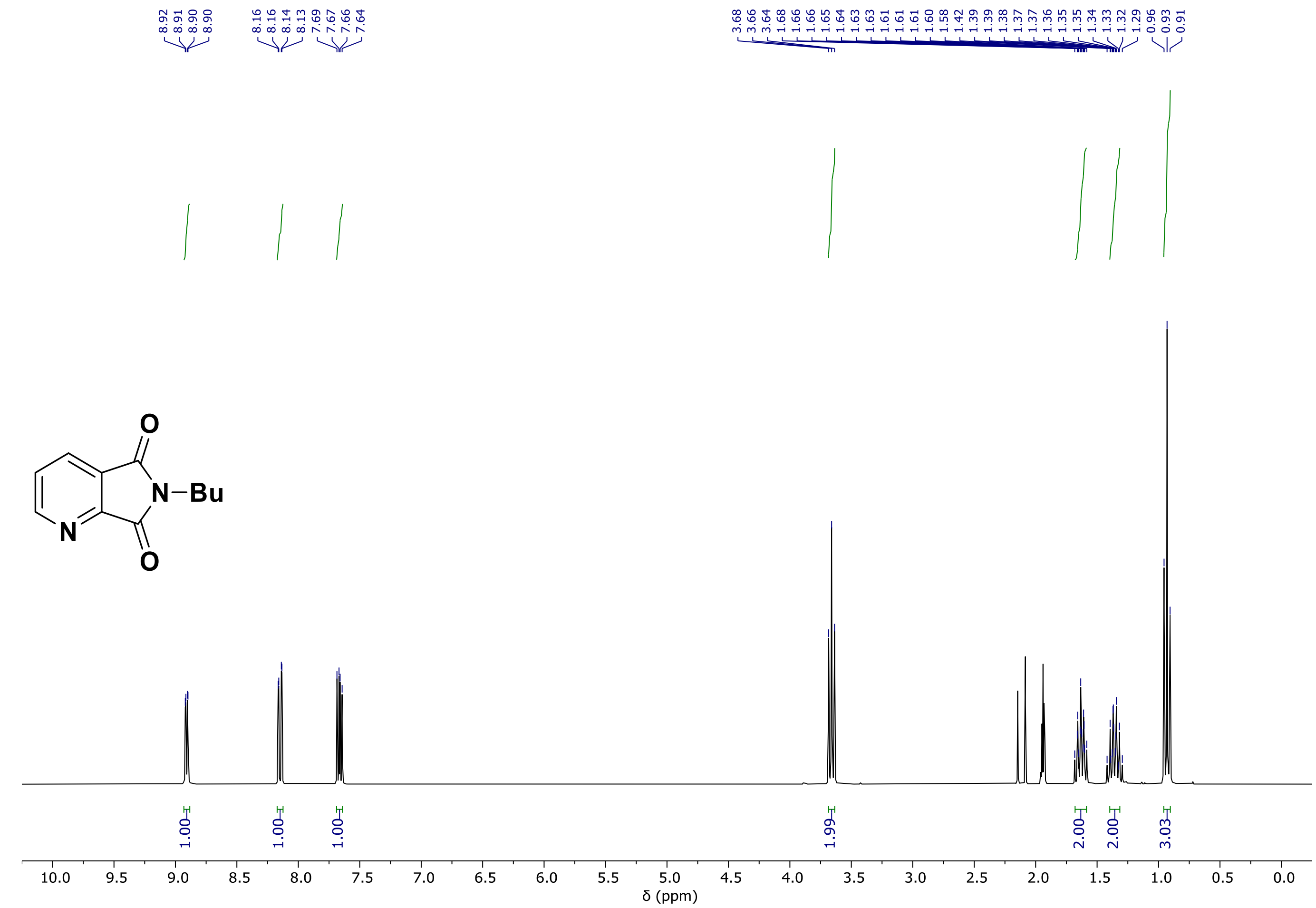

^{13}C NMR (Compound **S17**)

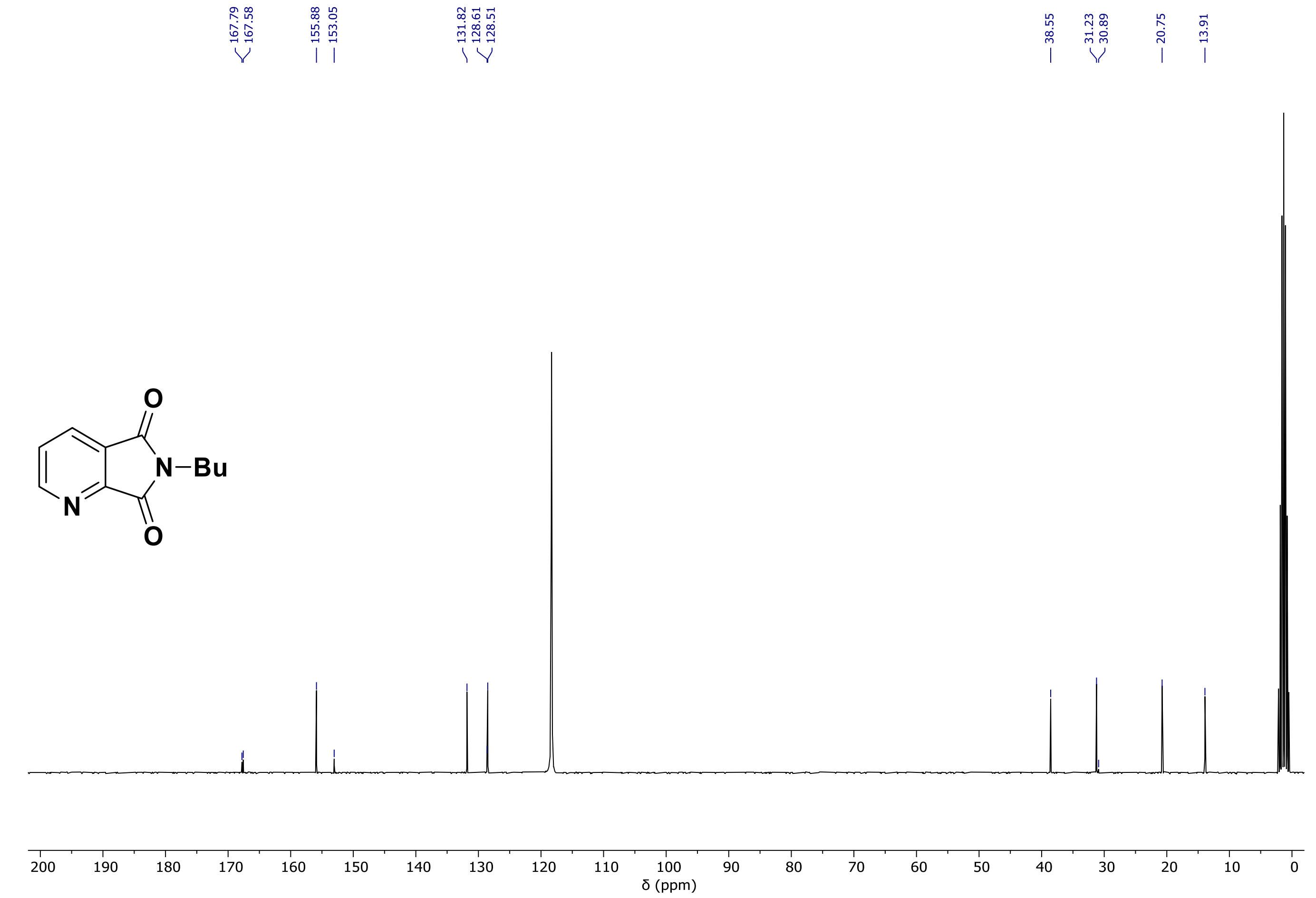

^{1}H NMR (Compound **S18)**

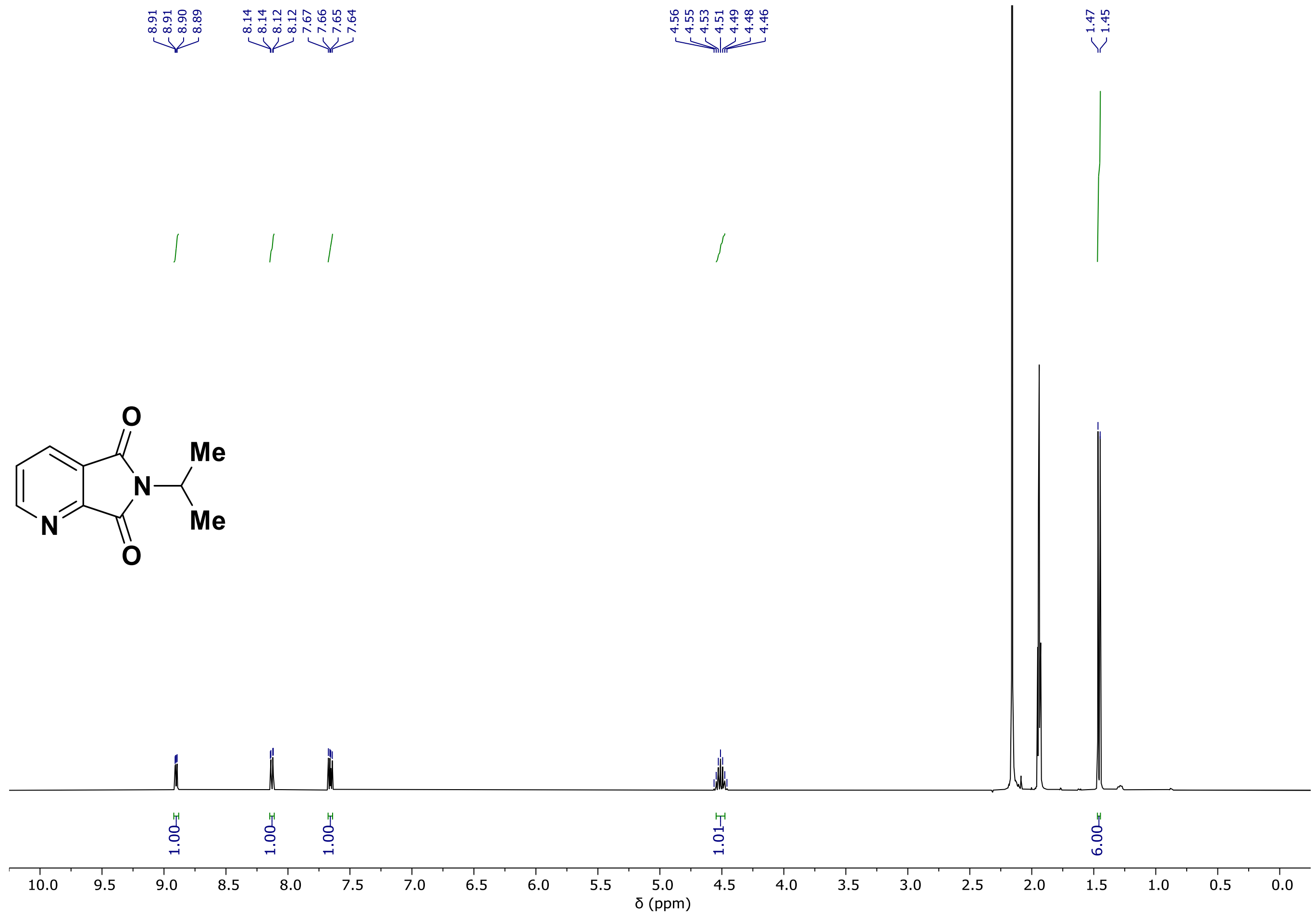

^{13}C NMR (Compound **S18)**

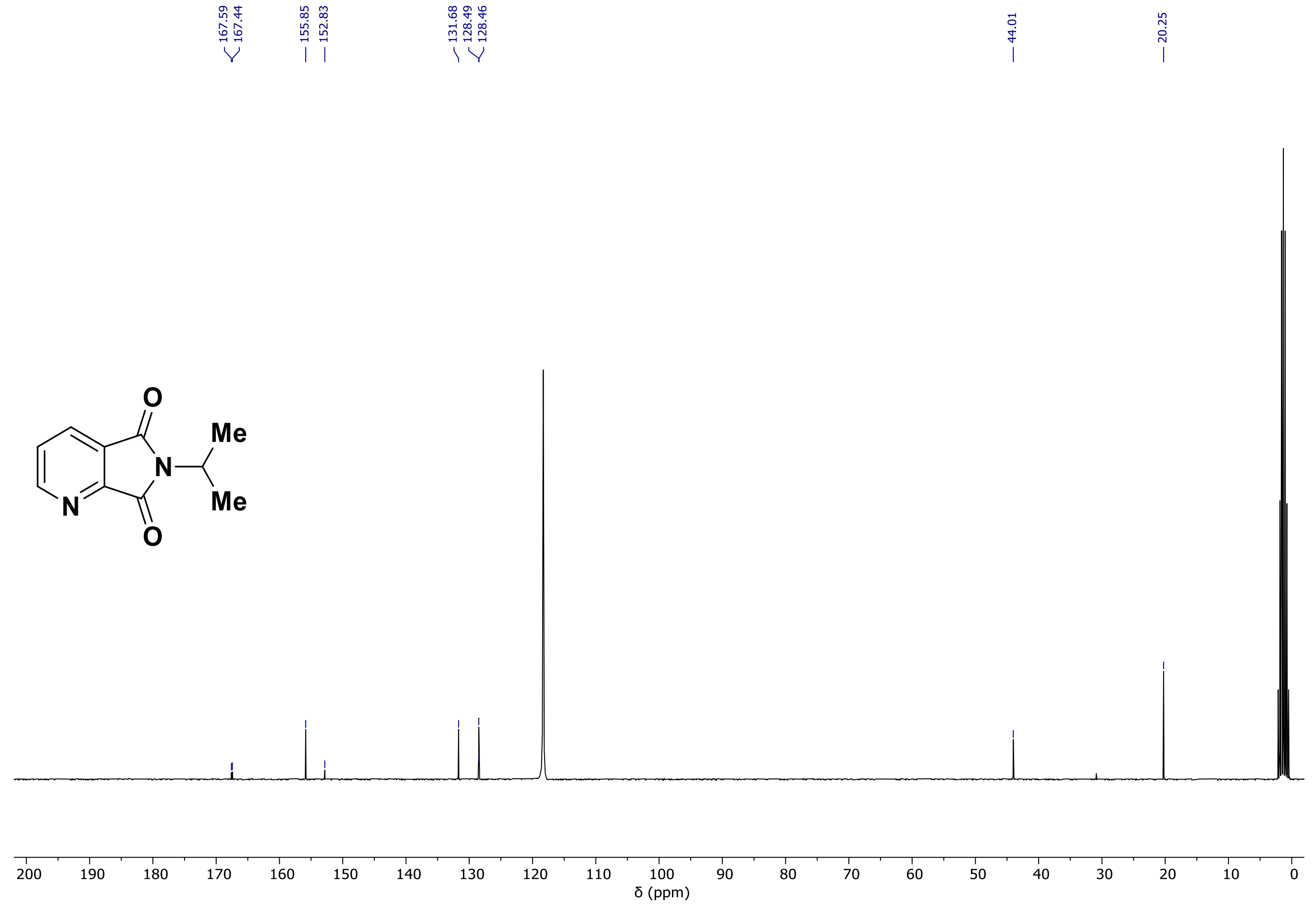

^{1}H NMR (Compound **S19)**

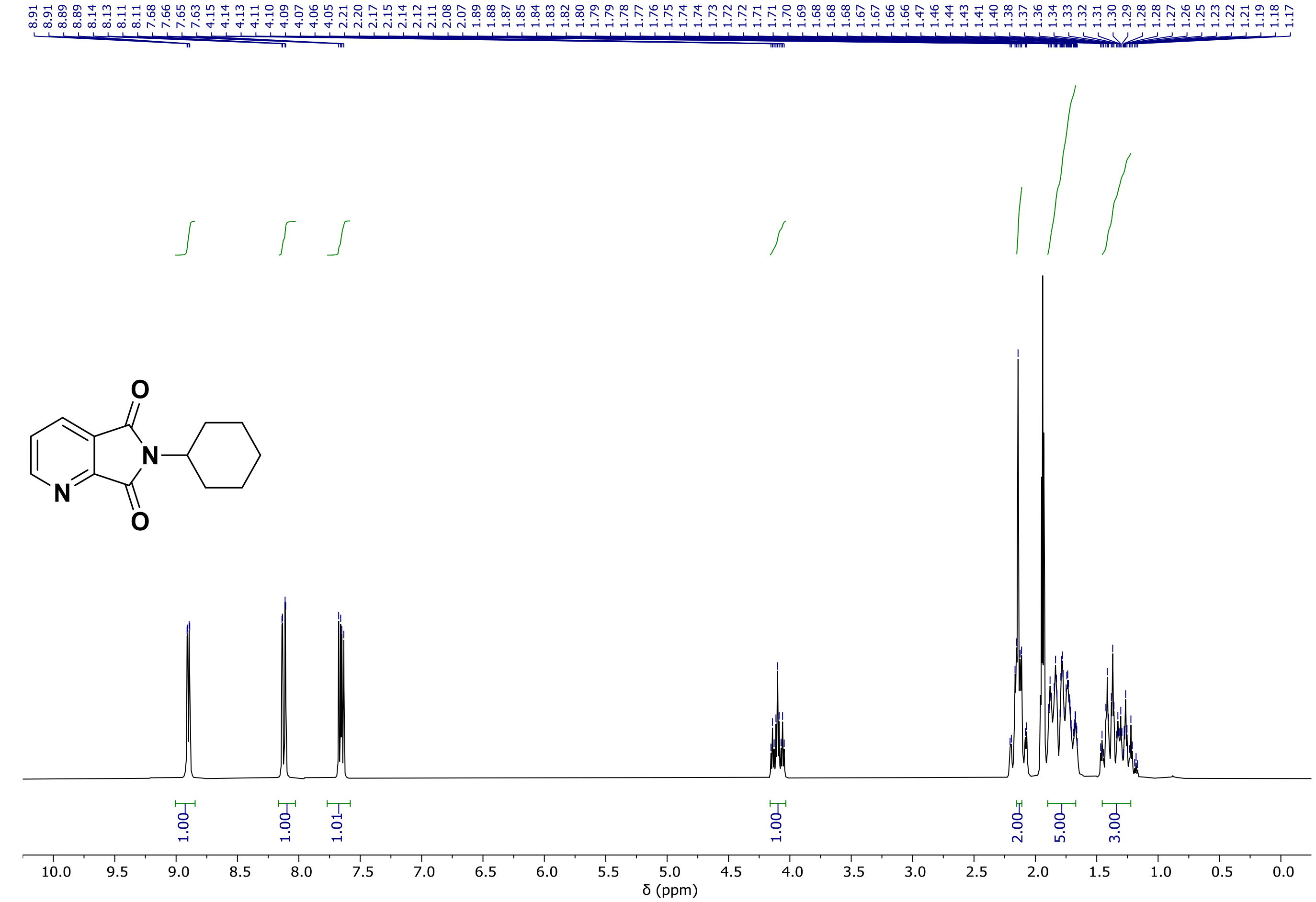

^{13}C NMR (Compound **S19)**

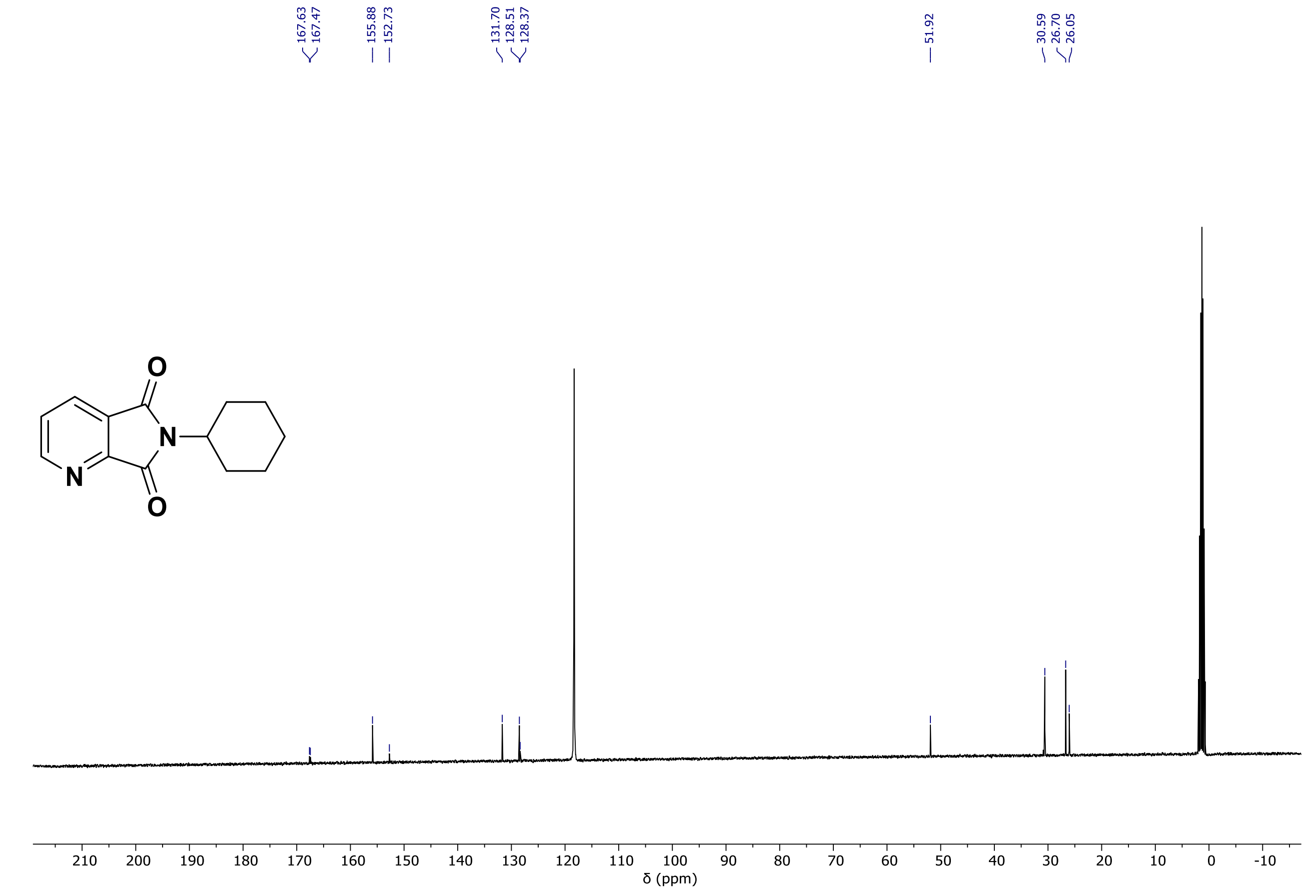

^{1}H NMR (Compound **S20)**

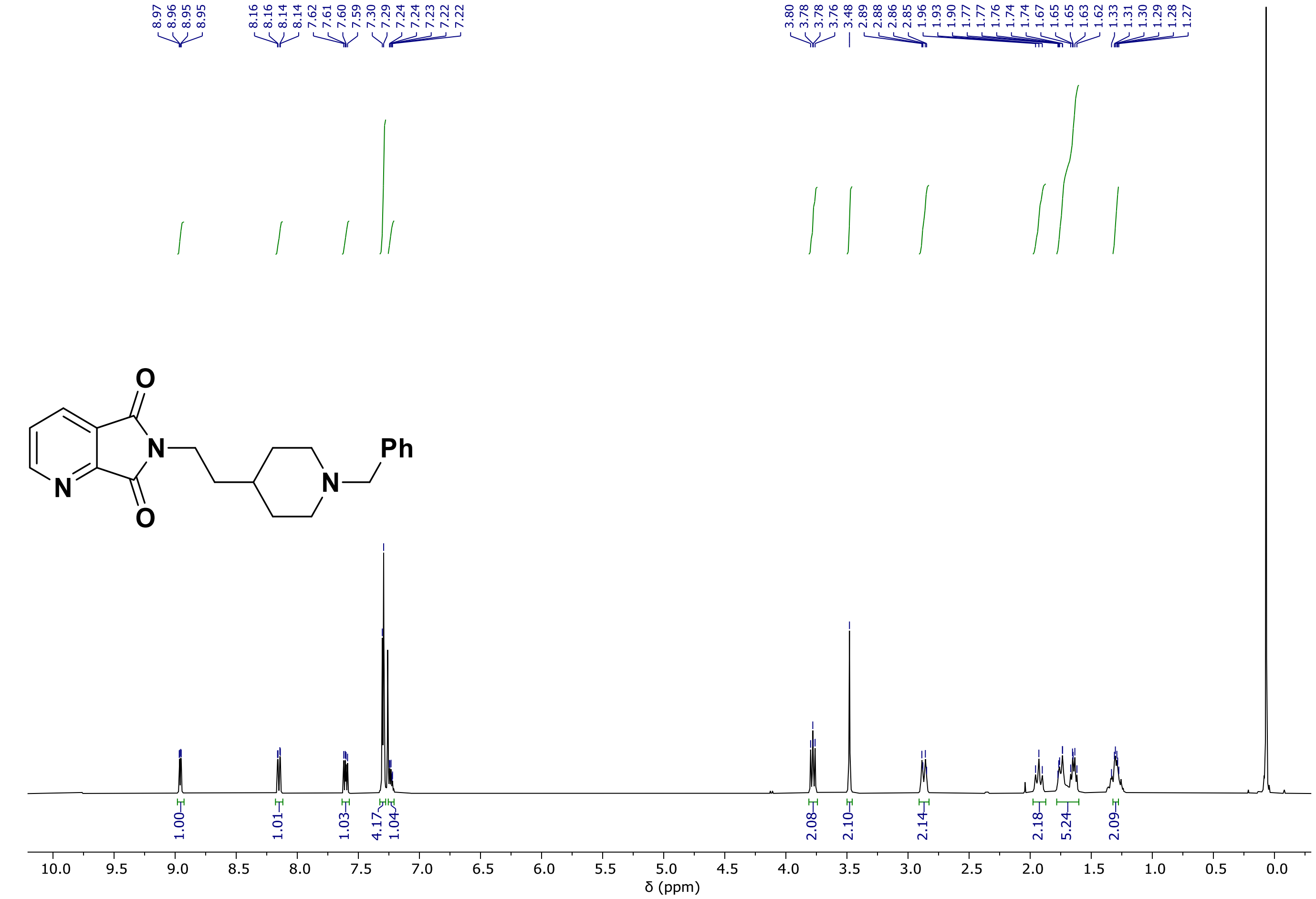

^{13}C NMR (Compound **S20)**

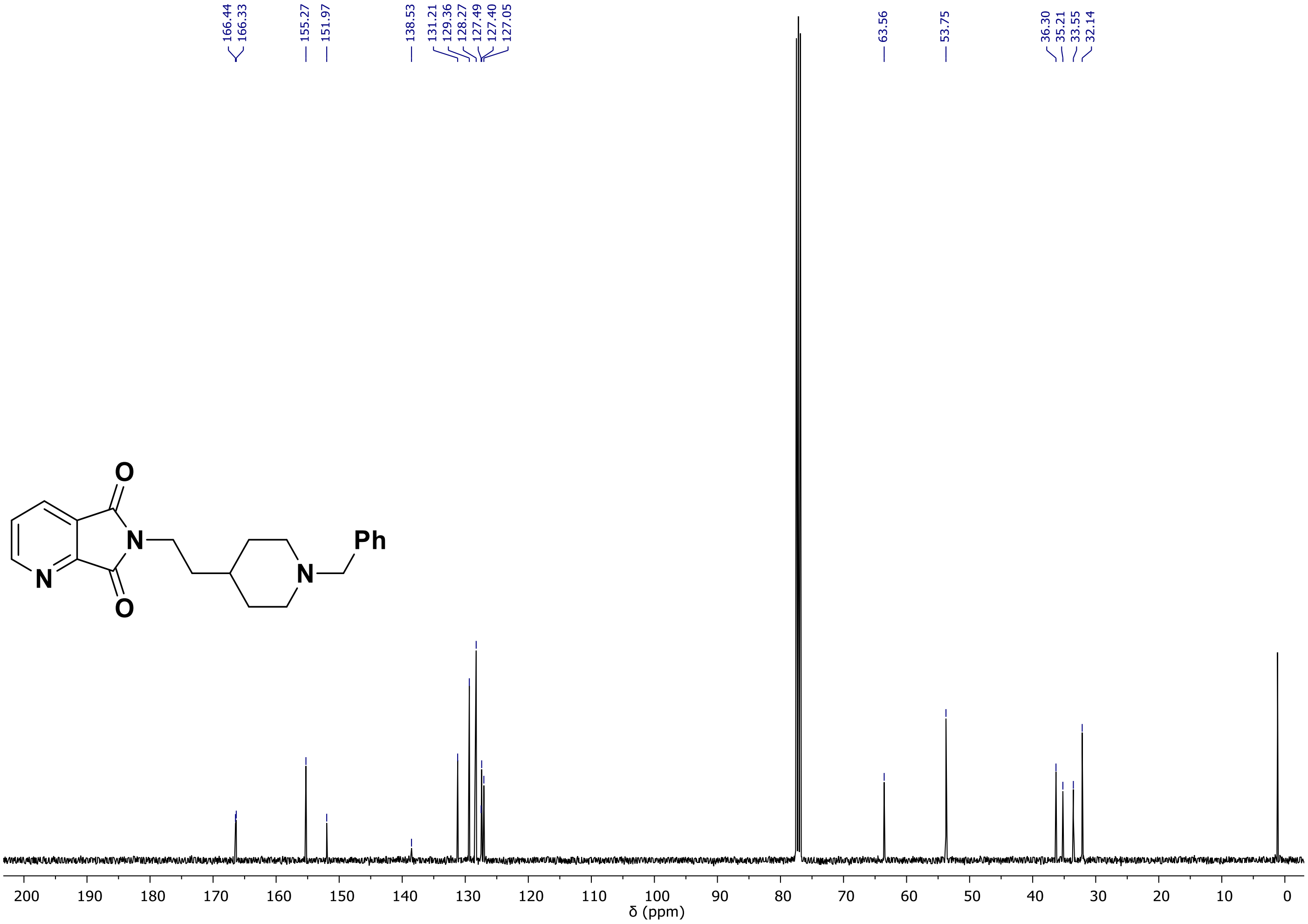

^{1}H NMR (Compound **2**)

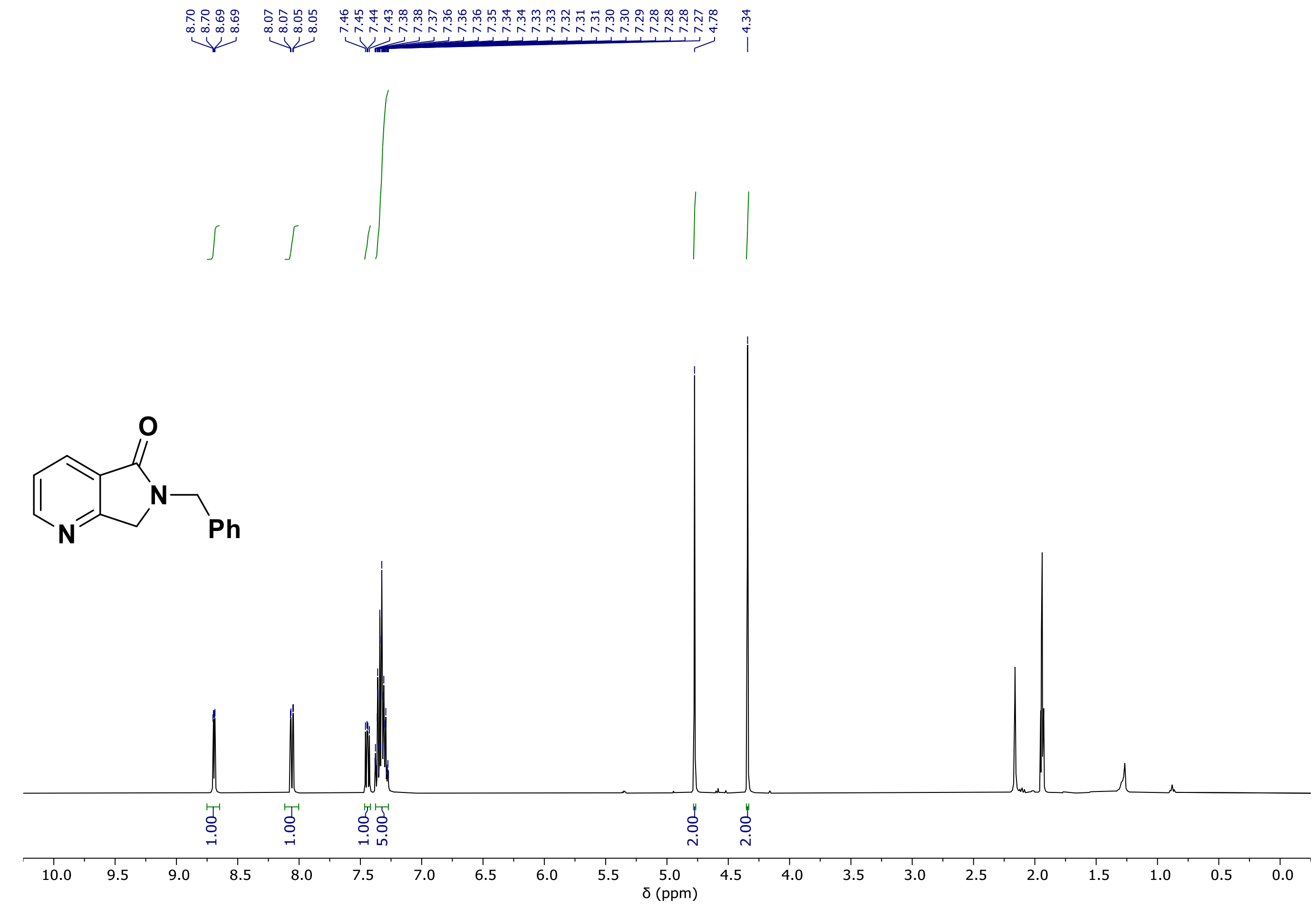

^{13}C NMR (Compound **2**)

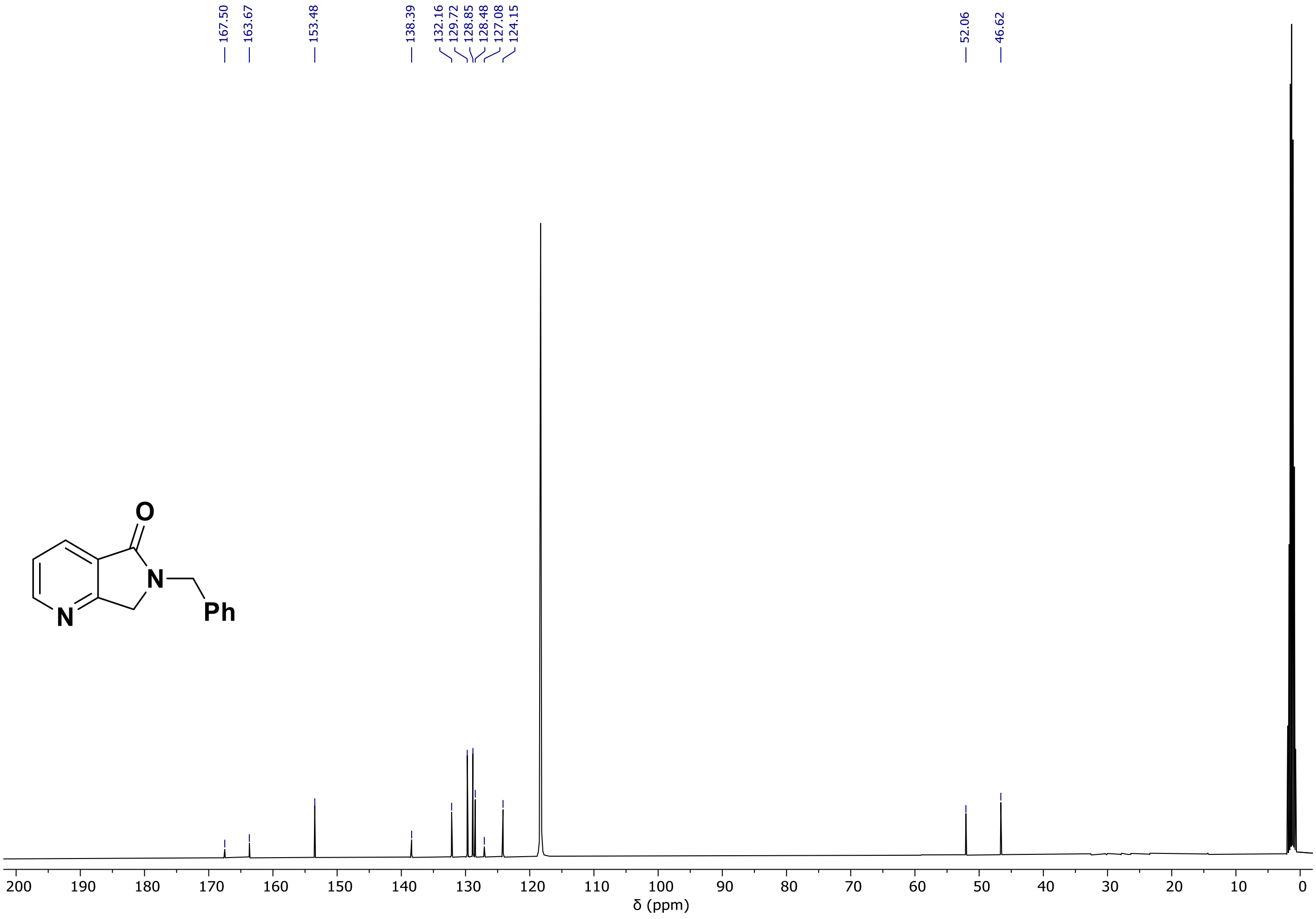

^1H – ^{13}C **HMBC** (Compound **2**)

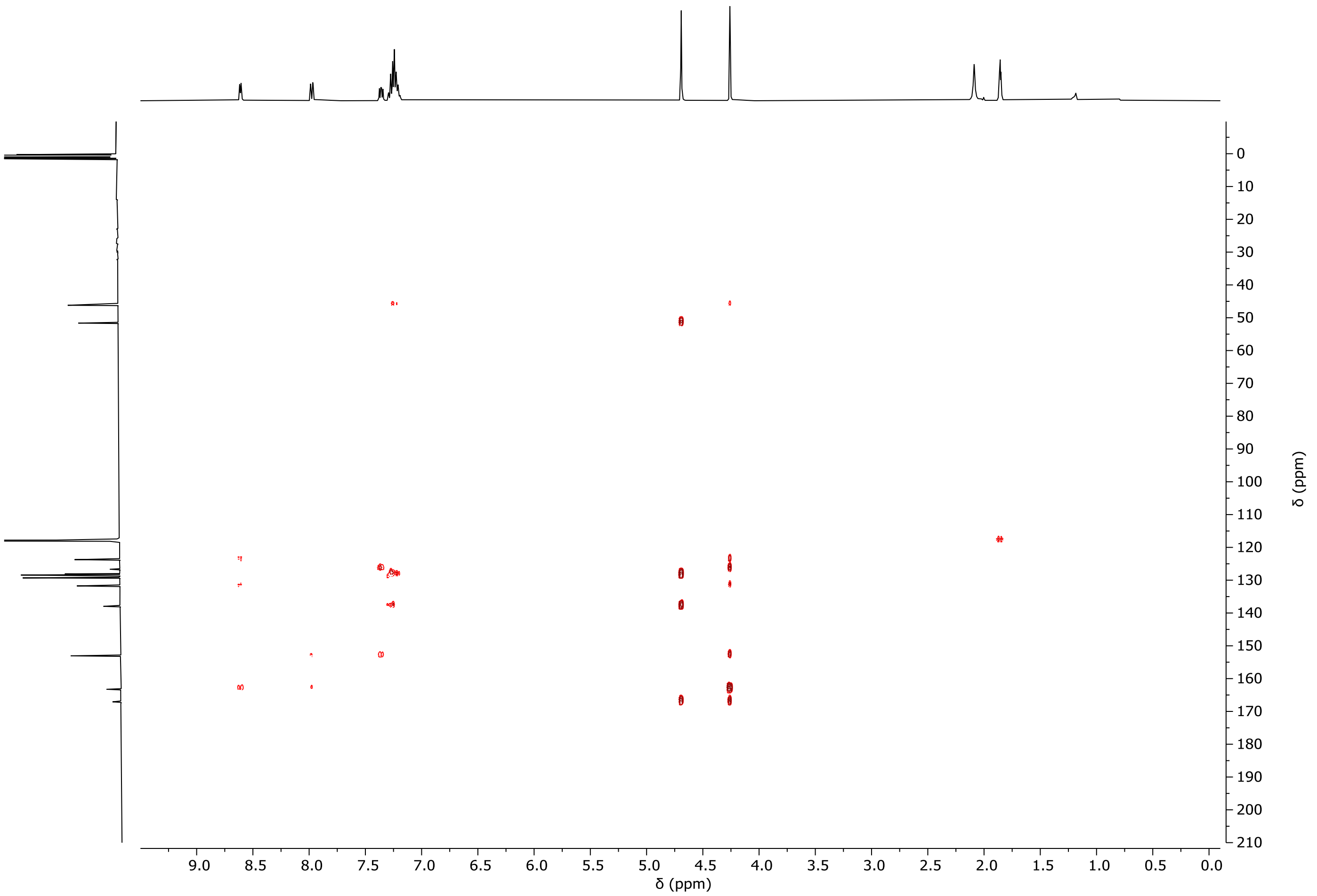

^1H – ^{13}C **HMBC (assessment of the aliphatic signals)** (Compound **2**)

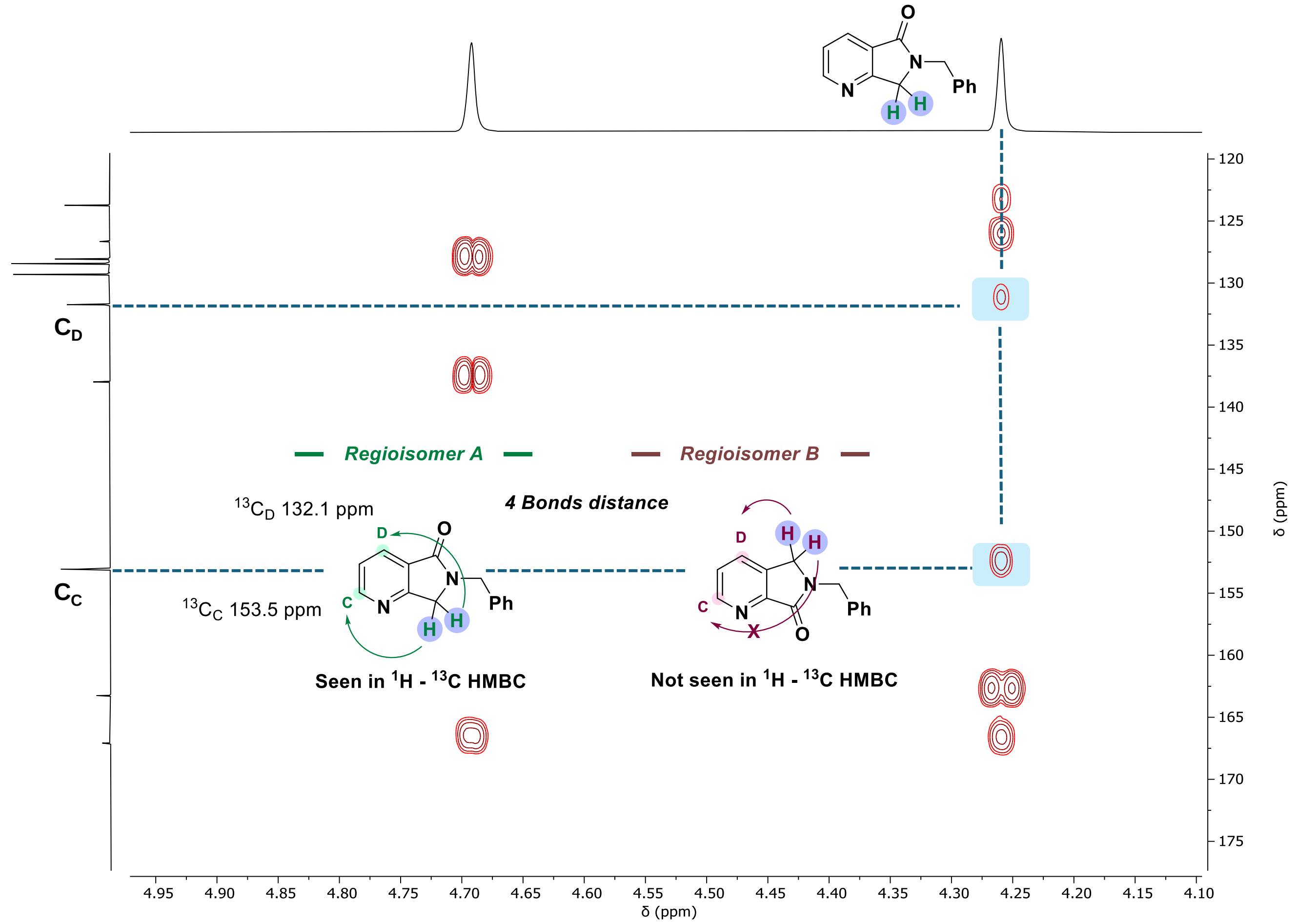

^{1}H – ^{13}C **HMBC (assessment of the aromatic signals)** (Compound **2**)

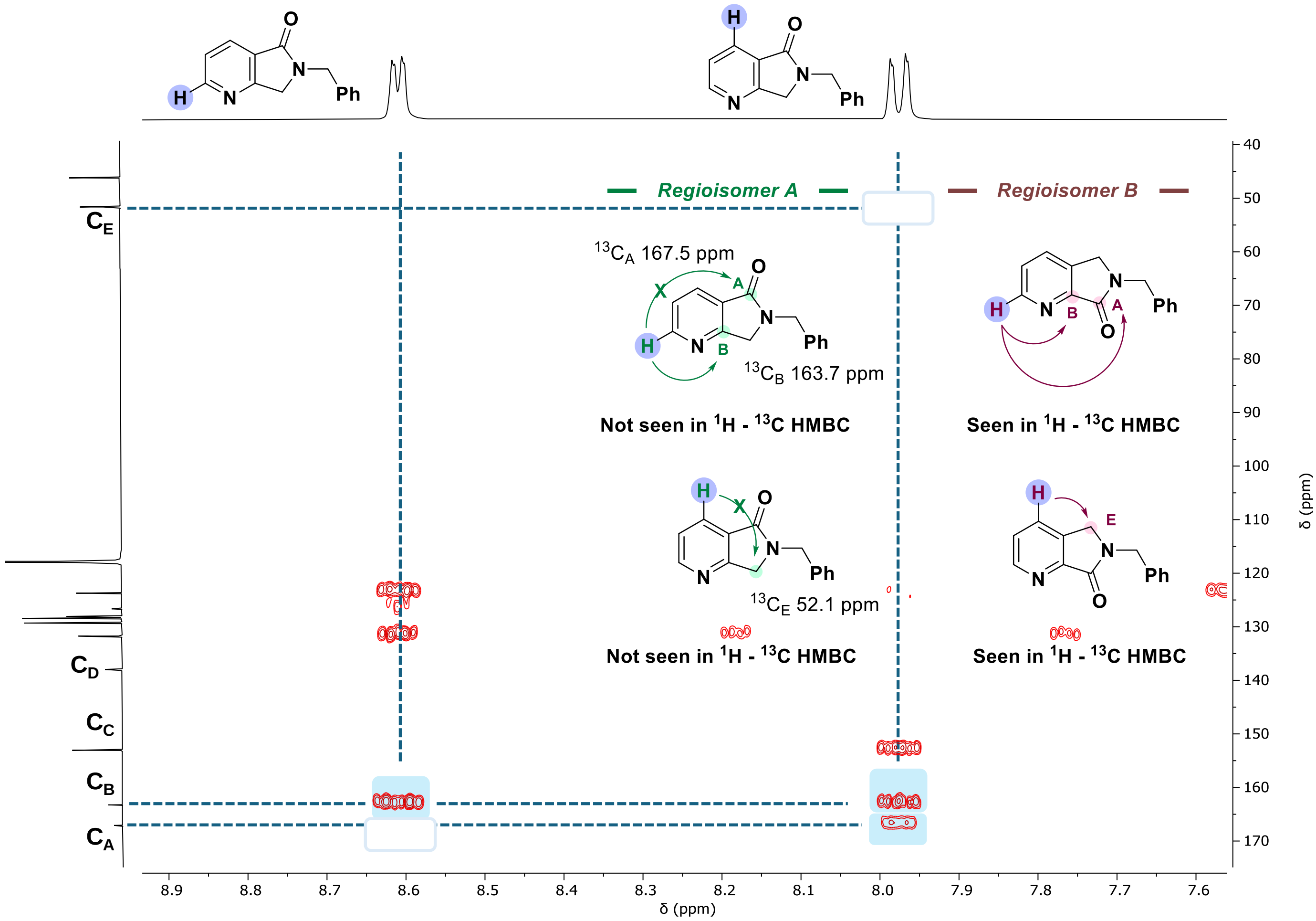

^{1}H NMR (Compound **6**)

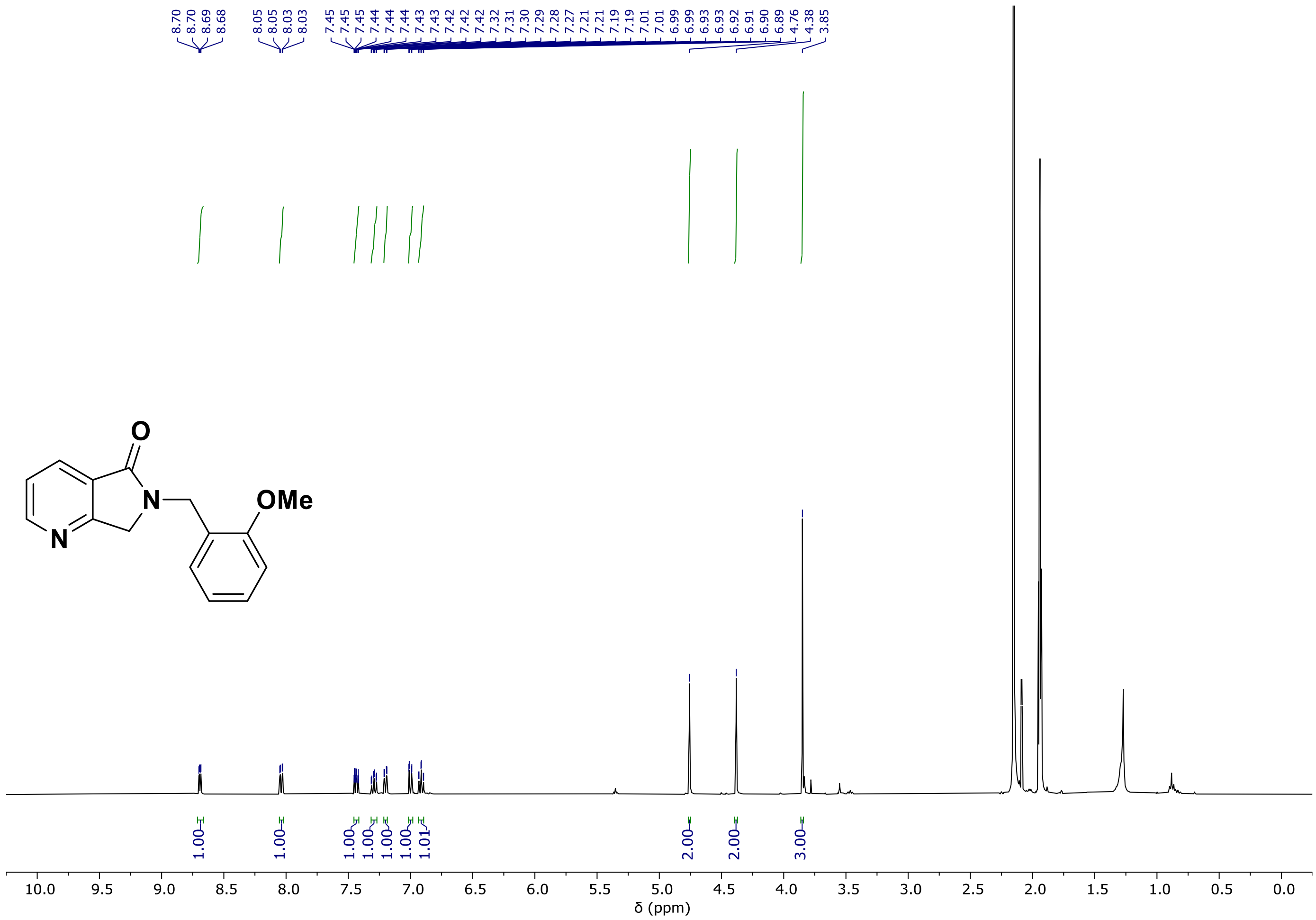

^{13}C NMR (Compound **6**)

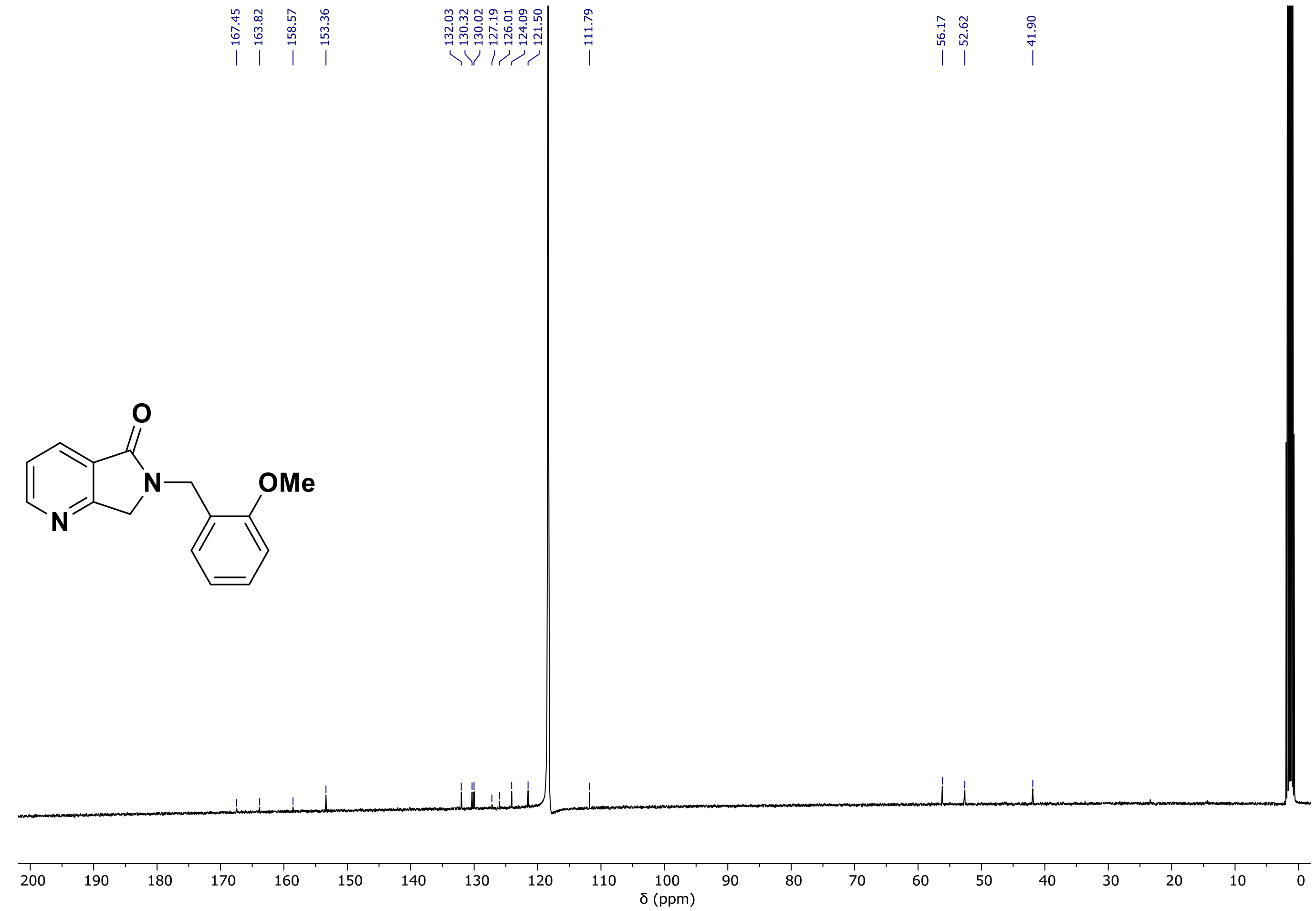

^{1}H NMR (Compound **7**)

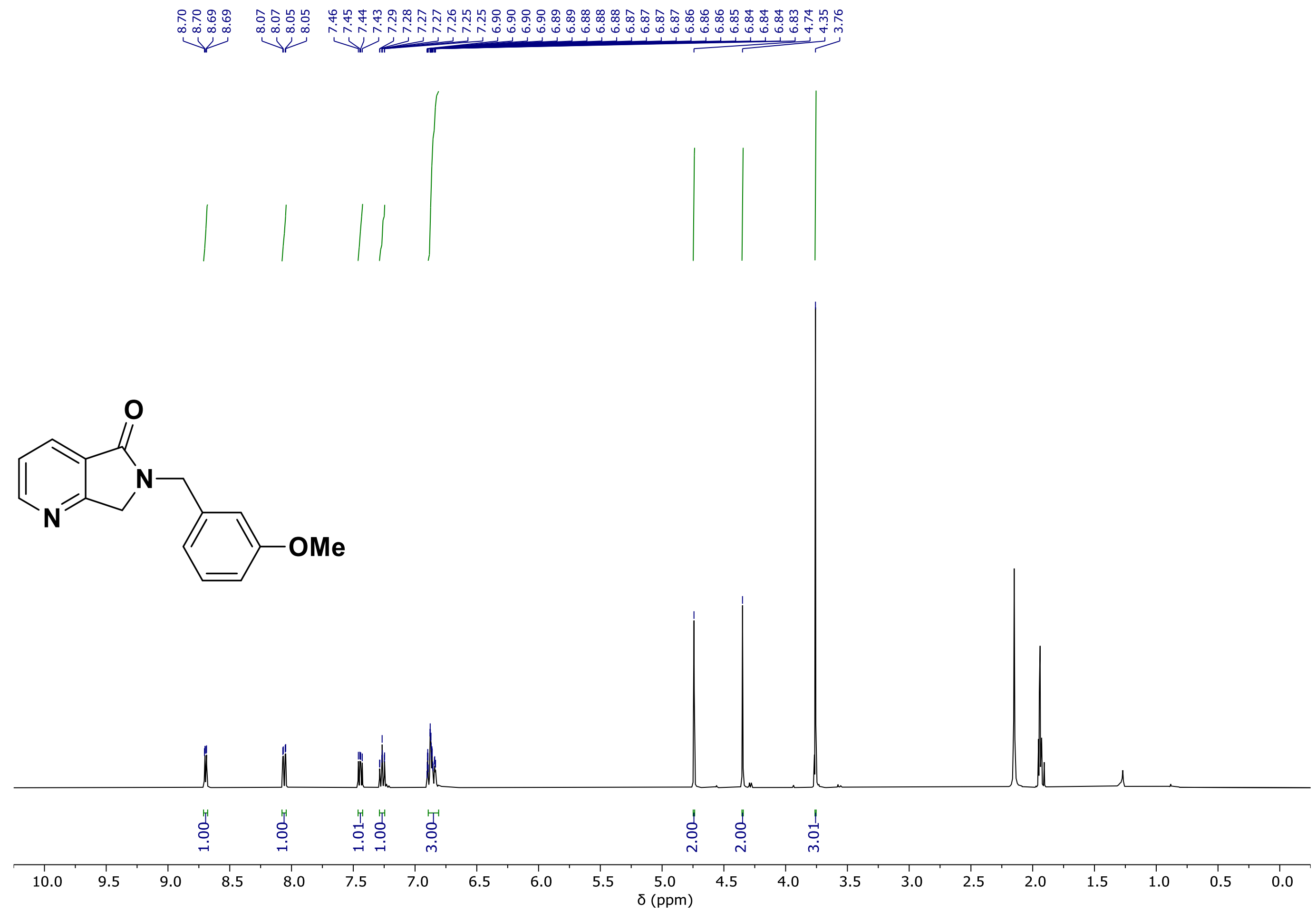

^{13}C NMR (Compound **7**)

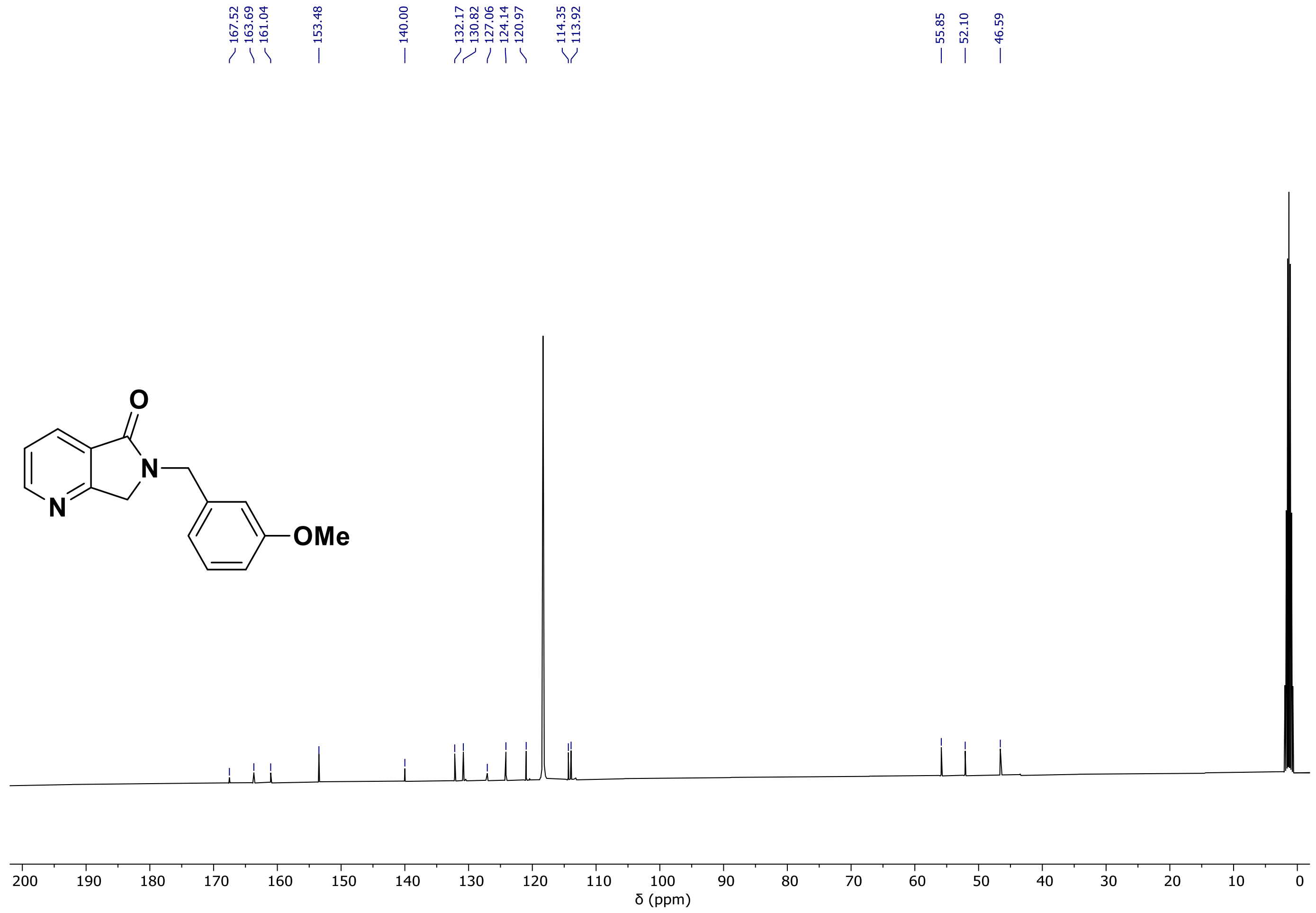

^{1}H NMR (Compound **8**)

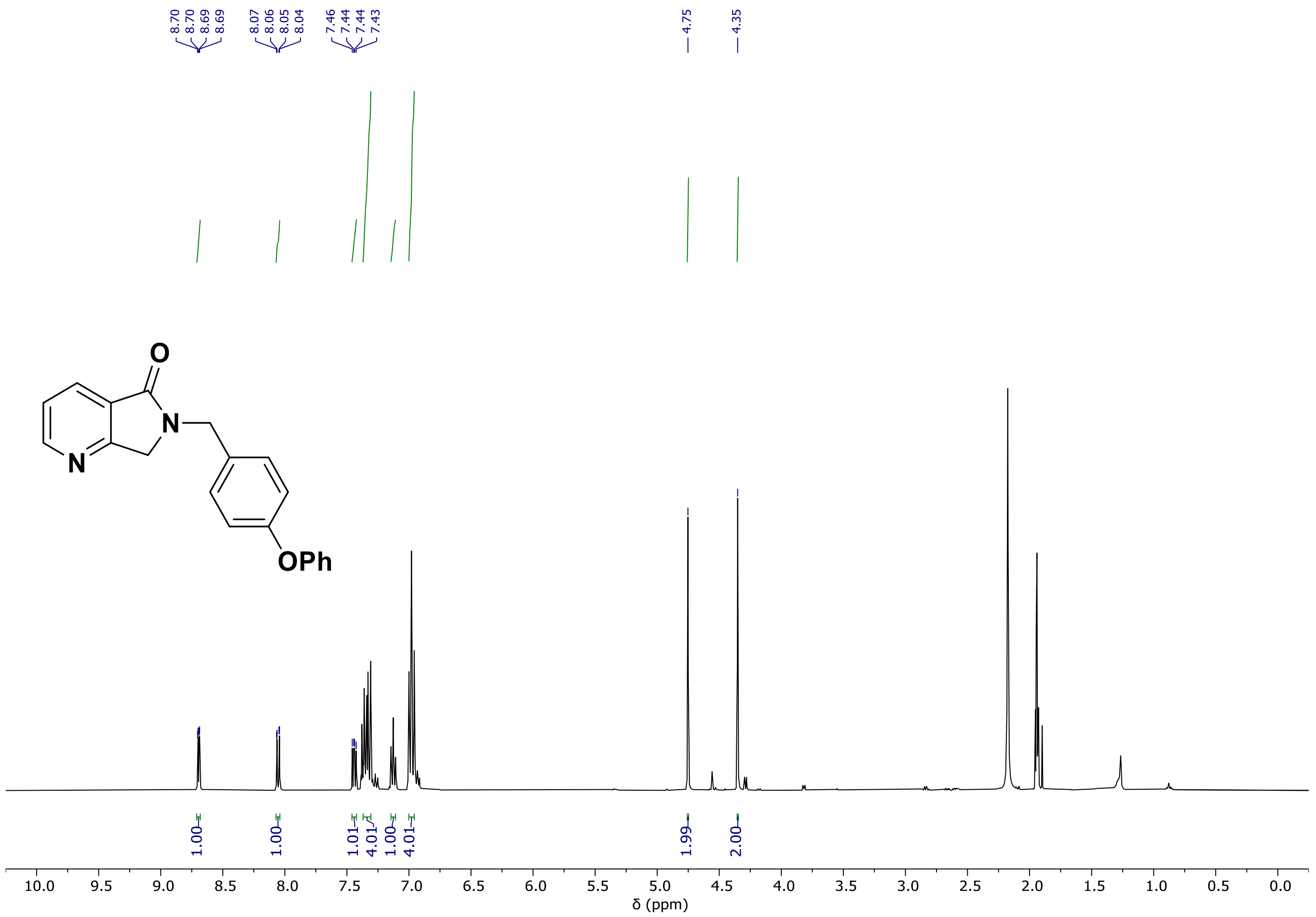

^{13}C NMR (Compound **8**)

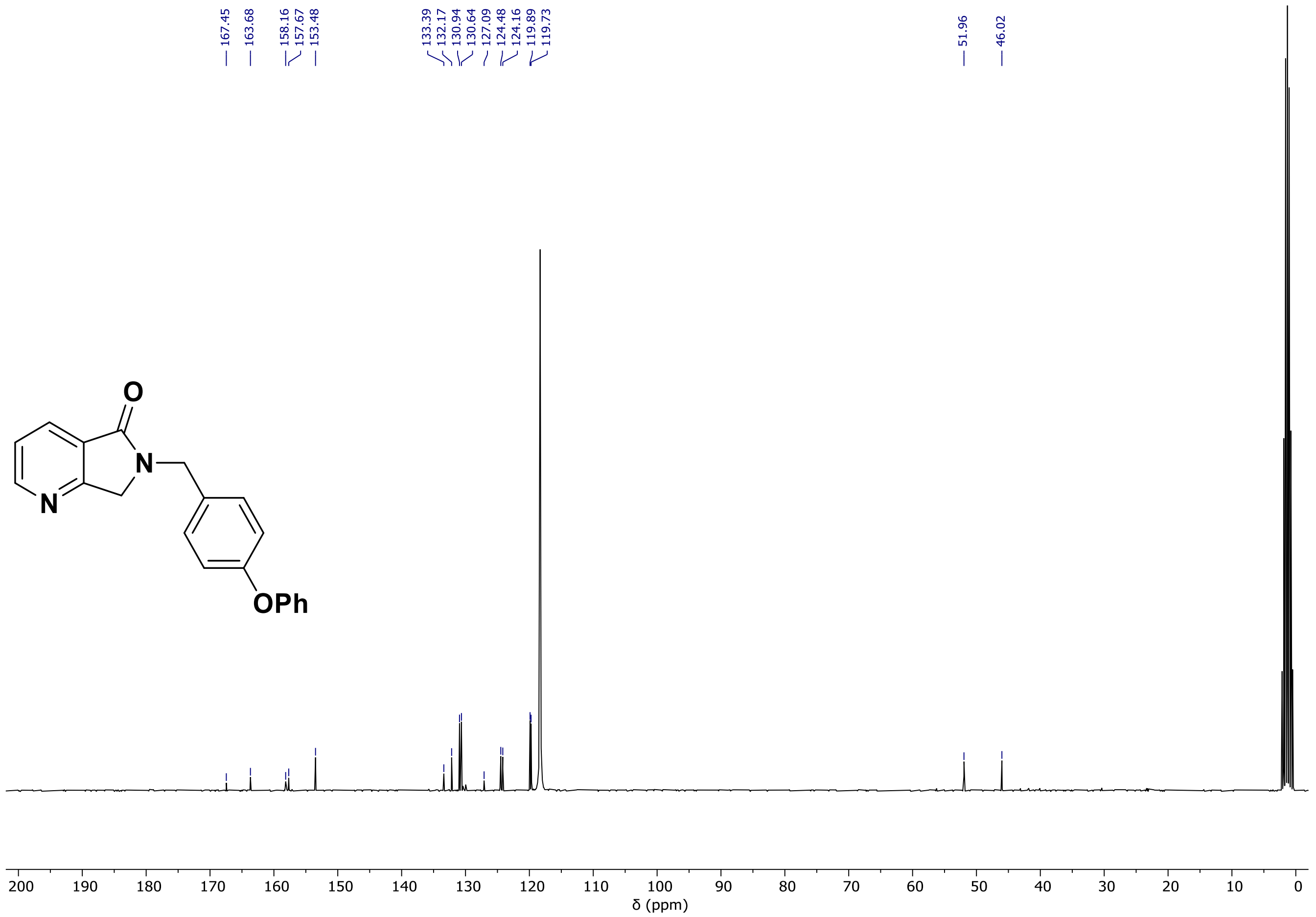

^{1}H NMR (Compound **9**)

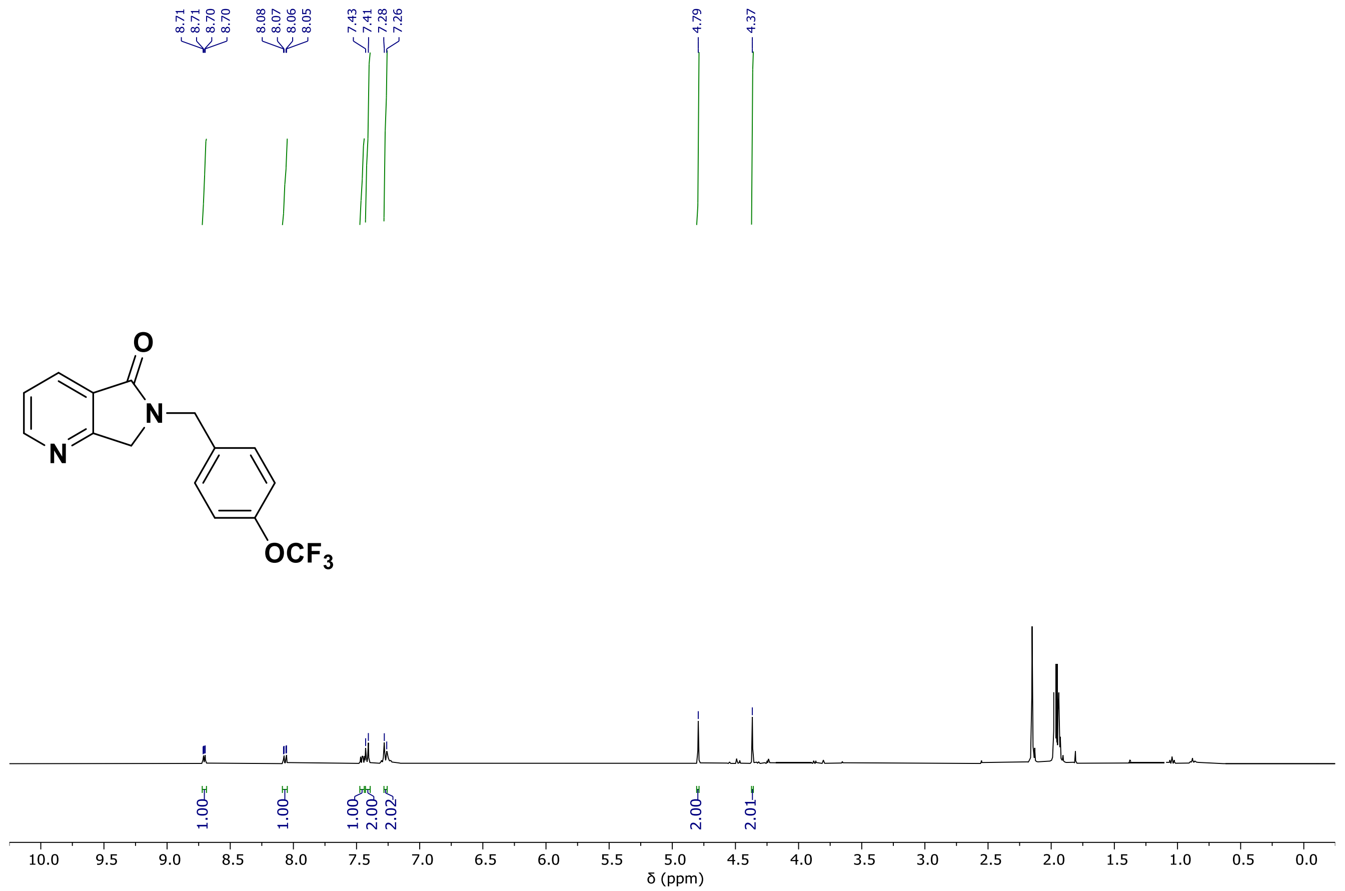

^{13}C NMR (Compound **9**)

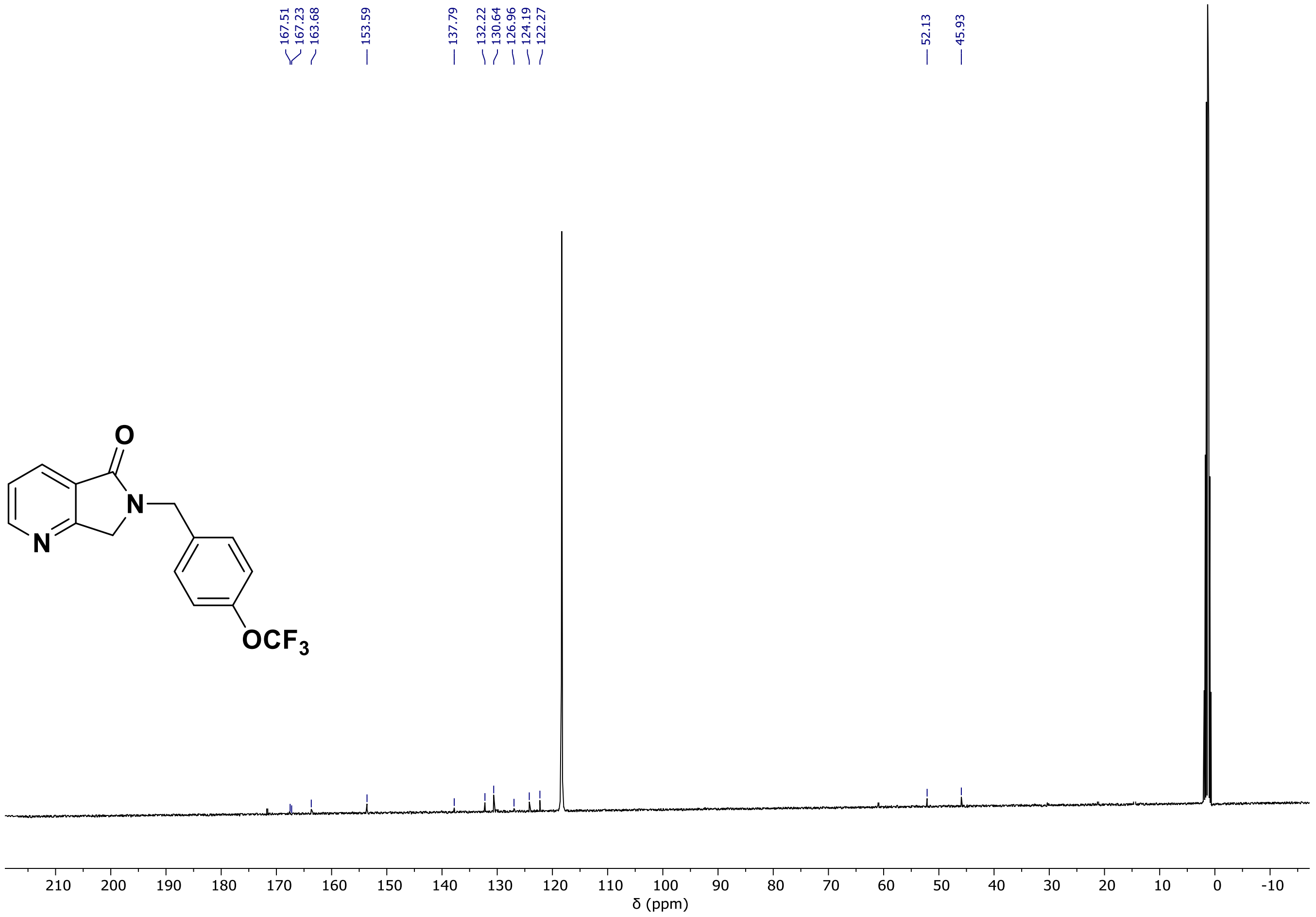

^{19}F NMR (Compound **9**)

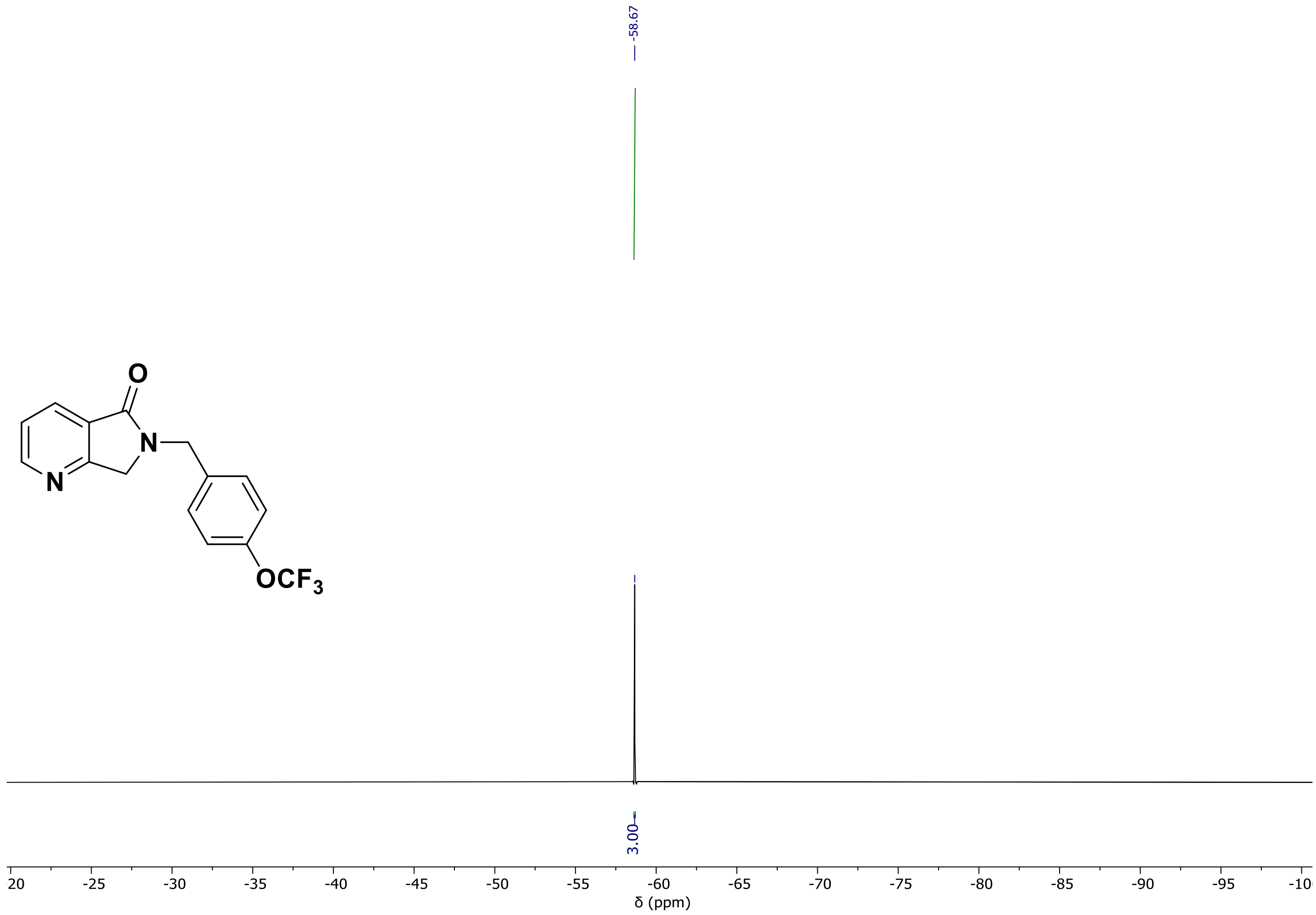

^{1}H NMR (Compound **10**)

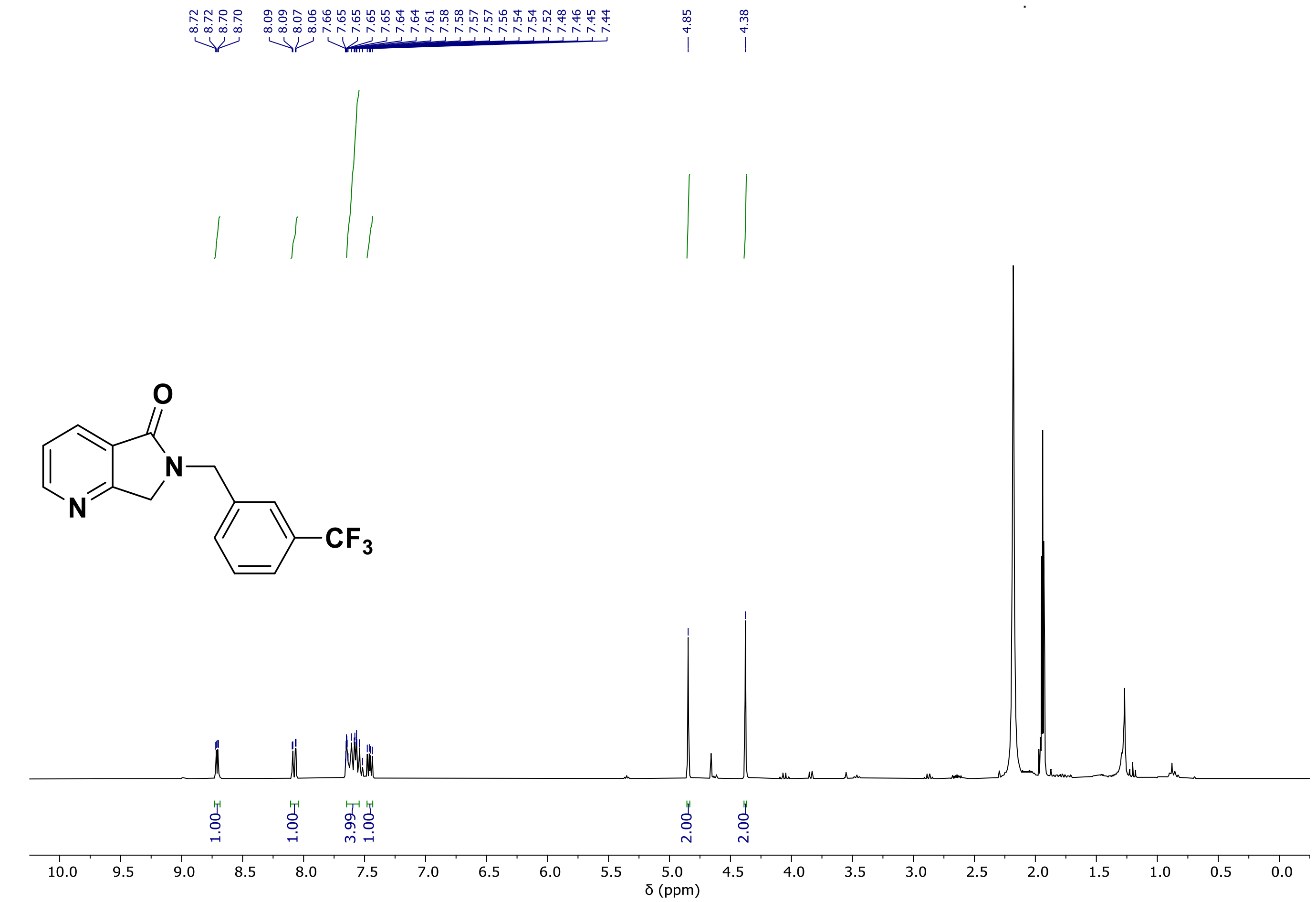

^{13}C NMR (Compound **10**)

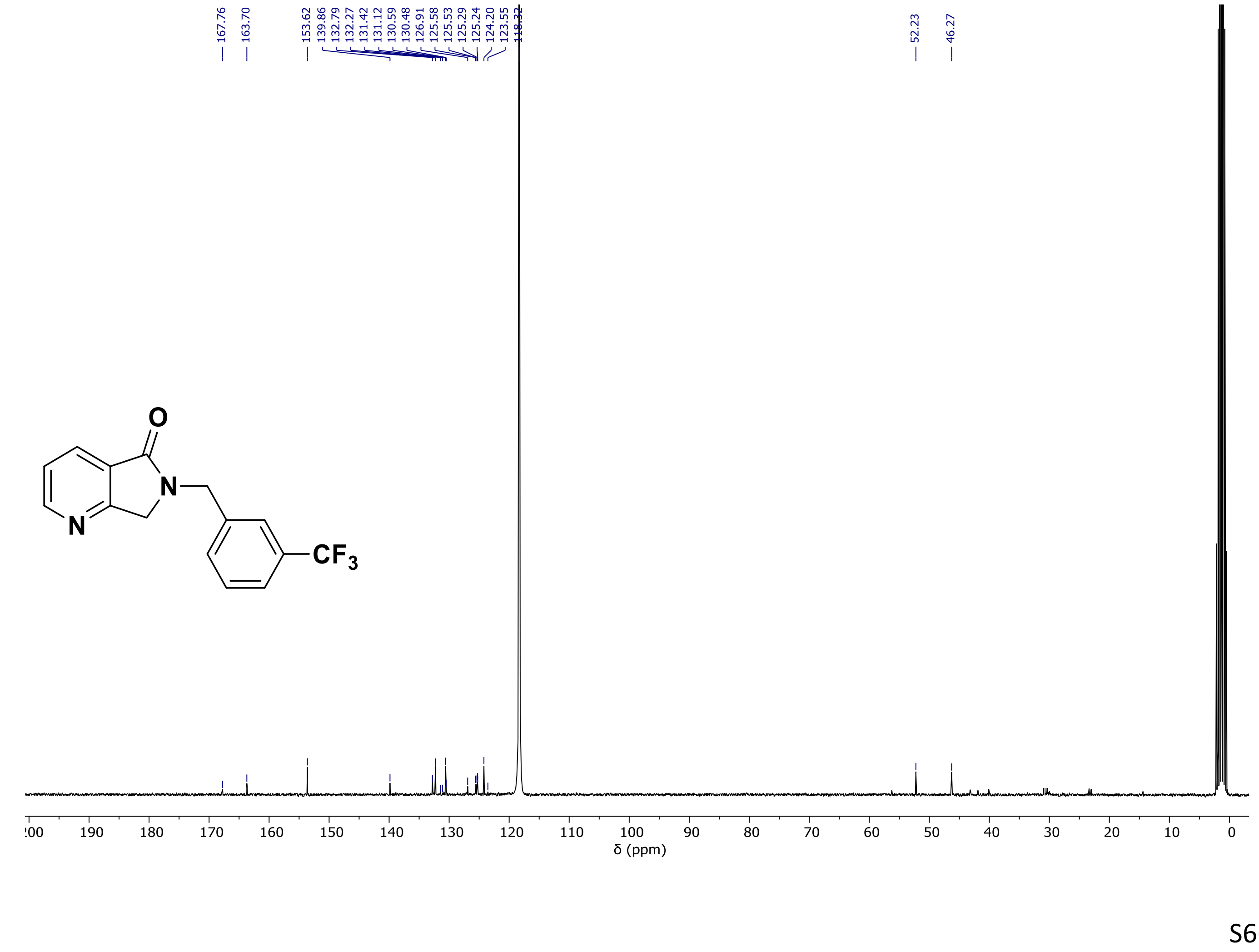

^{19}F NMR (Compound **10**)

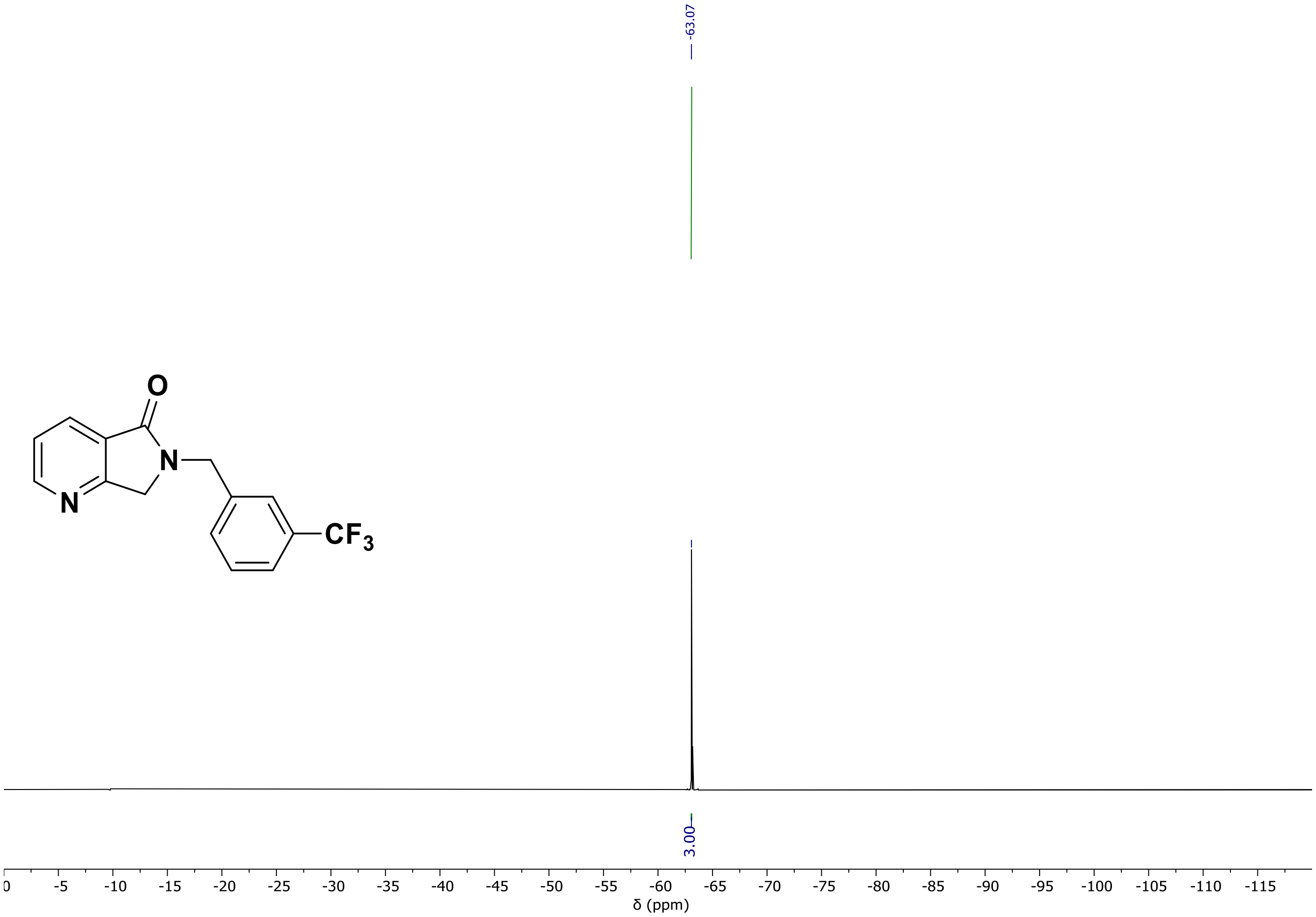

^{1}H NMR (Compound **11**)

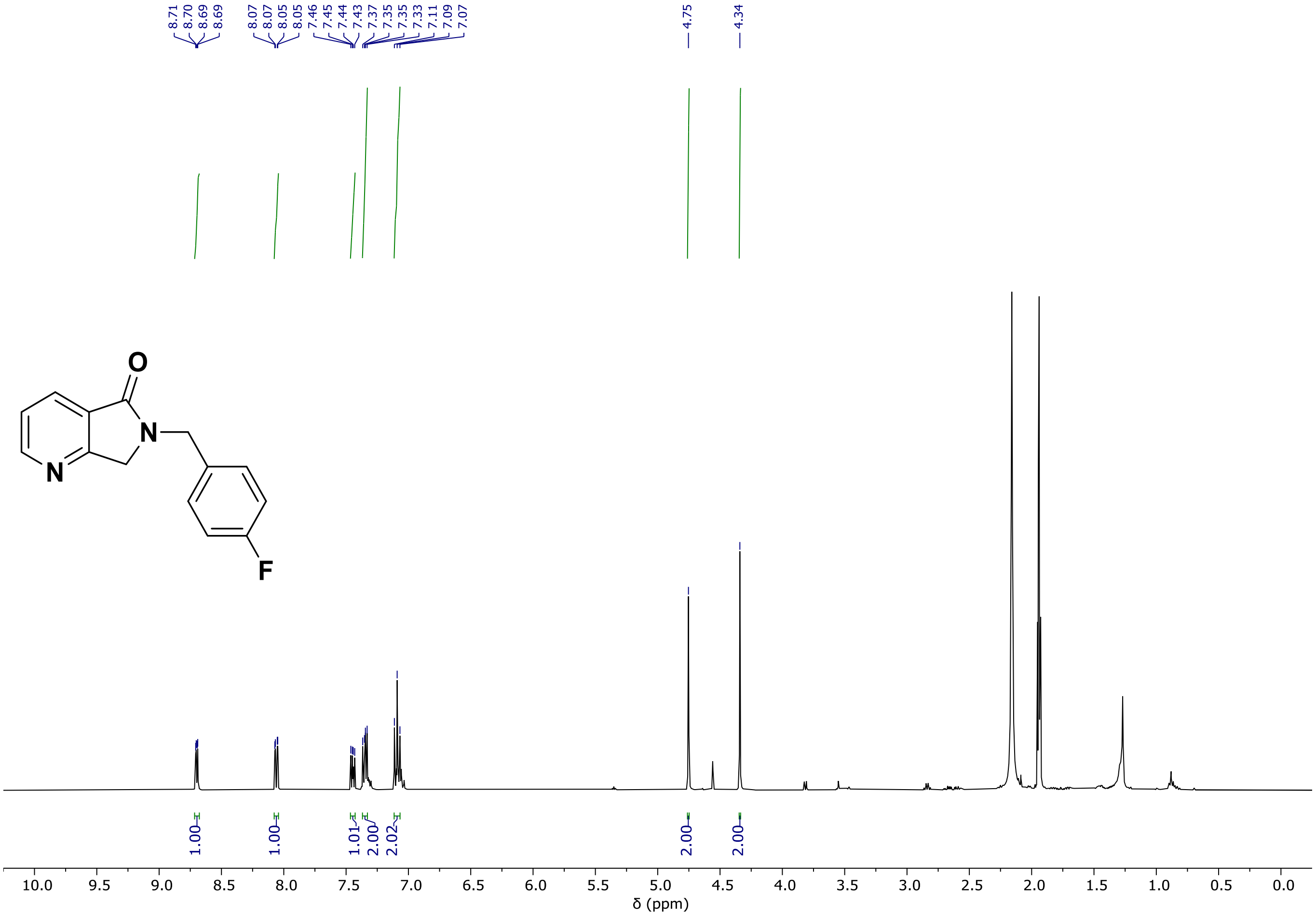

^{13}C NMR (Compound **11**)

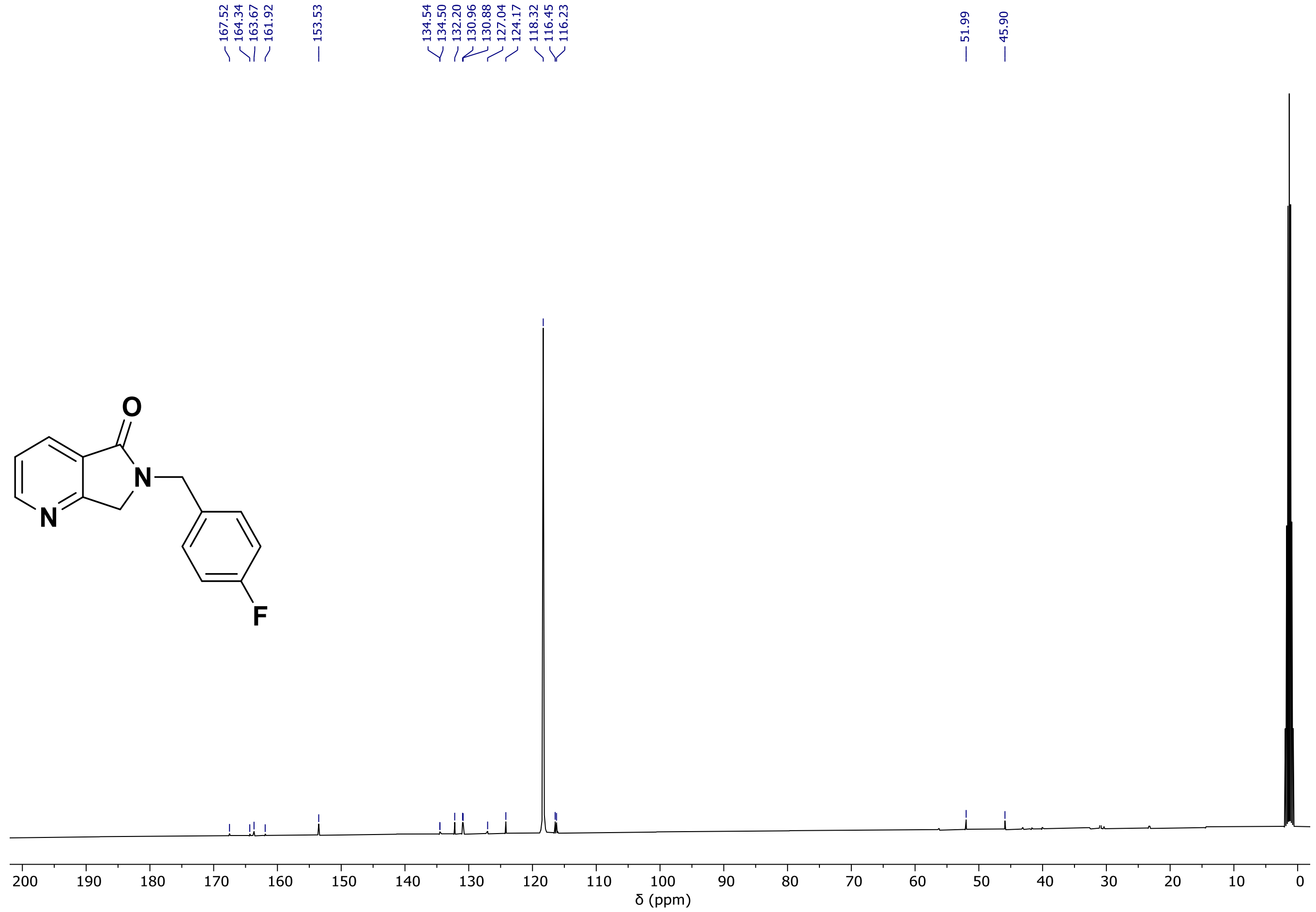

^{19}F NMR (Compound **11**)

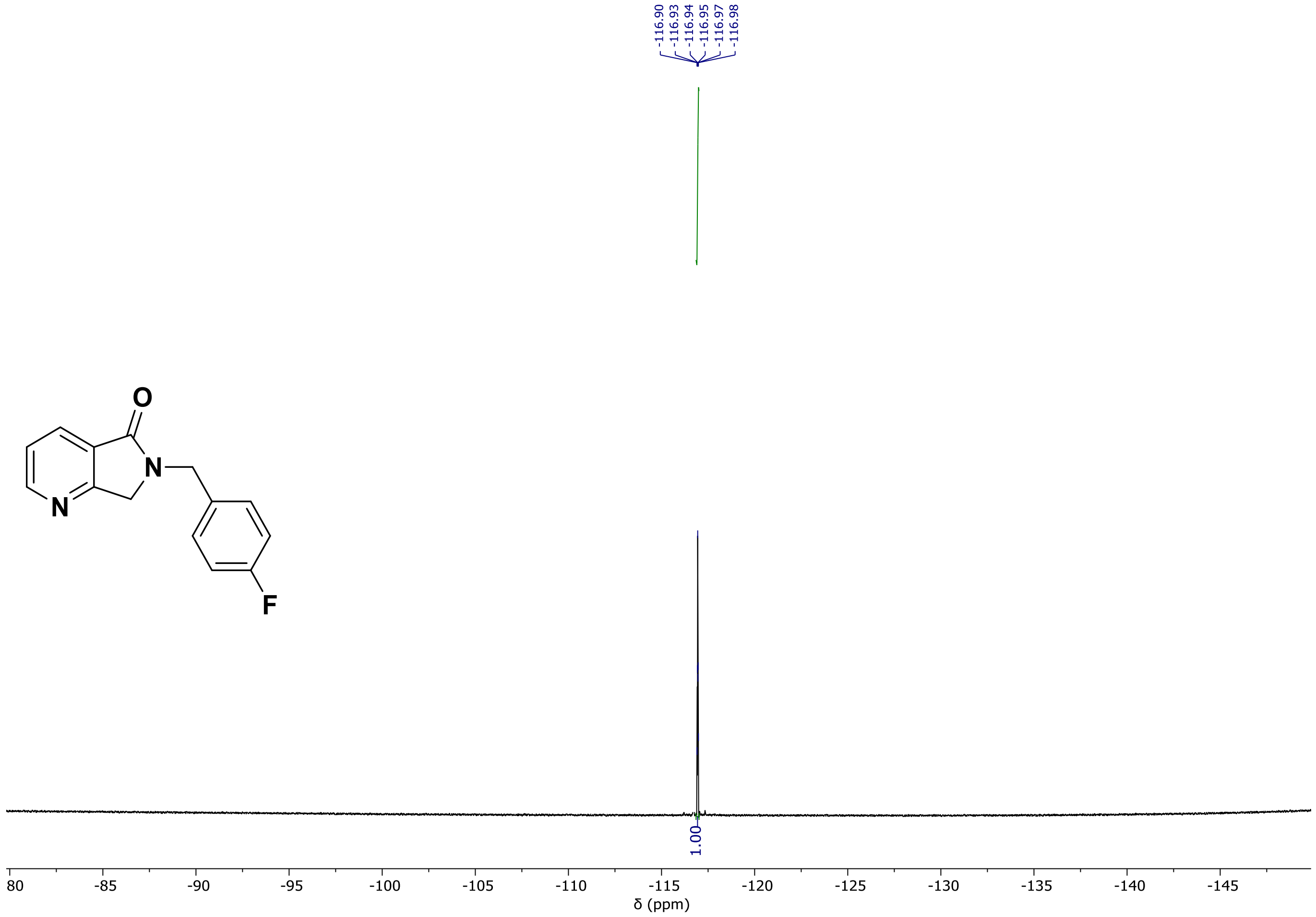

^{1}H NMR (Compound **12**)

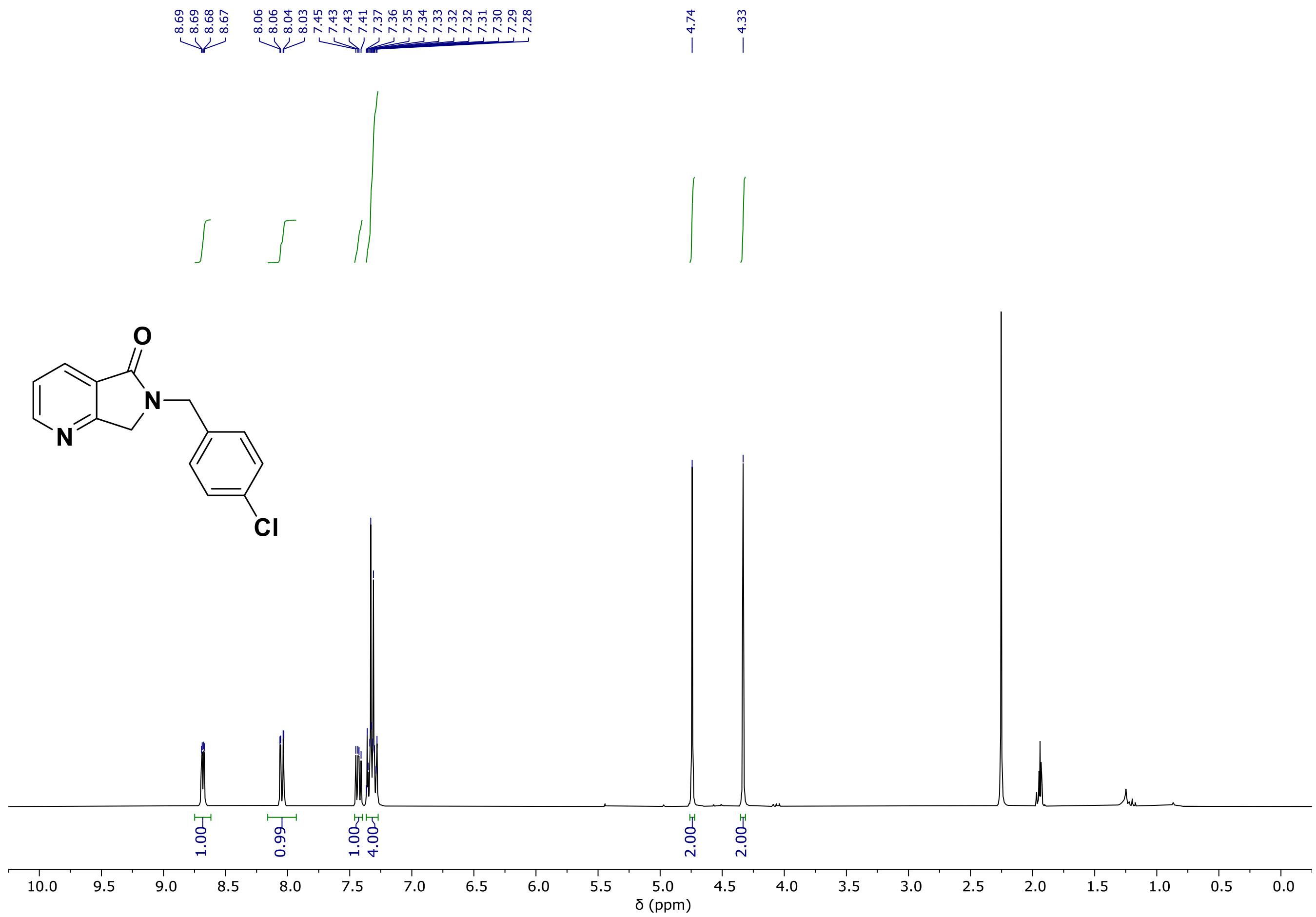

^{13}C NMR (Compound **12**)

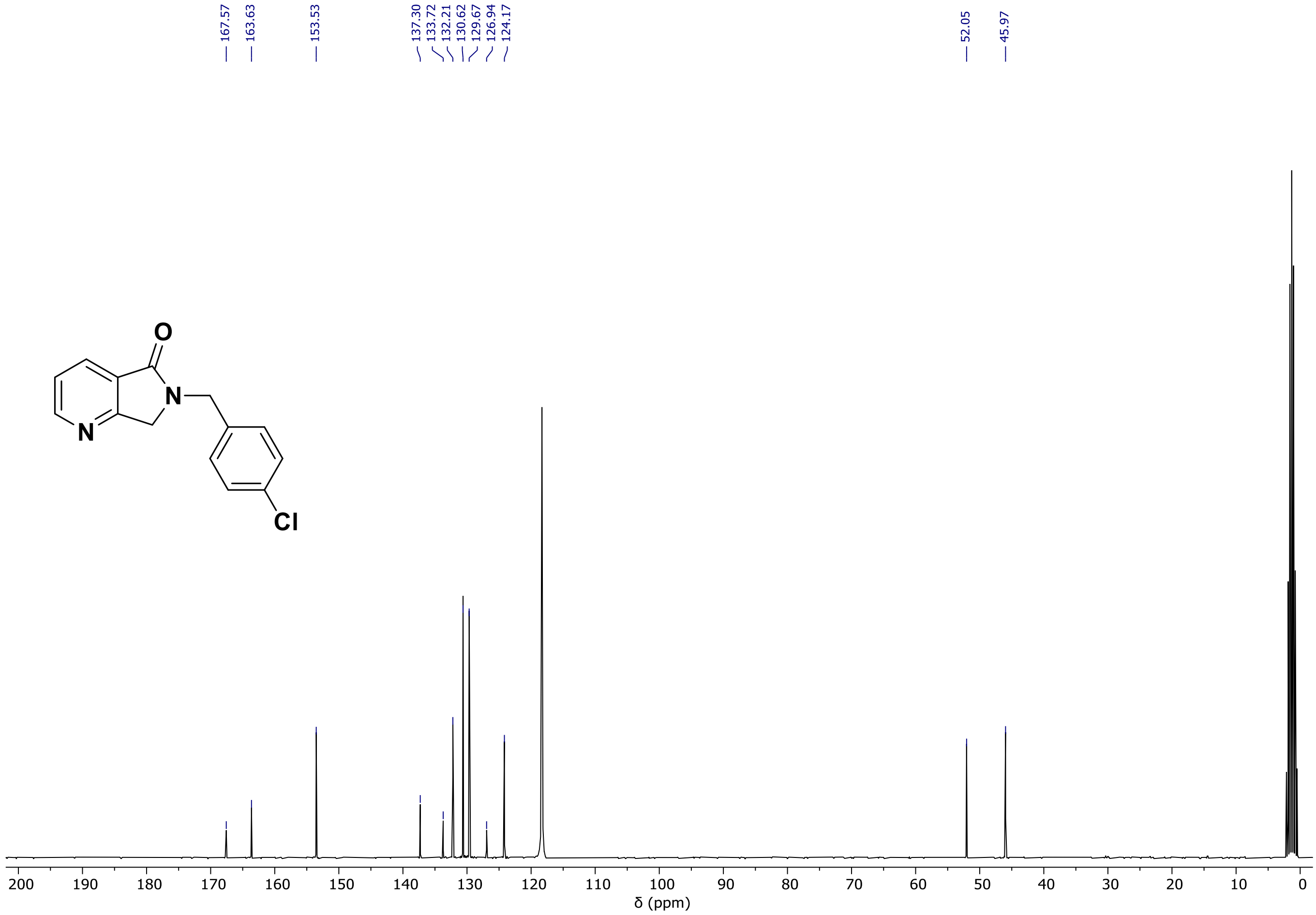

^{1}H NMR (Compound **13**)

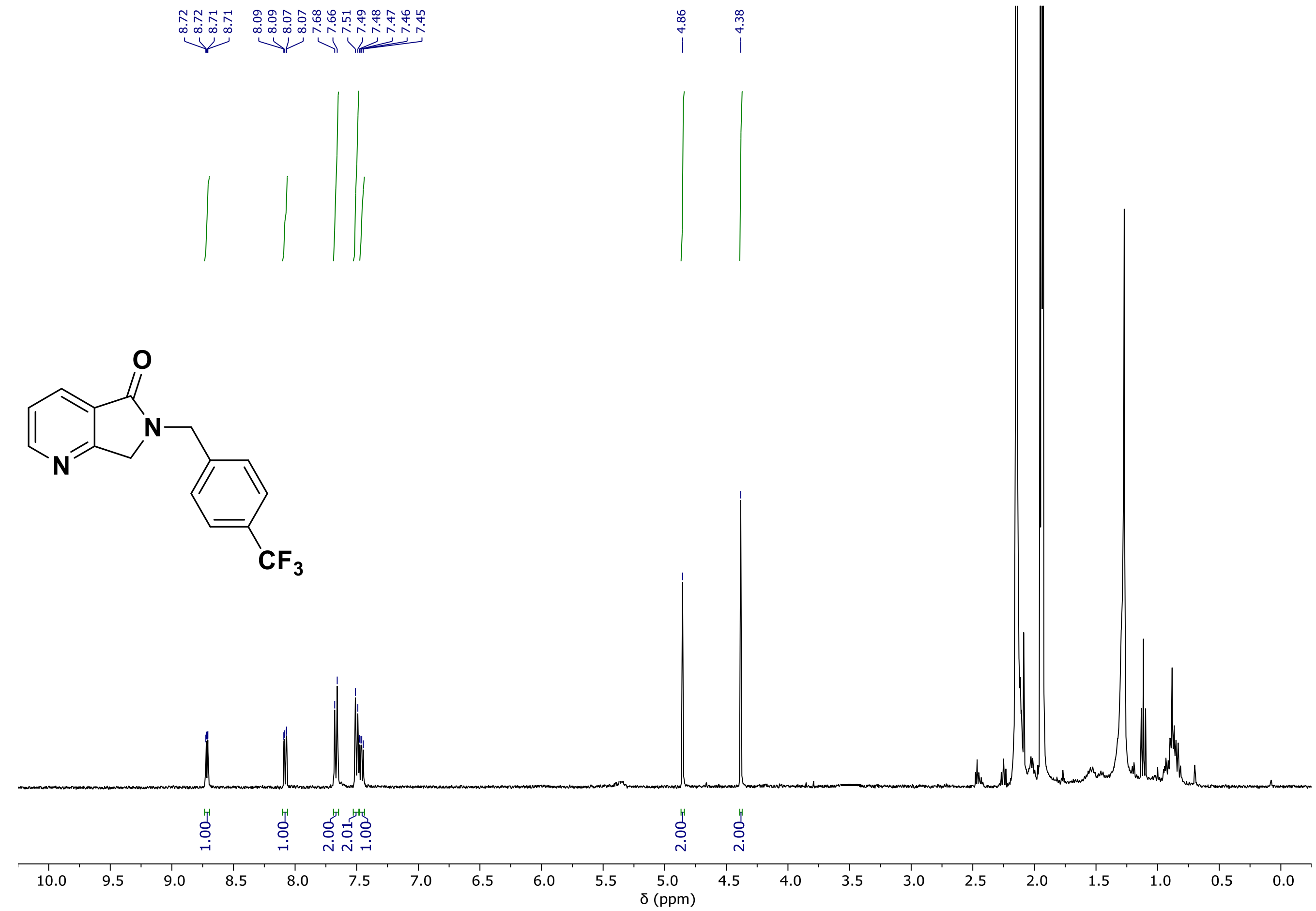

^{13}C NMR (Compound **13**)

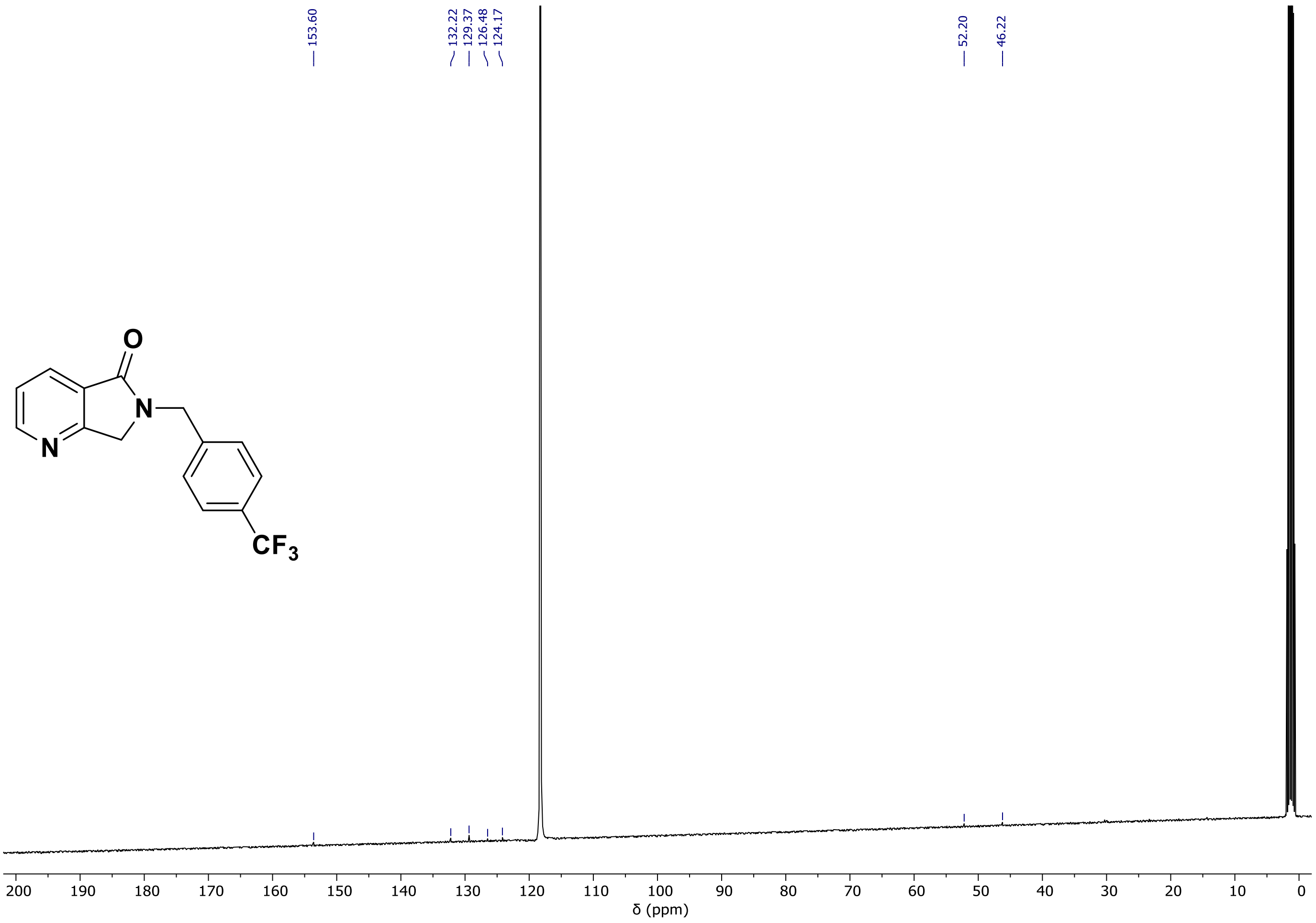

^{19}F NMR (Compound **13**)

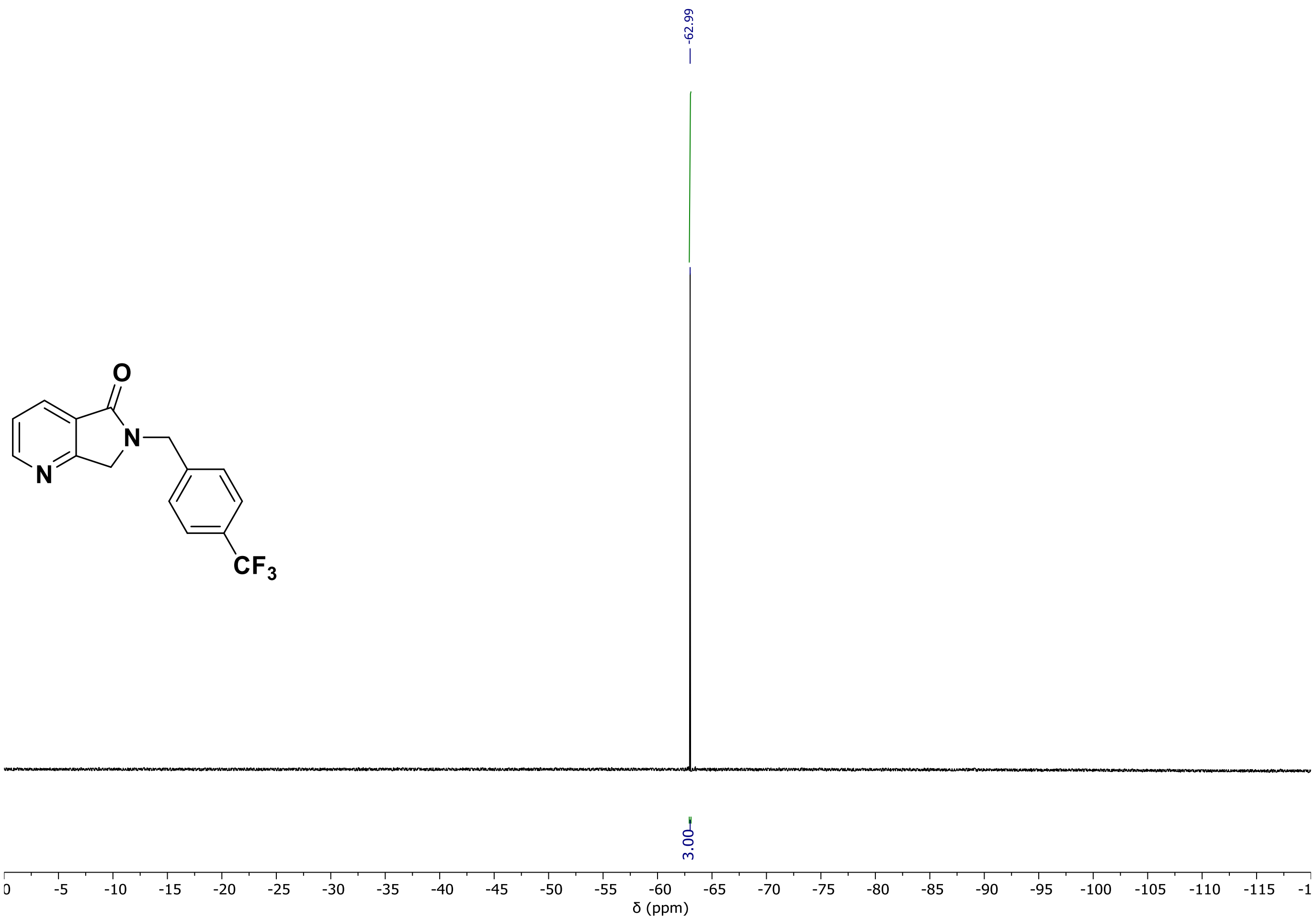

^{1}H NMR (Compound **14**)

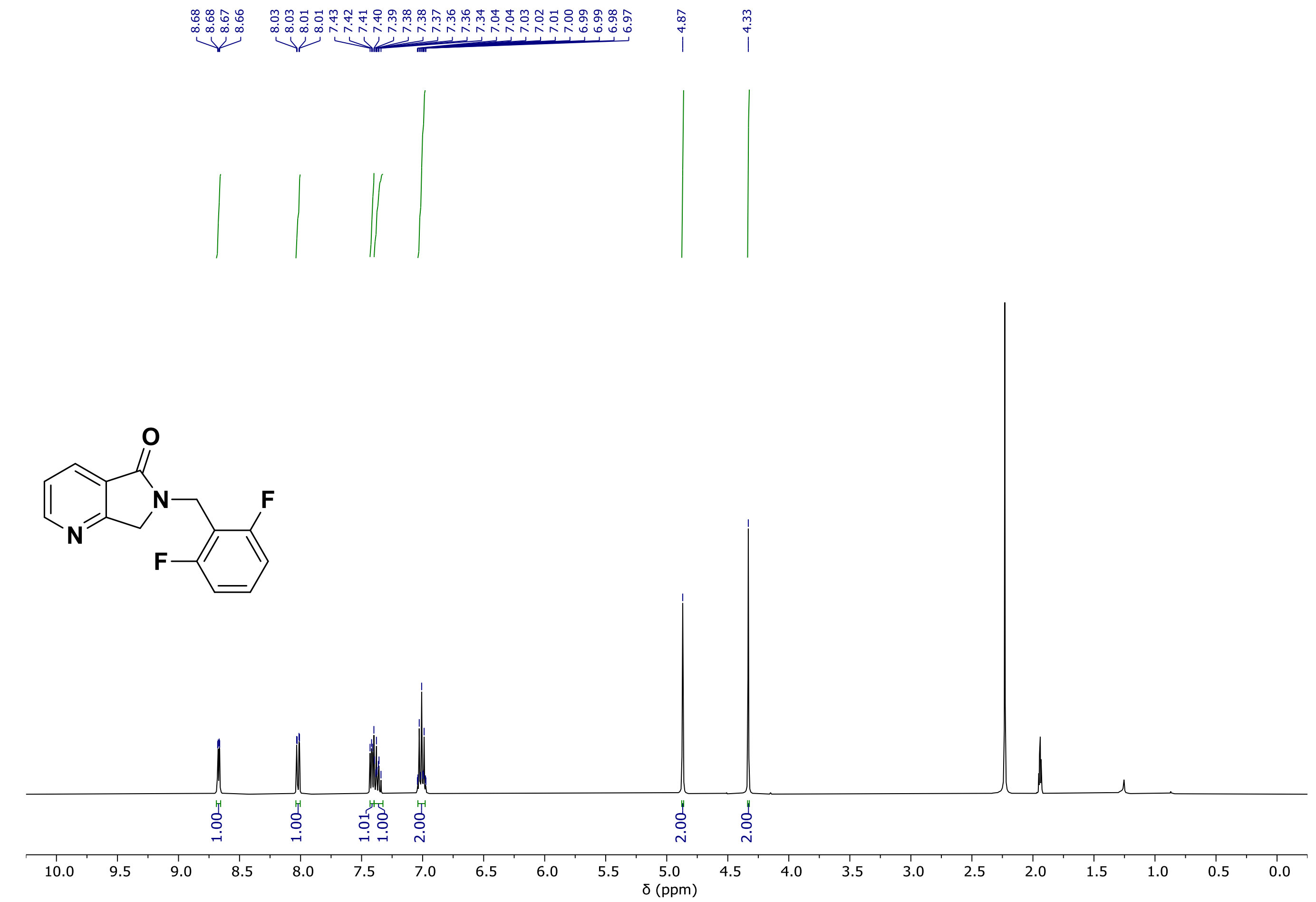

^{13}C NMR (Compound **14**)

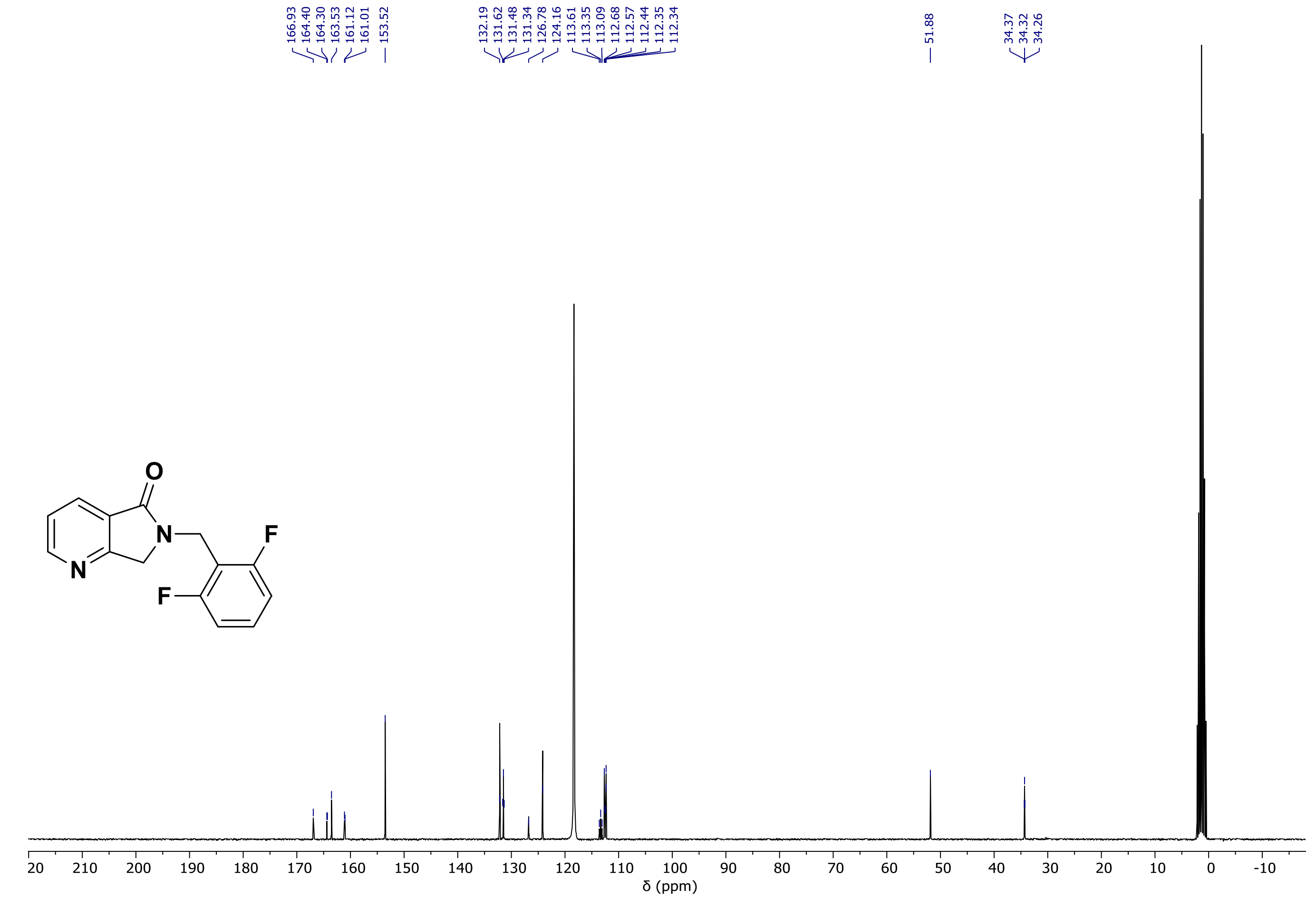

^{19}F NMR (Compound **14**)

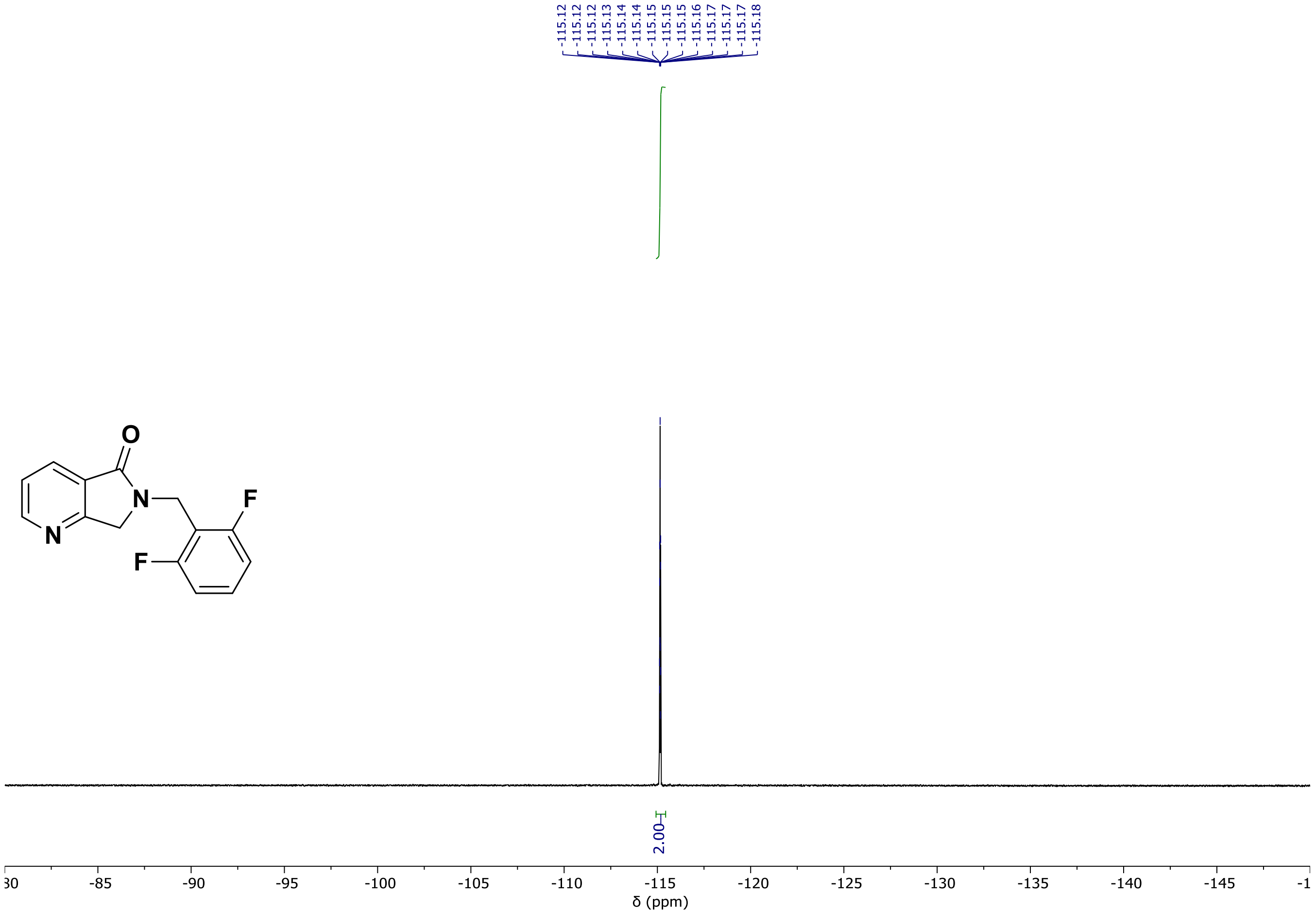

^{1}H NMR (Compound **15**)

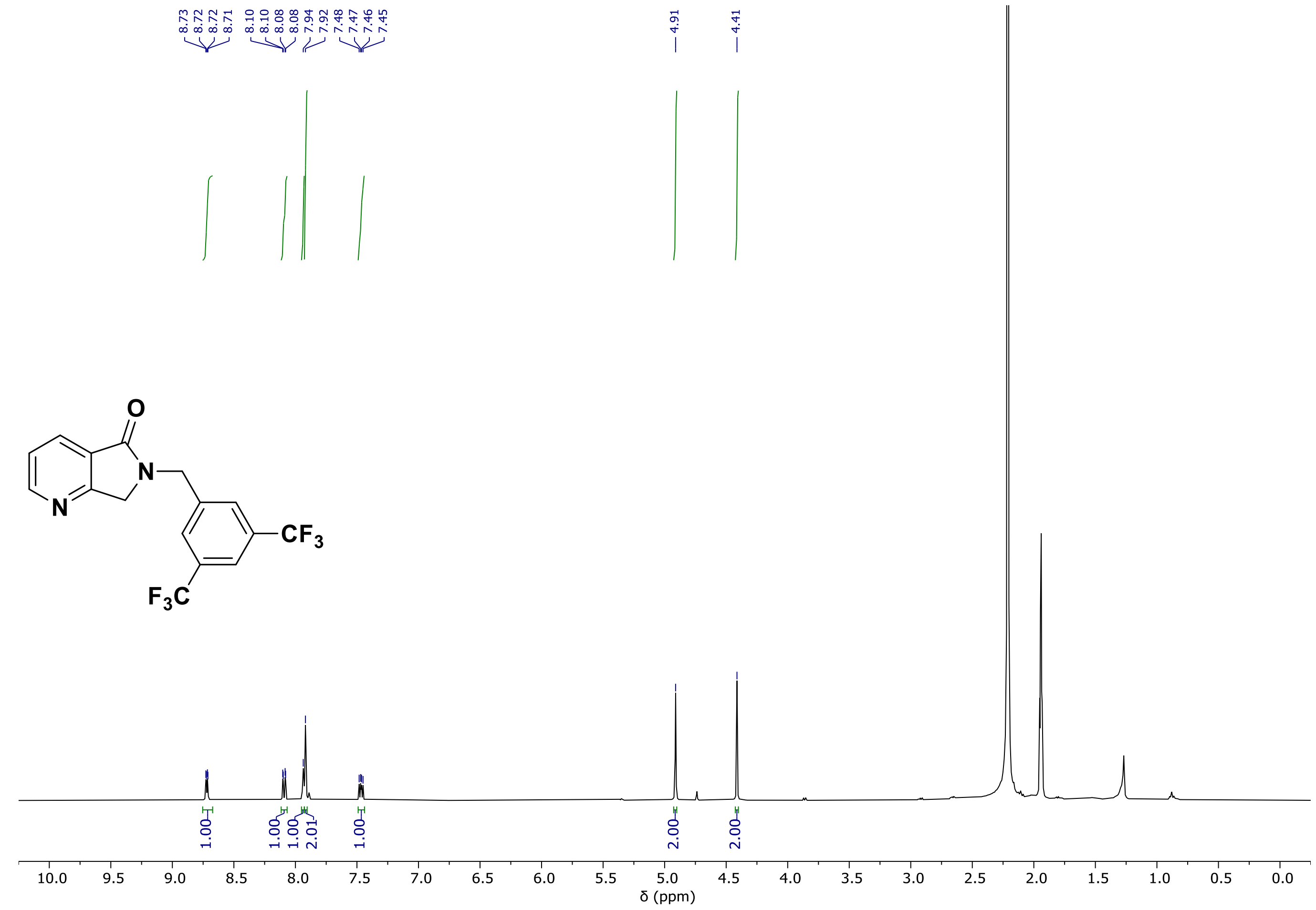

^{13}C NMR (Compound **15**)

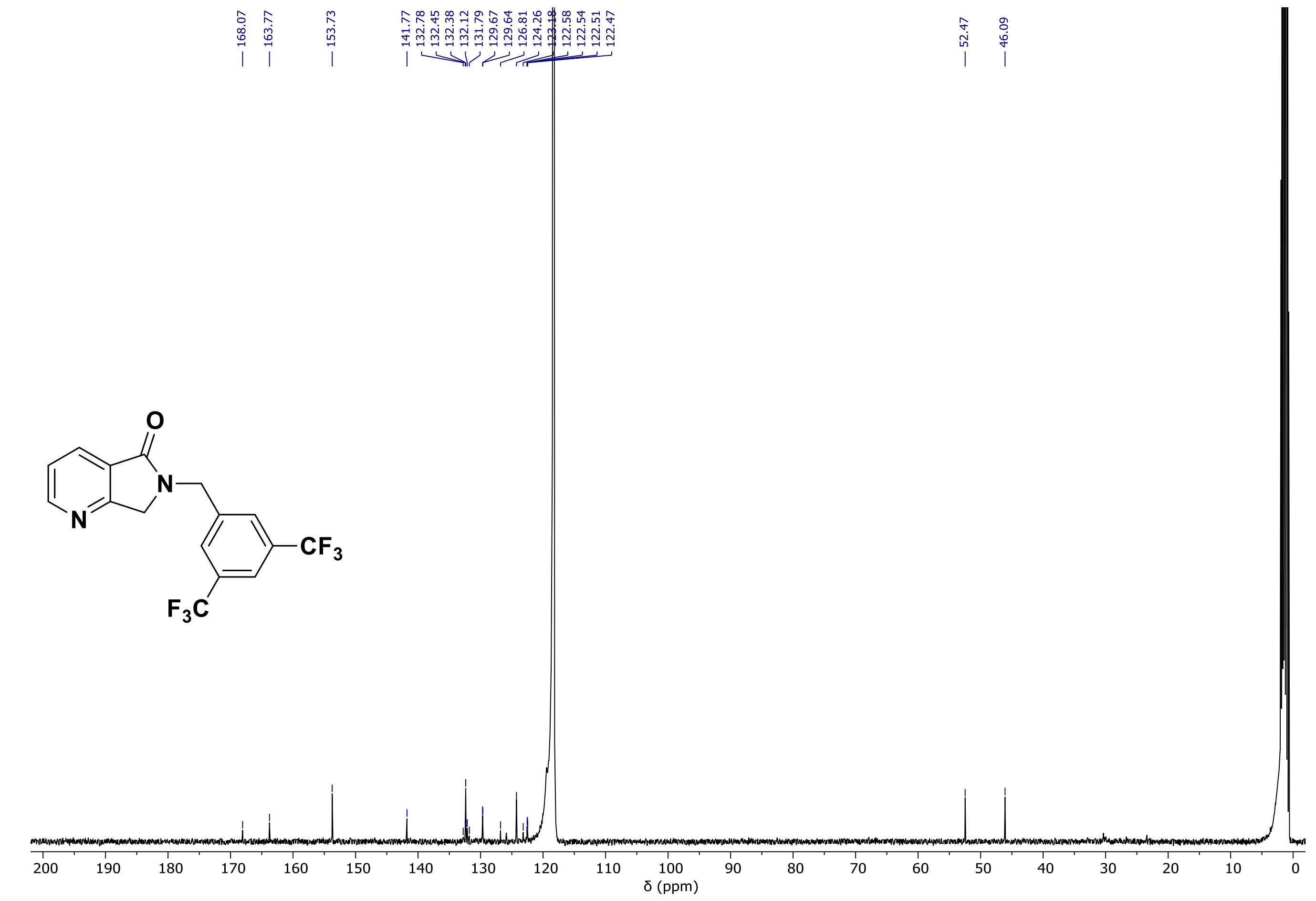

^{19}F NMR (Compound **15**)

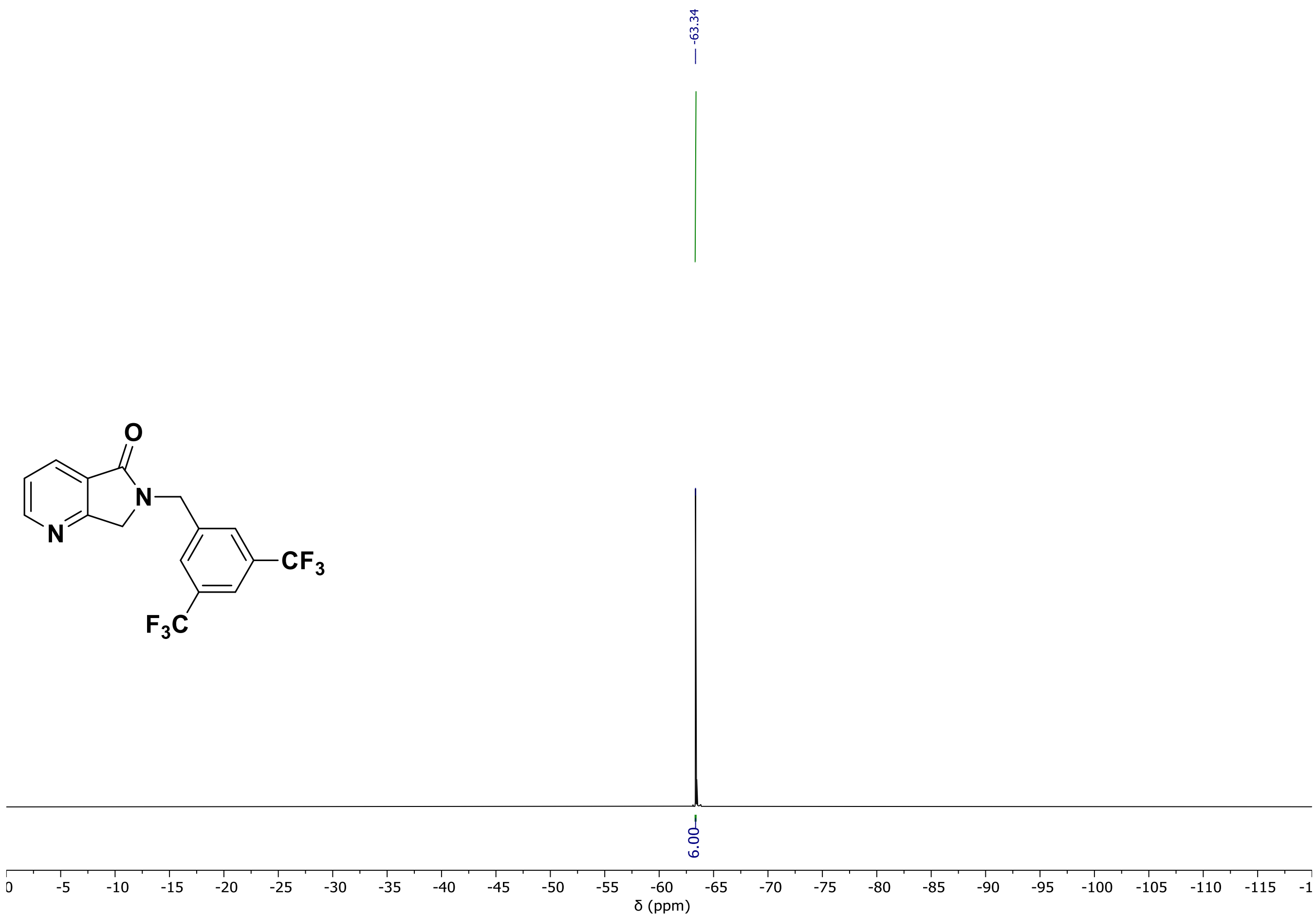

^{1}H NMR (Compound **16**)

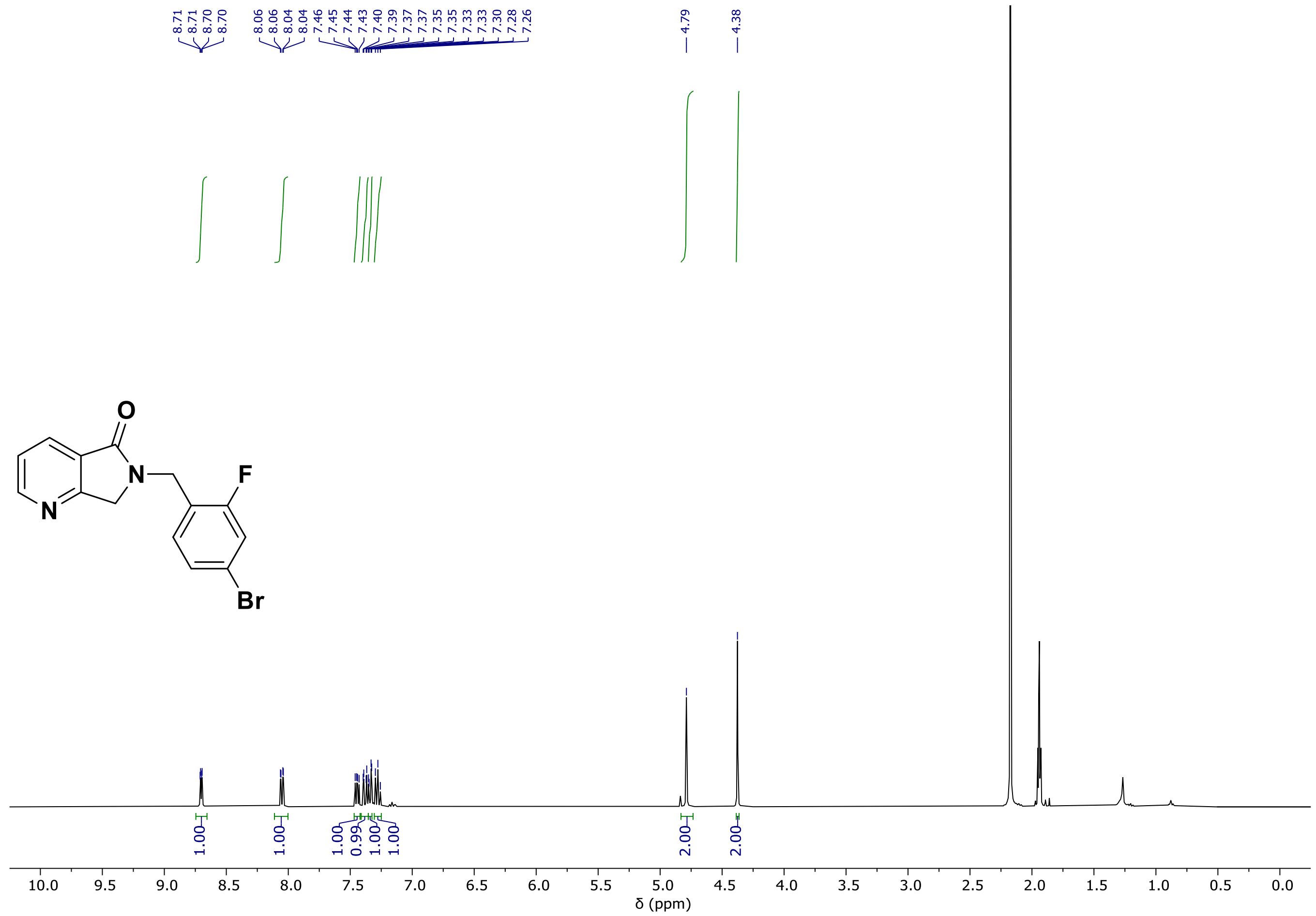

^{13}C NMR (Compound **16**)

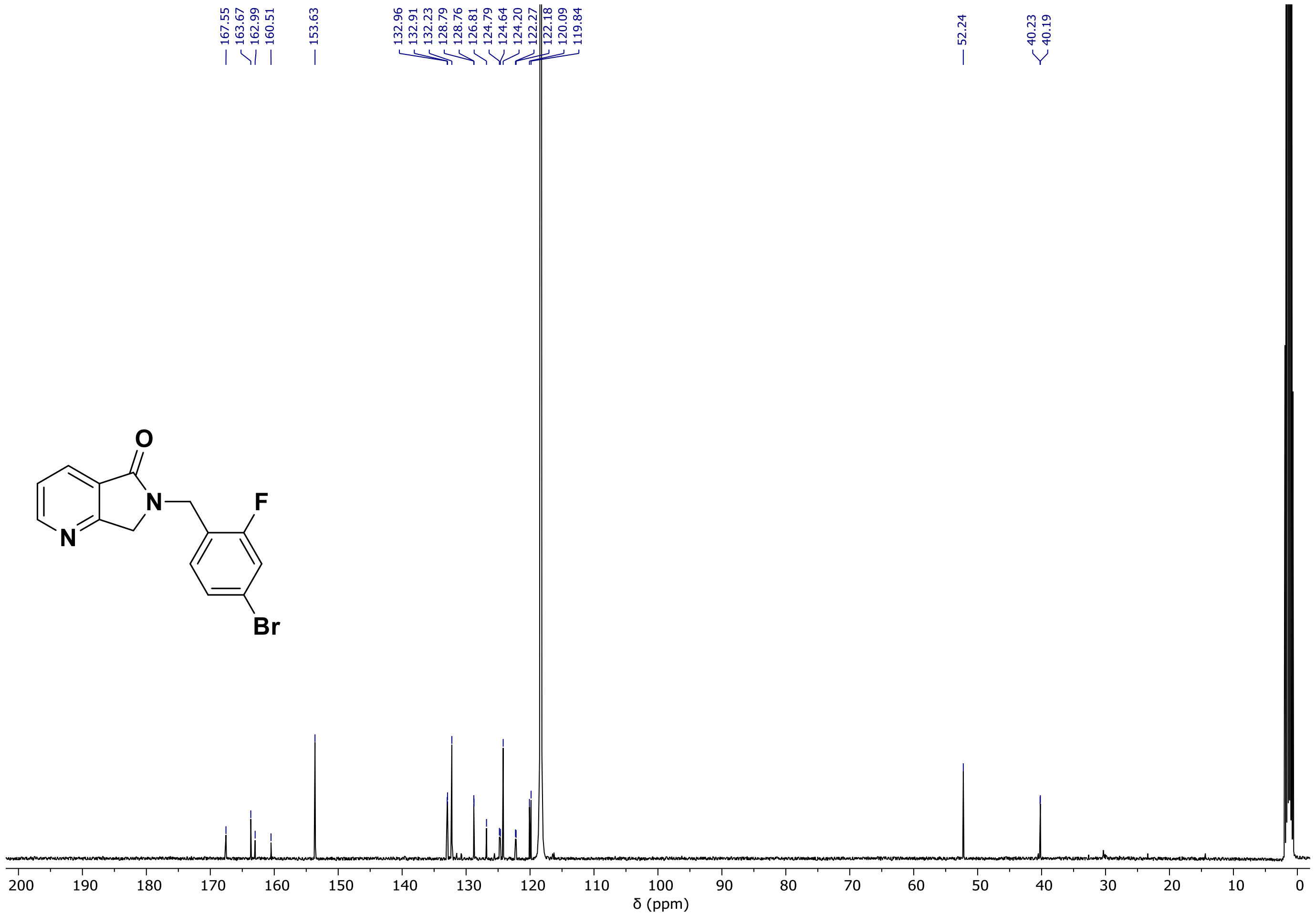

^{19}F NMR (Compound **16**)

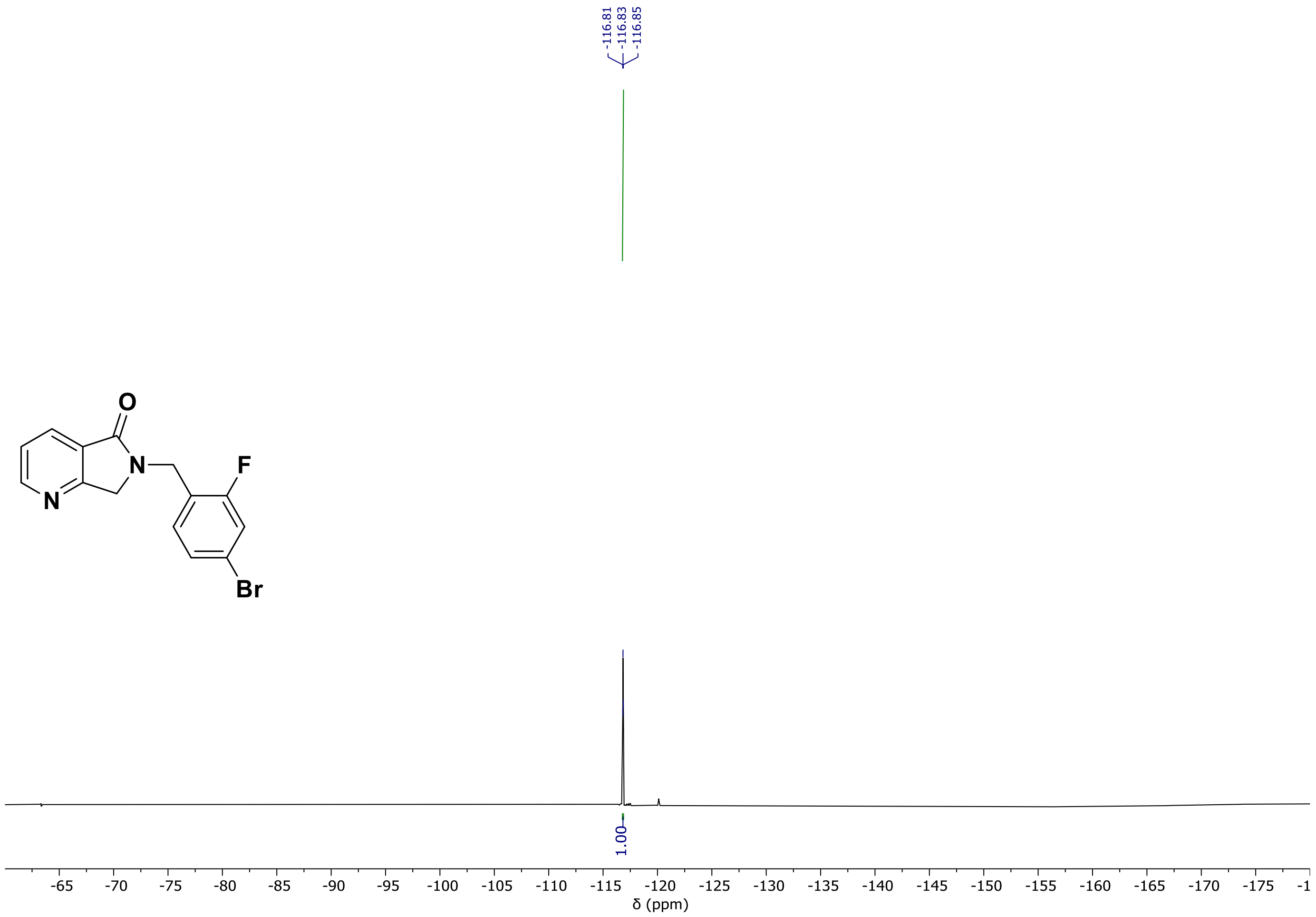

^{1}**H NMR** (Compound **17**)

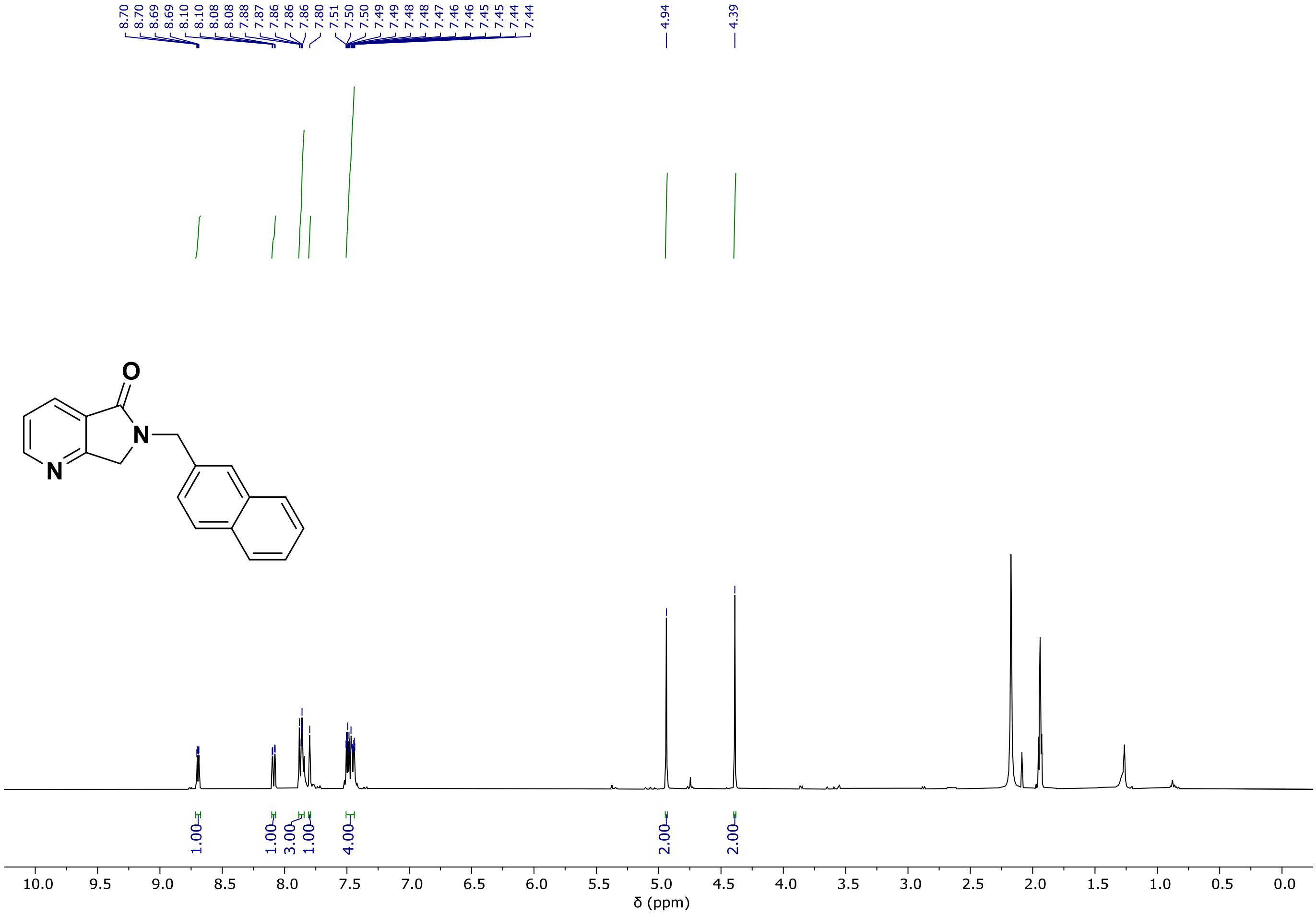

^{13}**C NMR** (Compound **17**)

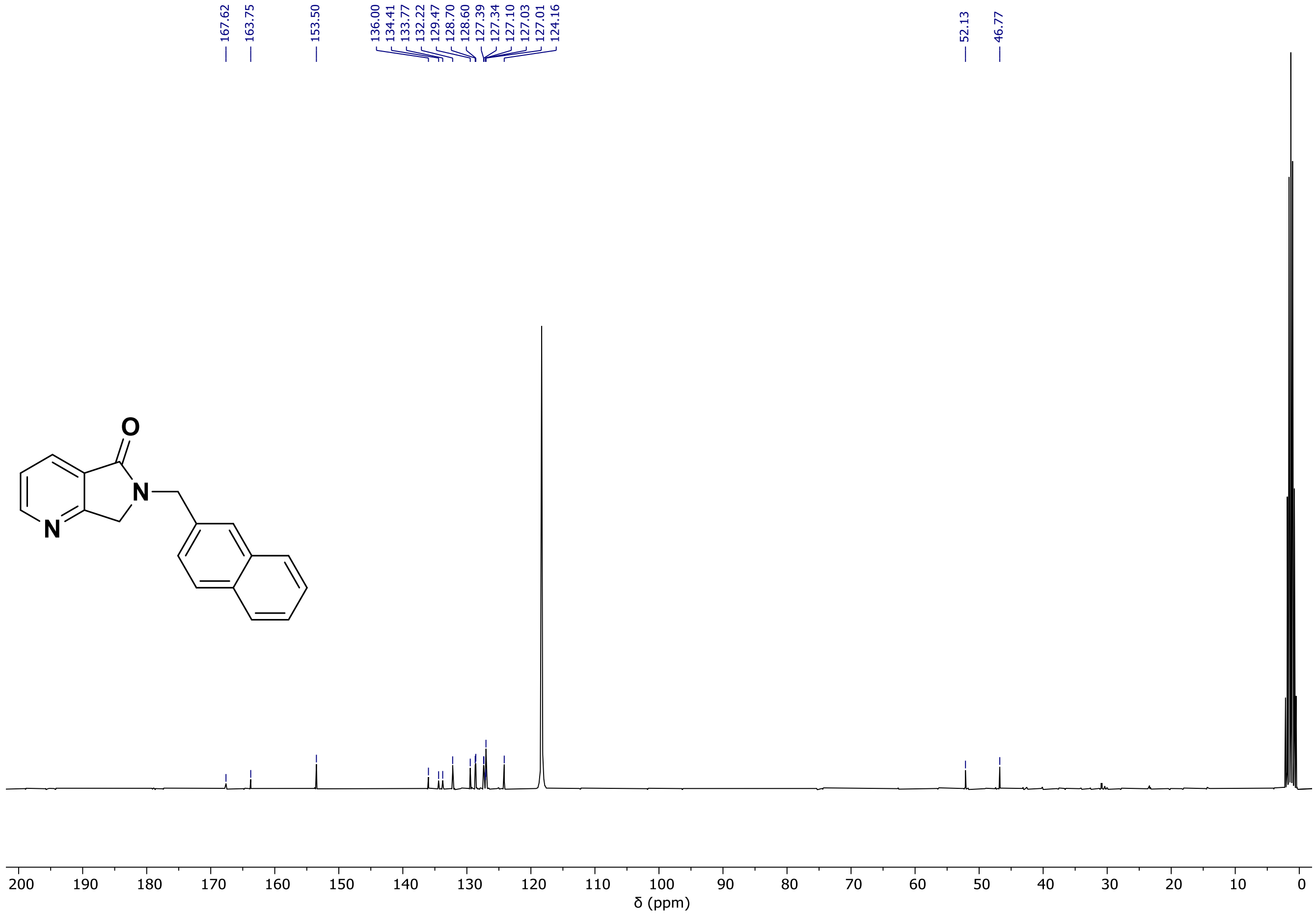

^{1}H NMR (Compound **18**)

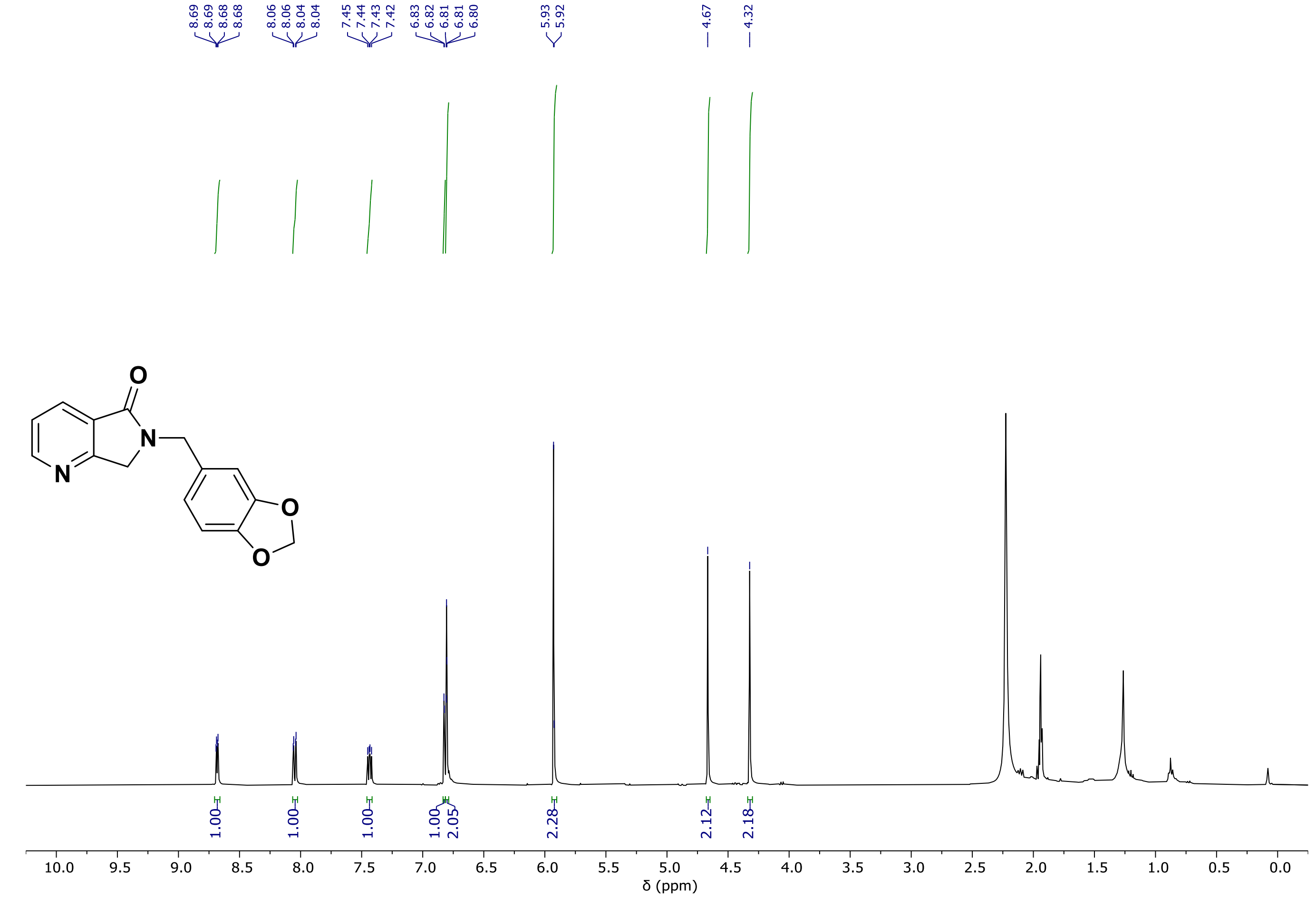

^{13}C NMR (Compound **18**)

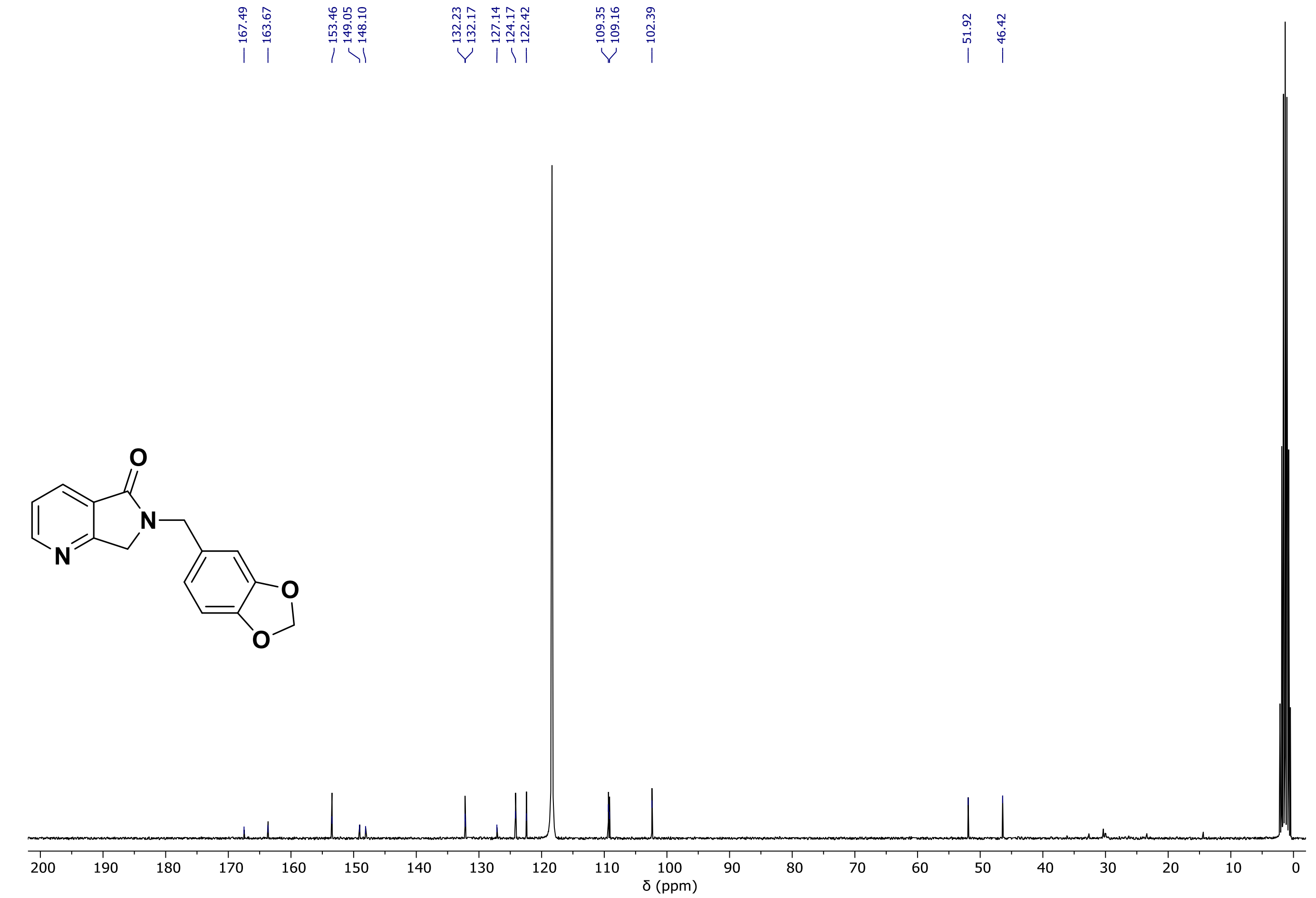

^{1}H NMR (Compound **19**)

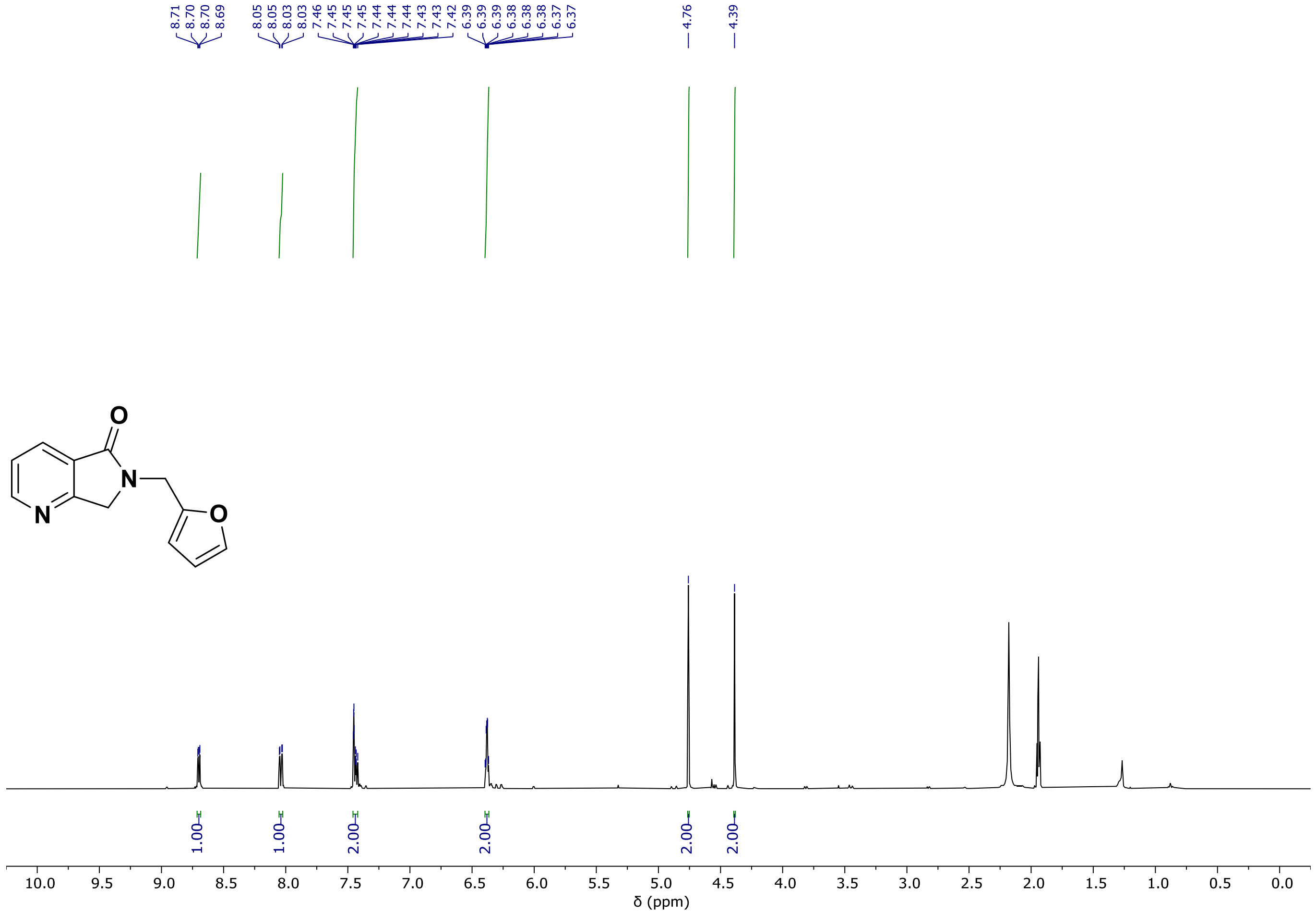

^{13}C NMR (Compound **18**)

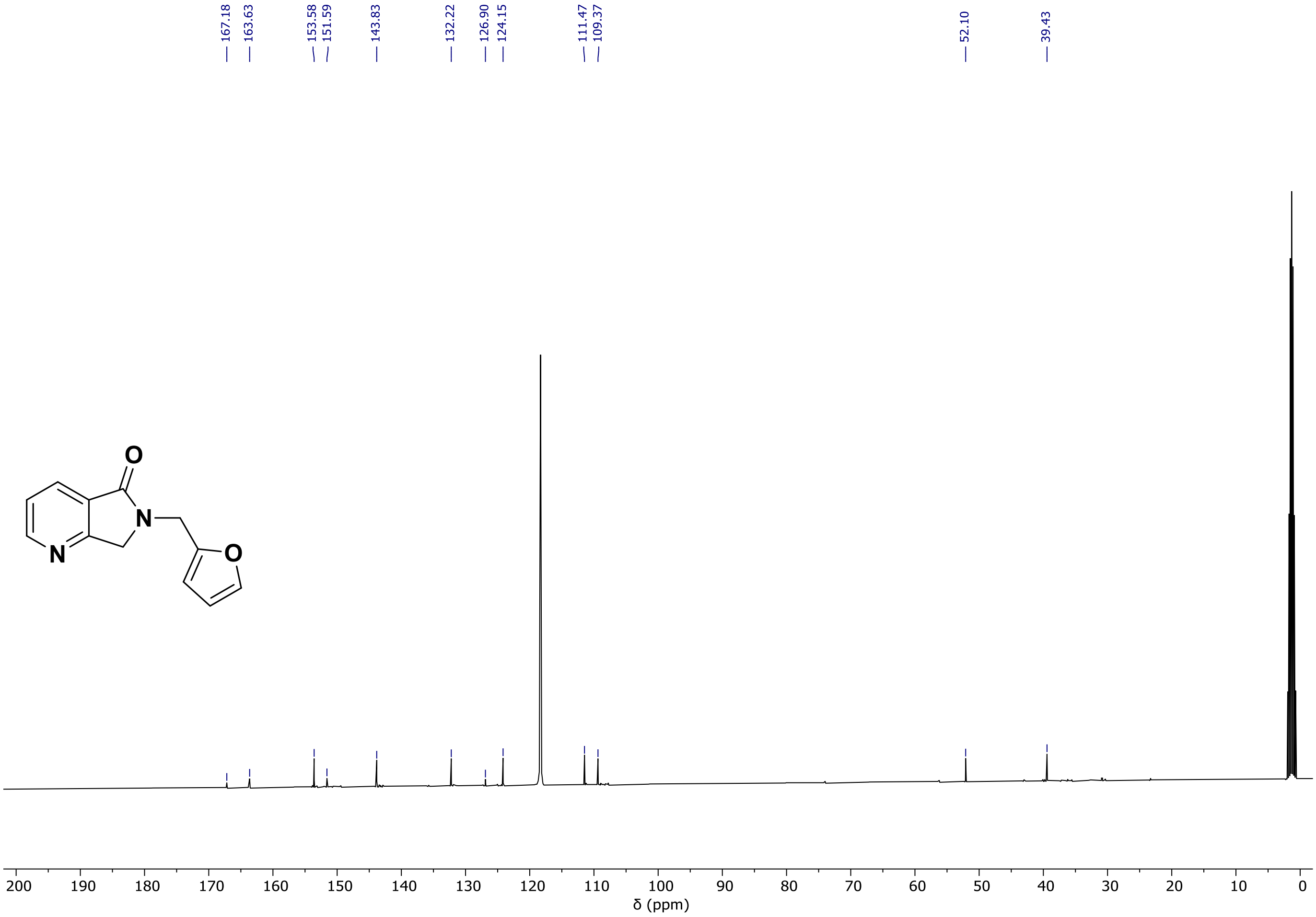

^{1}H NMR (Compound **20**)

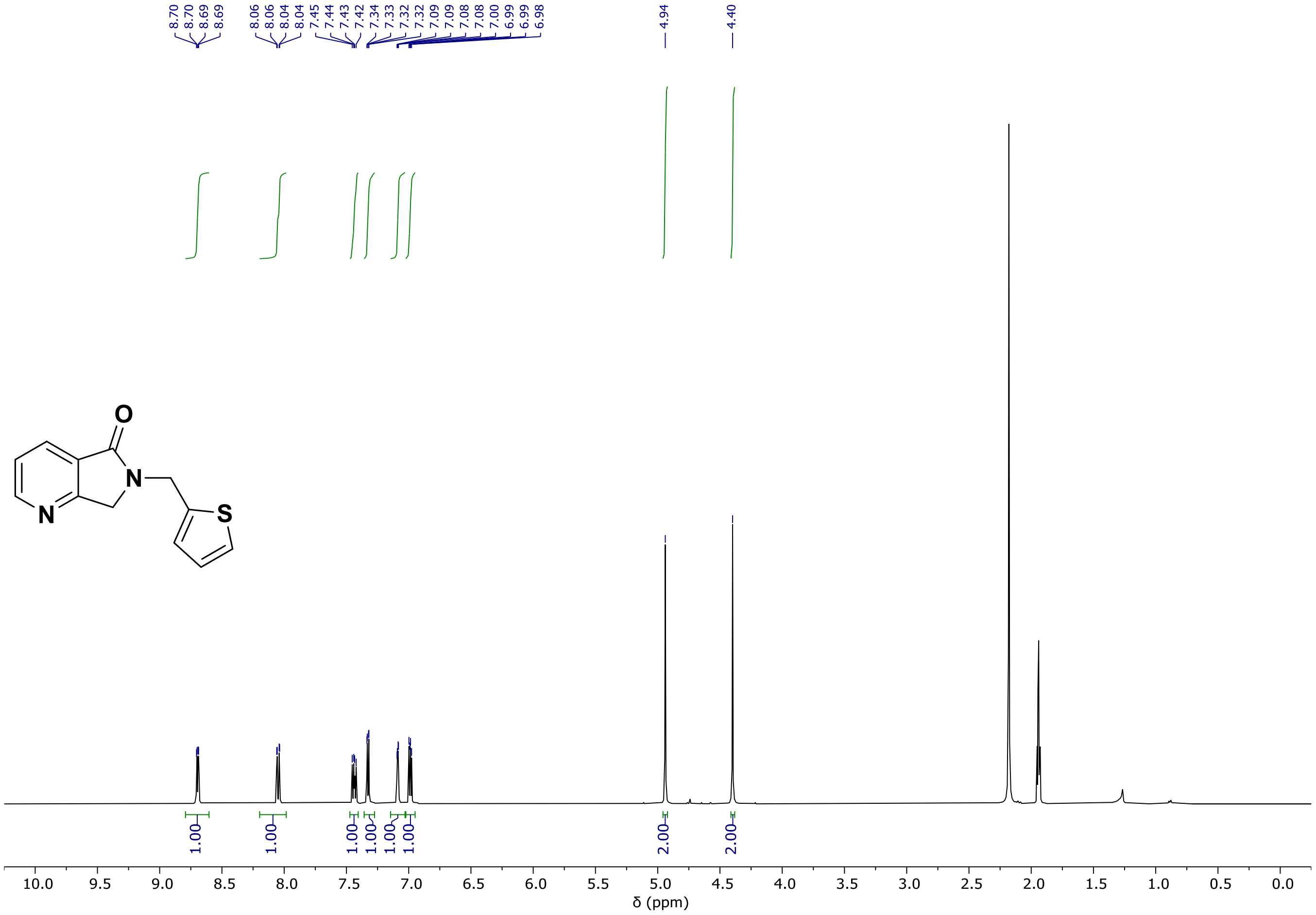

^{13}C NMR (Compound **20**)

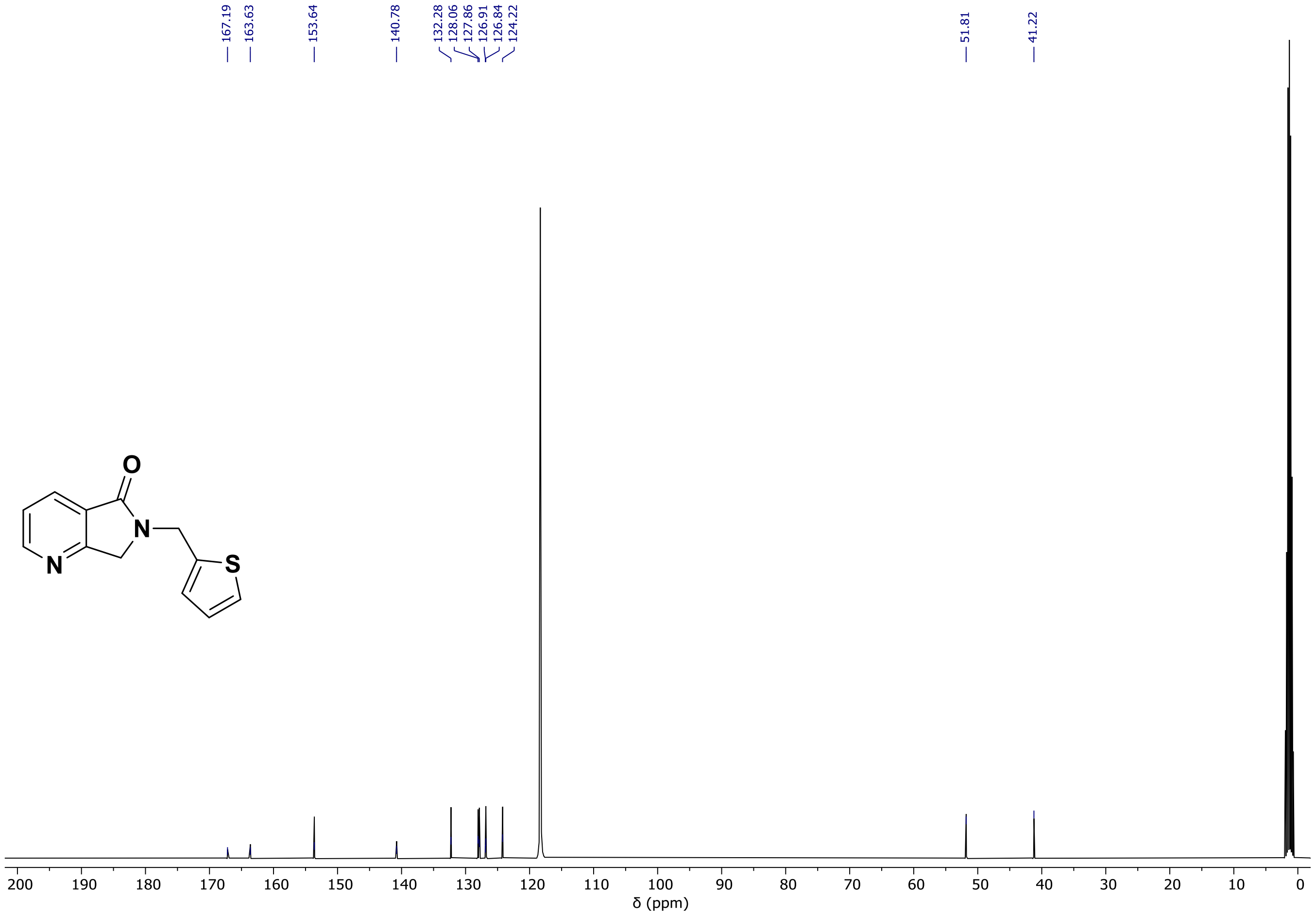

^{1}H NMR (Compound **22**)

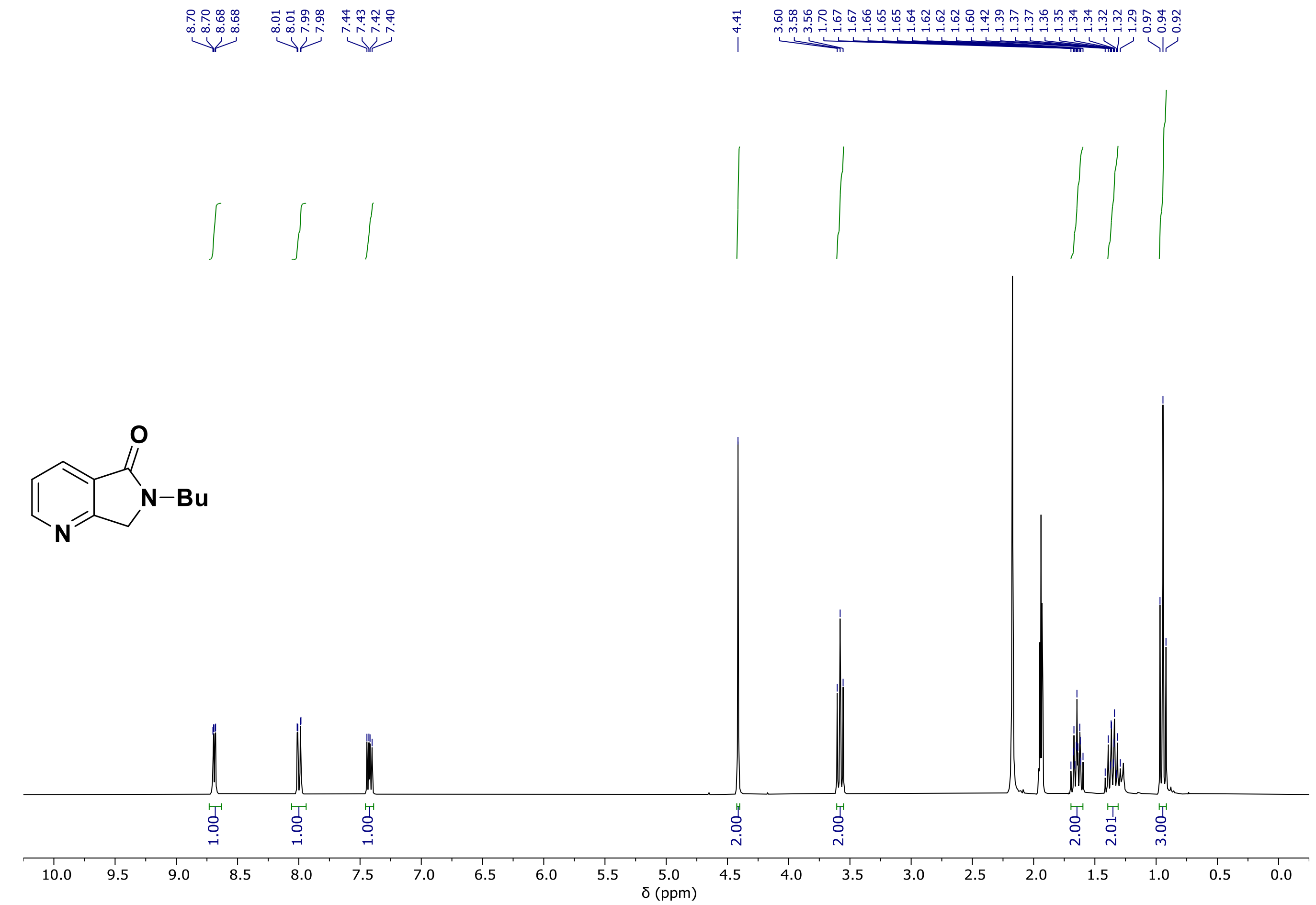

^{13}C NMR (Compound **22**)

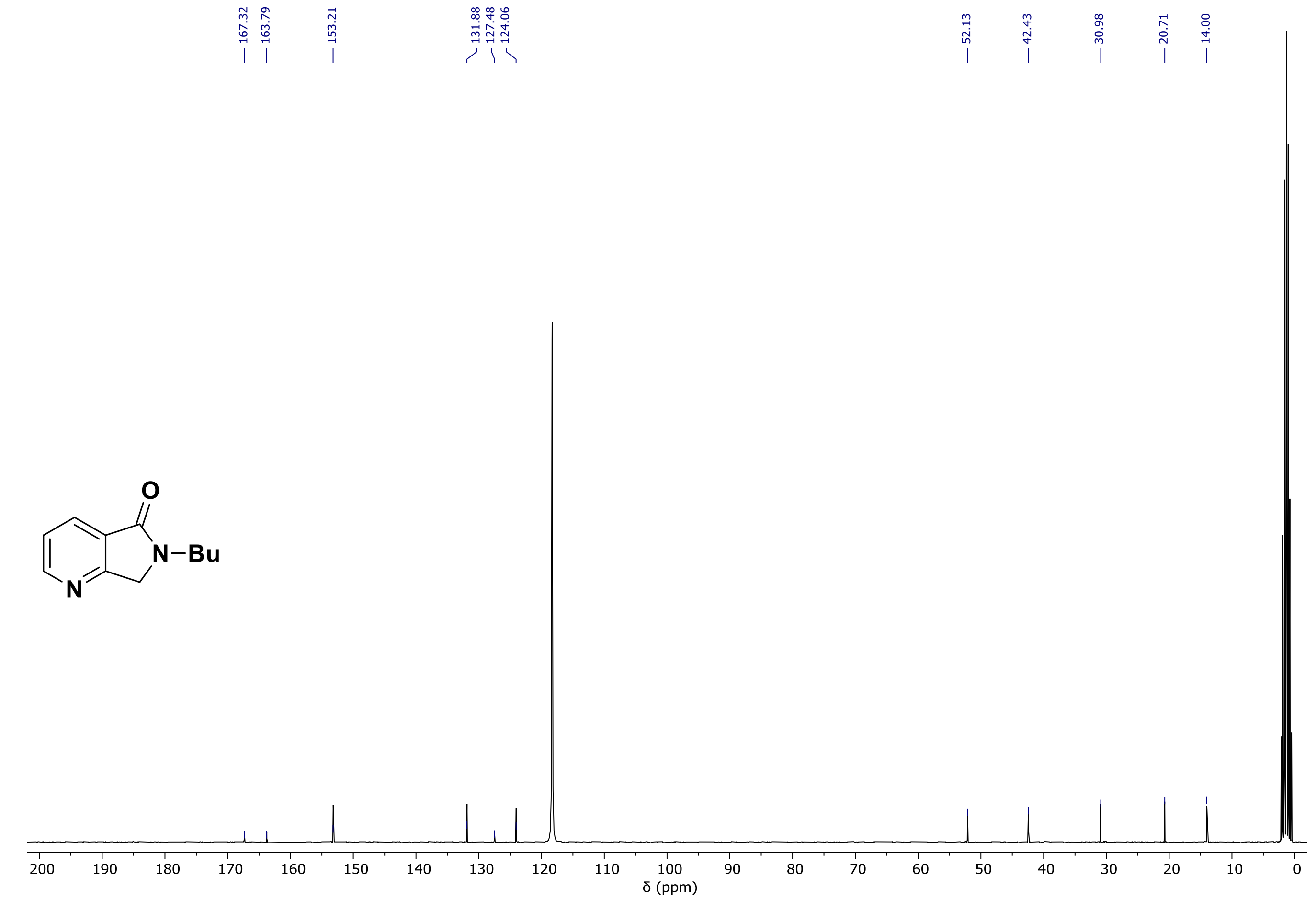

^{1}H NMR (Compound **23**)

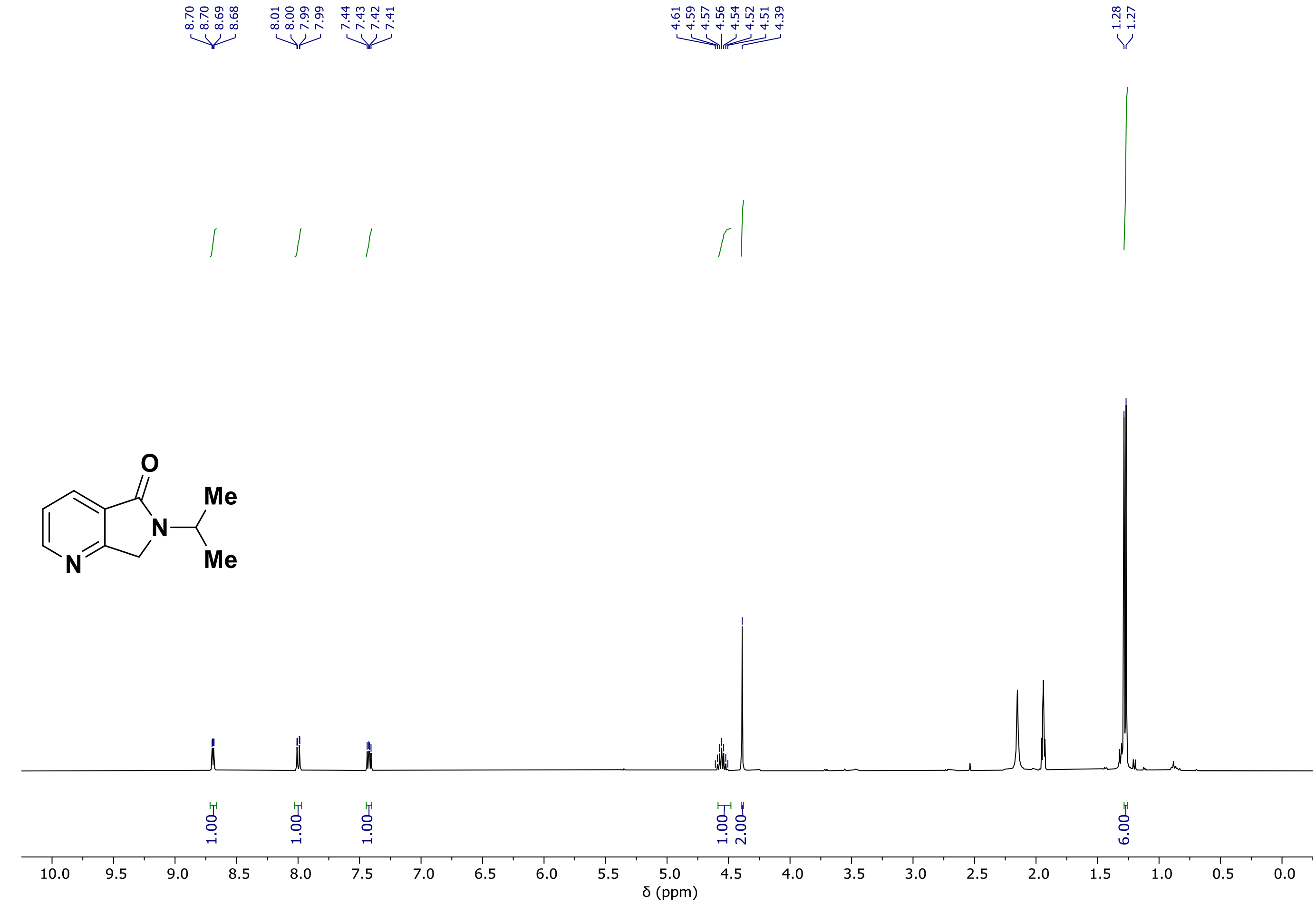

^{13}C NMR (Compound **23**)

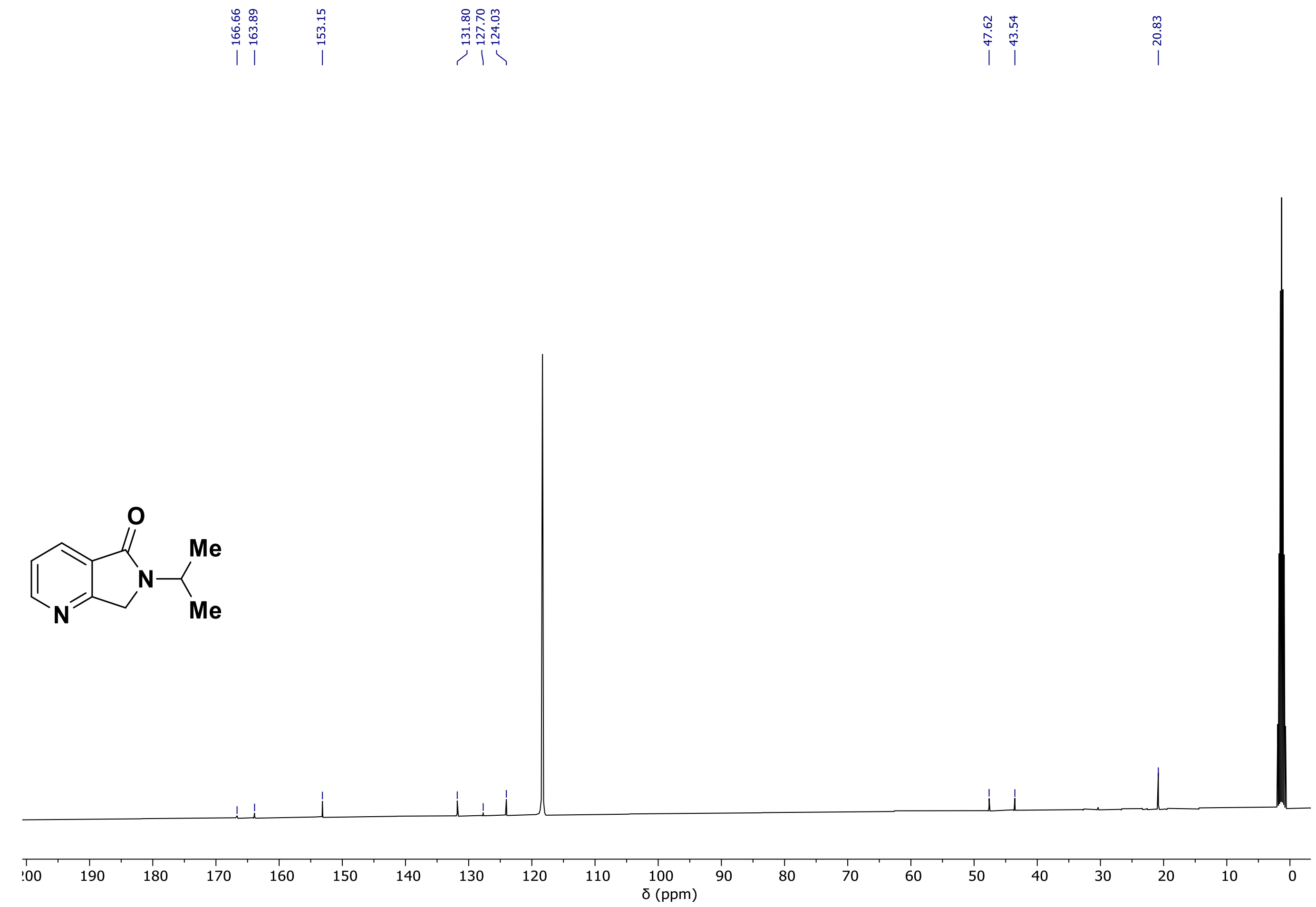

^{1}H NMR (Compound **24**)

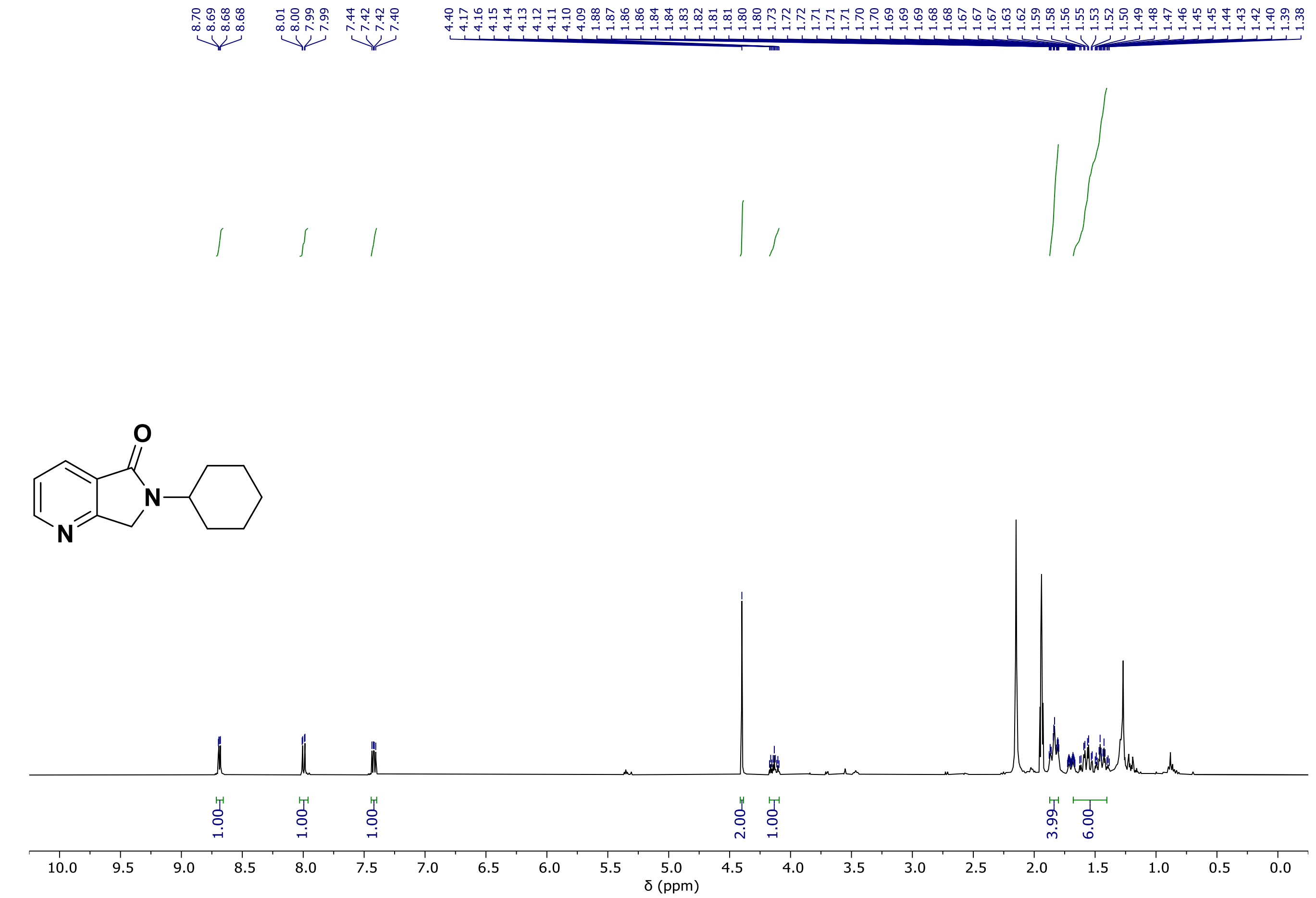

^{13}C NMR (Compound **24**)

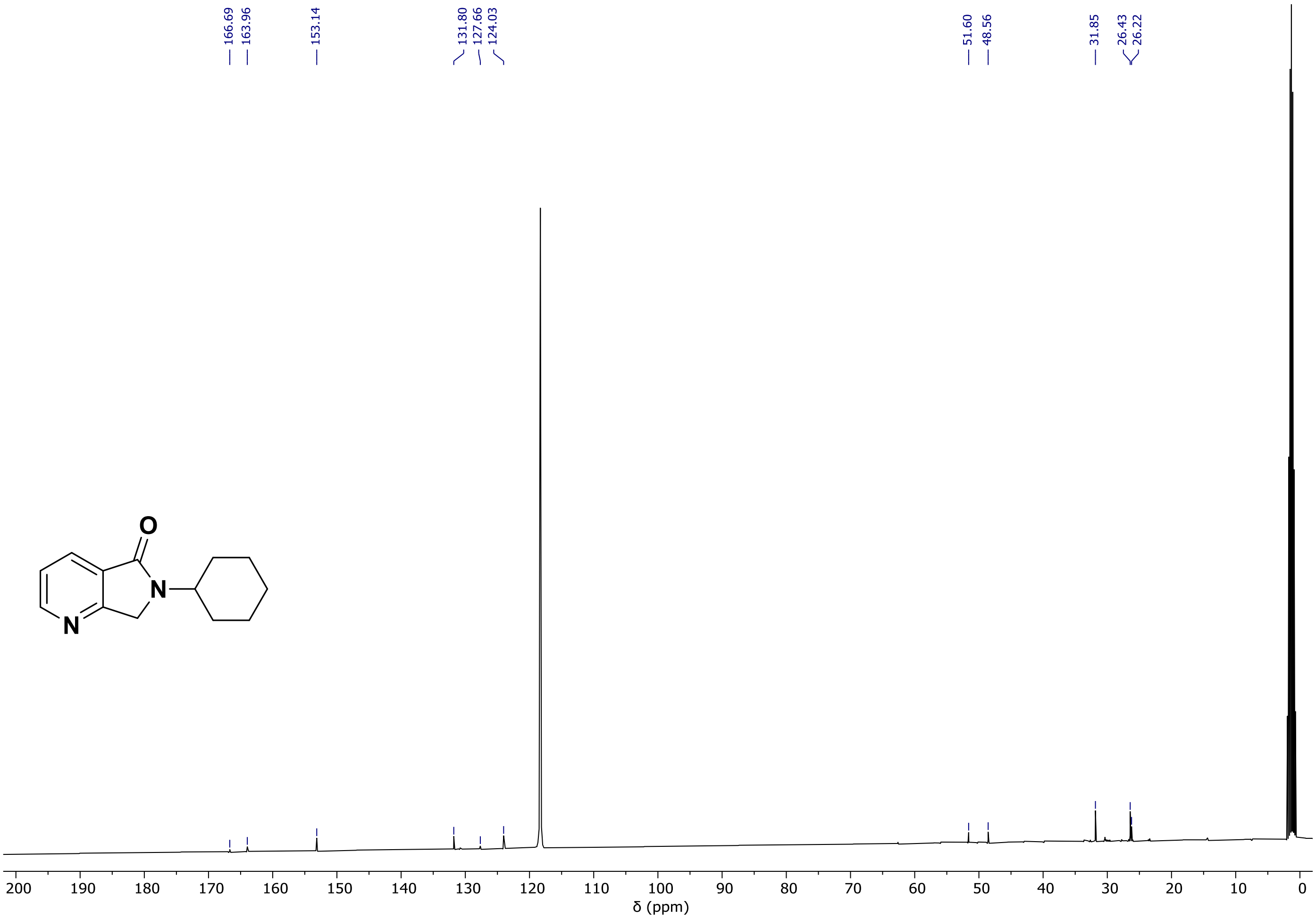

^{1}**H NMR** (Compound **26**)

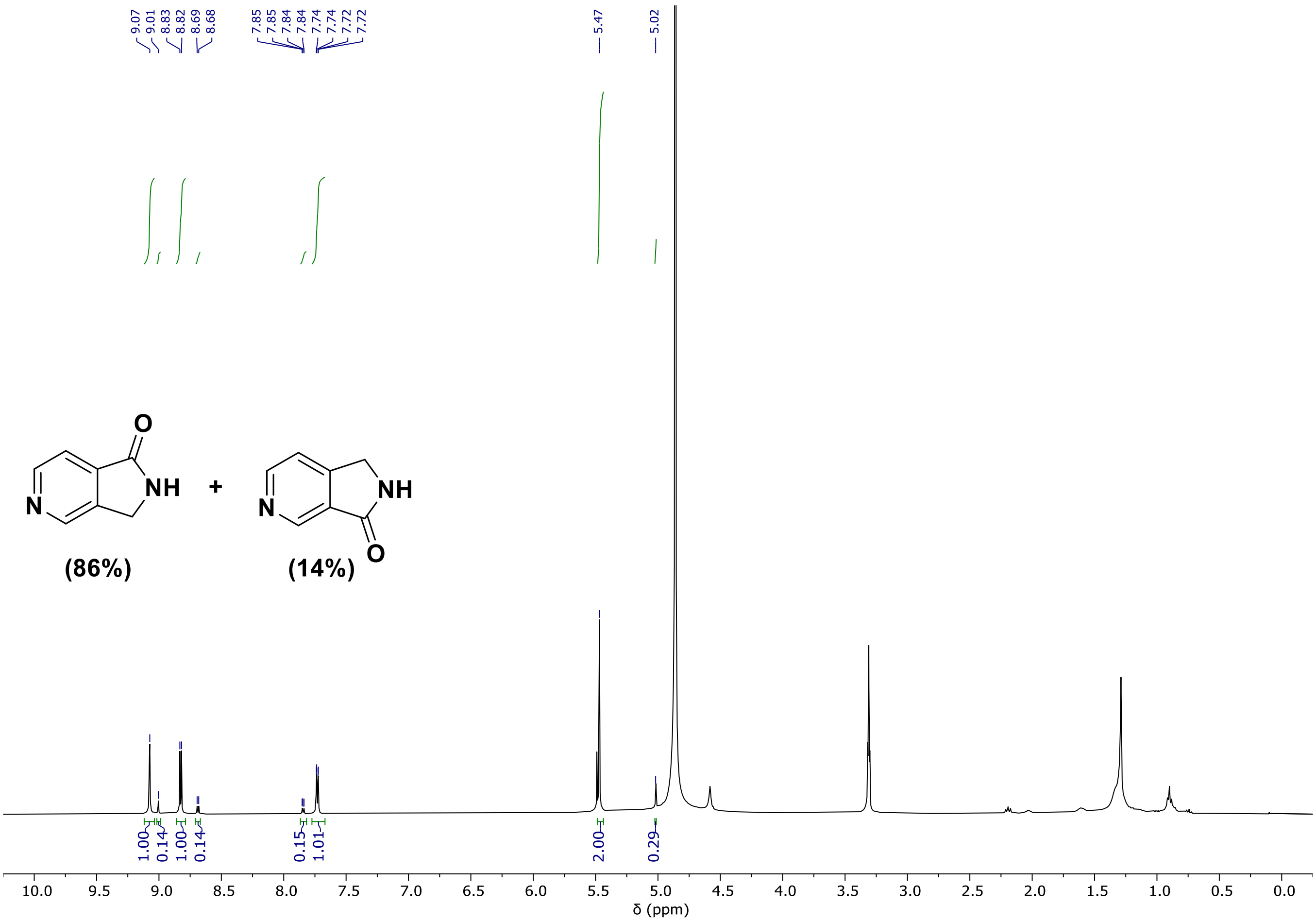

^{13}**C NMR** (Compound **26**)

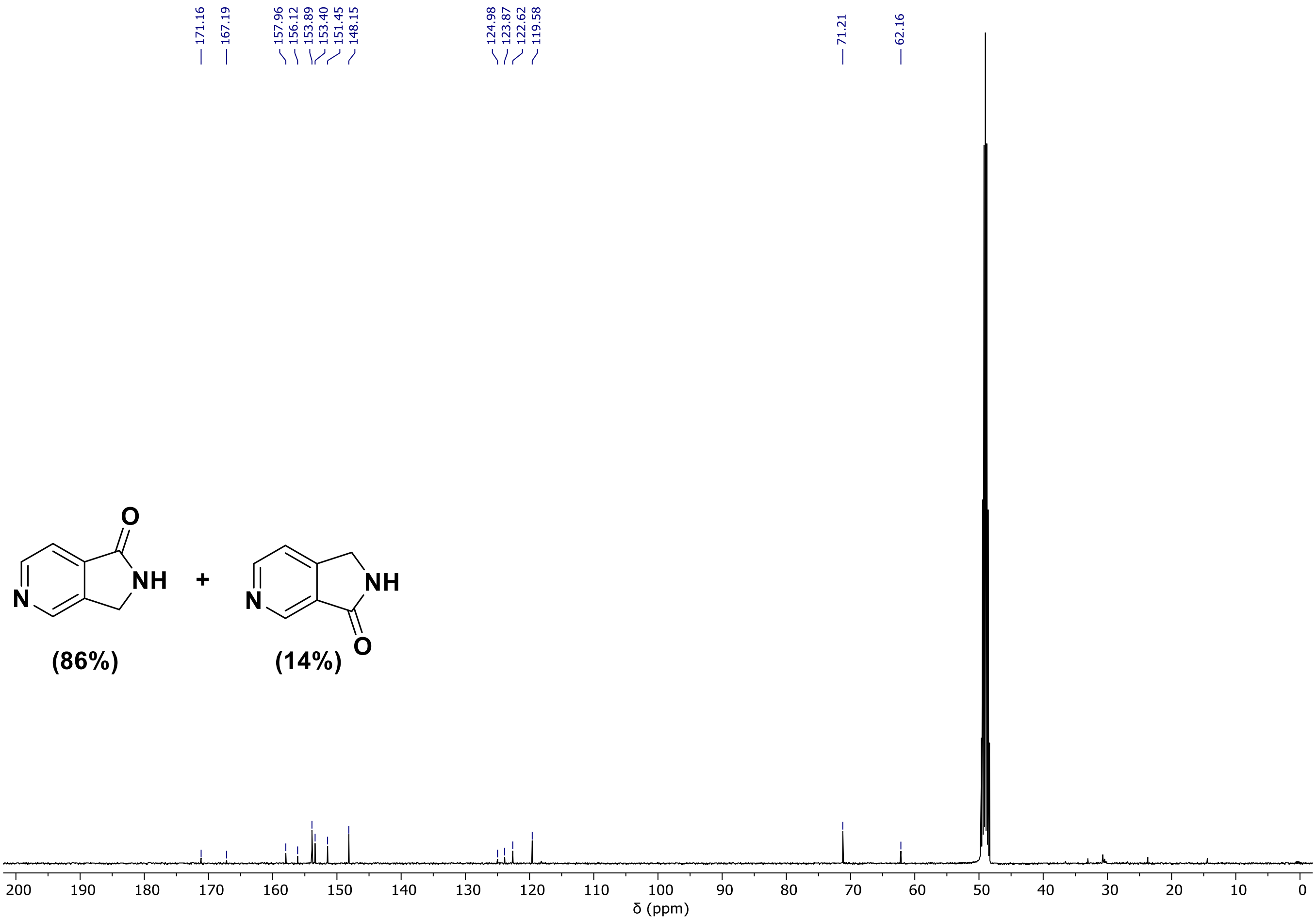

^{1}H – ^{1}H NOESY (Compound **26**)

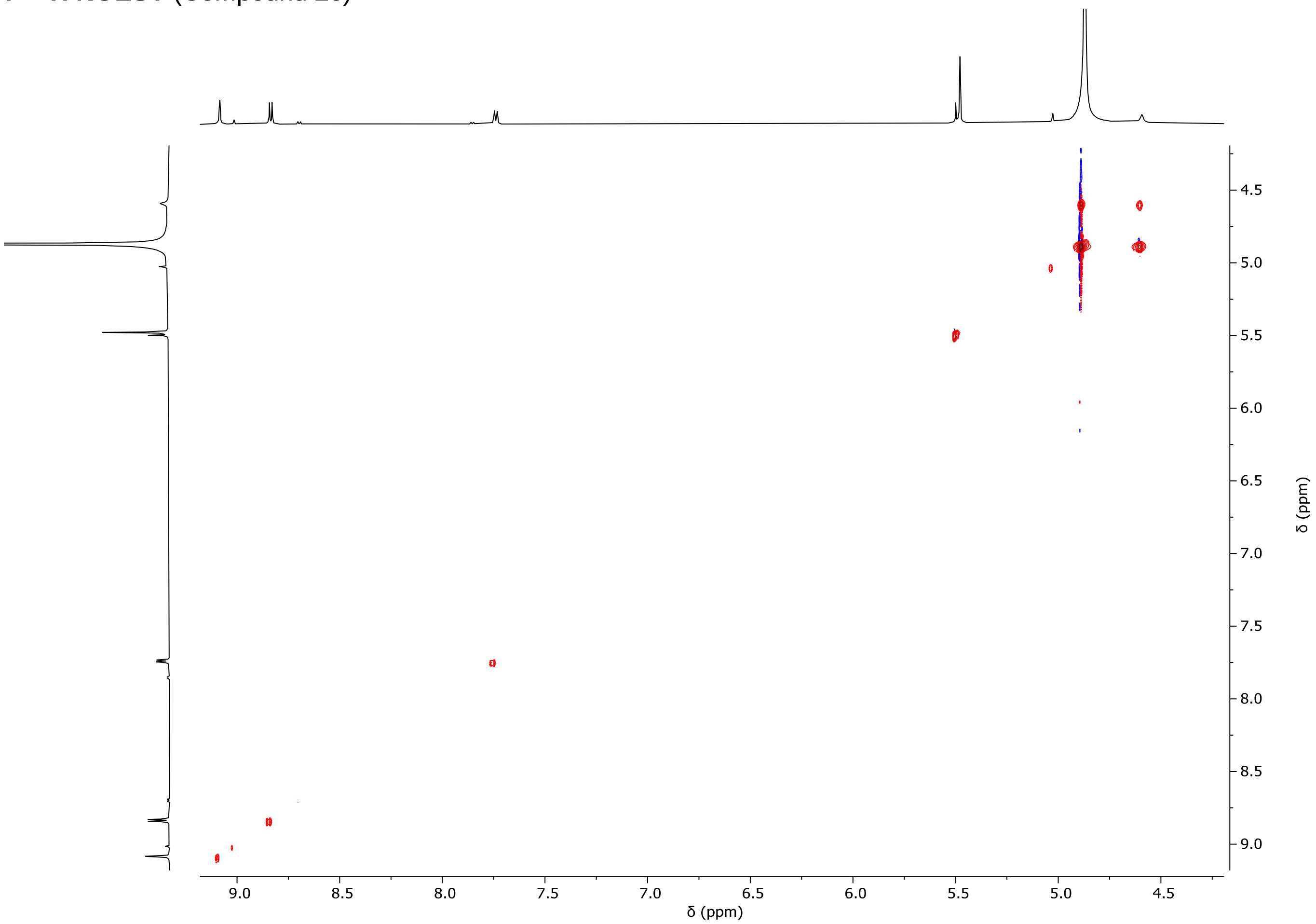

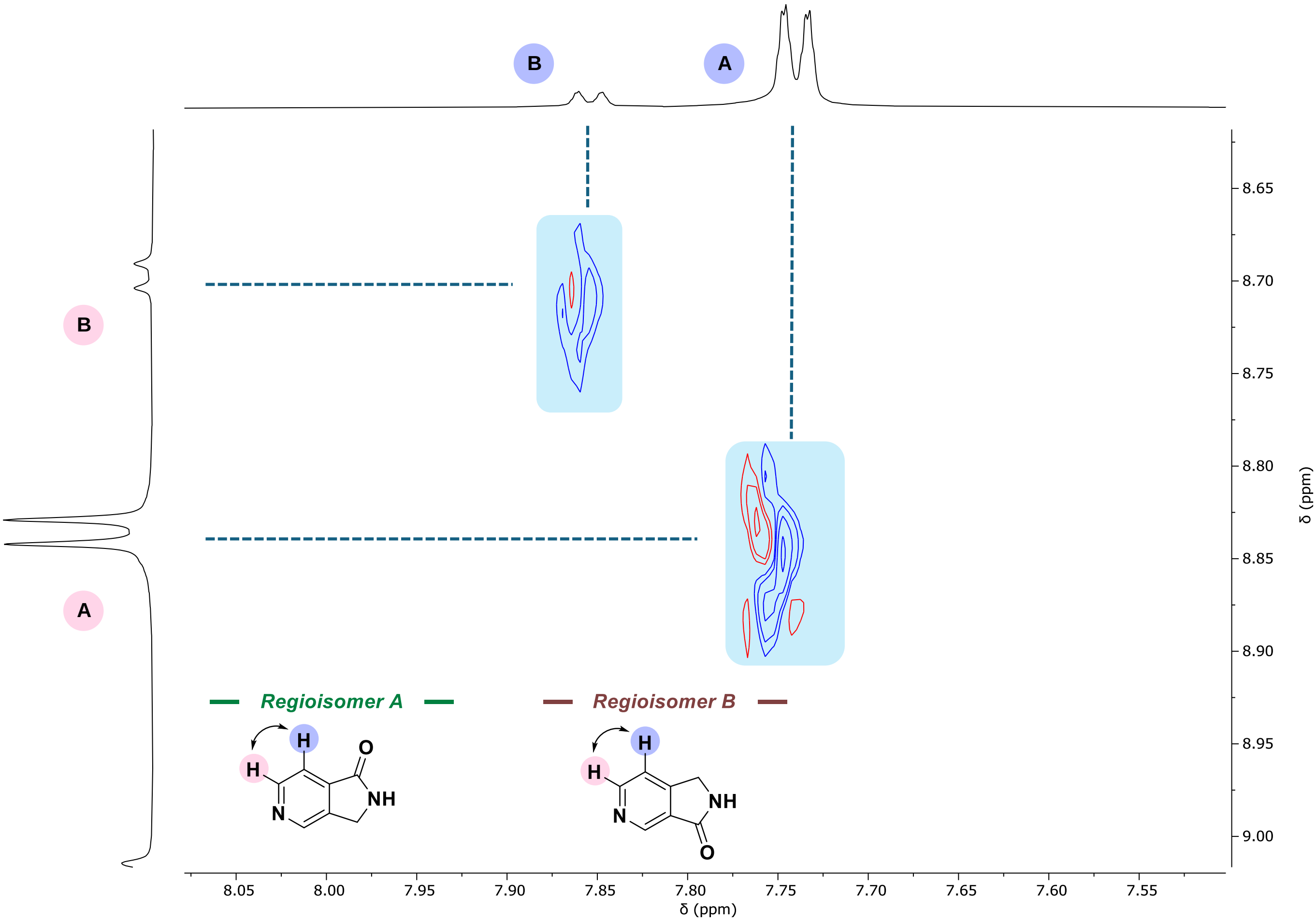

^1H – ^{13}C **HSQC** (Compound **26**)

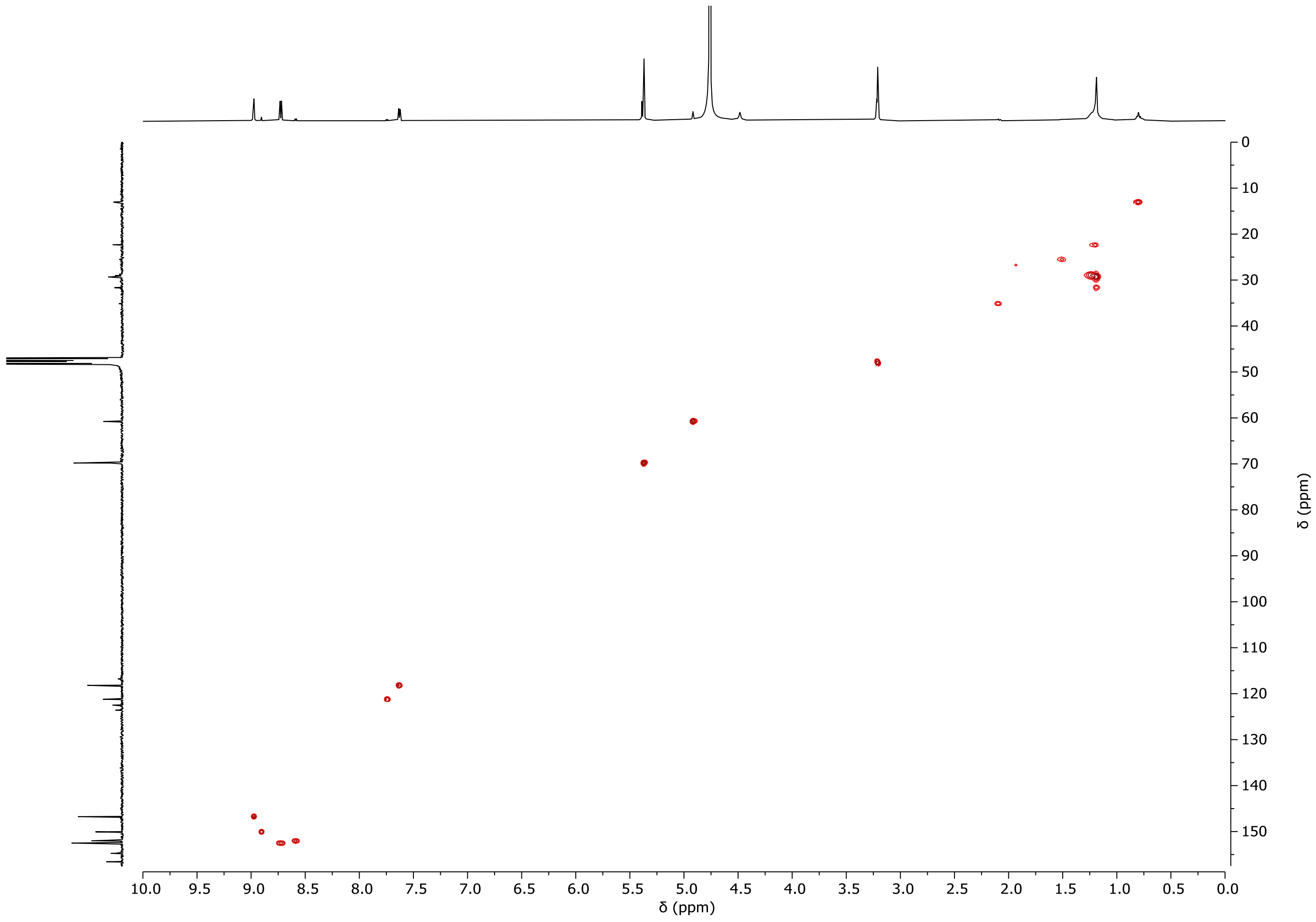

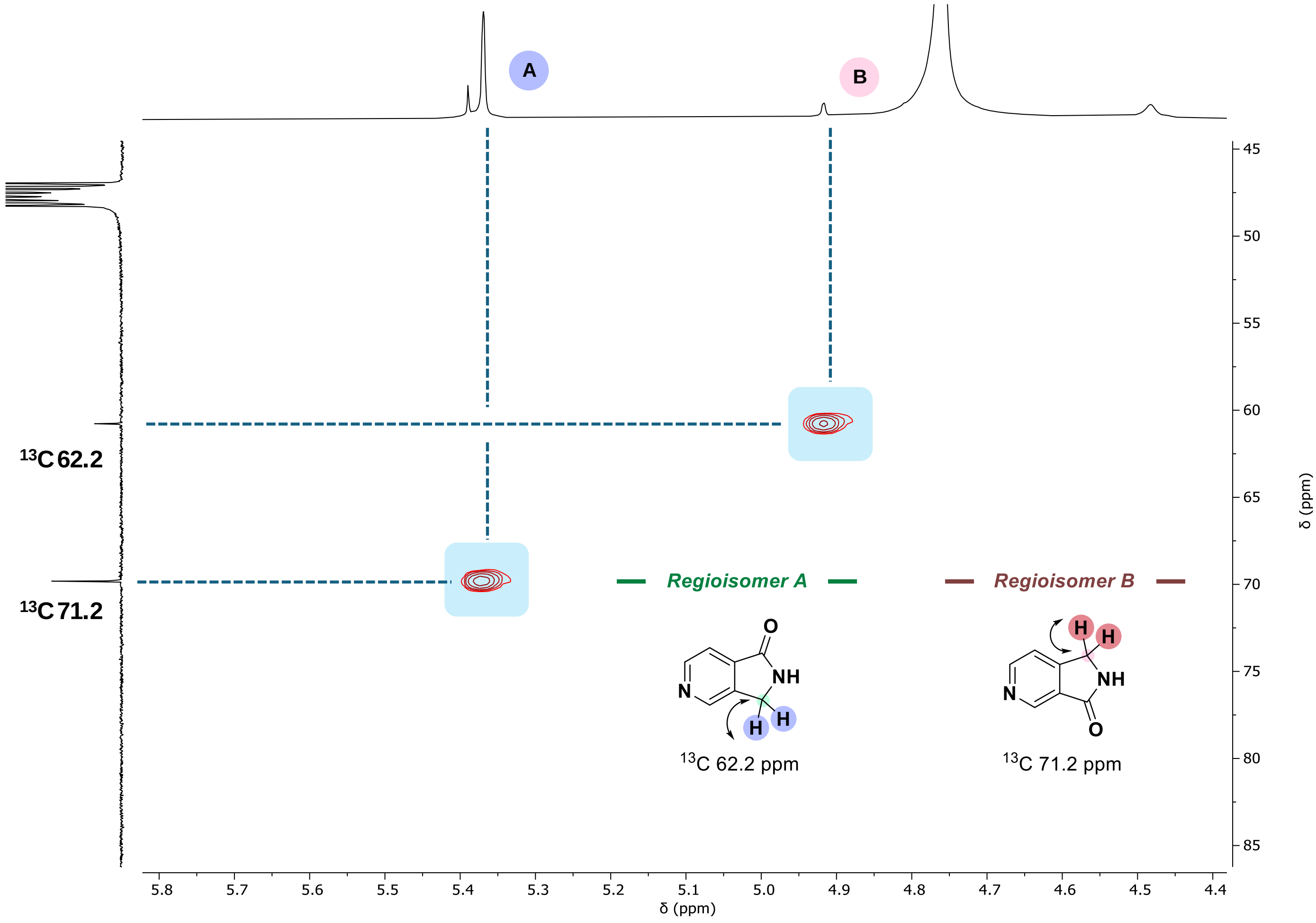

^{1}H – ^{13}C HMBC (Compound **26**)

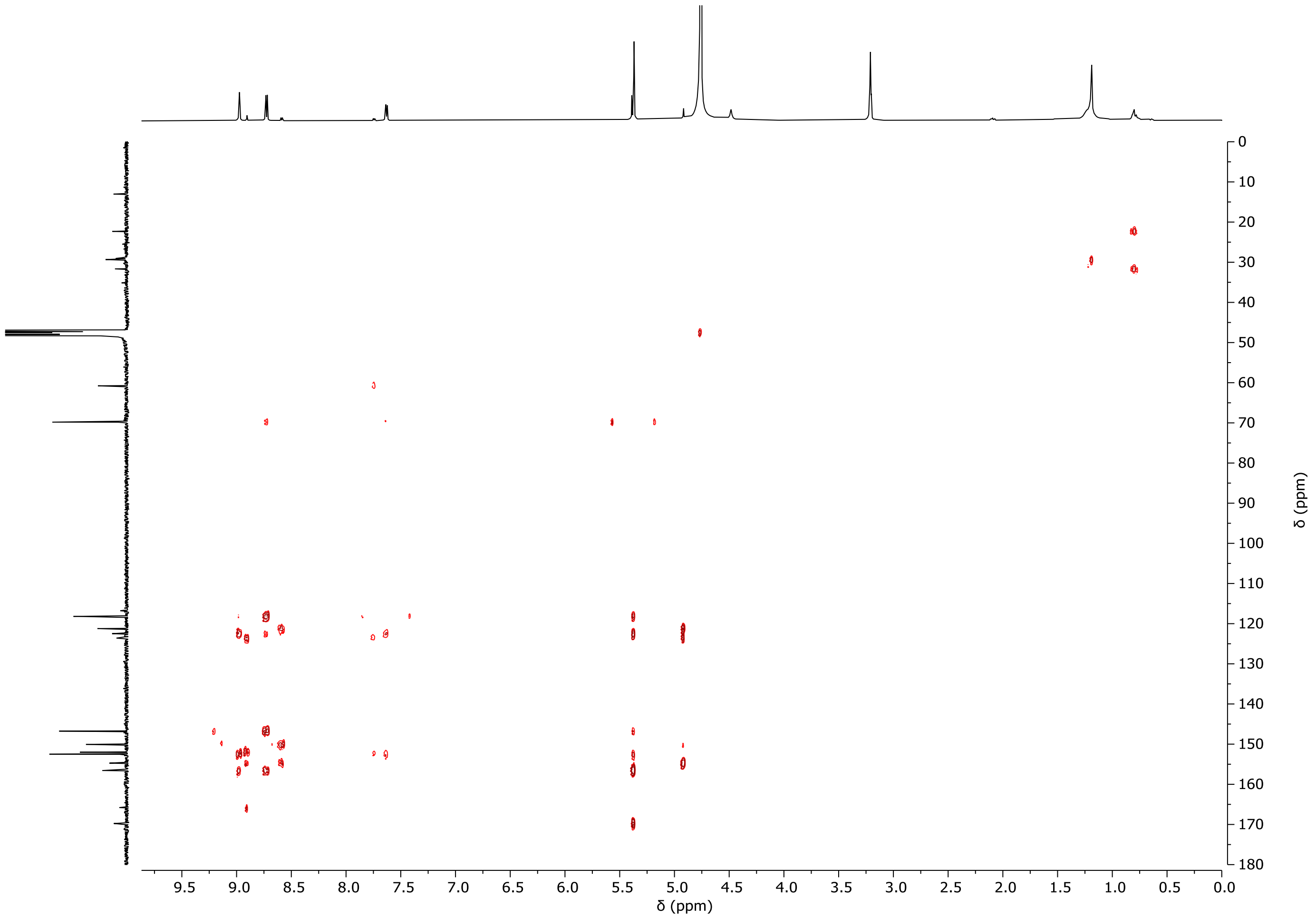

^{1}H – ^{13}C HMBC (signals assessment) (Compound **26**)

Major regioisomer ***Minor regioisomer***

2 Bonds distance

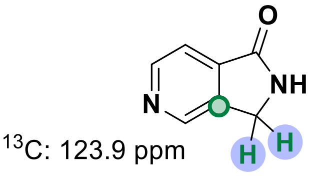

Seen in ^{1}H - ^{13}C HMBC

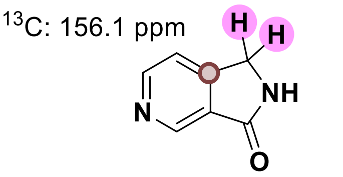

Seen in ^{1}H - ^{13}C HMBC

3 Bonds distance

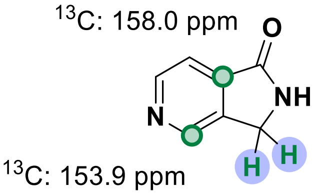

Seen in ^{1}H - ^{13}C HMBC

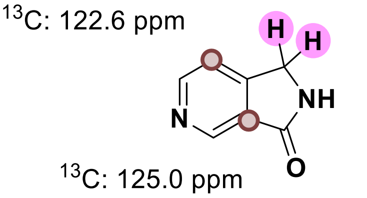

Seen in ^{1}H - ^{13}C HMBC

4 Bonds distance

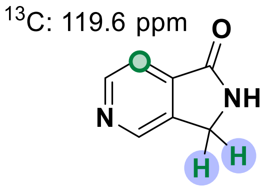

Seen in ^{1}H - ^{13}C HMBC

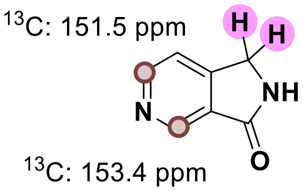

Not seen in ^{1}H - ^{13}C HMBC
